# Asymmetric Total Syntheses, Stereostructures, and Cytotoxicities of Marine Bromotriterpenoids Aplysiol B (Laurenmariannol) and Saiyacenol A

**DOI:** 10.1002/asia.202101137

**Published:** 2021-11-16

**Authors:** Kento Nishikibe, Keisuke Nishikawa, Momochika Kumagai, Matsumi Doe, Yoshiki Morimoto

**Affiliations:** ^1^ Department of Chemistry Graduate School of Science Osaka City University Sumiyoshi-ku Osaka 558–8585 Japan; ^2^ Faculty of Fisheries Kagoshima University Shimoarata Kagoshima 8900056 Japan

**Keywords:** configuration determination, cytotoxicity, natural products, structure elucidation, total synthesis

## Abstract

There are marine cytotoxic bromotriterpenoids, named the thyrsiferol family that are structurally characterized by some tetrahydropyran (THP) and tetrahydrofuran (THF) rings. The thyrsiferol family belongs to natural products that are often difficult to determine their stereostructures even by the current, highly advanced spectroscopic methods, especially in acyclic systems including stereogenic tetrasubstituted carbon centers. In such cases, it is effective to predict and synthesize the possible stereostructures. Herein, to elucidate ambiguous stereostructures and unassigned absolute configurations of aplysiol B, laurenmariannol, and saiyacenol A, members of the thyrsiferol family, we carried out their asymmetric chemical syntheses featuring 6‐*exo* and 5‐*exo* oxacyclizations of epoxy alcohol precursors and 6‐*endo* bromoetherification of a bishomoallylic alcohol. In this paper, we report total assignments of their stereostructures through their asymmetric chemical syntheses and also their preliminary cytotoxic activities against some tumor cells. These results could not have been achieved without depending on asymmetric total synthesis.

## Graphical abstract

**Total assignments** of stereostructures of marine cytotoxic bromotriterpenoids, aplysiol B (laurenmariannol) and saiyacenol A, were accomplished through their asymmetric chemical syntheses to elucidate their ambiguous stereostructures and whether or not other members showing the opposite chirality for the ABC ring system exist. Moreover, their preliminary growth inhibitory activities against some tumor cells were also evaluated.

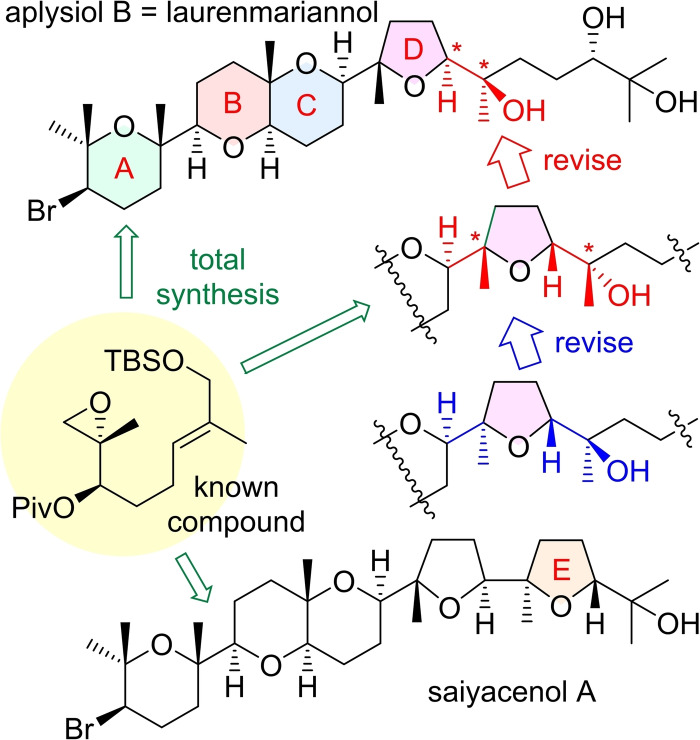

Aplysiol B with feeding‐deterrence and ichthyotoxicity properties, a marine bromotriterpenoid structurally related to the cytotoxic thyrsiferol family that possess as a partial structure a common dioxabicyclo[4.4.0]decane ring system with a bromine‐containing tetrahydropyranyl ring at C7 (ABC ring system),^[1]^ was isolated from the mantle of the sea hare *Aplysia dactylomela* by Manzo and co‐workers in 2007.^[2]^ The original structure **1** bearing *threo* configurations at C14−C15 and C18−C19 was determined based on NMR analysis and an integrated NMR‐QM (Quantum Mechanical) approach (Scheme 1). Afterwards, the original structure **1** was revised to a structure **2** bearing *erythro* configurations by Bowden and co‐workers in 2010 based on the biogenetic considerations from a squalene polyepoxide precursor.^[3]^ However, taking account of the biogenetic epoxide‐opening cascade triggered by a bromo cation as shown in **4**, we think the stereostructure at C18−C19 should be further revised to **3** because squalene tetraepoxide **5** has been proposed as a plausible biogenetic precursor for many triterpenoids.[^1a^, ^4^] The ambiguity of the stereostructure would be due to technical limitations of current spectroscopic methods for acyclic systems that include stereogenic tetrasubstituted carbon centers.^[5]^

**Scheme 1:**
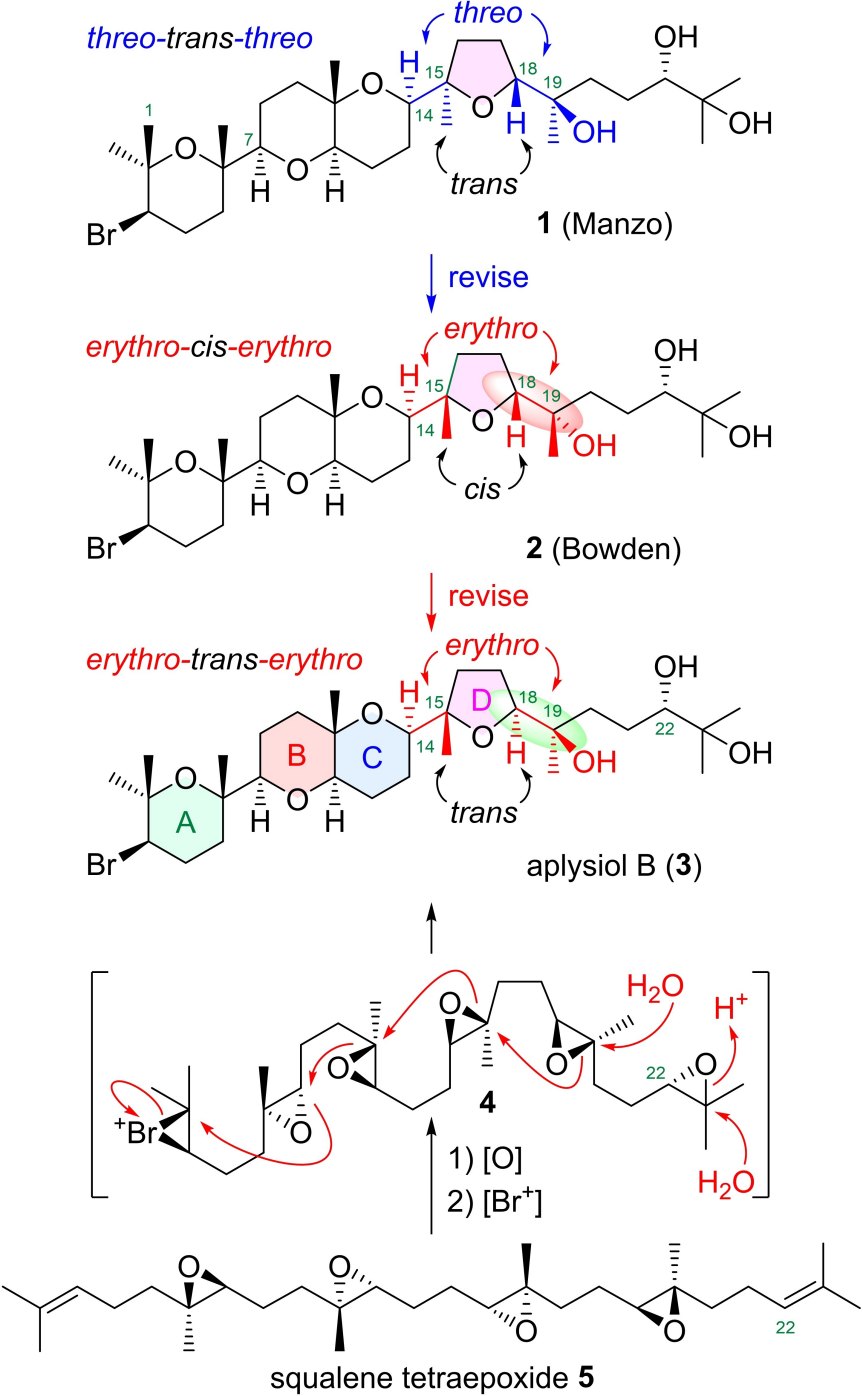
Proposed stereostructures and biosynthetic pathway of aplysiol B.

On the other hand, cytotoxic laurenmariannol (IC_50_=0.99 μM against P388 tumor cells) has also been isolated from the marine red alga *Laurencia mariannensis* by Wang and co‐workers in 2008 as a member of the thyrsiferol family.^[6]^ The original structure **6** with unknown stereochemistries at C19 and C22 was suggested by means of spectroscopic analyses (Figure 1). Saiyacenol A (**7**) exhibiting moderate cytotoxicities against some human tumor cells was isolated from the red alga *Laurencia viridis* by Fernández and co‐workers in 2012.^[7]^ Since the ABC ring system (C1−C14) in these compounds is common to the thyrsiferol family, it has been thought that the absolute configuration of the system is the same as that^[4a]^ of the first member thyrsiferol (**8**). However, we recently discovered that isodehydrothyrsiferol (**9**), a member of the thyrsiferol family, possesses the enantiomeric ABC ring system.^[4l]^ The phenomenon of enantiodivergence^[8]^ in the ABC ring system common to the thyrsiferol family would be of great interest in the fields of natural product chemistry and biosynthesis. Thus, to elucidate ambiguous stereostructures and unknown absolute configurations of aplysiol B, laurenmariannol, and saiyacenol A and whether or not other members showing the opposite chirality for the ABC ring system than **9** exist, we performed asymmetric chemical syntheses of these molecules.^[9]^ In this contribution, we report that the relative and absolute configurations of aplysiol B and saiyacenol A are shown in **3** and **7**, respectively, and spectroscopic data of aplysiol B (**3**) are identical to those of laurenmariannol through their asymmetric chemical syntheses. Further, preliminary cytotoxicities of synthetic compounds were also evaluated against some tumor cells.

**Figure 1:**
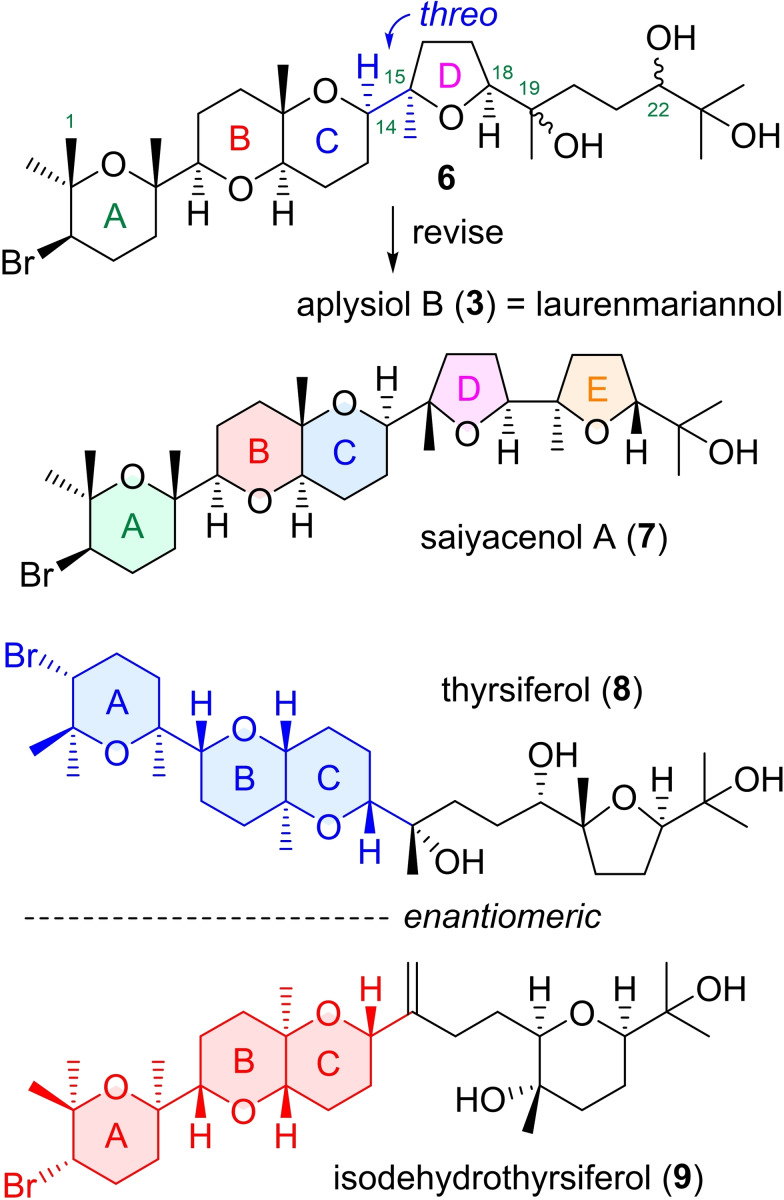
Proposed structures **6** and **7** for laurenmariannol and saiyacenol A, respectively, and enantiodivergency in the ABC ring system common to the thyrsiferol family.

The retrosynthetic analysis of our target structure **3** for aplysiol B as a representative of these compounds is indicated in Scheme 2. The A ring could be cyclized by bromoetherification from bishomoallylic alcohol **10**. The D ring would be formed from diene **11** by regioselective Shi epoxidation^[10]^ of a trisubstituted alkene at C18 followed by 5‐*exo* oxacyclization. Disconnection at the C16−C17 bond in **11** generates the known C_10_ unit **13** ^[11]^ and the common fused BC ring system **12**, both tetrahydropyran (THP) rings of which could be constructed by 6‐*exo* oxacyclization from the corresponding epoxy alcohol.^[4l]^

**Scheme 2:**
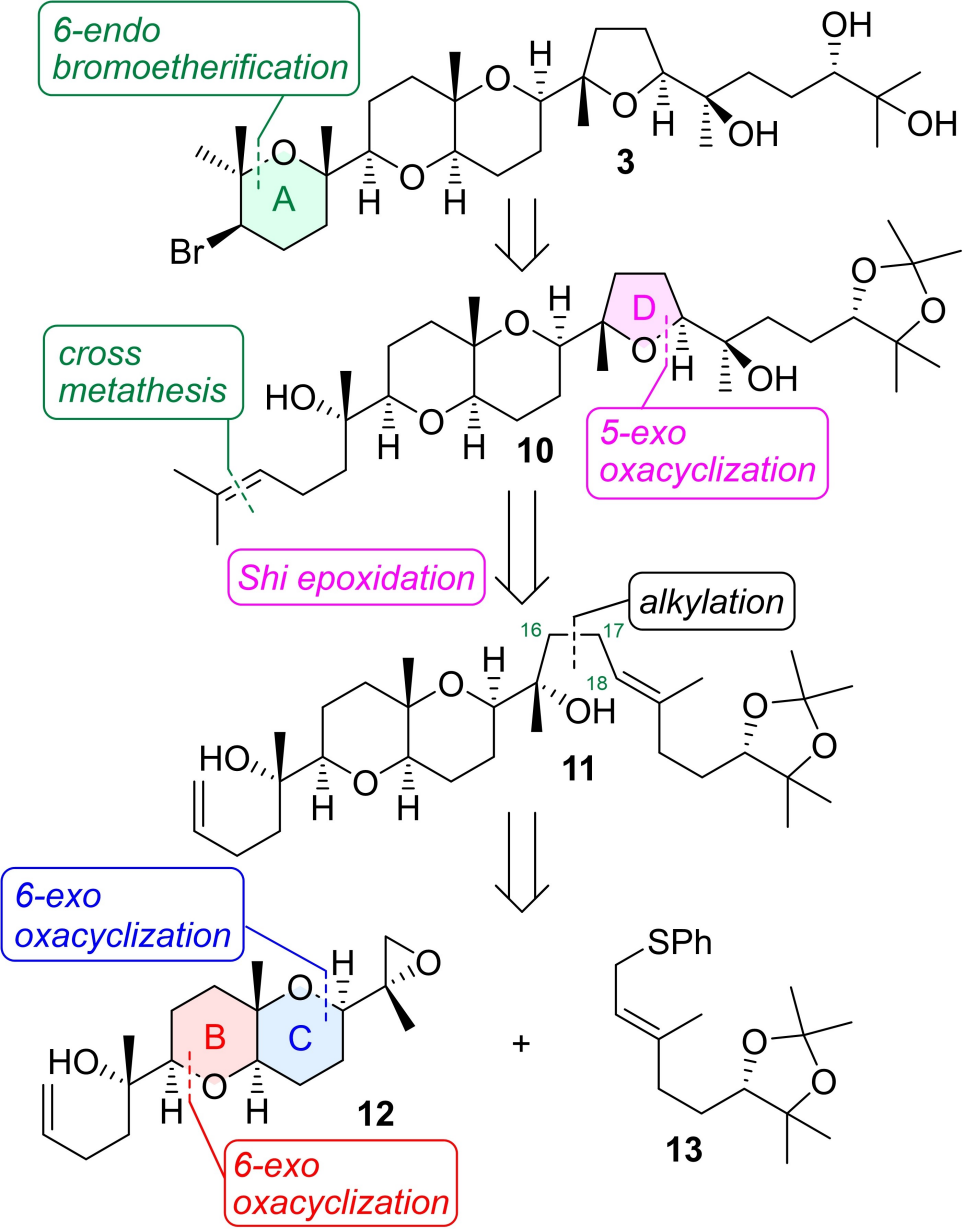
Retrosynthetic analysis of target structure **3**.

We embarked on the asymmetric chemical synthesis of the target molecule **3** with known compounds chiral epoxide **14** ^[4l]^ and allylic sulfide **15** ^[12]^ (Scheme 3). The lithiation of **15** and alkylation of the lithio derivative with epoxide **14** were carried out in situ in the presence of TMEDA, and subsequent desulfurization and selective TES protection of the secondary hydroxy group afforded alcohol **16**. Diastereoselective epoxidation^[4b]^ of the bishomoallylic alcohol **16** provided epoxy alcohol **17** in 79% yield. An epoxy alcohol obtained by manipulation of protecting groups was treated with KO*t*‐Bu in DMSO to give THP product **18** ^[13]^ in a 6‐*exo* selective manner and quantitative yield. Demethoxymethylation of **18** yielded a triol, the stoichiometric Sharpless asymmetric epoxidation[^4a^, ^14^] of which was accompanied with stepwise elevation of the reaction temperature^[4l]^ to successfully achieve 6‐*exo* oxacyclization, that led to the C ring adopting a twist‐boat conformation, in 79% yield. Selective mesylation of the primary hydroxy group in triol **19** ^[13]^ and the basic treatment furnished terminal epoxide **12**. Attachment of the C_10_ unit **13** ^[11]^ to the terminal epoxide **12** provided diene **11**, wherein Shi asymmetric epoxidation using D‐ketone **20** ^[10a]^ regioselectively proceeded for the trisubstituted double bond to afford epoxy alcohol **21** in high yield. Construction of the D ring was performed in 96% yield by 5‐*exo* oxacyclization of the epoxy alcohol **21** with CSA. Cross‐metathesis of the terminal olefin **22** ^[13]^ with Grubbs second generation catalyst **23** ^[15]^ generated a trisubstituted alkene, and bromoetherification of the resulting bishomoallylic alcohol **10** with bromodiethylsulfonium bromopentachloroantimonate (BDSB)^[16]^ in MeNO_2_ gave the desired 6‐*endo* product **24** ^[13]^ along with 5‐*exo* byproduct **25** ^[13]^ and recovered **10**. Finally, removal of an acetonide protecting group in **24** yielded our target structure **3**. The ^1^H‐ and ^13^C‐NMR spectra of synthetic **3**, [α]^28^_D_ −7.8 (*c* 0.12, CHCl_3_), were identical to those of the natural product, [α]^27^_D_ −9.0 (*c* 0.7, CHCl_3_),^[2]^ kindly provided by Manzo. Surprisingly, it was also found that the spectral data of synthetic **3** are consistent with those reported for laurenmariannol, [α]^18^_D_ −15.7 (*c* 0.41, CHCl_3_).^[6]^

**Scheme 3:**
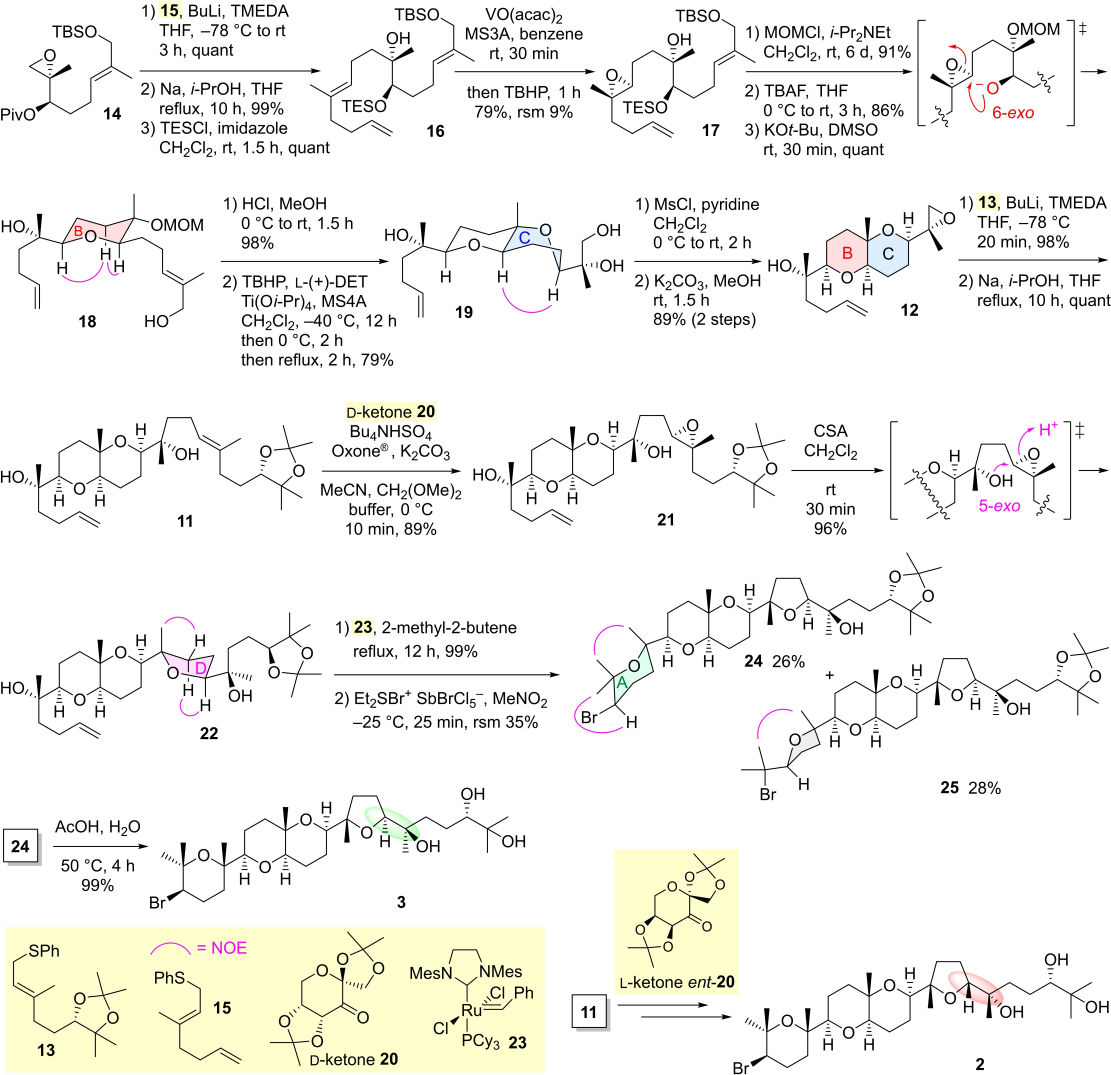
Asymmetric chemical syntheses of our target structure **3** and Bowden structure **2**. Piv=pivaloyl, TBS=*t*‐butyldimethylsilyl, TMEDA=*N*,*N*,*N'*,*N'*‐tetramethylethylenediamine, THF=tetrahydrofuran, TES=triethylsilyl, MS=molecular sieves, TBHP=*t*‐butyl hydroperoxide, rsm=recovered starting material, MOM=methoxymethyl, TBAF=tetrabutylammonium fluoride, DMSO=dimethyl sulfoxide, DET=diethyl tartrate, Ms=methanesulfonyl, CSA=(±)‐10‐camphorsulfonic acid, Mes=mesityl.

To confirm that the structure **2** proposed by Bowden et al. is not that of aplysiol B, we intended to synthesize the structure **2** bearing another *erythro* configuration at C18−C19 different from that of **3** as well. The synthesis of **2** was carried out in the same way as that from the synthetic intermediate **11** to **3** except for Shi asymmetric epoxidation of **11** using L‐ketone *ent*‐**20** ^[10b]^ instead of **20** (Scheme 3). Predictably, it was confirmed that the spectral data (^1^H‐ and ^13^C‐NMR) of synthetic **2** are inconsistent with those reported for the natural products aplysiol B^[2]^ and laurenmariannol.^[6]^ Thus, it has been revealed that aplysiol B and laurenmariannol are the same compound and the correct stereostructure is not **1**, **2**, and **6** but **3**.

Next, we commenced the asymmetric chemical synthesis of the stereostructure **7** proposed for another target saiyacenol A. The synthetic intermediate **12** to aplysiol B (**3**) was extended to diene **26** by alkylating with the known C_10_ unit *ent*‐**13**,^[12]^ an enantiomer of allylic sulfide **13**, followed by desulfurization (Scheme 4). Deprotection of an acetonide group in **26** and epoxidation of the resulting vicinal diol afforded a (22*S*)‐epoxide, which was subjected to regioselective Shi asymmetric epoxidation using D‐ketone **20** at C18 alkene to yield a double cyclization precursor diepoxide **27**. The double oxacyclization^[4h]^ of the diepoxy alcohol **27** smoothly proceeded in a 5‐*exo* selective manner to provide the desirable bisTHF product **28** ^[13]^ in a quantitative yield. The construction of the A ring was performed through cross‐metathesis of the terminal olefin **28** and subsequent bromoetherification by BDSB to accomplish the chemical synthesis of the target structure **7**.^[13]^ The spectral data (^1^H‐ and ^13^C‐NMR) of synthetic **7**, [α]^25^_D_ +1.6 (*c* 0.12, CHCl_3_), were identical to those reported for saiyacenol A, [α]^25^_D_ +1.53 (*c* 0.37, CHCl_3_).^[7]^ Thus, it was confirmed that the absolute configuration of saiyacenol A is shown as **7**.

**Scheme 4:**
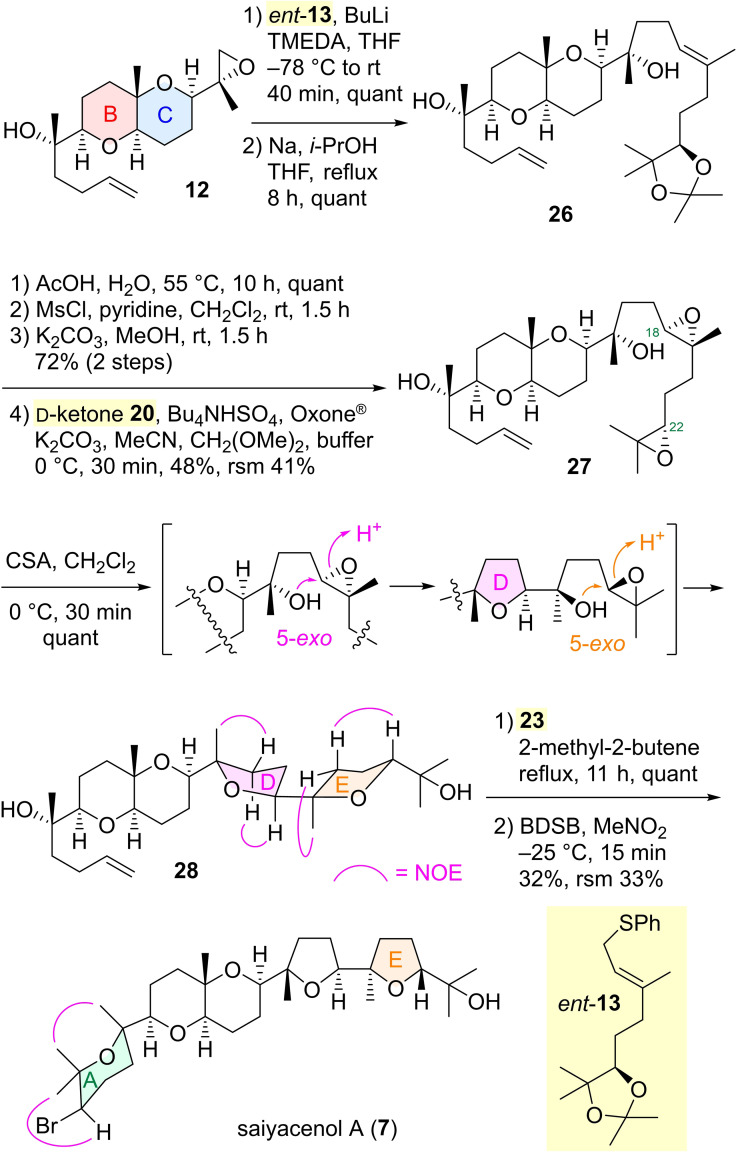
Asymmetric chemical synthesis of saiyacenol A (**7**).

With these synthetic aplysiol B (**3**), compound **2**, and saiyacenol A (**7**) in hand, we evaluated their cell growth inhibitory activities against some tumor cell lines. The results were shown in terms of IC_50_ (50% inhibitory concentration) values against P388, HT‐29, and HeLa (Table 1). All three compounds specifically indicated stronger activity for P388 than that for HT‐29 and HeLa. The synthetic aplysiol B (**3**) which was a naturally occurring compound inhibited cell growth stronger than compound **2** on every cell line. These results suggested the importance of 18*S* and 19*R* stereochemistries on biological activities. The activity of synthetic saiyacenol A (**7**) was lower than that of synthetic aplysiol B (**3**) in all cell lines, indicating that the formation of THF ring (E ring) reduces the biological activity.

**Table 1:** In vitro growth inhibitory activities of synthetic compounds **3**, **2**, and **7** on tumor cell lines.

Compound	IC_50_ [μM]		
	P388	HT‐29	HeLa
**3**	0.10 (0.99)^[a]^	31	51
**2**	2.0	81	>100
**7**	5.4	85	>100 (27.5)^[a]^

[a] Numerals in parentheses are IC_50_ (μM) values cited from ref. [6] for natural **3** and ref. [7] for natural **7**.

In conclusion, we have accomplished the asymmetric total syntheses of marine cytotoxic bromotriterpenoids aplysiol B (**3**), laurenmariannol (**3**), and saiyacenol A (**7**) with ambiguous stereostructures and unknown absolute configurations and determined their correct relative and absolute configurations. In addition, we have also revealed that aplysiol B and laurenmariannol reported as different compounds possess the same stereostructure **3**. These results could not have been achieved without depending on asymmetric total synthesis. Exploration of other members exhibiting the ABC ring system enantiomeric to that of thyrsiferol (**8**), the first member of the thyrsiferol family, and more detailed cytotoxicities of synthetic compounds are under investigation in our laboratory.

## Experimental Section

Experimental procedures, spectroscopic data, and copies of ^1^H‐ and ^13^C‐NMR spectra are available in the Supporting Information (SI).

## Conflict of interest

The authors declare no conflict of interest.

## Supporting information

As a service to our authors and readers, this journal provides supporting information supplied by the authors. Such materials are peer reviewed and may be re‐organized for online delivery, but are not copy‐edited or typeset. Technical support issues arising from supporting information (other than missing files) should be addressed to the authors.

Supporting Information
