# Supplementary material for: Asymmetric Total Syntheses, Stereostructures, and Cytotoxicities of Marine Bromotriterpenoids Aplysiol B (Laurenmariannol) and Saiyacenol A
Source: Chem Asian J. 2021 Nov 16;17(1):e202101137. doi: 10.1002/asia.202101137 (PMC9299038; doi:10.1002/asia.202101137)

CHEMISTRY
AN ASIAN JOURNAL

Supporting Information

Asymmetric Total Syntheses, Stereostructures, and Cytotoxicities of Marine Bromotriterpenoids Aplysiol B (Laurenmariannol) and Saiyacenol A

Kento Nishikibe, Keisuke Nishikawa,* Momochika Kumagai, Matsumi Doe, and Yoshiki Morimoto*© 2021 The Authors. Chemistry - An Asian Journal published by Wiley-VCH GmbH. This is an open access article under the terms of the Creative Commons Attribution License, which permits use, distribution and reproduction in any medium, provided the original work is properly cited.

Table of Contents

Experimental procedures: 3–21
General procedures: 3
Synthesis of Bowden structure 2: 9
Table S1 (^{1}H-NMR data of synthetic aplysiol B (3), synthetic Bowden structure 2, natural aplysiol B (3), and natural laurenmariannol (3)): 12
Table S2 (^{13}C-NMR data of synthetic aplysiol B (3), synthetic Bowden structure 2, natural aplysiol B (3), and natural laurenmariannol (3)): 13
Table S3 (^{1}H-NMR data of synthetic and natural saiyacenol A (7)): 17
Table S4 (^{13}C-NMR data of synthetic and natural saiyacenol A (7)): 18
Biological experimental procedure and data: 18–21
References: 22
Author contributions: 22

Spectral data: 23–68
^{1}H- and ^{13}C-NMR spectra of sulfide S1: 23
^{1}H- and ^{13}C-NMR spectra of diol S2: 24
^{1}H- and ^{13}C-NMR spectra of TES ether 16: 25
^{1}H- and ^{13}C-NMR spectra of epoxide 17: 26
^{1}H- and ^{13}C-NMR spectra of MOM ether S3: 27
^{1}H- and ^{13}C-NMR spectra of diol S4: 28
^{1}H- and ^{13}C-NMR spectra of THP 18: 29
NOESY spectrum of THP 18: 30
^{1}H- and ^{13}C-NMR spectra of triol S5: 31
^{1}H- and ^{13}C-NMR spectra of triol 19: 32
NOESY spectrum of triol 19: 33
^{1}H- and ^{13}C-NMR spectra of epoxide 12: 34
^{1}H- and ^{13}C-NMR spectra of sulfide S6: 35
^{1}H- and ^{13}C-NMR spectra of diol 11: 36
^{1}H- and ^{13}C-NMR spectra of bishomoepoxy alcohol 21: 37
^{1}H- and ^{13}C-NMR spectra of THF 22: 38
NOESY spectrum of THF 22: 39
^{1}H- and ^{13}C-NMR spectra of alkene 10: 40
^{1}H- and ^{13}C-NMR spectra of THP 24: 41
NOESY spectrum of THP 24: 42
^{1}H- and ^{13}C-NMR spectra of THF 25: 43
NOESY spectrum of THF 25: 44
^{1}H- and ^{13}C-NMR spectra of aplysiol B (3) = laurenmariannol: 45
NOESY spectrum of aplysiol B (3) = laurenmariannol: 46
^{1}H- and ^{13}C-NMR spectra of bishomoepoxy alcohol S7: 47
^{1}H- and ^{13}C-NMR spectra of THF S8: 48
NOESY spectrum of THF S8: 49
^{1}H- and ^{13}C-NMR spectra of alkene S9: 50
^{1}H- and ^{13}C-NMR spectra of THP S10: 51
NOESY spectrum of THP S10: 52
^{1}H- and ^{13}C-NMR spectra of THF S11: 53
NOESY spectrum of THF S11: 54
^{1}H- and ^{13}C-NMR spectra of Bowden structure 2: 55
NOESY spectrum of Bowden structure 2: 56
^{1}H- and ^{13}C-NMR spectra of sulfide S12: 57
^{1}H- and ^{13}C-NMR spectra of diol 26: 58
^{1}H- and ^{13}C-NMR spectra of tetraol S13: 59
^{1}H- and ^{13}C-NMR spectra of epoxide S14: 60
^{1}H- and ^{13}C-NMR spectra of diepoxide 27: 61
^{1}H- and ^{13}C-NMR spectra of bis-THF 28: 62
NOESY spectrum of bis-THF 28: 63
^{1}H- and ^{13}C-NMR spectra of alkene S15: 64
^{1}H- and ^{13}C-NMR spectra of saiyacenol (7): 65

NOESY spectrum of saiyacenol (7): 66
^{1}H- and ^{13}C-NMR spectra of THP S16: 67
NOESY spectrum of THP S16: 68

Experimental procedures

General procedures. ^{1}H-NMR spectra were recorded in deuteriochloroform on JEOL JNM-ECZ400S or Bruker Biospin Avance III HD 400 (400 MHz) and Bruker Biospin Avance III HD 600 (600 MHz) spectrometers. ^{13}C-NMR spectra were measured in deuteriochloroform on JEOL JNM-ECZ400S or Bruker Biospin Avance III HD 400 (100 MHz) and Bruker Biospin Avance III HD 600 (150 MHz) spectrometers. Chemical shifts were reported in parts per million (ppm) from tetramethylsilane with the solvent resonance as an internal standard ($CDCl_3$: 7.26 ppm for ^{1}H-NMR, 77.0 ppm for ^{13}C-NMR). Splitting patterns were designated as "s, d, t, q, and m" to indicate "singlet, doublet, triplet, quartet, and multiplet," respectively. IR spectra were recorded on a JASCO FT/IR-4100 spectrophotometer by the attenuated total reflection (ATR), unless otherwise noted. Low- and high-resolution mass spectra were obtained on a JEOL AccuTOF LC-plus JMS-T100LP (ESI and DART) spectrometer. Optical rotations were determined on a JASCO DIP-370 digital polarimeter. Analytical TLC was carried out by precoated silica gel (Merck TLC plates silica gel 60 F254). Flash column chromatographies were performed with Merck silica gel 60 (particle size 63–200 µm), Wakogel® 60N (particle size 38–100 µm), and KANTO silica gel 60N (particle size 40–50 µm), respectively. All reactions were performed in oven-dried glassware. Tetrahydrofuran (THF) was distilled over sodium metal/benzophenone ketyl. Acetonitrile (MeCN), benzene, dichloromethane (CH_2Cl_2), dimethyl sulfoxide (DMSO), 2-propanol (*i*-PrOH), pyridine, *N*,*N*,*N*′,*N*′-tetramethylethylenediamine (TMEDA), and triethylamine (NEt_3) were distilled over calcium hydride under normal or reduced pressure. Methanol (MeOH) was distilled from $Mg(OMe)_2$. Activation of powdered 3 and 4 Å molecular sieves (MS3A and MS4A) involved heating in a vacuum oven at 160 °C and 0.05 mmHg pressure for at least 3 h. Nitromethane ($MeNO_2$) was dried over activated MS4A.

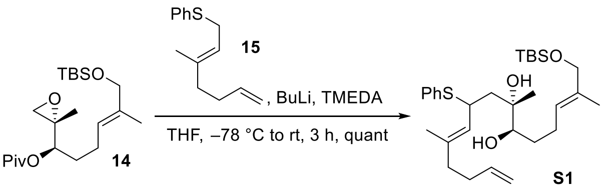

Sulfide S1. To a solution of allylic sulfide **15**[1] (2.84 g, 13.0 mmol) and TMEDA (4.91 mL, 32.5 mmol) in THF (25 mL) was added *n*-BuLi (10.0 mL, 13.0 mmol, 1.56 M in hexane) at –78 °C under a nitrogen atmosphere, and the mixture was stirred for 1 h. To the solution was added dropwise a solution of epoxide **14**[2] (833 mg, 2.17 mmol) in THF (6.0 mL), and the mixture was stirred for 1 h. After the solution was stirred at room temperature for additional 2 h, H_2O was added to the solution, and the mixture was extracted with Et_2O (× 3). The organic layers were washed with brine, dried over anhydrous Na_2SO_4, filtered, and concentrated under reduced pressure. The residue was subjected to flash column chromatography (EtOAc/hexane, 10:90 to 15:85) on silica gel to yield **S1** (1.12 g, 2.16 mmol, quant) as a mixture of diastereomeric sulfides and a colorless oil: R_f = 0.68 (EtOAc/hexane, 30:70); ^{1}H-NMR (400 MHz, $CDCl_3$) δ 7.45–7.41 (2H, m), 7.27–7.24 (3H, m), 5.69 (1H, ddt, *J* = 17.2, 10.8, 6.4 Hz), 5.40 (1H, t, *J* = 7.4 Hz), 5.16 (1H, d, *J* = 10.0 Hz), 4.99–4.92 (2H, m), 4.19–4.09 (1H, m), 4.01 (2H, s), 3.35 (1H, d, *J* = 4.8 Hz), 2.56 (1H, br s), 2.39 (1H, br s), 2.32–1.93 (6H, m), 1.79 (1H, dd, *J* = 10.0, 4.2 Hz), 1.62 (3H, s), 1.51–1.34 (3H, m), 1.31 (3H, s), 1.14 (3H, s), 0.91 (9H, s), 0.06 (6H, s); ^{13}C-NMR (100 MHz, $CDCl_3$) δ 138.1, 135.2, 134.9, 134.6, 133.9, 129.1, 129.0, 128.8, 128.6, 128.3, 127.7, 127.0, 126.6, 124.1, 123.8, 115.1, 78.1, 75.3, 68.6, 43.8, 41.0, 40.8, 38.8, 31.9, 31.0, 30.9, 26.1, 24.9, 24.2, 18.5, 16.2, 16.1, 13.7, 13.5, –5.3; IR (ATR) 3431, 3077, 2935, 2865, 1461, 1378, 1276, 1102, 912, 747 cm^{-1}; ESI-HRMS calcd for $C_{30}H_{50}NaO_3SSi$ [$(M+Na)^+$] 541.3148, found 541.3155.

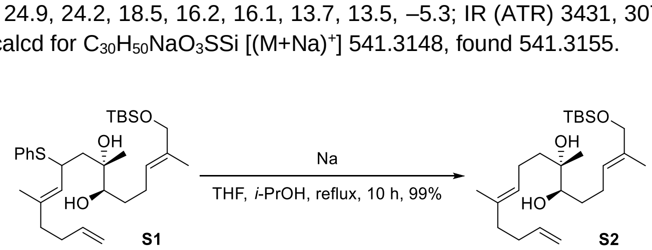

Diol S2. Sulfide **S1** (9.56 g, 18.4 mmol) was dissolved in THF (180 mL) and *i*-PrOH (60.0 mL) under a nitrogen atmosphere. Several pieces of metallic sodium (12.7 g, 553 mmol) was added portionwise to the boiling solution under reflux, and the resulting mixture was refluxed for 10 h. After the mixture was cooled to 0 °C, H_2O was added to the solution, and the mixture was extracted with Et_2O (× 3). The organic layers were washed with brine, dried over anhydrous Na_2SO_4, filtered, and concentrated under reduced pressure. The residue was purified by column chromatography (EtOAc/hexane, 5:95 to 10:90) on silica gel to afford **S2** (7.50 g, 18.3 mmol, 99%) as a colorless oil: R_f = 0.35 (EtOAc/hexane, 20:80); $[\alpha]^{20}_D$ +4.6 (*c* 1.0, $CHCl_3$); ^{1}H-NMR (400 MHz, $CDCl_3$) δ 5.79 (1H, ddt, *J* = 17.2, 10.0, 6.4 Hz), 5.42 (1H, td, *J* = 7.4, 1.2 Hz), 5.16 (1H, t, *J* = 7.2 Hz), 5.00 (1H, dq, *J* = 17.2, 1.7 Hz), 4.96–4.92 (1H, m), 4.01 (2H, s), 3.43–3.36 (1H, m), 2.33–2.24 (1H, m), 2.20–2.01 (7H, m), 1.77 (1H, br s), 1.70–1.50 (2H, m), 1.63 (6H, s), 1.48–1.34 (2H, m), 1.17 (3H, s), 0.91 (9H, s), 0.06 (6H, s); ^{13}C-NMR (100 MHz, $CDCl_3$) δ 138.4, 135.0, 134.7, 124.7, 123.8, 114.3, 78.0, 74.7, 68.4, 38.9, 35.7, 32.2, 31.0, 25.9, 24.7, 23.4, 21.9, 18.3, 15.9, 13.4, –5.4; IR (ATR) 3447, 2952, 2928, 2856, 1461, 1386, 1362, 1254, 1109, 1067, 1006, 939, 909, 836, 776 cm^{-1}; DART-HRMS calcd for $C_{24}H_{47}O_3Si$ [$(M+H)^+$] 411.3294, found 411.3283.

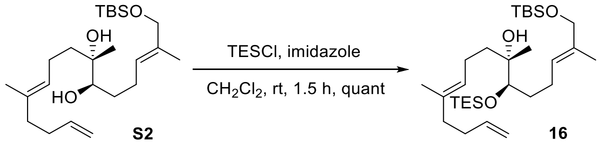

TES ether 16. To a solution of **S2** (7.50 g, 18.3 mmol) in CH_2Cl_2 (180 mL) were added imidazole (3.73 g, 54.8 mmol) and TESCl (4.26 mL, 27.4 mmol) at room temperature under a nitrogen atmosphere, and the solution was stirred for 1.5 h. H_2O was added to the solution, and the mixture was extracted with CH_2Cl_2 (× 3). The organic layers were washed with brine, dried over anhydrous Na_2SO_4, filtered, and concentrated under reduced pressure. The residue was purified by flash column chromatography (EtOAc/hexane, 5:95) on silica gel to furnish **16** (9.56 g, 18.2 mmol, quant) as a colorless oil: R_f = 0.49 (EtOAc/hexane, 10:90); $[\alpha]^{23}_D$ +3.8 (*c* 0.50, $CHCl_3$); ^{1}H-NMR (400 MHz, $CDCl_3$) δ 5.80 (1H, ddt, *J* = 16.8, 10.0, 6.4 Hz), 5.37 (1H, t, *J* = 7.2 Hz), 5.15 (1H, t, *J* = 6.8 Hz), 5.02–4.98 (1H, m), 4.93 (1H, dd, *J* = 10.0, 1.2 Hz), 4.00 (2H, s), 3.50 (1H, dd, *J* = 7.6, 3.2 Hz), 2.28–1.95 (8H, m), 1.66–1.36 (4H, m), 1.60 (6H, s), 1.12 (3H, s), 0.98 (9H, t, *J* = 7.8 Hz), 0.91 (9H, s), 0.66 (6H, q, *J* = 8.0 Hz), 0.06 (6H, s); ^{13}C-NMR (100 MHz, $CDCl_3$) δ 138.7, 134.7, 134.6, 125.0, 124.1, 114.3, 79.9, 74.6, 68.5, 39.0, 36.4, 32.7, 32.3, 25.9, 25.0, 23.7, 22.1, 18.5, 15.9, 13.5, 7.0, 5.4, –5.3; IR (ATR) 3467, 2956, 2930, 2878, 2856, 1460, 1254, 1106, 1070, 1009, 909, 835, 776, 741 cm^{-1}; DART-HRMS calcd for $C_{30}H_{61}O_3Si_2$ [(M+H)$^+$] 525.4159, found 525.4127.

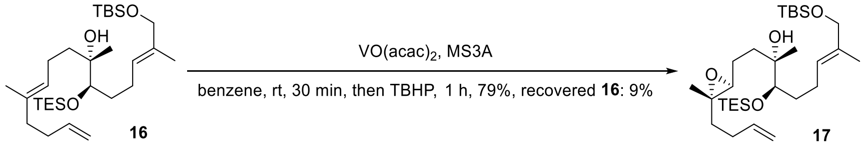

Epoxide 17.[3] To a solution of **16** (6.18 g, 11.8 mmol) in benzene (39 mL) were added MS3A (1.23 g, 200 wt%) and VO(acac)$_2$ (312 mg, 1.18 mmol) at room temperature under a nitrogen atmosphere, and the solution was stirred for 30 min. To the solution was slowly added dropwise TBHP (5.50 M in decane, 5.03 mL, 27.7 mmol), and the solution was stirred for 1 h. After filtration through a pad of Celite under reduced pressure, H_2O was added to the filtrate, and the mixture was extracted with CH_2Cl_2 (× 3). The organic layers were washed with a saturated aqueous solution of $NaHCO_3$, dried over anhydrous Na_2SO_4, filtered, and concentrated under reduced pressure. The residue was purified by flash column chromatography (EtOAc/hexane, 2:98 for **16** to 6:94 for **17**) on silica gel treated with a 1% solution of NEt_3 in hexane to afford **17** (5.06 g, 9.36 mmol, 79%) and recovered **16** (540 mg, 1.30 mmol, 9%) as each colorless oil. **17**: R_f = 0.60 (EtOAc/hexane, 20:80); $[\alpha]^{25}_D$ +2.4 (*c* 1.1, $CHCl_3$); ^{1}H-NMR (400 MHz, $CDCl_3$) δ 5.78 (1H, ddt, *J* = 16.8, 10.0, 6.4 Hz), 5.37–5.34 (1H, m), 5.00 (1H, dd, *J* = 15.2, 1.8 Hz), 4.94 (1H, dd, *J* = 10.4, 1.8 Hz), 4.00 (2H, s), 3.49 (1H, dd, *J* = 7.6, 3.0 Hz), 2.71 (1H, t, *J* = 6.2 Hz), 2.27–1.92 (4H, m), 2.02 (1H, s), 1.86–1.38 (8H, m), 1.59 (3H, s), 1.26 (3H, s), 1.11 (3H, s), 0.96 (9H, t, *J* = 8.0 Hz), 0.89 (9H, s), 0.64 (6H, q, *J* = 8.0 Hz), 0.05 (6H, s); ^{13}C-NMR (100 MHz, $CDCl_3$) δ 137.9, 134.7, 123.9, 114.7, 79.9, 74.1, 68.4, 63.8, 60.8, 37.9, 32.7, 29.5, 25.9, 25.8, 25.0, 23.4, 22.9, 18.4, 16.4, 13.4, 7.0, 5.4, –5.3; IR (ATR) 3466, 2954, 2931, 2877, 2856, 1461, 1252, 1102, 1069, 1005, 911, 835, 814, 775, 726 cm^{-1}; DART-HRMS calcd for $C_{30}H_{61}O_4Si_2$ [(M+H)$^+$] 541.4108, found 541.4099.

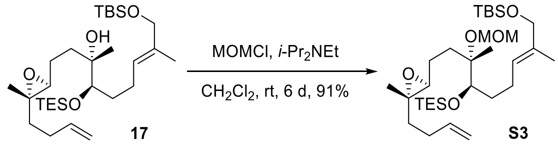

MOM ether S3. To a solution of **17** (72.8 mg, 135 μmol) in CH_2Cl_2 (1.3 mL) were slowly added dropwise *i*-Pr$_2$NEt (329 μL, 1.88 mmol) and MOMCl (60.8 μL, 807 μmol) at 0 °C under a nitrogen atmosphere, and the solution was stirred at room temperature for 6 d. A saturated aqueous solution of $NaHCO_3$ was added to the solution, and the mixture was extracted with CH_2Cl_2 (× 3). The organic layers were dried over anhydrous Na_2SO_4, filtered, and concentrated under reduced pressure. The residue was purified by flash column chromatography (EtOAc/hexane, 5:95) on silica gel to provide **S3** (71.5 mg, 122 μmol, 91%) as a colorless oil: R_f = 0.63 (EtOAc/hexane, 20:80); $[\alpha]^{24}_D$ –3.0 (*c* 0.76, $CHCl_3$); ^{1}H-NMR (400 MHz, $CDCl_3$) δ 5.80 (1H, ddt, *J* = 16.8, 10.0, 6.4 Hz), 5.39 (1H, td, *J* = 7.0, 1.2 Hz), 5.05 (1H, dq, *J* = 17.2, 1.9 Hz), 4.96 (1H, dd, *J* = 10.4, 1.8 Hz), 4.77 (1H, d, *J* = 7.2 Hz), 4.64 (1H, d, *J* = 7.2 Hz), 4.01 (2H, s), 3.57 (1H, dd, *J* = 8.4, 2.6 Hz), 3.36 (3H, s), 2.69 (1H, t, *J* = 6.2 Hz), 2.30–2.08 (3H, m), 2.03–1.95 (1H, m), 1.83–1.67 (4H, m), 1.61 (3H, s), 1.56–1.33 (4H, m), 1.26 (3H, s), 1.17 (3H, s), 0.97 (9H, t, *J* = 8.0 Hz), 0.91 (9H, s), 0.63 (6H, q, *J* = 8.0 Hz), 0.06 (6H, s); ^{13}C-NMR (100 MHz, $CDCl_3$) δ 138.0, 134.4, 124.4, 114.8, 91.1, 80.4, 77.3, 68.5, 63.7, 60.5, 55.5, 38.0, 32.7, 32.6, 29.6, 25.9, 25.4, 22.9, 19.2, 18.4, 16.4, 13.5, 7.1, 5.6, –5.3; IR (ATR) 2954, 2934, 2877, 2858, 1461, 1376, 1253, 1111, 1034, 914, 837, 774 cm^{-1}; ESI-HRMS calcd for $C_{32}H_{64}NaO_5Si_2$ [(M+Na)$^+$] 607.4190, found 607.4205.

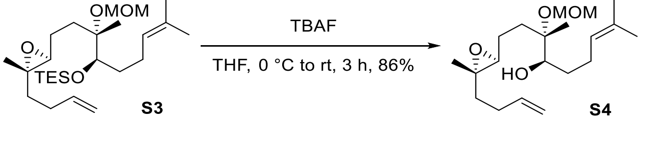

Diol S4. To a solution of **S3** (32.8 mg, 56.3 μmol) in THF (560 μL) was slowly added dropwise TBAF (338 μL, 338 μmol, 1.00 M in THF) at 0 °C under a nitrogen atmosphere, and the solution was stirred at room temperature for 3 h. H_2O was added to the solution, and the mixture was extracted with Et_2O (× 3). The organic layers were dried over anhydrous Na_2SO_4, filtered, and concentrated under reduced pressure. The residue was purified by flash column chromatography (EtOAc/hexane, 50:50) on silica gel to yield **S4** (17.3 mg, 48.6 mmol, 86%) as a colorless oil: R_f = 0.34 (EtOAc/hexane, 50:50); $[\alpha]^{25}_D$ +11.6 (*c* 1.7, $CHCl_3$); ^{1}H-NMR (400 MHz, $CDCl_3$) δ 5.80 (1H, ddt, *J* = 16.8, 10.0, 6.4 Hz), 5.44 (1H, t, *J* = 7.2 Hz), 5.03 (1H, dq, *J* = 17.0, 2.0 Hz), 4.97 (1H, dd, *J* = 10.0, 1.6 Hz), 4.74 (1H, d, *J* = 7.6 Hz), 4.71 (1H, d, *J* = 7.2 Hz), 4.00 (2H, s), 3.49–3.42 (1H, m), 3.41 (3H, s), 3.20 (1H, br d, *J* = 3.6 Hz), 2.72 (1H, t, *J* = 6.4 Hz), 2.37–2.27 (1H, m), 2.25–2.08 (3H, m), 2.01–1.80 (2H, m), 1.75–1.57 (1H, m), 1.55–1.40 (5H, m), 1.69 (3H, s), 1.27 (3H, s), 1.19 (3H, s); ^{13}C-NMR (100 MHz, $CDCl_3$) δ 137.9, 135.3, 125.5, 114.8, 90.9, 80.8, 75.6, 68.7, 63.6, 60.7, 55.6, 37.8, 31.1, 31.0, 29.4, 24.9, 22.7, 19.9, 16.4, 13.6; IR (ATR) 3430, 2974, 2925, 2857, 1455, 1384, 1142, 1070, 1026, 913 cm^{-1}; DART-HRMS calcd for $C_{20}H_{37}O_5$ [(M+H)$^+$] 357.2641, found 357.2633.

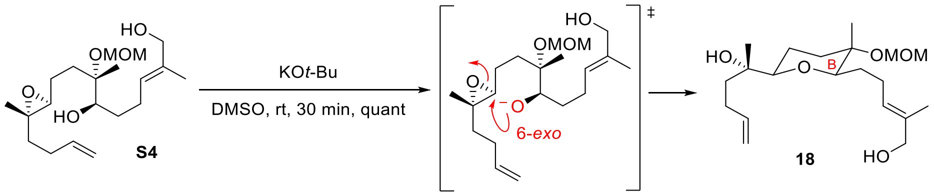

THP 18. To a solution of **S4** (140 mg, 391 μmol) in DMSO (5.8 mL) was slowly added dropwise a solution of KO*t*-Bu (90.5 mg, 782 μmol, 97% purity) in DMSO (2.0 mL) at 20 °C under a nitrogen atmosphere, and the solution was stirred at room temperature for 30 min. After the solution was cooled to 20 °C, H_2O was slowly added dropwise to the solution, and the mixture was extracted with EtOAc (× 3). The organic layers were washed with brine, dried over anhydrous Na_2SO_4, filtered, and concentrated under reduced pressure. The residue was purified by flash column chromatography (EtOAc/hexane, 20:80) on silica gel to give **18** (139 mg, 390 μmol, quant) as a colorless oil: R_f = 0.43 (EtOAc/hexane, 50:50); $[\alpha]^{25}_D$ +16.4 (*c* 1.1, $CHCl_3$); ^{1}H-NMR (600 MHz, $CDCl_3$) δ 5.85 (1H, ddt, *J* = 16.8, 10.2, 6.6 Hz), 5.44 (1H, t, *J* = 7.2 Hz), 5.04 (1H, dq, *J* = 17.4, 1.8 Hz), 4.95 (1H, d, *J* = 10.2 Hz), 4.76 (1H, d, *J* = 7.2 Hz), 4.70 (1H, d, *J* = 7.2 Hz), 3.99 (2H, s), 3.36 (3H, s), 3.20–3.16 (2H, m), 2.49 (1H, br s), 2.28–2.20 (2H, m), 2.16–2.02 (3H, m), 1.83–1.73 (1H, m), 1.68 (3H, s), 1.67–1.57 (3H, m), 1.54–1.45 (1H, m), 1.44–1.34 (2H, m), 1.20 (3H, s), 1.16 (3H, s); ^{13}C-NMR (150 MHz, $CDCl_3$) δ 139.2, 134.9, 126.5, 114.2, 90.6, 84.0, 83.3, 74.9, 73.4, 69.0, 55.3, 36.3, 35.8, 28.5, 27.8, 25.7, 23.5, 23.3, 17.2, 13.7; IR (ATR) 3408, 2972, 2944, 2859, 1443, 1377, 1309, 1070, 1032, 911 cm^{-1}; DART-HRMS calcd for $C_{20}H_{37}O_5$ [(M+H)$^+$] 357.2641, found 357.2637.

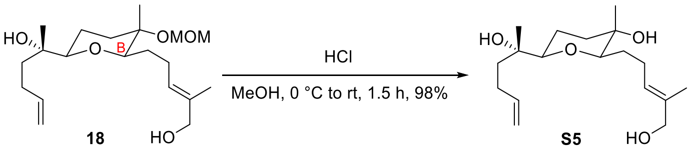

Triol S5. To a solution of **18** (230 mg, 644 μmol) in MeOH (13 mL) was added concentrated HCl (1.23 mL, 14.8 mmol, 12.0 M aqueous solution) at 0 °C under a nitrogen atmosphere, and the solution was stirred at room temperature for 1.5 h. A saturated aqueous solution of $NaHCO_3$ was added to the solution, and the mixture was extracted with EtOAc (× 3). The organic layers were washed with brine, dried over anhydrous Na_2SO_4, filtered, and concentrated under reduced pressure. The residue was purified by flash column chromatography (EtOAc/hexane, 50:50 to 60:40) on silica gel to afford **S5** (197 mg, 631 mmol, 98%) as a colorless oil: R_f = 0.35 (EtOAc/hexane, 70:30); $[\alpha]^{25}_D$ +23.5 (*c* 1.0, $CHCl_3$); ^{1}H-NMR (400 MHz, $CDCl_3$) δ 5.85 (1H, ddt, *J* = 16.8, 10.4, 6.4 Hz), 5.43 (1H, dt, *J* = 7.2, 1.2 Hz), 5.04 (1H, dd, *J* = 16.8, 2.0 Hz), 4.95 (1H, dd, *J* = 10.0, 2.0 Hz), 4.00 (2H, s), 3.22–3.14 (1H, m), 3.10 (1H, dd, *J* = 10.0, 1.8 Hz), 2.46 (1H, br s), 2.24–2.18 (2H, m), 2.17–2.05 (2H, m), 1.89–1.85 (1H, m), 1.83–1.72 (1H, m), 1.68 (3H, s), 1.66–1.52 (4H, m), 1.50–1.37 (2H, m), 1.17 (6H, s); ^{13}C-NMR (100 MHz, $CDCl_3$) δ 139.1, 135.1, 125.9, 114.2, 83.9, 73.4, 69.6, 68.6, 39.7, 35.9, 28.3, 27.7, 24.6, 24.0, 23.2, 19.9, 13.7; IR (ATR) 3373, 2976, 2940, 2858, 1439, 1069, 1007, 943, 912, 751 cm^{-1}; DART-HRMS calcd for $C_{18}H_{33}O_4$ [(M+H)$^+$] 313.2379, found 313.2367.

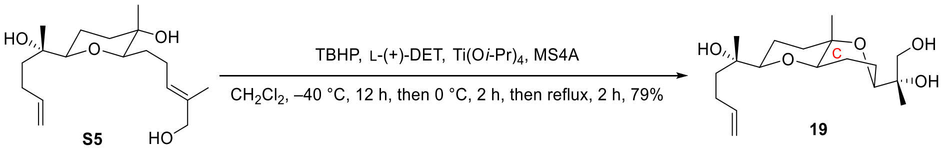

Triol 19.[4] To a suspension of activated MS4A (107 mg, 230 wt%) in CH_2Cl_2 (1.4 mL) were sequentially added Ti(O*i*-Pr)$_4$ (101 μL, 342 μmol) and a solution of L-(+)-DET (98.6 mg, 478 μmol) in CH_2Cl_2 (1.0 mL) at –40 °C under a nitrogen atmosphere, and the solution was stirred for 30 min. To the solution was slowly added dropwise TBHP (80.5 μL, 410 μmol, 5.09 M in CH_2Cl_2), and the solution was stirred for 30 min. A solution of **S5** (92.8 mg, 297 μmol) in CH_2Cl_2 (1.0 mL) was then added dropwise to the solution, and the mixture was stirred for 12 h and at 0 °C for 2 h. The reaction mixture was further refluxed for 2 h. After the mixture was cooled to room temperature, the catalyst was quenched with H_2O, and the mixture was stirred at 0 °C for 30 min. Hydrolysis of the tartrate was effected by adding a 30% aqueous solution of NaOH saturated with NaCl and stirring rigorously at room temperature for 1 h. The resultant mixture was

filtered through a pad of Celite, and the filtrate was extracted with CH_2Cl_2 (× 3). The organic layers were dried over anhydrous Na_2SO_4, filtered, and concentrated under reduced pressure. The residue was purified by flash column chromatography (EtOAc/hexane, 30:70 to 50:50) on silica gel to yield **19** (77.3 mg, 236 μmol, 79%) as a colorless oil: R_f = 0.30 (EtOAc/hexane, 70:30); $[\alpha]^{24}_D$ –9.2 (*c* 2.0, $CHCl_3$); ^{1}H-NMR (600 MHz, $CDCl_3$) δ 5.85 (1H, ddt, *J* = 16.8, 10.2, 6.6 Hz), 5.03 (1H, dq, *J* = 17.4, 1.8 Hz), 4.95 (1H, dd, *J* = 9.6, 1.8 Hz), 3.92 (1H, dd, *J* = 12.0, 3.9 Hz), 3.73 (1H, dd, *J* = 11.4, 2.1 Hz), 3.66 (1H, dd, *J* = 10.8, 7.5 Hz), 3.38 (1H, dd, *J* = 10.2, 8.1 Hz), 3.26 (1H, dd, *J* = 11.4, 2.4 Hz), 2.81 (1H, s), 2.63 (1H, dd, *J* = 8.4, 3.0 Hz), 2.30 (1H, s), 2.27–2.20 (1H, m), 2.12–2.06 (1H, m), 2.03–1.97 (1H, m), 1.89–1.78 (3H, m), 1.67–1.52 (5H, m), 1.44 (1H, ddd, *J* = 13.7, 11.7, 5.1 Hz), 1.22 (3H, s), 1.15 (3H, s), 1.07 (3H, s); ^{13}C-NMR (150 MHz, $CDCl_3$) δ 139.2, 114.2, 84.7, 76.1, 76.0, 73.17, 73.16, 72.2, 67.4, 38.4, 35.6, 27.7, 23.7, 23.2, 21.4, 21.1, 21.0, 20.2; IR (ATR) 3432, 2935, 2864, 1461, 1377, 1276, 1263, 1103, 912, 747 cm^{-1}; DART-HRMS calcd for $C_{18}H_{33}O_5$ [(M+H)$^+$] 329.2328, found 329.2317.

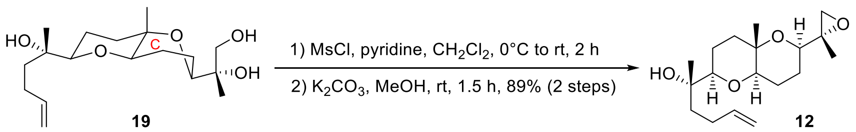

19 12

Epoxide 12. To a solution of **19** (440 mg, 1.34 mmol) in CH_2Cl_2 (4.5 mL) were added dropwise pyridine (270 μL, 3.35 mmol) and MsCl (155 μL, 2.01 mmol) at 0 °C under a nitrogen atmosphere, and the solution was allowed to warm to room temperature and stirred for 2 h. A saturated aqueous solution of $NaHCO_3$ was added to the solution, and the mixture was extracted with CH_2Cl_2 (× 3). The organic layers were dried over anhydrous Na_2SO_4, filtered, and concentrated under reduced pressure to afford a mixture including the desired mesylate, which was used in the next reaction without further purification.

To a solution of the mixture including the desired mesylate in MeOH (6.7 mL) was added K_2CO_3 (463 mg, 3.35 mmol) at room temperature under a nitrogen atmosphere, and the mixture was stirred for 1.5 h. H_2O was added to the solution, and the mixture was extracted with CH_2Cl_2 (× 3). The organic layers were washed with brine, dried over anhydrous Na_2SO_4, filtered, and concentrated under reduced pressure. The residue was purified by column chromatography (EtOAc/hexane, 30:70 to 50:50) on silica gel to provide **12** (371 mg, 1.20 mmol, 89% in 2 steps) as a colorless oil: R_f = 0.63 (EtOAc/hexane, 70:30); $[\alpha]^{25}_D$ –15.5 (*c* 0.83, $CHCl_3$); ^{1}H-NMR (400 MHz, $CDCl_3$) δ 5.85 (1H, ddt, *J* = 16.8, 10.4, 6.8 Hz), 5.04 (1H, dq, *J* = 17.4, 2.0 Hz), 4.95 (1H, dq, *J* = 10.0, 1.6 Hz), 3.63 (1H, dd, *J* = 9.6, 4.4 Hz), 3.51 (1H, dd, *J* = 11.0 , 5.7 Hz), 3.25 (1H, m), 2.72 (1H, d, *J* = 4.8 Hz), 2.62 (1H, d, *J* = 5.2 Hz), 2.34 (1H, s), 2.29–2.19 (1H, m), 2.14–2.04 (1H, m), 1.98–1.77 (4H, m), 1.69–1.54 (5H, m), 1.44 (1H, ddd, *J* = 13.7, 11.7, 5.1 Hz), 1.33 (3H, s), 1.28 (3H, s), 1.15 (3H, s); ^{13}C-NMR (100 MHz, $CDCl_3$) δ 139.2, 114.2, 84.6, 77.5, 73.4, 73.2, 71.8, 57.8, 53.8, 38.5, 35.6, 27.7, 23.7, 23.2, 22.3, 21.3, 20.3, 16.4; IR (ATR) 3489, 3041, 2973, 2943, 2865, 1460, 1443, 1379, 1097, 1070, 1010, 905, 747 cm^{-1}; DART-HRMS calcd for $C_{18}H_{31}O_4$ [(M+H)$^+$] 311.2222, found 311.2220.

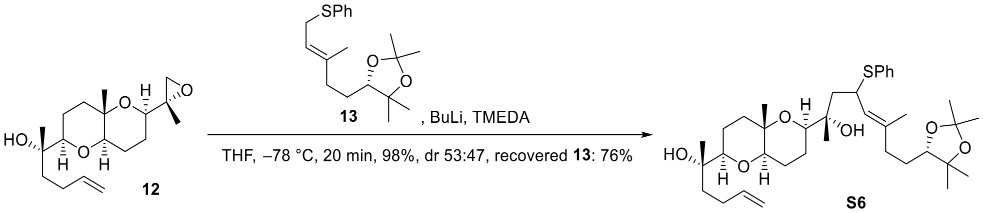

12 S6

Sulfide S6. To a solution of **12** (293 mg, 945 μmol), allylic sulfide **13**[5] (1.82 g, 5.67 mmol), and TMEDA (2.14 mL, 80.4 mmol) in THF (13.5 mL) was added dropwise *n*-BuLi (3.63 mL, 5.67 mmol, 1.56 M in hexane) at –78 °C under a nitrogen atmosphere, and the mixture was stirred for 20 min. After the solution was allowed to warm to room temperature, H_2O was added to the solution, and the mixture was extracted with Et_2O (× 3). The organic layers were dried over anhydrous Na_2SO_4, filtered, and concentrated under reduced pressure. The residue was subjected to flash column chromatography (EtOAc/hexane, 5:95 for **13** to 40:60 for **S6**) on silica gel to afford **S6** (581 mg, 922 μmol, 98%, dr 53:47 according to ^{1}H-NMR analysis) as a mixture of diastereomeric sulfides and a colorless oil and recovered **13** (1.37 g, 4.28 mmol, 76%) as a colorless oil. **S6**: R_f = 0.73 (EtOAc/hexane, 50:50); ^{1}H-NMR (400 MHz, $CDCl_3$) δ 7.45–7.41 (2H, m), 7.31–7.22 (3H, m), 5.85 (1H, ddt, *J* = 16.8, 10.4, 6.8 Hz), 5.15 (1H, t, *J* = 9.2 Hz), 5.04 (1H, dd, *J* = 17.0, 1.4 Hz), 4.95 (1H, d, *J* = 10.0 Hz), 4.31 (0.47H, dt, *J* = 9.6, 3.2 Hz), 4.15–4.09 (0.53H, m), 3.73 (0.53H, dd, *J* = 11.8, 3.8 Hz), 3.66–3.56 (2.47H, m,), 3.24 (1H, d, *J* = 10.4 Hz), 2.53 (1H, d, *J* = 6.8 Hz), 2.35 (1H, d, *J* = 4.8 Hz), 2.30–2.05 (4H, m), 2.01–1.86 (4H, m), 1.84–1.35 (10H, m), 1.61 (3H, s), 1.41 (3H, br s), 1.40 (3H, s), 1.32 (3H, s), 1.22 (3H, br s), 1.14 (3H, s), 1.10 (3H, s), 1.08 (3H, br s); ^{13}C-NMR (100 MHz, $CDCl_3$) δ 139.2, 137.0, 136.0, 134.5, 134.3, 134.2, 128.6, 128.5, 127.6, 127.44, 127.38, 114.2, 106.5, 84.6, 82.9, 82.8, 80.1, 80.0, 76.7, 76.5, 76.4, 76.3, 75.4, 73.8, 73.7, 73.2, 71.7, 71.6, 43.5, 43.0, 42.1, 41.2, 38.6, 38.5, 36.6, 36.5, 35.5, 28.5, 27.7, 27.6, 27.5, 26.89, 26.85, 26.0, 23.7, 23.2, 23.1, 22.9, 21.4, 21.3, 21.1, 20.6, 20.3, 16.4, 16.2; IR (ATR) 3475, 2977, 2941, 2868, 1456, 1440, 1370, 1215, 1199, 1098, 1002, 910, 856, 748, 693 cm^{-1}; ESI-HRMS calcd for $C_{37}H_{58}NaO_6S$ [(M+Na)$^+$] 653.3852, found 653.3846.

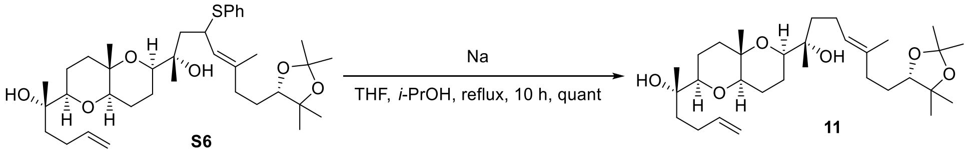

Diol 11. Sulfide **S6** (581 mg, 922 μmol, dr 53:47) was dissolved in THF (12 mL) and *i*-PrOH (6.0 mL) under a nitrogen atmosphere. Several pieces of metallic sodium (741 mg, 32.2 mmol) was added portionwise to the boiling solution under reflux, and the resulting mixture was refluxed for 10 h. After the mixture was cooled to 0 °C, H_2O was added to the solution, and the mixture was extracted with Et_2O (× 3). The organic layers were dried over anhydrous Na_2SO_4, filtered, and concentrated under reduced pressure. The residue was purified by column chromatography (EtOAc/hexane, 10:90) on silica gel to give **11** (482 mg, 922 μmol, quant) as a colorless oil: R_f = 0.73 (EtOAc/hexane, 50:50); $[\alpha]^{26}_D$ –8.4 (*c* 1.6, $CHCl_3$); ^{1}H-NMR (400 MHz, $CDCl_3$) δ 5.85 (1H, ddt, *J* = 16.8, 10.0, 6.4 Hz), 5.18 (1H, t, *J* = 6.6 Hz), 5.04 (1H, dd, *J* = 17.4, 1.8 Hz), 4.95 (1H, dd, *J* = 10.0, 1.2 Hz), 3.72 (1H, dd, *J* = 12.8, 3.4 Hz), 3.68–3.62 (2H, m), 3.26 (1H, dd, *J* = 11.2, 2.8 Hz) 2.39 (1H, s), 2.37 (1H, s), 2.31–1.91 (7H, m), 1.88–1.80 (2H, m), 1.74–1.40 (10H, m), 1.63 (3H, s), 1.43 (3H, s), 1.37–1.26 (1H, m), 1.33 (3H, s), 1.24 (3H, s), 1.20 (3H, s), 1.15 (3H, s), 1.12 (3H, s), 1.10 (3H, s); ^{13}C-NMR (100 MHz, $CDCl_3$) δ 139.2, 134.3, 125.0, 114.2, 106.4, 84.6, 82.7, 80.1, 76.4, 75.7, 73.21, 73.17, 71.6, 38.5, 36.6, 36.1, 35.4, 28.5, 27.7, 27.6, 26.8, 26.0, 23.7, 23.2, 23.1, 22.9, 21.9, 21.4, 21.0, 20.5, 16.0; IR (ATR) 3470, 2976, 2939, 2864, 1458, 1372, 1105, 1065, 1009, 911, 856 cm^{-1}; ESI-HRMS calcd for $C_{31}H_{54}NaO_6$ $[(M+Na)^+]$ 545.3818, found 545.3835.

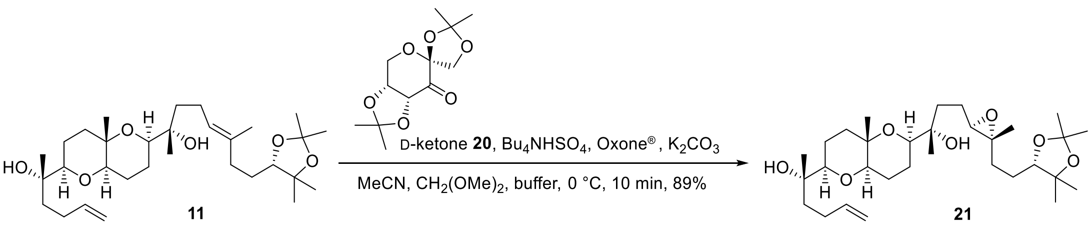

Bishomoepoxy alcohol 21. After **11** (20.2 mg, 38.7 μmol) was dissolved in MeCN (430 μL) and $CH_2(OMe)_2$ (860 μL), Shi's D-ketone **20**[6] (6.00 mg, 23.2 μmol), Bu_4NHSO_4 (1.1 mg, 3.24 μmol), and buffer (860 μL, 5.00 × 10^{-2} M solution of $Na_2B_4O_7 \cdot 10H_2O$ in 4.00 × 10^{-4} M aqueous Na_2(EDTA)) were added to the solution. A solution of Oxone® (65.7 mg, 107 μmol) in aqueous Na_2(EDTA) (860 μL, 4.00 × 10^{-4} M) and a solution of K_2CO_3 (62.6 mg, 453 μmol) in H_2O (860 μL) were added dropwise separately to the solution at 0 °C over a period of 10 min via additional funnels, and the mixture was stirred for another 10 min. The mixture was diluted with H_2O and extracted with CH_2Cl_2 (× 3). The organic layers were washed with brine, dried over anhydrous Na_2SO_4, filtered, and concentrated in vacuo. The residue was purified by flash column chromatography (EtOAc/hexane, 15:85 to 25:75) on silica gel treated with a 1% solution of NEt_3 in hexane to give **21** (18.6 mg, 34.5 μmol, 89%) as a colorless oil: R_f = 0.49 (EtOAc/hexane, 50:50); $[\alpha]^{25}_D$ –3.7 (*c* 0.18, $CHCl_3$); ^{1}H-NMR (400 MHz, $CDCl_3$) δ 5.85 (1H, ddt, *J* = 16.8, 10.4, 6.8 Hz), 5.04 (1H, dd, *J* = 17.2, 1.6 Hz), 4.95 (1H, dd, *J* = 10.0, 1.2 Hz), 3.73 (1H, dd, *J* = 12.8, 2.8 Hz), 3.66–3.62 (2H, m), 3.26 (1H, dd, *J* = 10.4, 2.4 Hz), 2.76 (1H, dd, *J* = 7.2, 5.0 Hz), 2.39 (1H, s), 2.32 (1H, s), 2.32–2.18 (1H, m), 2.15–1.93 (2H, m), 1.91–1.72 (5H, m), 1.70–1.38 (12H, m), 1.41 (3H, s), 1.32 (3H, s), 1.30 (3H, s), 1.24 (3H, s), 1.20 (3H, s), 1.15 (3H, s), 1.10 (3H, s), 1.09 (3H, s); ^{13}C-NMR (100 MHz, $CDCl_3$) δ 139.2, 114.2, 106.6, 84.6, 83.4, 80.2, 77.2, 76.4, 75.7, 73.2, 73.0, 71.7, 64.4, 61.0, 38.5, 36.3, 35.6, 32.8, 28.6, 27.7, 26.9, 26.1, 24.9, 23.7, 23.2, 22.9, 22.8, 21.4, 21.1, 20.6, 16.5; IR (ATR) 3468, 2969, 2931, 2861, 1461, 1374, 1273, 1200, 1106, 1006, 910, 857 cm^{-1}; ESI-HRMS calcd for $C_{31}H_{55}NaO_7$ $[(M+Na)^+]$ 561.3767, found 561.3768.

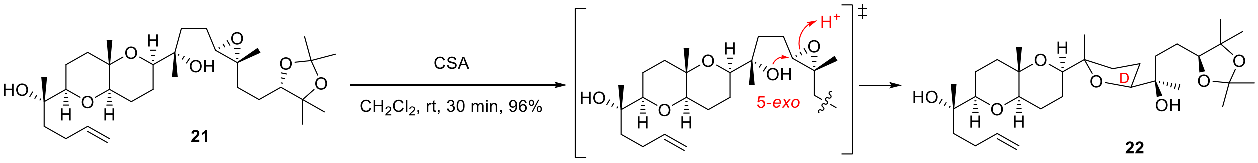

THF 22. To a solution of **21** (37.3 mg, 69.2 μmol) in CH_2Cl_2 (6.9 mL) was added CSA (1.6 mg, 6.89 μmol), and the solution was stirred at room temperature for 30 min under a nitrogen atmosphere. NEt_3 was added to the solution, and the resulting mixture was concentrated under reduced pressure. The residue was purified by column chromatography (EtOAc/hexane, 15:85) on silica gel to give **22** (35.8 mg, 66.5 μmol, 96%) as a colorless oil: R_f = 0.51 (EtOAc/hexane, 50:50); $[\alpha]^{25}_D$ –17.4 (*c* 1.1, $CHCl_3$); ^{1}H-NMR (600 MHz, $CDCl_3$) δ 5.85 (1H, ddt, *J* = 17.2, 10.8, 6.8 Hz), 5.04 (1H, dd, *J* = 16.8, 1.2 Hz), 4.95 (1H, dd, *J* = 10.0, 1.2 Hz), 3.77–3.71 (2H, m), 3.67–3.60 (2H, m), 3.30–3.22 (1H, m), 2.40 (1H, s), 2.32–2.21 (1H, m), 2.19 (1H, s), 2.14–1.88 (3H, m), 1.85–1.77 (5H, m), 1.74–1.40 (11H, m), 1.42 (3H, s), 1.34 (3H, s), 1.26 (3H, s), 1.20 (3H, s), 1.19 (3H, s), 1.15 (3H, s), 1.12 (3H, s), 1.10 (3H, s); ^{13}C-NMR (150 MHz, $CDCl_3$) δ 139.3, 114.2, 106.5, 86.2, 84.7, 84.2, 84.1, 80.3, 76.8, 75.5, 73.2, 72.1, 71.2, 38.7, 35.8, 35.6, 34.6, 28.6, 27.8, 26.9, 26.1, 25.9, 24.1, 23.8, 23.23, 23.21, 23.0, 21.7, 21.4, 21.3, 21.2; IR (ATR) 3467, 2976, 2939, 2867, 1459, 1373, 1262, 1219, 1199, 1103, 1064, 1037, 1018, 910, 856 cm^{-1}; DART-HRMS calcd for $C_{31}H_{55}O_7$ $[(M+H)^+]$ 539.3948, found 539.3955.

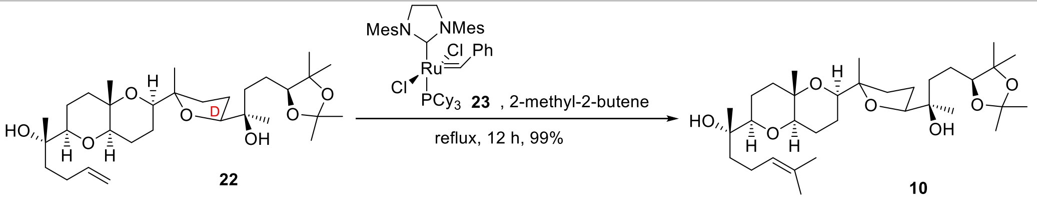

Alkene 10. To a solution of **22** (5.2 mg, 9.66 μmol) in 2-methyl-2-butene (1.2 mL) was added Grubbs' catalyst **23**[7] (1.6 mg, 1.88 μmol) under a nitrogen atmosphere, and the solution was refluxed for 12 h. After the solution was allowed to cool to room temperature, CH_2Cl_2 was added to the solution, and the resulting solution was concentrated under reduced pressure. The residue was purified by column chromatography (EtOAc/hexane, 5:95) on silica gel to provide **10** (5.4 mg, 9.53 μmol, 99%) as a colorless oil: R_f = 0.68 (EtOAc/hexane, 50:50); $[\alpha]^{23}_D$ –15.0 (*c* 1.2, $CHCl_3$); ^{1}H-NMR (400 MHz, $CDCl_3$) δ 5.11 (1H, t, *J* = 6.4 Hz), 3.77–3.71 (2H, m), 3.67–3.60 (2H, m), 3.27–3.24 (1H, m), 2.36 (1H, s), 2.17 (1H, s), 2.16–2.08 (1H, m), 2.07–1.89 (3H, m), 1.88–1.66 (7H, m), 1.69 (3H, s), 1.64–1.45 (9H, m), 1.62 (3H, s), 1.42 (3H, s), 1.33 (3H, s), 1.26 (3H, s), 1.20 (3H, s), 1.18 (3H, s), 1.15 (3H, s), 1.12 (3H, s), 1.10 (3H, s); ^{13}C-NMR (100 MHz, $CDCl_3$) δ 131.5, 124.7, 106.5, 86.2, 84.7, 84.2, 84.1, 80.3, 77.2, 76.8, 75.5, 73.3, 72.1, 71.2, 38.7, 36.5, 35.8, 34.5, 28.6, 26.9, 26.1, 25.9, 25.7, 24.1, 23.8, 23.23, 23.19, 23.0, 22.0, 21.7, 21.4, 21.2, 17.6; IR (ATR) 3464, 2973, 2936, 2866, 1459, 1373, 1262, 1196, 1100, 1015, 947, 912, 855 cm^{-1}; ESI-HRMS calcd for $C_{33}H_{58}NaO_7$ $[(M+Na)^+]$ 589.4080, found 589.4092.

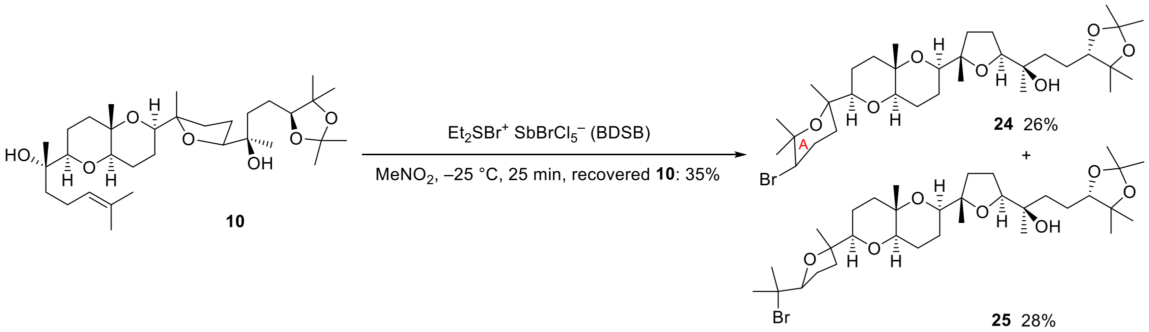

THP 24 and THF 25. To a solution of **10** (31.2 mg, 55.1 μmol) in $MeNO_2$ (5.4 mL) was added a solution of bromodiethylsulfonium bromopentachloroantimonate (BDSB)[8] (46.1 mg, 84.1 μmol) in $MeNO_2$ (250 μL) at –25 °C under a nitrogen atmosphere, and the solution was stirred for 25 min. A 5% aqueous solution of $NaHCO_3$ and a 5% aqueous solution of Na_2SO_3 were added to the solution, and the resulting mixture was stirred for 1 h. H_2O was added to the solution, and the mixture was extracted with CH_2Cl_2 (× 3). The organic layers were dried over anhydrous Na_2SO_4, filtered, and concentrated under reduced pressure. The residue was purified by flash column chromatography (EtOAc/hexane, 5:95 for **24** and **25** to 25:75 for **10**) on silica gel to provide **24** (9.3 mg, 14.4 μmol, 26%), **25** (9.8 mg, 15.2 μmol, 28%), and recovered **10** (11.0 mg, 19.4 μmol, 35%) as each colorless oil. **24**: R_f = 0.53 (EtOAc/hexane, 30:70); $[\alpha]^{26}_D$ –10.4 (*c* 0.30, $CHCl_3$); ^{1}H-NMR (600 MHz, $CDCl_3$) δ 3.90 (1H, dd, *J* = 8.0, 2.8 Hz), 3.76–3.70 (2H, m), 3.66–3.64 (1H, m), 3.55 (1H, dd, *J* = 11.4, 7.2 Hz), 3.04 (1H, dd, *J* = 12.0, 2.1 Hz), 2.24 (1H, dq, *J* = 13.2, 3.6 Hz), 2.16 (1H, s), 2.14–2.09 (1H, m), 2.02–1.95 (1H, m), 1.91–1.86 (1H, m), 1.84–1.66 (9H, m), 1.61–1.46 (7H, m), 1.42 (3H, s), 1.40 (3H, s), 1.33 (3H, s), 1.27 (3H, s), 1.26 (3H, s), 1.20 (3H, s), 1.18 (6H, s), 1.12 (3H, s), 1.09 (3H, s); ^{13}C-NMR (150 MHz, $CDCl_3$) δ 106.5, 86.5, 86.2, 84.3, 84.1, 80.3, 76.8, 75.4, 74.9, 74.4, 72.1, 71.4, 59.1, 38.6, 37.1, 35.8, 34.5, 31.0, 28.6, 26.9, 26.1, 25.9, 24.1, 23.7, 23.2, 23.01, 22.97, 21.7, 21.5, 21.31, 21.26, 20.1; IR (ATR) 3465, 2973, 2932, 2867, 1459, 1372, 1260, 1217, 1198, 1096, 1063, 1015, 910 cm^{-1}; DART-HRMS calcd for $C_{33}H_{58}{}^{79}BrO_7$ $[(M+H)^+]$ 645.3366, found 645.3339; DART-HRMS calcd for $C_{33}H_{58}{}^{81}BrO_7$ $[(M+H)^+]$ 647.3345, found 647.3374. **25**: R_f = 0.49 (EtOAc/hexane, 30:70); $[\alpha]^{25}_D$ –10.6 (*c* 0.17, $CHCl_3$); ^{1}H-NMR (600 MHz, $CDCl_3$) δ 3.88 (1H, t, *J* = 6.9 Hz), 3.77–3.72 (2H, m), 3.66–3.64 (1H, m), 3.59 (1H, dd, *J* = 10.8, 7.5 Hz), 3.31 (1H, dd, *J* = 12.0, 2.4 Hz), 2.17 (1H, s), 2.11–1.75 (8H, m), 1.71 (3H, s), 1.70 (3H, s), 1.70–1.44 (12H, m), 1.42 (3H, s), 1.33 (3H, s), 1.26 (3H, s), 1.20 (3H, s), 1.19 (3H, s), 1.18 (3H, s), 1.12 (3H, s), 1.10 (3H, s); ^{13}C-NMR (150 MHz, $CDCl_3$) δ 106.5, 87.4, 86.2, 85.4, 84.3, 84.1, 83.9, 80.3, 76.6, 75.5, 72.1, 71.3, 68.7, 38.7, 35.8, 34.6, 34.2, 31.2, 29.4, 29.3, 28.6, 26.9, 26.1, 25.9, 24.7, 24.1, 23.2, 23.0, 22.8, 21.7, 21.5, 21.4, 21.2; IR (ATR) 3452, 2976, 2938, 2869, 1457, 1373, 1268, 1219, 1200, 1101, 1070, 1018, 910 cm^{-1}; ESI-HRMS calcd for $C_{33}H_{57}{}^{79}BrNaO_7$ $[(M+Na)^+]$ 667.3185, found 667.3192; ESI-HRMS calcd for $C_{33}H_{57}{}^{81}BrNaO_7$ $[(M+Na)^+]$ 669.3165, found 669.3181.

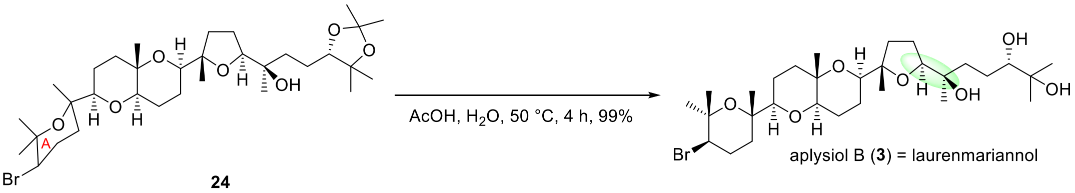

Aplysiol B (3) = laurenmariannol. THP **24** (5.9 mg, 9.16 μmol) was dissolved in AcOH (300 μL) and H_2O (300 μL) under a nitrogen atmosphere, and the mixture was stirred at 50 °C for 4 h. After the solution was allowed to cool to room temperature, a saturated aqueous solution of $NaHCO_3$ was added to the solution, and the mixture was extracted with CH_2Cl_2 (× 3). The organic layers were dried

over anhydrous Na_2SO_4, filtered, and concentrated under reduced pressure. The residue was purified by column chromatography (EtOAc/hexane, 50:50 to 70:30) on silica gel to provide **3** (5.5 mg, 9.10 μmol, 99%) as a colorless oil: R_f = 0.24 (EtOAc/hexane, 70:30); $[\alpha]^{28}_{D}$ –7.8 (*c* 0.12, $CHCl_3$); ^{1}H-NMR (600 MHz, $CDCl_3$) δ 3.89 (1H, dd, *J* = 12.5, 4.0 Hz), 3.74–3.70 (2H, m), 3.55 (1H, dd, *J* = 11.4, 7.3 Hz), 3.44 (1H, dd, *J* = 10.6, 2.2 Hz), 3.04 (1H, dd, *J* = 11.4, 2.2 Hz), 2.74 (1H, br s), 2.31 (1H, br s), 2.24 (1H, dd, *J* = 13.2, 3.9 Hz), 2.13–2.08 (1H, m), 2.03–1.96 (1H, m), 1.92–1.42 (17H, m), 1.40 (3H, s), 1.27 (3H, s), 1.23 (3H, s), 1.20 (3H, s), 1.18 (6H, s), 1.17 (3H, s), 1.09 (3H, s); ^{13}C-NMR (150 MHz, $CDCl_3$) δ 86.5, 86.4, 84.3, 78.4, 76.7, 75.4, 74.9, 74.4, 73.1, 72.3, 71.5, 59.1, 38.6, 37.1, 35.8, 33.6, 31.0, 28.2, 26.7, 25.9, 25.4, 24.1, 23.7, 23.2, 23.0, 21.7, 21.5, 21.3, 21.2, 20.1; IR (ATR) 3463, 2954, 2924, 2871, 2851, 1458, 1374, 1317, 1258, 1217, 1146, 1102, 1030, 914, 835 cm^{-1}; DART-HRMS calcd for $C_{30}H_{54}{}^{79}BrO_7$ [(M+H)$^+$] 605.3053, found 605.3060; DART-HRMS calcd for $C_{30}H_{54}{}^{81}BrO_7$ [(M+H)$^+$] 607.3032, found 607.3046.

Synthesis of Bowden structure 2. The synthesis of structure **2** proposed by Bowden et al.[9] was carried out in the same way as that from the synthetic intermediate **11** to **3** except for Shi asymmetric epoxidation of **11** using L-ketone *ent*-**20**[10] instead of **20** (Scheme S1). Shi asymmetric epoxidation using *ent*-**20** regioselectively proceeded for the trisubstituted double bond to give epoxy alcohol **S7** in modest yield (47%), along with recovery of the starting material **11** (52%). We achieved the construction of the D ring in 99% yield via 5-*exo* cyclization of **S7** using CSA. After cross-metathesis of the terminal olefin in **S8** was performed by the use of Grubbs catalyst™ 2nd generation **23**[7] to provide alkene **S9** in high yield (98%), bromoetherification of the resulting **S9** with BDSB[8] in $MeNO_2$ afforded the desired 6-*endo* product **S10** (29%), 5-*exo* byproduct **S11** (47%), and recovered **S9** (22%). Finally, removal of an acetonide protecting group in **S10** provided Bowden structure **2** in quantitative yield.

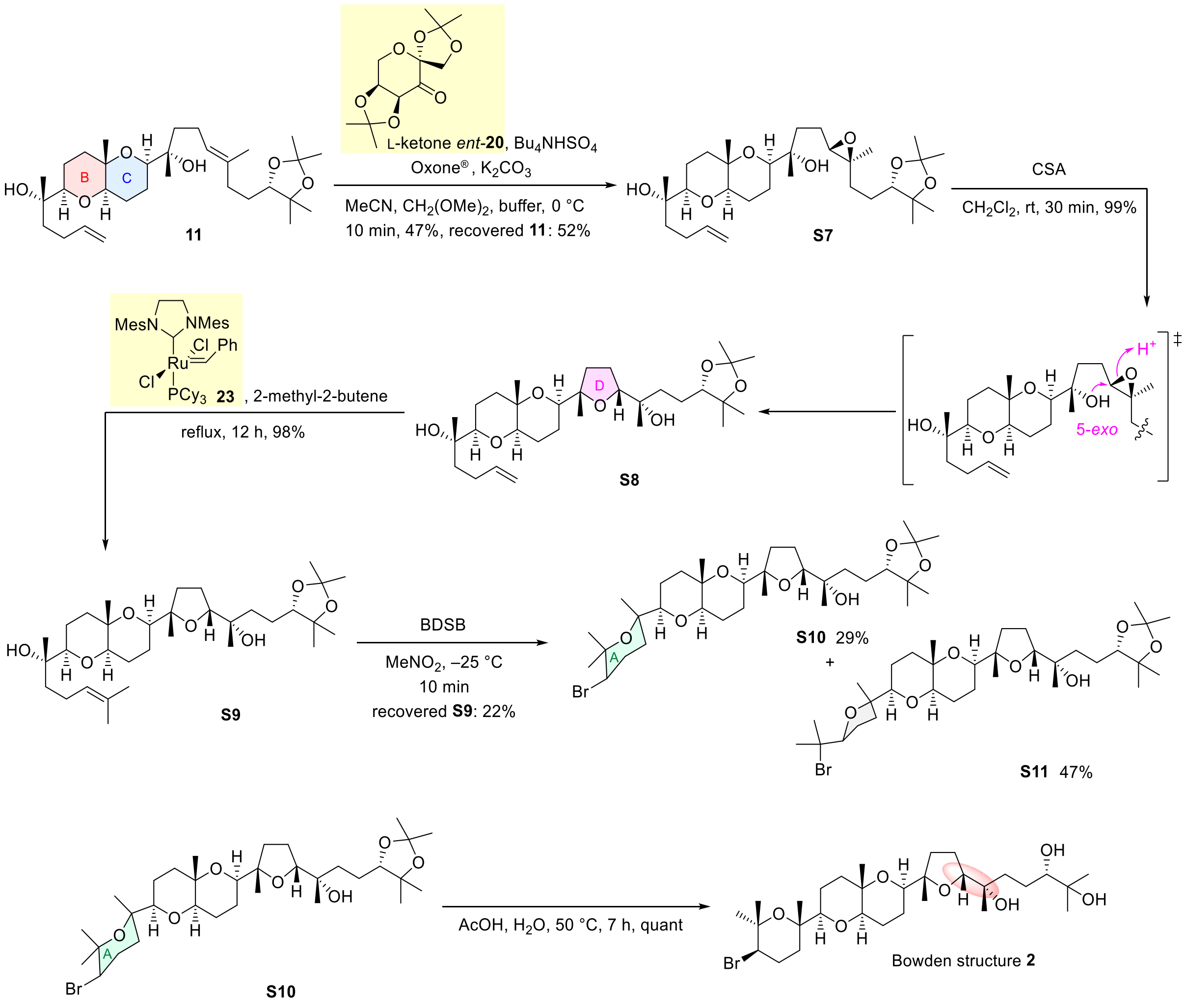

Scheme S1. Synthesis of Bowden structure **2** from diol **11**.

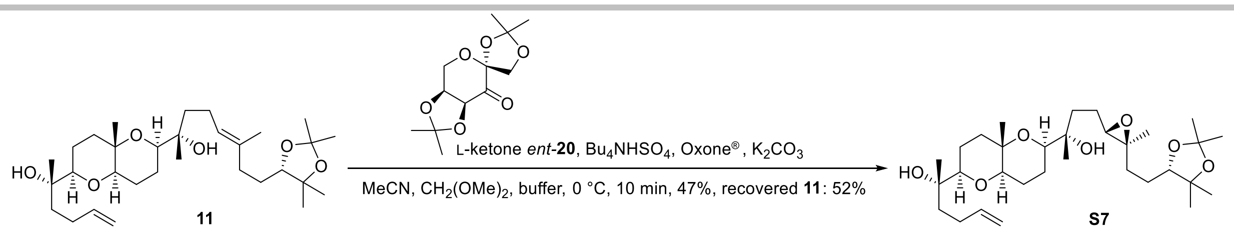

Bishomoepoxy alcohol S7. After **11** (27.7 mg, 53.0 μmol) was dissolved in MeCN (590 μL) and $CH_2(OMe)_2$ (1.2 mL), Shi's L-ketone *ent*-**20**[10] (8.2 mg, 31.8 μmol), Bu_4NHSO_4 (1.4 mg, 4.12 μmol), and buffer (1.2 mL, 5.00 × 10^{-2} M solution of $Na_2B_4O_7$·$10H_2O$ in 4.00 × 10^{-4} M aqueous Na_2(EDTA)) were added to the solution. A solution of Oxone® (89.9 mg, 146 μmol) in aqueous Na_2(EDTA) (1.2 mL, 4.00 × 10^{-4} M) and a solution of K_2CO_3 (85.7 mg, 620 μmol) in H_2O (1.2 mL) were added dropwise separately to the solution at 0 °C over a period of 10 min via additional funnels, and the mixture was stirred for another 10 min. The mixture was diluted with H_2O and extracted with CH_2Cl_2 (× 3). The organic layers were washed with brine, dried over anhydrous Na_2SO_4, filtered, and concentrated in vacuo. The residue was purified by flash column chromatography (EtOAc/hexane, 20:80 to 30:70) on silica gel treated with a 1% solution of NEt_3 in hexane to give **S7** (13.4 mg, 24.9 μmol, 47%) and recovered **11** (14.5 mg, 27.6 μmol, 52%) as each colorless oil. **S7**: R_f = 0.54 (EtOAc/hexane, 50:50); $[\alpha]^{26}_D$ –12.6 (*c* 0.67, $CHCl_3$); ^{1}H-NMR (400 MHz, $CDCl_3$) δ 5.85 (1H, ddt, *J* = 16.8, 10.4, 6.8 Hz), 5.04 (1H, dq, *J* = 16.8, 1.8 Hz), 4.95 (1H, dd, *J* = 10.0, 1.6 Hz), 3.73 (1H, dd, *J* = 13.6, 2.6 Hz), 3.67–3.62 (2H, m), 3.26 (1H, dd, *J* = 10.4, 2.4 Hz), 2.79 (1H, dd, *J* = 6.4, 5.6 Hz), 2.43 (1H, s), 2.32 (1H, s), 2.28–2.19 (1H, m), 2.14–1.92 (2H, m), 1.90–1.44 (17H, m), 1.41 (3H, s), 1.32 (3H, s), 1.29 (3H, s), 1.24 (3H, s), 1.20 (3H, s), 1.15 (3H, s), 1.09 (6H, s); ^{13}C-NMR (100 MHz, $CDCl_3$) δ 139.2, 114.2, 106.6, 84.7, 83.0, 80.1, 77.2, 76.4, 76.0, 73.2, 73.0, 71.7, 63.2, 60.6, 38.5, 35.4, 32.5, 28.5, 27.7, 26.9, 26.1, 24.6, 23.7, 23.2, 22.92, 22.90, 21.4, 21.1, 20.7, 17.0; IR (ATR) 3472, 2975, 2933, 2863, 1460, 1269, 1231, 1216, 1199, 1104, 910, 857 cm^{-1}; ESI-HRMS calcd for $C_{31}H_{54}NaO_7$ $[(M+Na)^+]$ 561.3767, found 561.3769.

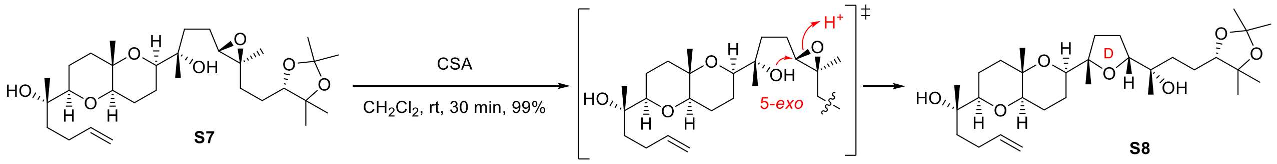

THF S8. To a solution of **S7** (43.4 mg, 80.6 μmol) in CH_2Cl_2 (8.1 mL) was added CSA (1.9 mg, 8.18 μmol) under a nitrogen atmosphere, and the solution was stirred at room temperature for 30 min. NEt_3 was added to the solution, and the resulting mixture was concentrated under reduced pressure. The residue was purified by column chromatography (EtOAc/hexane, 10:90 to 20:80) on silica gel to give **S8** (43.0 mg, 79.9 μmol, 99%) as a colorless oil: R_f = 0.51 (EtOAc/hexane, 50:50); $[\alpha]^{23}_D$ –0.5 (*c* 0.70, $CHCl_3$); ^{1}H-NMR (600 MHz, $CDCl_3$) δ 5.84 (1H, ddt, *J* = 16.8, 10.2, 6.6 Hz), 5.03 (1H, dd, *J* = 16.8, 1.8 Hz), 4.95 (1H, d, *J* = 10.2 Hz), 4.04 (1H, br s), 3.98 (1H, dd, *J* = 12.6, 3.0 Hz), 3.87 (1H, dd, *J* = 7.8, 5.4 Hz), 3.67 (1H, dd, *J* = 11.4, 7.5 Hz), 3.63 (1H, dd, *J* = 7.8, 4.8 Hz), 3.26 (1H, dd, *J* = 11.4, 3.0 Hz), 2.30 (1H, s), 2.26–2.17 (2H, m), 2.13–2.05 (2H, m), 2.01–1.86 (2H, m), 1.78–1.39 (13H, m), 1.41 (3H, s), 1.33–1.28 (1H, m), 1.32 (3H, s), 1.25 (3H, s), 1.23 (3H, s), 1.22 (3H, s), 1.14 (3H, s), 1.11 (3H, s), 1.10 (3H, s); ^{13}C-NMR (150 MHz, $CDCl_3$) δ 139.2, 114.2, 106.4, 85.6, 84.7, 84.5, 83.9, 80.2, 76.3, 75.4, 73.5, 73.2, 71.8, 38.3, 35.6, 35.4, 31.2, 28.5, 27.8, 26.8, 26.3, 26.2, 24.6, 24.5, 23.64, 23.62, 23.2, 22.8, 22.6, 21.09, 21.07; IR (ATR) 3459, 2976, 2938, 2869, 1457, 1373, 1219, 1201, 1101, 1071, 1019, 909 cm^{-1}; ESI-HRMS calcd for $C_{31}H_{54}NaO_7$ $[(M+Na)^+]$ 561.3767, found 561.3770.

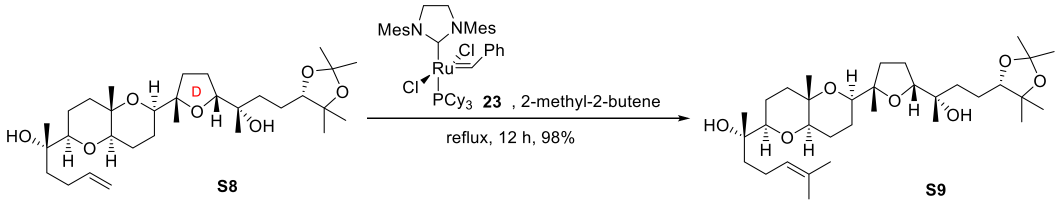

Alkene S9. To a solution of **S8** (8.4 mg, 15.6 μmol) in 2-methyl-2-butene (2.0 mL) was added Grubbs' catalyst **23**[7] (2.0 mg, 2.36 μmol) under a nitrogen atmosphere, and the solution was refluxed for 12 h. After the solution was cooled to room temperature, CH_2Cl_2 was added to the solution, and the resulting solution was concentrated under reduced pressure. The residue was purified by column chromatography (EtOAc/hexane, 5:95 to 20:80) on silica gel to provide **S9** (8.7 mg, 15.4 μmol, 98%) as a colorless oil: R_f = 0.68 (EtOAc/hexane, 50:50); $[\alpha]^{24}_D$ –2.4 (*c* 0.17, $CHCl_3$); ^{1}H-NMR (400 MHz, $CDCl_3$) δ 5.11 (1H, t, *J* = 7.2 Hz), 4.13 (1H, s), 3.98 (1H, dd, *J* = 12.4, 3.0 Hz), 3.88 (1H, dd, *J* = 7.6, 5.4 Hz), 3.69–3.61 (2H, m), 3.26 (1H, dd, *J* = 11.2, 3.0 Hz), 2.32 (1H, s), 2.25–2.07 (3H, m), 2.06–1.29 (17H, m), 1.69 (3H, s), 1.62 (3H, s), 1.41 (3H, s), 1.32 (3H, s), 1.26 (3H, s), 1.23 (3H, s), 1.22 (3H, s), 1.15 (3H, s), 1.11 (3H, s), 1.10 (3H, s); ^{13}C-NMR (100 MHz, $CDCl_3$) δ 131.6, 124.6, 106.4, 85.6, 84.7, 84.5, 83.8, 80.2, 77.2, 76.2, 75.4, 73.5, 73.3, 71.9, 38.3, 36.3, 35.5, 31.0, 28.5, 26.8, 26.3, 26.2, 25.7, 24.7, 24.5, 23.6, 23.2, 22.8, 22.6, 22.0, 21.1, 21.0, 17.7; IR (ATR) 3446, 2960, 2930, 2872, 1462, 1377, 1273, 1122, 1073, 1039, 1020, 999, 951 cm^{-1}; ESI-HRMS calcd for $C_{33}H_{58}NaO_7$ $[(M+Na)^+]$ 589.4080, found 589.4071.

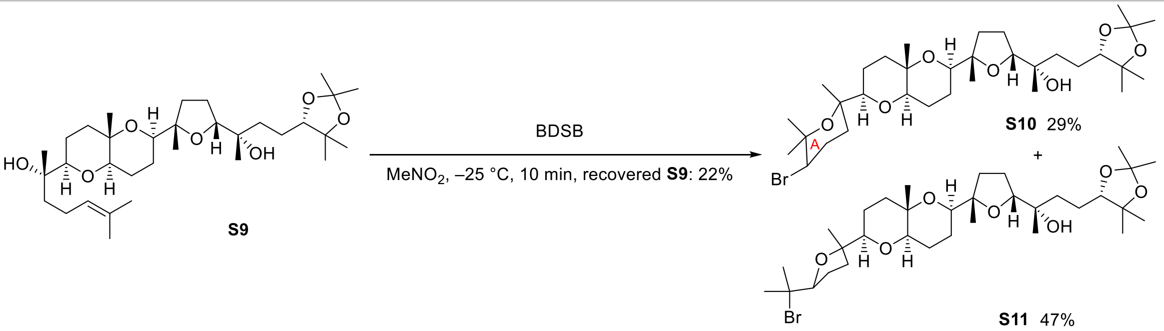

THP S10 and THF S11. To a solution of **S9** (34.8 mg, 62.5 µmol) in $MeNO_2$ (6.0 mL) was added a solution of BDSB[8] (34.3 mg, 62.5 µmol) in $MeNO_2$ (250 µL) at –25 °C under a nitrogen atmosphere, and the solution was stirred for 10 min. A 5% aqueous solution of $NaHCO_3$ and a 5% aqueous solution of Na_2SO_3 were added to the solution, and the resulting mixture was stirred at room temperature for 1 h. H_2O was added to the solution, and the mixture was extracted with CH_2Cl_2 (× 3). The organic layers were dried over anhydrous Na_2SO_4, filtered, and concentrated under reduced pressure. The residue was purified by flash column chromatography (EtOAc/hexane, 2:98 for **S10** and **S11** to 10:90 for **S9**) on silica gel to provide **S10** (11.6 mg, 18.0 µmol, 29%), **S11** (19.1 mg, 29.6 µmol, 47%), and recovered **S9** (7.7 mg, 13.6 µmol, 22%) as each colorless oil. **S10**: R_f = 0.53 (EtOAc/hexane, 30:70); $[\alpha]^{22}_D$ +0.9 (*c* 0.15, $CHCl_3$); ^1H-NMR (600 MHz, $CDCl_3$) δ 4.09 (1H, s), 3.96 (1H, dd, *J* = 12.6, 5.7 Hz), 3.90–3.86 (2H, m), 3.65–3.59 (2H, m), 3.05 (1H, dd, *J* = 11.4, 2.7 Hz), 2.27–2.16 (2H, m), 2.15–2.08 (2H, m), 1.94–1.87 (2H, m), 1.84–1.77 (2H, m), 1.75–1.29 (10H, m), 1.47–1.38 (1H, m), 1.41 (3H, s), 1.39 (3H, s), 1.36–1.28 (1H, m), 1.32 (3H, s), 1.26 (3H, s), 1.25 (3H, s), 1.22 (3H, s), 1.194 (3H, s), 1.186 (3H, s), 1.11 (3H, s), 1.09 (3H, s); ^{13}C-NMR (150 MHz, $CDCl_3$) δ 106.4, 86.6, 85.6, 84.5, 83.9, 80.2, 76.1, 75.2, 74.9, 74.3, 73.6, 72.1, 59.0, 38.2, 37.0, 35.4, 31.1, 31.0, 28.5, 28.2, 26.8, 26.3, 26.2, 24.6, 24.5, 23.7, 22.92, 22.85, 22.7, 21.14, 21.10, 20.1; IR (ATR) 3442, 2979, 2946, 2868, 1458, 1371, 1101, 1062, 1021, 910, 886, 858 cm^{-1}; DART-HRMS calcd for $C_{33}H_{58}{}^{79}BrO_7$ $[(M+H)^+]$ 645.3366, found 645.3349; DART-HRMS calcd for $C_{33}H_{58}{}^{81}BrO_7$ $[(M+H)^+]$ 647.3345, found 647.3330. **S11:** R_f = 0.49 (EtOAc/hexane, 30:70); $[\alpha]^{25}_D$ +0.7 (*c* 0.60, $CHCl_3$); ^1H-NMR (600 MHz, $CDCl_3$) δ 4.12 (1H, br s), 3.98 (1H, dd, *J* = 12.9, 2.7 Hz), 3.89–3.85 (2H, m), 3.66–3.62 (2H, m), 3.32 (1H, dd, *J* = 11.7, 2.1 Hz), 2.22–2.17 (1H, m), 2.14–2.02 (3H, m), 2.01–1.83 (4H, m), 1.76–1.29 (12H, m), 1.71 (3H, s), 1.70 (3H, s), 1.41 (3H, s), 1.32 (3H, s), 1.25 (3H, s), 1.23 (3H, s), 1.22 (3H, s), 1.16 (3H, s), 1.11 (3H, s), 1.10 (3H, s); ^{13}C-NMR (150 MHz, $CDCl_3$) δ 106.4, 87.5, 85.7, 85.3, 84.5, 84.0, 83.9, 80.2, 76.0, 75.3, 73.6, 72.0, 68.7, 38.3, 35.4, 34.1, 31.2, 31.1, 29.4, 29.3, 28.5, 26.8, 26.3, 26.2, 24.65, 24.61, 24.4, 23.6, 22.85, 22.82, 22.7, 21.2, 21.1; IR (ATR) 3420, 2961, 2926, 2855, 1458, 1373, 1259, 1090, 1020, 861 cm^{-1}; DART-HRMS calcd for $C_{33}H_{58}{}^{79}BrO_7$ $[(M+H)^+]$ 645.3366, found 645.3350; DART-HRMS calcd for $C_{33}H_{58}{}^{81}BrO_7$ $[(M+H)^+]$ 647.3345, found 647.3359.

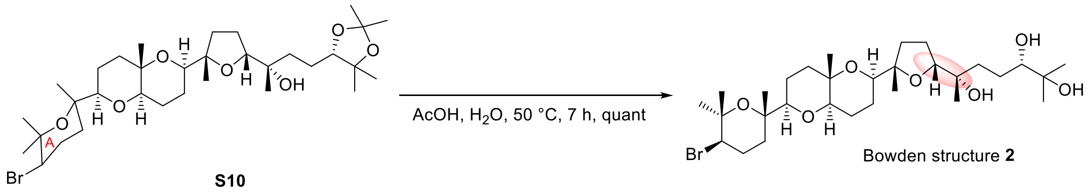

Bowden structure 2. A solution of **S10** (6.1 mg, 9.47 µmol) in AcOH (400 µL) and H_2O (100 µL) was stirred at 50 °C for 7 h. After the solution was allowed to cool to room temperature, a saturated aqueous solution of $NaHCO_3$ was added to the solution, and the mixture was extracted with CH_2Cl_2 (× 3). The organic layers were dried over anhydrous Na_2SO_4, filtered, and concentrated under reduced pressure. The residue was purified by column chromatography (EtOAc/hexane, 50:50) on silica gel to provide **2** (5.7 mg, 9.43 µmol, quant) as a colorless oil: R_f = 0.24 (EtOAc/hexane, 70:30); $[\alpha]^{27}_D$ +15.2 (*c* 0.10, $CHCl_3$); ^1H-NMR (600 MHz, $CDCl_3$) δ 5.08 (1H, br s), 4.98 (1H, s), 3.99 (1H, dd, *J* = 13.0, 3.1 Hz), 3.89 (1H, dd, *J* = 12.3, 3.9 Hz), 3.83 (1H, dd, *J* = 7.9, 4.6 Hz), 3.60 (1H, dd, *J* = 11.2, 7.5 Hz), 3.31 (1H, br d, *J* = 8.1 Hz), 3.05 (1H, dd, *J* = 11.6, 2.0 Hz), 2.62 (1H, s), 2.29–2.05 (4H, m), 1.99–1.87 (2H, m), 1.83–1.40 (14H, m), 1.39 (3H, s), 1.26 (3H, s), 1.23 (3H, s), 1.20 (3H, s), 1.19 (3H, s), 1.18 (3H, s), 1.17 (3H, s), 1.09 (3H, s), ^{13}C-NMR (150 MHz, $CDCl_3$) δ 86.6, 85.9, 85.4, 79.4, 76.0, 75.6, 74.9, 74.3, 74.2, 72.7, 72.4, 59.0, 38.2, 36.9, 35.5, 30.9, 30.7, 28.2, 26.3, 26.2, 25.5, 25.0, 24.0, 23.76, 23.67, 22.9, 22.8, 21.2, 21.0, 20.1; IR (ATR) 3439, 2974, 2936, 2868, 1458, 1235, 1199, 1099, 1021, 911, 856 cm^{-1}; DART-HRMS calcd for $C_{30}H_{54}{}^{79}BrO_7$ $[(M+H)^+]$ 605.3053, found 605.3062; DART-HRMS calcd for $C_{30}H_{54}{}^{81}BrO_7$ $[(M+H)^+]$ 607.3032, found 607.3051.

Table S1. ^{1}H-NMR data of synthetic aplysiol B (**3**), synthetic Bowden structure **2**, natural aplysiol B (**3**), and natural laurenmariannol (**3**).

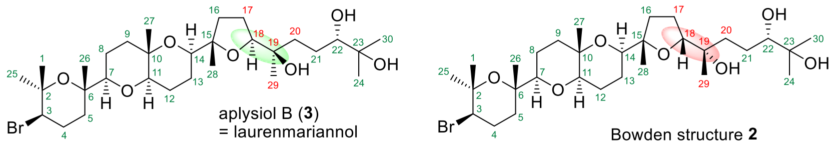

aplysiol B (**3**) = laurenmariannol; Bowden structure **2**

Position	^{1}H δ (ppm) Our synthetic aplysiol B (**3**) = laurenmariannol (600 MHz, $CDCl_3$)[a]	Our synthetic Bowden structure **2** (600 MHz, $CDCl_3$)[a]	Natural aplysiol B (**3**) (600 MHz, $CDCl_3$)[b]	Natural laurenmariannol (**3**) (500 MHz, $CDCl_3$)[c]
1	1.40, 3H, s	1.39, 3H, s	1.40, 3H, s	1.39, 3H, s
3	3.89, dd, *J* = 12.5, 4.0 Hz	3.89, dd, *J* = 12.3, 3.9 Hz	3.89, dd, *J* = 12.2, 4.1 Hz	3.89, dd, *J* = 12.3, 4.0 Hz
4	2.24, 1H, m	2.24, 1H, m	2.22, 1H, m	2.23, 1H, m
	2.10, 1H, m	2.09, 1H, m	2.10, 1H, m	2.10, 1H, m
5	1.81, 1H, m	1.78, 1H, m	1.82, 1H, m	1.80, 1H, m
	1.54, 1H, m	1.53, 1H, m	1.53, 1H, m	1.52, 1H, m
7	3.04, 1H, dd, *J* = 11.4, 2.2 Hz	3.05, 1H, dd, *J* = 11.6, 2.0 Hz	3.04, 1H, br d, *J* = 11.4 Hz	3.03, 1H, dd, *J* = 11.4, 2.2 Hz
8	1.73, 1H, m	1.78, 1H, m	1.72, 2H, m[d]	1.70, 1H, m
	1.42, 1H, m	1.40, 1H, m		1.40, 1H, m
9	1.73, 1H, m	1.78, 1H, m	1.71, 1H, m	1.72, 1H, m
	1.52, 1H, m	1.58, 1H, m	1.41, 1H, m[d]	1.51, 1H, m
11	3.55, 1H, dd, *J* = 11.4, 7.3 Hz	3.60, 1H, dd, *J* = 11.2, 7.5 Hz	3.55, 1H, dd, *J* = 11.1, 7.0 Hz	3.54, dd, *J* = 11.1, 7.2 Hz
12	1.87, 1H, m	1.92, 1H, m	1.87, 1H, m	1.86, 1H, m
	1.50, 1H, m	1.52, 1H, m	1.52, 1H, m	1.50, 1H, m
13	1.78, 2H, m	1.66, 2H, m	1.80, 2H, m	1.77, 1H, m
				1.73, 1H, m[e]
14	3.72, 1H, m	3.99, 1H, dd, *J* = 12.8, 3.2 Hz	3.71, 1H, m	3.70, 1H, dd, *J* = 8.3, 3.5 Hz
16	1.98, 1H, m	2.16, 1H, m	1.98, 1H, m	1.98, 1H, m
	1.67, 1H, m	1.45, 1H, m	1.69, 1H, m	1.65, 1H, m
17	1.81, 2H, m	2.13, 1H, m	1.80, 2H, m	1.79, 1H, m
		1.93, 1H, m		1.66, 1H, m[e]
18	3.72, 1H, m	3.83, 1H, dd, *J* = 7.9, 4.6 Hz	3.71, 1H, m	3.72, 1H, dd, *J* = 7.0, 3.7 Hz
20	1.56, 2H, m	1.58, 1H, m	1.57, 2H, m	1.59, 1H, m
		1.40, 1H, m		1.52, 1H, m
21	1.67, 1H, m	1.68, 1H, m	1.67, 1H, m	1.78, 1H, m[e]
	1.42, 1H, m	1.58, 1H, m	1.40, 1H, m	1.66, 1H, m
22	3.44, 1H, dd, *J* = 10.6, 2.2 Hz	3.31, 1H, br d, *J* = 8.1 Hz	3.44, 1H, dd, *J* = 10.4, 2.2 Hz	3.42, 1H, dd, *J* = 10.6, 1.9 Hz
24	1.17, 3H, s	1.17, 3H, s	1.17, 3H, s	1.16, 3H, s
25	1.27, 3H, s	1.26, 3H, s	1.27, 3H, s	1.26, 3H, s
26	1.20, 3H, s	1.18, 3H, s	1.20, 3H, s	1.19, 3H, s
27	1.18, 3H, s	1.19, 3H, s	1.18, 3H, s	1.17, 3H, s
28	1.09, 3H, s	1.09, 3H, s	1.09, 3H, s	1.08, 3H, s
29	1.18, 3H, s	1.23, 3H, s	1.18, 3H, s	1.16, 3H, s
30	1.23, 3H, s	1.20, 3H, s	1.23, 3H, s	1.22, 3H, s

[a] An internal standard ($CDCl_3$: 7.26 ppm). [b] Cited from ref. [11]. An internal standard is $CDCl_3$: 7.26 ppm for the ^{1}H-NMR spectrum of the natural product reported by Manzo et al. [c] Cited from ref. [12]. [d] It is considered that these chemical shift values are misassigned because the ^{1}H- and ^{13}C-NMR spectra of our synthetic **3** were identical to those of the natural aplysiol B provided by Manzo. [e] It is considered that these chemical shift values are misassigned because the ^{13}C-NMR data of our synthetic **3** were identical to those reported for the natural laurenmariannol.

Table S2. ^{13}C-NMR data of synthetic aplysiol B (**3**), synthetic Bowden structure **2**, natural aplysiol B (**3**), and natural laurenmariannol (**3**).

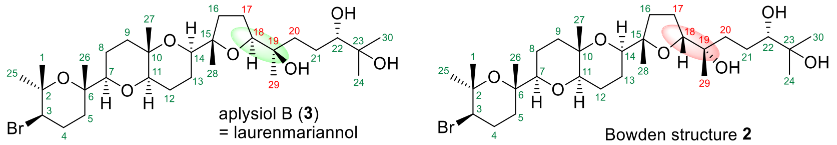

Position	^{13}C δ (ppm) (Δδ = δ$_{natural\ aplysiol\ B}$ – δ$_{our\ synthetic\ sample}$, Δδ = δ$_{natural\ laurenmariannol}$ – δ$_{our\ synthetic\ sample}$)			
	Our synthetic aplysiol B (**3**) = laurenmariannol (150 MHz, $CDCl_3$)[a]	Our synthetic Bowden structure **2** (150 MHz, $CDCl_3$)[a]	Natural aplysiol B (**3**) (150 MHz, $CDCl_3$)[b]	Natural laurenmariannol (**3**) (125 MHz, $CDCl_3$)[c]
1	23.7 (0.0, 0.0)	23.7 (0.0, 0.0)	23.7[d]	23.7
2	74.9 (0.0, 0.0)	74.9 (0.0, 0.0)	74.9	74.9
3	59.1 (0.0, –0.1)	59.0 (+0.1, 0.0)	59.1	59.0
4	28.2 (0.0, +0.1)	28.2 (0.0, +0.1)	28.2	28.3
5	37.1 (0.0, –0.1)	36.9 (+0.2, +0.1)	37.1	37.0
6	74.4 (0.0, 0.0)	74.3 (+0.1, +0.1)	74.4	74.4
7	86.5 (0.0, +0.1)	86.6 (–0.1, 0.0)	86.5	86.6
8	23.0 (0.0, 0.0)	22.9 (+0.1, +0.1)	23.0	23.0
9	38.6 (0.0, +0.1)	38.2 (+0.4, +0.5)	38.6	38.7
10	71.5 (–0.1, 0.0)	72.4 (–1.0, –0.9)	71.4	71.5
11	76.7 (–0.1, 0.0)	76.0 (+0.6, +0.7)	76.6	76.7
12	21.3 (0.0, 0.0)	21.0 (+0.3, +0.3)	21.3	21.3
13	21.5 (–0.1, 0.0)	22.8 (–1.4, –1.3)	21.4	21.5
14	75.4 (0.0, –0.1)	75.6 (–0.2, –0.3)	75.4	75.3
15	84.3 (0.0, 0.0)	85.9 (–1.6, –1.6)	84.3	84.3
16	35.8 (0.0, –0.1)	30.7 (+5.1, +5.0)	35.8	35.7
17	25.9 (0.0, 0.0)	26.3 (–0.4, –0.4)	25.9	25.9
18	86.4 (0.0, 0.0)	85.4 (+1.0, +1.0)	86.4	86.4
19	72.3 (0.0, +0.1)	74.2 (–1.9, –1.8)	72.3	72.4
20	33.6 (+0.1, +0.2)	35.5 (–1.8, –1.7)	33.7	33.8
21	25.4 (0.0, 0.0)	25.5 (–0.1, –0.1)	25.4	25.4
22	78.4 (0.0, +0.1)	79.4 (–1.0, –0.9)	78.4	78.5
23	73.1 (0.0, 0.0)	72.7 (+0.4, +0.4)	73.1	73.1
24	23.2 (0.0, +0.1)	23.7 (–0.5, –0.4)	23.2[d]	23.3
25	31.0 (0.0, 0.0)	30.9 (+0.1, +0.1)	31.0	31.0
26	20.1 (0.0, 0.0)	20.1 (0.0, 0.0)	20.1	20.1
27	21.2 (0.0, 0.0)	21.2 (0.0, 0.0)	21.2	21.2
28	21.7 (–0.1, 0.0)	25.0 (–3.4, –3.3)	21.6[d]	21.7
29	24.1 (0.0, 0.0)	24.0 (+0.1, +0.1)	24.1[d]	24.1
30	26.7 (0.0, –0.1)	26.2 (+0.5, +0.4)	26.7	26.6

[a] An internal standard ($CDCl_3$: 77.0 ppm). [b] Cited from ref. [11]. An internal standard is $CDCl_3$: 77.0 ppm for the ^{13}C-NMR spectrum of the natural product reported by Manzo et al. [c] Cited from ref. [12]. [d] These assignments were wrong and revised because the ^{1}H- and ^{13}C-NMR spectra of our synthetic **3** were identical to those of the natural aplysiol B provided by Manzo.

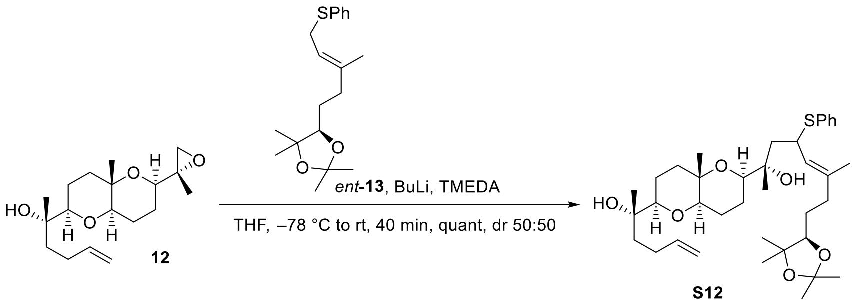

Sulfide S12. To a solution of **12** (140 mg, 451 μmol), sulfide *ent*-**13**[1] (865 mg, 2.70 mmol), and TMEDA (1.02 mL, 6.75 mmol) in THF (6.4 mL) was added dropwise *n*-BuLi (1.73 mL, 2.70 mmol, 1.56 M in hexane) at –78 °C under a nitrogen atmosphere, and the mixture

was stirred for 20 min. After the solution was further stirred at room temperature for 20 min, H_2O was added to the solution, and the mixture was extracted with EtOAc (× 3). The organic layers were dried over anhydrous Na_2SO_4, filtered, and concentrated under reduced pressure. The residue was subjected to flash column chromatography (EtOAc/hexane, 5:95 to 25:75) on silica gel to afford **S12** (284 mg, 451 µmol, quant, dr 50:50 according to ^{1}H-NMR analysis) as a mixture of diastereomeric sulfides and a colorless oil: R_f = 0.66 (EtOAc/hexane, 50:50); ^{1}H-NMR (400 MHz, $CDCl_3$) δ 7.47–7.41 (2H, m), 7.32–7.24 (3H, m), 5.85 (1H, ddt, *J* = 16.4, 10.0, 6.4 Hz), 5.15 (1H, t, *J* = 8.6 Hz), 5.03 (1H, dd, *J* = 16.9, 1.8 Hz), 4.95 (1H, dd, *J* = 8.8, 1.6 Hz), 4.31 (0.50H, dt, *J* = 9.8, 3.1 Hz), 4.15–4.07 (0.50H, m), 3.76–3.70 (0.50H, m), 3.66–3.57 (2.50H, m), 3.29–3.19 (1H, m), 2.54 (1H, d, *J* = 11.6 Hz), 2.40–2.34 (1H, m), 2.30–2.03 (4H, m), 2.01–1.82 (3H, m), 1.81–1.41 (11H, m), 1.41 (3H, s), 1.32 (6H, s), 1.22 (3H, s), 1.14 (3H, s), 1.10 (1.50H, s), 1.07 (3H, s), 1.02 (1.50H, s); ^{13}C-NMR (100 MHz, $CDCl_3$) δ 139.2, 137.0, 135.7, 134.5, 133.9, 128.4, 127.7 127.5, 127.4, 127.2, 114.2, 106.5, 84.6, 82.8, 82.6, 80.1, 80.0, 76.4, 76.2, 75.4, 73.6, 73.1, 71.6, 43.7, 42.8, 41.3, 38.5, 36.6, 35.5, 28.5, 27.7, 27.5, 26.0, 23.6, 23.1, 23.0, 22.9, 21.3, 21.2, 21.1, 20.5, 16.3, 16.2; IR (ATR) 3460, 2974, 2927, 2853, 1459, 1372, 1261, 1196, 1109, 1092, 1022, 1003, 691 cm^{-1}; ESI-HRMS calcd for $C_{37}H_{58}NaO_6S$ [(M+Na)$^+$] 653.3852, found 653.3881.

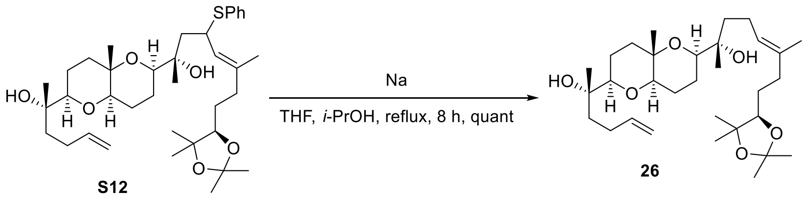

Diol 26. Sulfide **S12** (284 mg, 451 µmol, dr 50:50) was dissolved in THF (5.6 mL) and *i*-PrOH (2.8 mL) under a nitrogen atmosphere. Several pieces of metallic sodium (726 mg, 31.6 mmol) was added portionwise to the boiling solution under reflux, and the resulting mixture was refluxed for 8 h. After the mixture was allowed to cool to 0 °C, H_2O was added to the solution, and the mixture was extracted with Et_2O (× 3). The organic layers were dried over anhydrous Na_2SO_4, filtered, and concentrated under reduced pressure. The residue was purified by column chromatography (EtOAc/hexane, 10:90) on silica gel to give **26** (235 mg, 450 µmol, quant) as a colorless oil: R_f = 0.39 (EtOAc/hexane, 30:70); $[\alpha]^{27}_D$ –14.8 (*c* 0.54, $CHCl_3$); ^{1}H-NMR (400 MHz, $CDCl_3$) δ 5.85 (1H, ddt, *J* = 16.8, 10.0, 6.8 Hz), 5.18 (1H, t, *J* = 6.6 Hz), 5.04 (1H, dd, *J* = 17.4, 1.8 Hz), 4.95 (1H, d, *J* = 10.1 Hz), 3.72 (1H, dd, *J* = 12.8, 2.7 Hz), 3.69–3.61 (2H, m), 3.26 (1H, d, *J* = 10.5 Hz), 2.37 (1H, br s), 2.35 (1H, br s), 2.31–1.92 (9H, m), 1.91–1.42 (11H, m), 1.63 (3H, s), 1.42 (3H, s), 1.33 (3H, s), 1.24 (3H, s), 1.20 (3H, s), 1.15 (3H, s), 1.11 (3H, s), 1.10 (3H, s); ^{13}C-NMR (100 MHz, $CDCl_3$) δ 139.2, 134.3, 125.1, 114.2, 106.4, 84.6, 82.7, 80.1, 76.4, 75.7, 73.21, 73.17, 71.6, 38.5, 36.6, 36.1, 35.6, 28.5, 27.7, 27.6, 26.8, 26.0, 23.7, 23.2, 23.1, 22.9, 21.9, 21.4, 21.1, 20.5, 16.0; IR (ATR) 3472, 2975, 2938, 2864, 1458, 1373, 1274, 1199, 1103, 1065, 1005, 976, 941, 910, 855 cm^{-1}; ESI-HRMS calcd for $C_{31}H_{54}NaO_6$ [(M+Na)$^+$] 545.3818, found 545.3817.

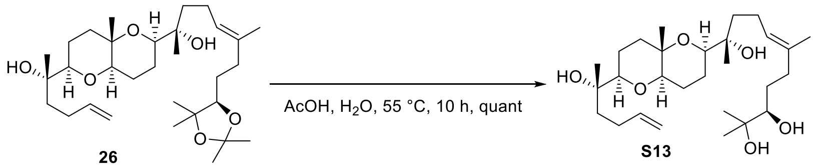

Tetraol S13. A solution of **26** (43.3 mg, 82.9 µmol) in AcOH (400 µL) and H_2O (100 µL) was stirred at 55 °C for 10 h. After the solution was allowed to cool to room temperature, a saturated aqueous solution of $NaHCO_3$ was added to the solution, and the mixture was extracted with CH_2Cl_2 (× 3). The organic layers were dried over anhydrous Na_2SO_4, filtered, and concentrated under reduced pressure. The residue was purified by column chromatography (EtOAc/hexane, 20:80) on silica gel to provide **S13** (40.0 mg, 82.9 µmol, quant) as a colorless oil: R_f = 0.23 (EtOAc/hexane, 50:50); $[\alpha]^{24}_D$ –4.7 (*c* 1.4, $CHCl_3$); ^{1}H-NMR (400 MHz, $CDCl_3$) δ 5.90–5.80 (1H, m), 5.22 (1H, t, *J* = 6.6 Hz), 5.04 (1H, d, *J* = 16.9 Hz), 4.95 (1H, d, *J* = 10.5 Hz), 3.73–3.62 (2H, m), 3.35 (1H, d, *J* = 9.6 Hz), 3.26 (1H, d, *J* = 10.5 Hz), 2.38 (1H, s), 2.35 (1H, s), 2.29–1.92 (10H, m), 1.88–1.68 (4H, m), 1.65–1.40 (6H, m), 1.63 (3H, s), 1.20 (6H, s), 1.15 (6H, s), 1.11 (3H, s); ^{13}C-NMR (100 MHz, $CDCl_3$) δ 139.2, 134.8, 125.4, 114.2, 84.6, 78.1, 76.4, 75.7, 73.3, 73.2, 73.0, 71.6, 38.5, 36.7, 36.0, 35.5, 29.5, 27.7, 26.4, 23.7, 23.2, 23.1, 23.0, 22.0, 21.3, 21.0, 20.5, 15.9; IR (ATR) 3418, 2969, 2943, 2865, 1458, 1375, 1095, 1065, 942, 911 cm^{-1}; ESI-HRMS calcd for $C_{28}H_{50}NaO_6$ [(M+Na)$^+$] 505.3505, found 505.3510.

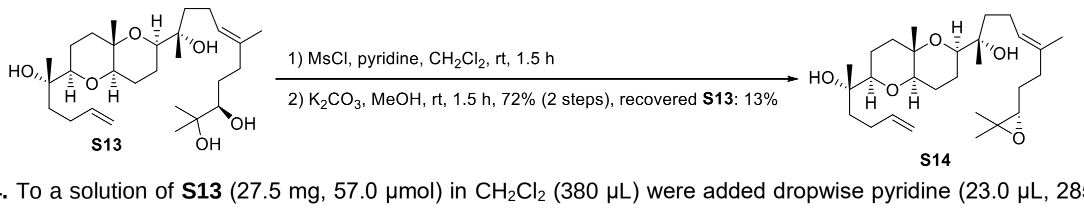

Epoxide S14. To a solution of **S13** (27.5 mg, 57.0 µmol) in CH_2Cl_2 (380 µL) were added dropwise pyridine (23.0 µL, 285 µmol) and MsCl (13.2 µL, 171 µmol) at room temperature under a nitrogen atmosphere, and the solution was stirred for 1.5 h. A saturated aqueous solution of $NaHCO_3$ was added to the solution, and the mixture was extracted with CH_2Cl_2 (× 3). The organic layers were dried over anhydrous Na_2SO_4, filtered, and concentrated under reduced pressure to afford a mixture including the desired mesylate, which was used in the next reaction without further purification.

To a solution of the mixture including the desired mesylate in MeOH (190 µL) was added K_2CO_3 (19.7 mg, 142 µmol) at room temperature under a nitrogen atmosphere, and the mixture was stirred for 1.5 h. H_2O was added to the solution, and the mixture was extracted with CH_2Cl_2 (× 3). The organic layers were washed with brine, dried over anhydrous Na_2SO_4, filtered, and concentrated under reduced pressure. The residue was purified by column chromatography (EtOAc/hexane, 20:80) on silica gel to provide **S14** (19.0 mg, 40.9 µmol, 72% in 2 steps) and recovered **S13** (3.6 mg, 7.46 µmol, 13%) as each colorless oil. **S14**: R_f = 0.44 (EtOAc/hexane, 50:50); $[\alpha]^{27}_D$ –16.2 (*c* 0.25, $CHCl_3$); ^{1}H-NMR (400 MHz, $CDCl_3$) δ 5.85 (1H, ddt, *J* = 16.8, 10.4, 6.8 Hz), 5.17 (1H, t, *J* = 6.9 Hz), 5.03 (1H, dq, *J* = 17.4, 1.4 Hz), 4.95 (1H, dd, *J* = 10.4, 1.1 Hz), 3.72 (1H, dd, *J* = 12.8, 2.7 Hz), 3.64 (1H, dd, *J* = 11.2 , 7.5 Hz), 3.26 (1H, dd, *J* = 10.4, 2.4 Hz), 2.70 (1H, t, *J* = 6.2 Hz), 2.36 (1H, br s), 2.34 (1H, br s), 2.27–1.91 (7H, m), 1.90–1.77 (2H, m), 1.75–1.50 (10H, m), 1.63 (3H, s), 1.48–1.40 (1H, m), 1.30 (3H, s), 1.26 (3H, s), 1.20 (3H, s), 1.15 (3H, s) 1.11 (3H, s); ^{13}C-NMR (100 MHz, $CDCl_3$) δ 139.2, 134.1, 125.3, 114.2, 84.6, 76.4, 75.7, 73.21, 73.17, 71.6, 64.2, 58.3, 38.5, 36.3, 36.2, 35.6, 27.7, 27.4, 24.9, 23.7, 23.2, 23.1, 21.9, 21.4, 21.1, 20.5, 18.7, 16.0; IR (ATR) 3460, 2962, 2924, 2865, 1457, 1377, 1102, 908 cm^{-1}; ESI-HRMS calcd for $C_{28}H_{48}NaO_5$ [(M+Na)$^+$] 487.3399, found 487.3394.

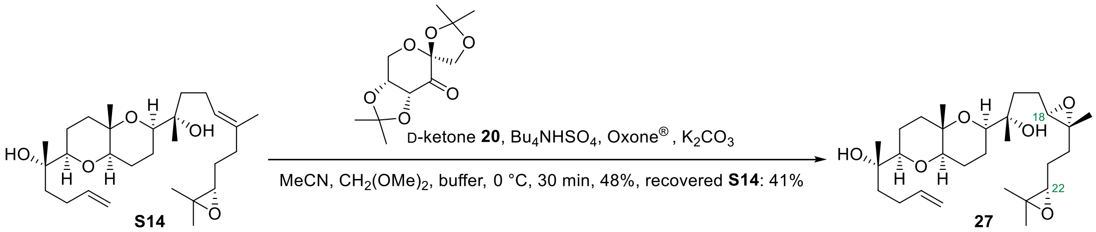

Diepoxide 27. After **S14** (16.0 mg, 34.5 µmol) was dissolved in MeCN (380 µL) and $CH_2(OMe)_2$ (770 µL), Shi's D-ketone **20**[6] (5.3 mg, 20.5 µmol), Bu_4NHSO_4 (0.9 mg, 2.65 µmol), and buffer (770 µL, 5.00 × 10^{-2} M solution of $Na_2B_4O_7{\cdot}10H_2O$ in 4.00 × 10^{-4} M aqueous Na_2(EDTA)) were added to the solution. A solution of Oxone® (58.5 mg, 95.2 µmol) in aqueous Na_2(EDTA) (770 µL, 4.00 × 10^{-4} M) and a solution of K_2CO_3 (60.5 mg, 438 µmol) in H_2O (770 µL) were added dropwise separately to the solution at 0 °C over a period of 10 min via additional funnels, and the mixture was stirred for another 30 min. The mixture was diluted with H_2O and extracted with CH_2Cl_2 (× 3). The organic layers were washed with brine, dried over anhydrous Na_2SO_4, filtered, and concentrated in vacuo. The residue was purified by flash column chromatography (EtOAc/hexane, 10:90 for **S14** to 30:70 for **27**) on silica gel treated with a 1% solution of triethylamine in hexane to give **27** (7.9 mg, 16.4 µmol, 48%) and recovered **S14** (6.5 mg, 14.0 µmol, 41%) as each colorless oil. **27**: R_f = 0.30 (EtOAc/hexane, 50:50); $[\alpha]^{25}_D$ –13.5 (*c* 0.40, $CHCl_3$); ^{1}H-NMR (400 MHz, $CDCl_3$) δ 5.85 (1H, ddt, *J* = 16.8, 10.0, 6.4 Hz), 5.03 (1H, dd, *J* = 16.9, 1.8 Hz), 4.95 (1H, dd, *J* = 11.0, 2.0 Hz), 3.73 (1H, dd, *J* = 12.8, 2.7 Hz), 3.64 (1H, dd, *J* = 11.2, 7.5 Hz), 3.26 (1H, d, *J* = 10.5 Hz), 2.76 (1H, dd, *J* = 6.9, 5.0 Hz), 2.70 (1H, t, *J* = 5.0 Hz), 2.41 (1H, s), 2.33 (1H, s), 2.29–2.18 (1H, m), 2.13–1.40 (19H, m), 1.31 (3H, s), 1.29 (3H, s), 1.28 (3H, s), 1.20 (3H, s), 1.15 (3H, s), 1.09 (3H, s); ^{13}C-NMR (100 MHz, $CDCl_3$) δ 139.2, 114.2, 84.7, 77.2, 76.4, 75.7, 73.2, 73.0, 71.7, 64.3, 64.0, 60.8, 58.4, 38.5, 35.7, 35.6, 32.7, 27.7, 24.83, 24.77, 23.7, 23.2, 22.9, 21.4, 21.1, 20.6, 18.7, 16.5; IR (ATR) 3448, 2968, 2933, 2864, 1459, 1378, 1101, 1066, 908 cm^{-1}; ESI-HRMS calcd for $C_{28}H_{48}NaO_6$ [(M+Na)$^+$] 503.3349, found 503.3345.

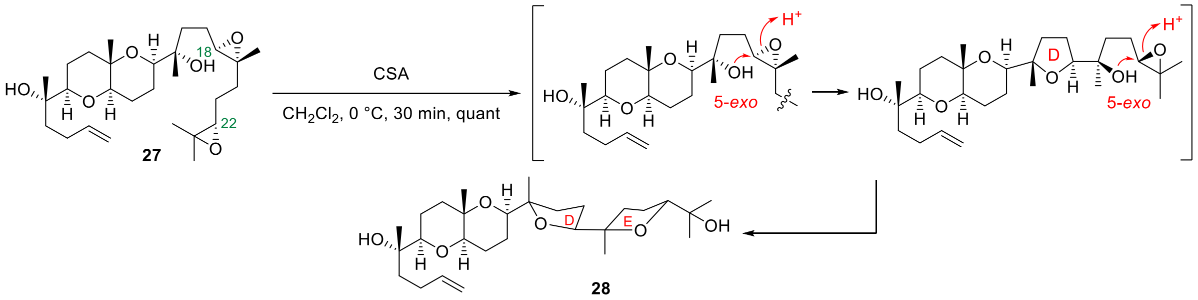

Bis-THF 28.[13] To a solution of **27** (7.9 mg, 16.4 µmol) in CH_2Cl_2 (1.6 mL) was added CSA (0.4 mg, 1.72 µmol) at 0 °C under a nitrogen atmosphere, and the solution was stirred for 30 min. NEt_3 was added to the solution, and the resulting mixture was concentrated under reduced pressure. The residue was purified by column chromatography (EtOAc/hexane, 10:90 to 30:70) on silica gel to give **28** (7.9 mg, 16.4 µmol, quant) as a colorless oil: R_f = 0.51 (EtOAc/hexane, 50:50); $[\alpha]^{25}_D$ –9.8 (*c* 0.23, $CHCl_3$); ^{1}H-NMR (600 MHz, $CDCl_3$) δ 5.85 (1H, ddt, *J* = 17.0, 10.3, 6.6 Hz), 5.03 (1H, dd, *J* = 17.1, 1.6 Hz), 4.95 (1H, d, *J* = 10.3 Hz), 3.86 (1H, dd, *J* = 8.4, 6.1 Hz), 3.77 (1H, dd, *J* = 7.4, 7.0 Hz), 3.73 (1H, dd, *J* = 11.3, 4.4 Hz), 3.61 (1H, dd, *J* = 11.0, 7.5 Hz), 3.25 (1H, dd, *J* = 11.0, 2.9 Hz), 2.37 (1H, s), 2.27–2.21 (1H, m), 2.18 (1H, s), 2.13–2.04 (1H, m), 2.00–1.91 (3H, m), 1.89–1.75 (6H, m), 1.70–1.52 (8H, m), 1.44 (1H, ddd, *J* = 13.6, 11.8, 5.0 Hz), 1.20 (6H, s), 1.14 (6H, s), 1.11 (3H, s), 1.10 (3H, s); ^{13}C-NMR (150 MHz, $CDCl_3$) δ 139.3, 114.2, 86.8, 85.9, 84.6, 84.5, 85.0, 77.0, 75.5, 73.2, 71.2, 70.6, 38.7, 35.6, 35.5, 33.9, 27.8, 27.7, 27.6, 26.6, 24.0, 23.8, 23.2, 21.6, 21.4, 21.3, 21.2; IR (ATR) 3448, 2971, 2934, 2867, 1459, 1374, 1099, 1063, 907 cm^{-1}; DART-HRMS calcd for $C_{28}H_{49}O_6$ [(M+H)$^+$] 481.3529, found 481.3555.

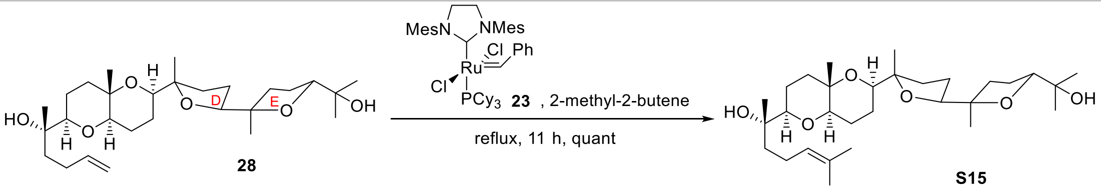

Alkene S15. To a solution of **28** (9.9 mg, 20.6 μmol) in 2-methyl-2-butene (2.6 mL) was added Grubbs' catalyst **23**[7] (2.6 mg, 3.06 μmol) under a nitrogen atmosphere, and the solution was refluxed for 11 h. After the solution was cooled to room temperature, CH_2Cl_2 was added to the solution, and the resulting solution was concentrated under reduced pressure. The residue was purified by column chromatography (EtOAc/hexane, 5:95 to 20:80) on silica gel to provide **S15** (10.5 mg, 20.6 μmol, quant) as a colorless oil: R_f = 0.51 (EtOAc/hexane, 50:50); $[\alpha]^{24}_{D}$ –10.1 (*c* 0.53, $CHCl_3$); ^{1}H-NMR (400 MHz, $CDCl_3$) δ 5.11 (1H, t, *J* = 6.4 Hz), 3.86 (1H, t, *J* = 7.1 Hz), 3.77 (1H, t, *J* = 7.5 Hz), 3.72 (1H, dd, *J* = 10.0, 5.0 Hz), 3.61 (1H, dd, *J* = 11.0, 7.3 Hz), 3.24 (1H, d, *J* = 7.3 Hz), 2.35 (s, 1H), 2.18 (s, 1H), 2.17–2.07 (1H, m), 2.06–1.50 (18H, m), 1.62 (3H, s), 1.60 (3H, s), 1.40–1.33 (1H, m), 1.20 (6H, s), 1.15 (3H, s), 1.14 (3H, s), 1.11 (3H, s), 1.10 (3H, s); ^{13}C-NMR (100 MHz, $CDCl_3$) δ 131.5, 124.8, 86.8, 85.9, 84.6, 84.5, 77.2, 75.5, 73.3, 71.2, 70.6, 38.7, 36.5, 35.5, 34.0, 27.6, 26.6, 25.7, 24.0, 23.8, 23.6, 23.2, 22.0, 21.5, 21.4, 21.3, 21.2, 17.7; IR (ATR) 3457, 2970, 2931, 2864, 1459, 1375, 1321, 1222, 1181, 1098, 1066, 1012, 948, 900, 834, 798, 772, 666, 642, 634 cm^{-1}; ESI-HRMS calcd for $C_{30}H_{52}NaO_6$ [(M+Na)$^+$] 531.3662, found 531.3661.

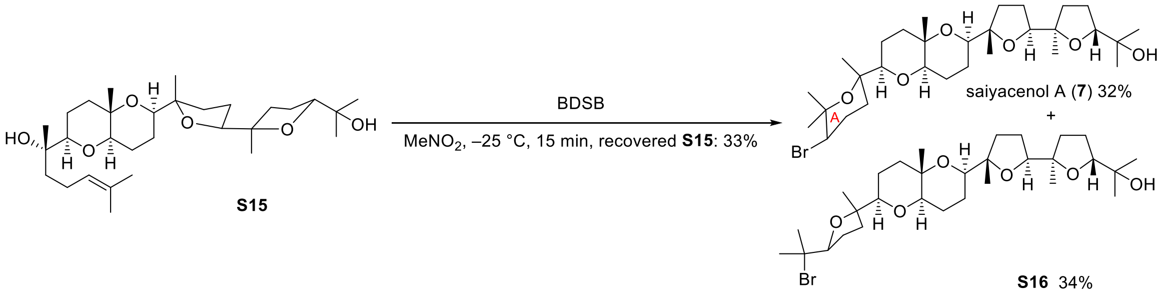

Saiyacenol A (7) and THF S16. To a solution of **S15** (10.5 mg, 20.6 μmol) in $MeNO_2$ (1.8 mL) was added a solution of BDSB[8] (11.3 mg, 20.6 μmol) in $MeNO_2$ (250 μL) at –25 °C under a nitrogen atmosphere, and the solution was stirred for 15 min. A 5% aqueous solution of $NaHCO_3$ and a 5% aqueous solution of Na_2SO_3 were added to the solution, and the resulting mixture was stirred at room temperature for 1 h. H_2O was added to the solution, and the mixture was extracted with EtOAc (× 3). The organic layers were dried over anhydrous Na_2SO_4, filtered, and concentrated under reduced pressure. The residue was purified by flash column chromatography (EtOAc/hexane, 5:95 for **7** and **S16** to 20:80 for **S15**) on silica gel to provide **7** (3.9 mg, 6.65 μmol, 32%) as white amorphous, **S16** (4.1 mg, 6.99 μmol, 34%) as a colorless oil, and recovered **S15** (4.0 mg, 6.82 μmol, 33%) as a colorless oil. **7**: R_f = 0.47 (EtOAc/hexane, 30:70); $[\alpha]^{25}_{D}$ +1.6 (*c* 0.12, $CHCl_3$); ^{1}H-NMR (600 MHz, $CDCl_3$) δ 3.90 (1H, dd, *J* = 12.2, 4.1 Hz), 3.86 (1H, dd, *J* = 8.4, 6.2 Hz), 3.77 (1H, dd, *J* = 8.6, 6.9 Hz), 3.72 (1H, dd, *J* = 10.7, 4.5 Hz), 3.54 (1H, dd, *J* = 11.2, 7.4 Hz), 3.04 (1H, dd, *J* = 11.5, 2.4 Hz), 2.24 (1H, dq, *J* = 12.8, 3.8 Hz), 2.14–2.07 (1H, m), 2.10 (1H, s), 2.00–1.93 (2H, m), 1.90–1.84 (2H, m), 1.83–1.70 (7H, m), 1.69–1.41 (7H, m), 1.40 (3H, s), 1.27 (3H, s), 1.199 (3H, s), 1.195 (3H, s), 1.17 (3H, s), 1.14 (3H, s), 1.11 (3H, s), 1.09 (3H, s); ^{13}C-NMR (150 MHz, $CDCl_3$) δ 86.8, 86.5, 85.9, 84.52, 84.49, 76.8, 75.4, 74.9, 74.4, 71.4, 70.6, 59.1, 38.7, 37.1, 35.6, 34.0, 31.0, 28.2, 27.7, 27.6, 26.6, 24.0, 23.7, 23.6, 23.0, 21.50, 21.45, 21.34, 21.26, 20.1; IR (ATR) 3440, 2959, 2925, 2855, 1463, 1377, 1278, 1100, 1065, 1025, 956 cm^{-1}; ESI-HRMS calcd for $C_{30}H_{51}{}^{79}BrNaO_6$ [(M+Na)$^+$] 609.2767, found 609.2767; ESI-HRMS calcd for $C_{30}H_{51}{}^{81}BrNaO_6$ [(M+Na)$^+$] 611.2746, found 611.2757. **S16:** R_f = 0.39 (EtOAc/hexane, 30:70); $[\alpha]^{24}_{D}$ –14.6 (*c* 0.11, $CHCl_3$); ^{1}H-NMR (600 MHz, $CDCl_3$) δ 3.87 (1H, t, *J* = 6.2 Hz), 3.86 (1H, t, *J* = 6.1 Hz), 3.77 (1H, dd, *J* = 8.5, 7.0 Hz), 3.72 (1H, dd, *J* = 10.9, 4.6 Hz), 3.57 (1H, dd, *J* = 11.1, 7.4 Hz), 3.30 (1H, dd, *J* = 11.6, 2.4 Hz), 2.13–2.03 (2H, m), 2.00–1.85 (5H, m), 1.83–1.74 (5H, m), 1.71 (3H, s), 1.70 (3H, s), 1.69–1.50 (7H, m), 1.47–1.39 (1H, m), 1.202 (3H, s), 1.197 (3H, s), 1.17 (3H, s), 1.14 (3H, s), 1.11 (3H, s), 1.10 (3H, s); ^{13}C-NMR (150 MHz, $CDCl_3$) δ 87.4, 86.8, 85.9, 85.4, 84.6, 84.5, 83.9, 76.6, 75.4, 71.3, 70.6, 68.8, 38.7, 35.5, 34.2, 33.9, 31.2, 29.5, 29.3, 27.7, 27.6, 26.6, 24.8, 24.0, 23.6, 22.8, 21.6, 21.5, 21.4, 21.2; IR (ATR) 3448, 2971, 2927, 2869, 1461, 1374, 1320, 1096, 1065, 1035, 953 cm^{-1}; ESI-HRMS calcd for $C_{30}H_{51}{}^{79}BrNaO_6$ [(M+Na)$^+$] 609.2767, found 609.2767; ESI-HRMS calcd for $C_{30}H_{51}{}^{81}BrNaO_6$ [(M+Na)$^+$] 611.2746, found 611.2761.

Table S3. ^{1}H-NMR data of synthetic and natural saiyacenol A (**7**).

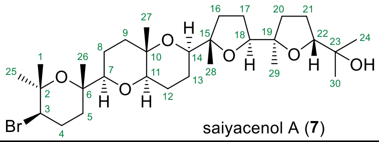

saiyacenol A (**7**)

Position	^{1}H δ (ppm)	
	Our synthetic sample (600 MHz, $CDCl_3$)[a]	Natural sample (600 MHz, $CDCl_3$)[b]
1	1.27, 3H, s	1.27, 3H, s
3	3.90, 1H, dd, *J* = 12.2, 4.1 Hz	3.89, 1H, dd, *J* = 12.3, 4.1 Hz
4	2.24, 1H, m	2.24, 1H, m
	2.11, 1H, m	2.10, 1H, m
5	1.82, 1H, m	1.81, 1H, m
	1.53, 1H, m	1.53, 1H, m
7	3.04, 1H, dd, *J* = 11.5, 2.4 Hz	3.04, 1H, dd, *J* = 11.4, 2.5 Hz
8	1.73, 1H, m	1.73, 1H, m
	1.42, 1H, m	1.41, 1H, m
9	1.74, 1H, m	1.75, 1H, m
	1.55, 1H, m	1.55, 1H, m
11	3.54, 1H, dd, *J* = 11.2, 7.4 Hz	3.54, 1H, dd, *J* = 11.1, 7.3 Hz
12	1.87, 1H, m	1.87, 1H, m
	1.49, 1H, m	1.49, 1H, m
13	1.78, 2H, m	1.78, 2H, m
14	3.72, 1H, dd, *J* = 10.7, 4.5 Hz	3.71, 1H, dd, *J* = 11.0, 4.3 Hz
16	1.96, 1H, m	1.96, 1H, m
	1.64, 1H, m	1.64, 1H, m
17	1.86, 1H, m	1.86, 1H, m
	1.66, 1H, m	1.66, 1H, m
18	3.86, 1H, dd, *J* = 8.4, 6.2 Hz	3.86, 1H, dd, *J* = 8.4, 6.0 Hz
20	1.97, 1H, m	1.97, 1H, m
	1.61, 1H, m	1.60, 1H, m
21	1.81, 2H, m	1.81, 2H, m
22	3.77, 1H, dd, *J* = 8.6, 6.9 Hz	3.77, 1H, dd, *J* = 8.6, 6.8 Hz
24	1.11, 3H, s	1.11, 3H, s
25	1.40, 3H, s	1.39, 3H, s
26	1.20, 3H, s	1.20, 3H, s
27	1.17, 3H, s	1.17, 3H, s
28	1.09, 3H, s	1.09, 3H, s
29	1.14, 3H, s	1.14, 3H, s
30	1.20, 3H, s	1.21, 3H, s

[a] An internal standard ($CDCl_3$: 7.26 ppm). [b] Cited from ref. [14]. An internal standard is $CDCl_3$: 7.26 ppm for the ^{1}H-NMR spectrum of the natural product reported by Fernández and Daranas et al.

Table S4. ^{13}C-NMR data of synthetic and natural saiyacenol A (**7**).

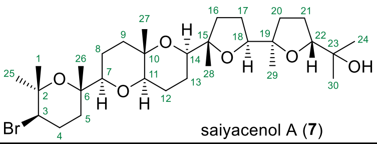

saiyacenol A (**7**)

Position	^{13}C δ (ppm) (Δδ = $δ_{natural\ sample}$ – $δ_{our\ synthetic\ sample}$)		Position	^{13}C δ (ppm) (Δδ = $δ_{natural\ sample}$ – $δ_{our\ synthetic\ sample}$)	
	Our synthetic sample (150 MHz, $CDCl_3$)[a]	Natural sample (150 MHz, $CDCl_3$)[b]		Our synthetic sample (150 MHz, $CDCl_3$)[a]	Natural sample (150 MHz, $CDCl_3$)[b]
1	31.0 (–0.1)	30.9	16	35.6 (–0.3)	35.3
2	74.9 (0.0)	74.9	17	27.7 (0.0)	27.7
3	59.1 (0.0)	59.1	18	85.9 (0.0)	85.9
4	28.2 (–0.1)	28.1	19	84.5 (+0.1)	84.6
5	37.1 (–0.2)	36.9	20	34.0 (0.0)	34.0
6	74.4 (0.0)	74.4	21	26.6 (0.0)	26.6
7	86.5 (+0.1)	86.6	22	86.8 (0.0)	86.8
8	23.0 (0.0)	23.0	23	70.6 (–0.1)	70.5
9	38.7 (–0.3)	38.4	24	24.0 (0.0)	24.0
10	71.4 (0.0)	71.4	25	23.7 (0.0)	23.7
11	76.8 (–0.2)	76.6	26	20.1 (0.0)	20.1
12	21.3 (0.0)	21.3	27	21.3 (–0.1)	21.2
13	21.5 (0.0)	21.5	28	21.5 (+0.1)	21.6
14	75.4 (–0.1)	75.3	29	23.6 (+0.1)	23.7
15	84.5 (+0.1)	84.6	30	27.6 (0.0)	27.6

[a] An internal standard ($CDCl_3$: 77.0 ppm). [b] Cited from ref. [14]. An internal standard is $CDCl_3$: 77.0 ppm for the ^{13}C-NMR spectrum of the natural product reported by Fernández and Daranas et al.

Biological experimental procedure and data

Cell culture. HeLa (human cervical carcinoma) and HT-29 (human colon carcinoma) cells were cultured in DMEM (HeLa) or MacCoy's 5A (HT-29) culture medium containing 10% (*v/v*) heat-inactivated fetal bovine serum (FBS), 100 units mL^{-1} penicillin, and 100 µg mL^{-1} streptomycin. P388 (mouse leukemia) cells were cultured in RPMI-1640 supplemented with 10% FBS, 1.5% HEPES buffer, 100 units mL^{-1} penicillin, and 100 µg mL^{-1} streptomycin. The cultures were maintained in a humidified atmosphere of 5% CO_2 at 37 °C.

Cell growth inhibition assay. The effects of compounds in the proliferation of tumor cell lines were evaluated by a WST-8 (Dojindo, Kumamoto, Japan) reagent.[15] HeLa and HT-29 cells were seeded into 96-well plates at a density of 1.0 × 10^3 cells and 3.0 × 10^3 cells in 50 µL per well, respectively. After 24 h, the cells were treated with sample solutions (50 µL) containing different concentrations of the test compounds dissolved in DMSO [final concentration of DMSO was less than 0.1% (*v/v*)] and incubated for 72 h at 37 °C with 5% CO_2. P388 cells were seeded into 96-well plates at a density of 0.5 × 10^3 cells in 50 µL, and then the cells were treated with sample solutions containing different concentrations of the test compounds and incubated for 72 h under the same conditions. After incubation, 10 µL of WST-8 solution was added and the cells were then incubated for 1 h (HeLa and HT-29) or 3 h (P388). The absorbance of each well at 450 nm was measured using microplate reader. The IC_{50} (50% inhibitory concentration of cellular proliferation with respect to the untreated control) value was determined for each compound by GraphPad Prism 8 for Windows (GraphPad software Inc, California, USA).

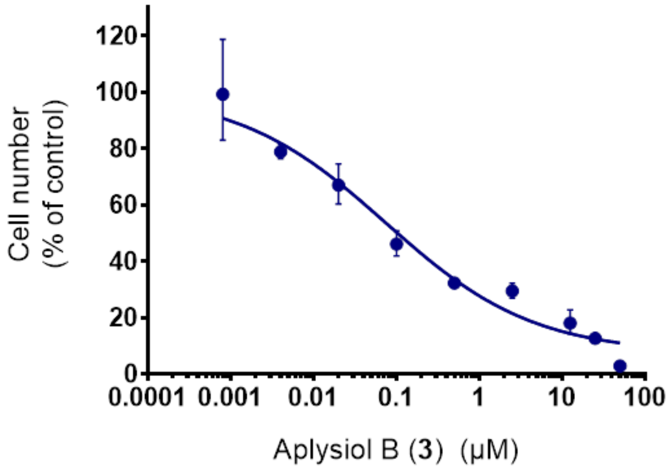

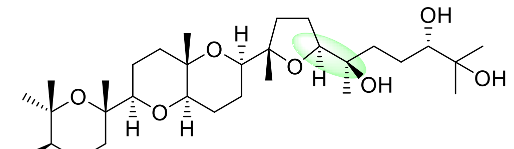

aplysiol B (**3**)

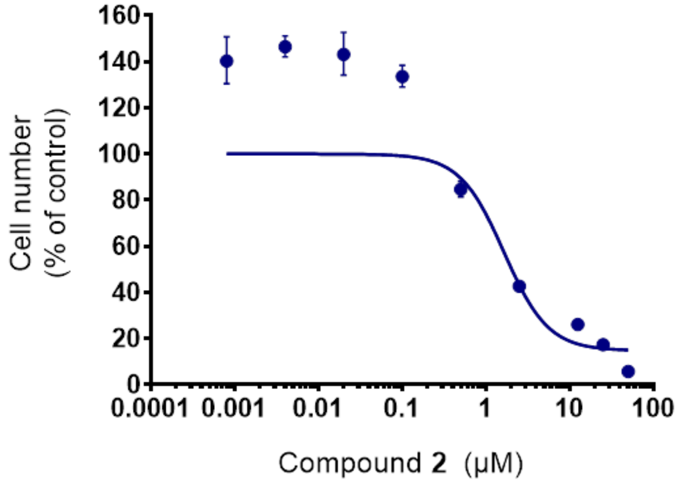

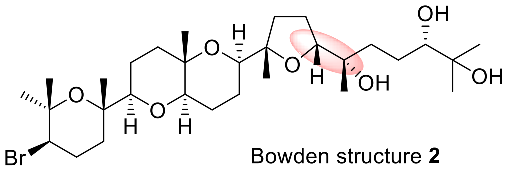

Bowden structure **2**

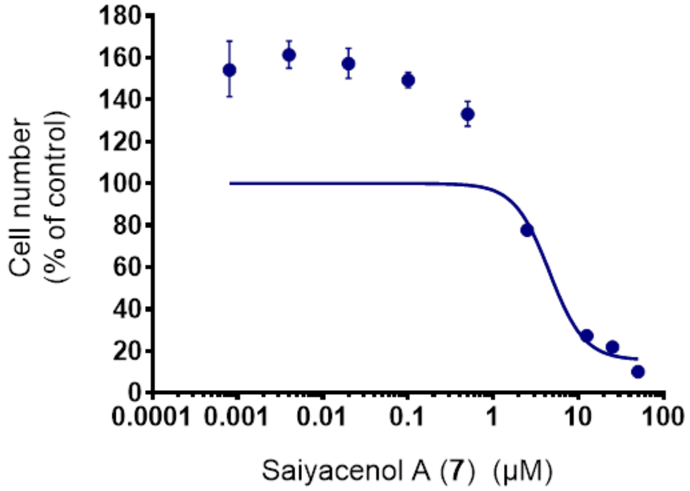

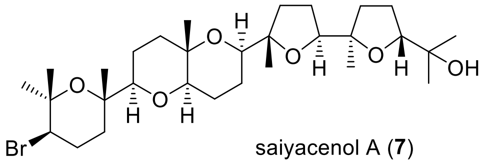

saiyacenol A (**7**)

Figure S1. Cell growth inhibition dose-response curve of compounds (P388).

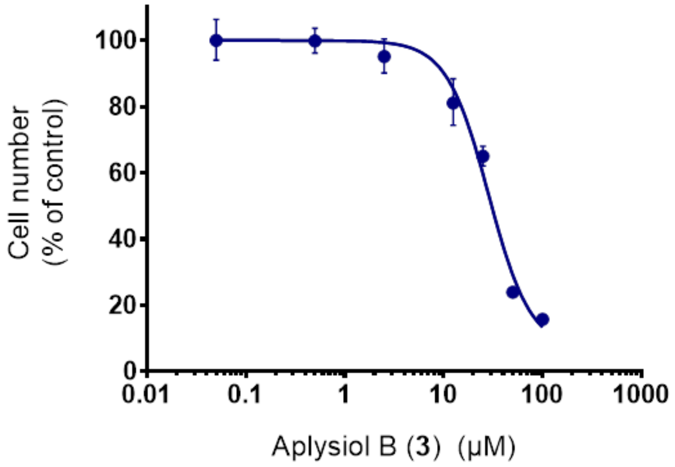

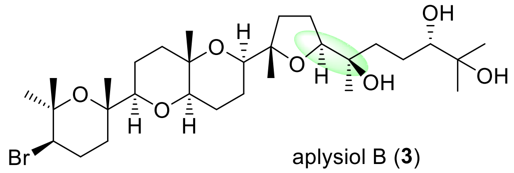

aplysiol B (**3**)

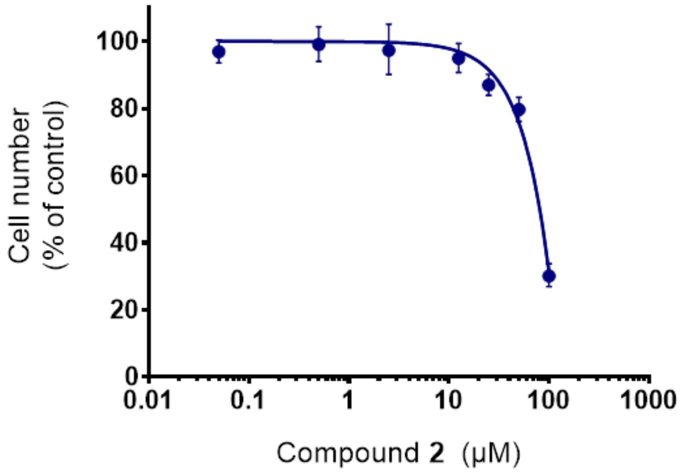

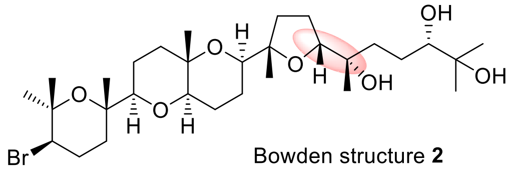

Bowden structure **2**

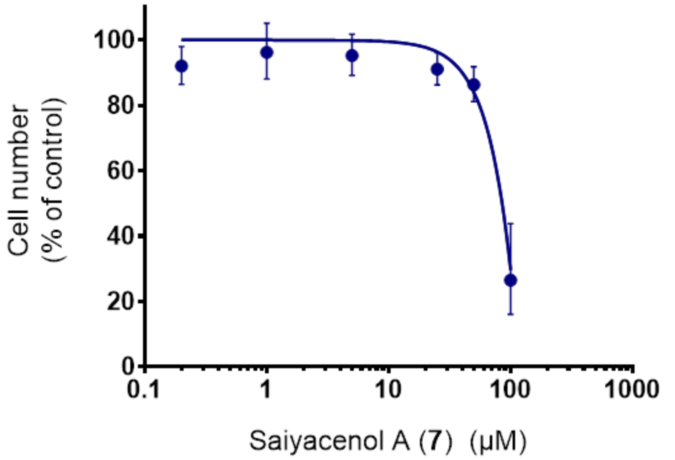

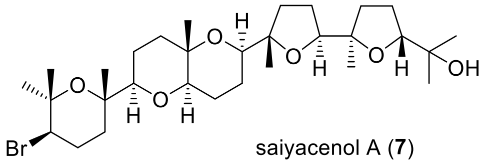

saiyacenol A (**7**)

Figure S2. Cell growth inhibition dose-response curve of compounds (HT-29).

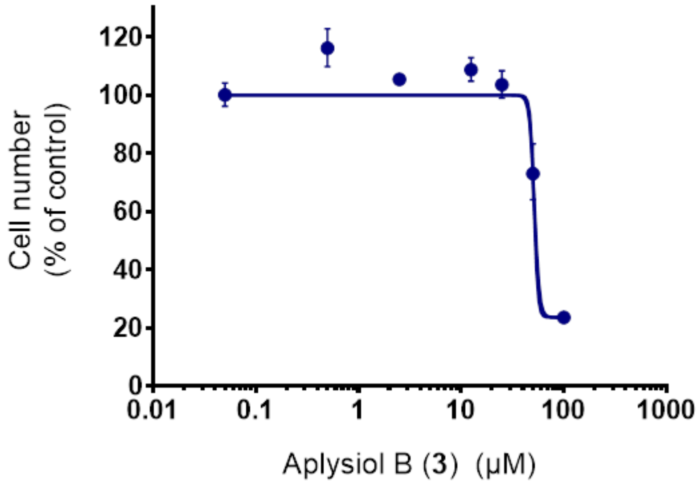

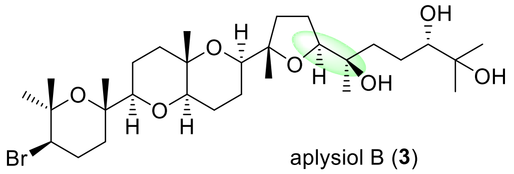

aplysiol B (**3**)

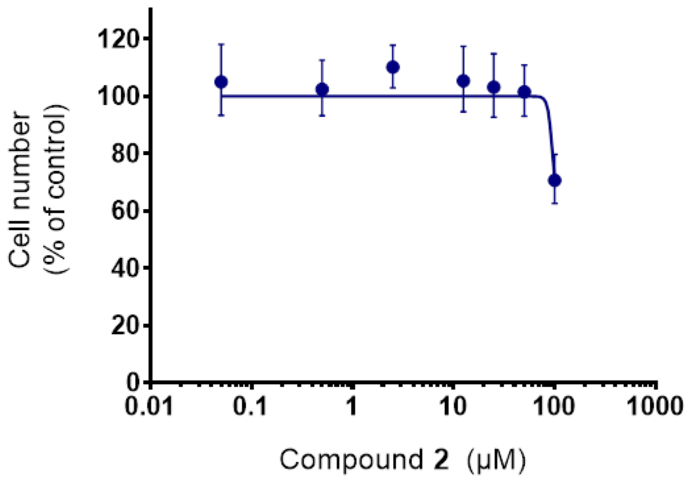

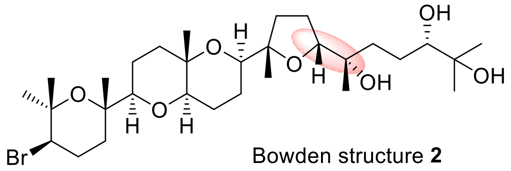

Bowden structure **2**

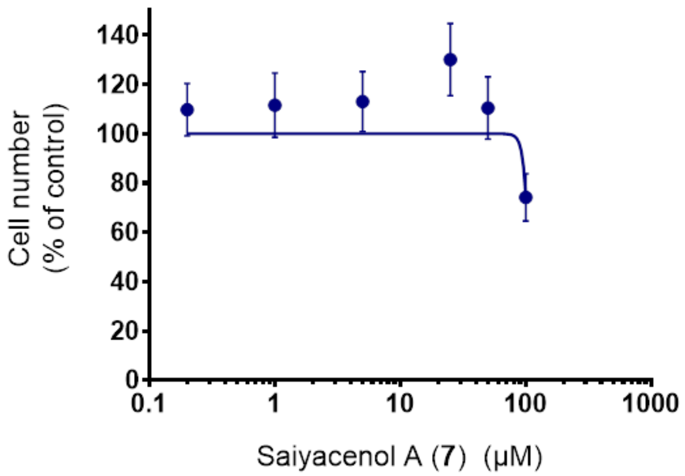

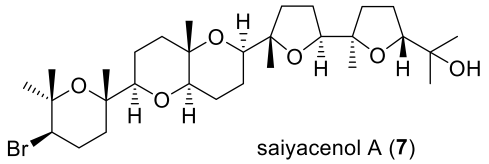

saiyacenol A (**7**)

Figure S3. Cell growth inhibition dose-response curve of compounds (HeLa).

References

[1] Y. Morimoto, Y. Nishikawa, M. Takaishi, *J. Am. Chem. Soc.* **2005**, *127*, 5806–5807.

[2] A. Hoshino, H. Nakai, M. Morino, K. Nishikawa, T. Kodama, K. Nishikibe, Y. Morimoto, *Angew. Chem. Int. Ed.* **2017**, *56*, 3064–3068; *Angew. Chem.* **2017**, *129*, 3110–3114.

[3] M. Hashimoto, H. Harigaya, M. Yanagiya, H. Shirahama, *J. Org. Chem.* **1991**, *56*, 2299–2311.

[4] a) T. Katsuki, K. B. Sharpless, *J. Am. Chem. Soc.* **1980**, *102*, 5974–5976; b) M. Hashimoto, T. Kan, K. Nozaki, M. Yanagiya, H. Shirahama, T. Matsumoto, *J. Org. Chem.* **1990**, *55*, 5088–5107.

[5] Y. Morimoto, T. Iwai, T. Kinoshita, *Tetrahedron Lett.* **2001**, *42*, 6307–6309.

[6] Z.-X. Wang, Y. Tu, M. Frohn, J.-R. Zhang, Y. Shi, *J. Am. Chem. Soc.* **1997**, *119*, 11224–11235.

[7] A. K. Chatterjee, D. P. Sanders, R. H. Grubbs, *Org. Lett.* **2002**, *4*, 1939–1942.

[8] S. A. Snyder, D. S. Treitler, A. P. Brucks, *J. Am. Chem. Soc.* **2010**, *132*, 14303–14314.

[9] A. R. B. Ola, A.-M. Babey, C. Motti, B. F. Bowden, *Aust. J. Chem.* **2010**, *63*, 907–914.

[10] M.-X. Zhao, Y. Shi, *J. Org. Chem.* **2006**, *71*, 5377–5379.

[11] E. Manzo, M. Gavagnin, G. Bifulco, P. Cimino, S. D. Micco, M. L. Ciavatta, Y. W. Guo, G. Cimino, *Tetrahedron* **2007**, *63*, 9970–9978.

[12] N.-Y. Ji, X.-M. Li, H. Xie, J. Ding, K. Li, L.-P. Ding, B.-G. Wang, *Helv. Chim. Acta* **2008**, *91*, 1940–1946.

[13] Y. Morimoto, H. Yata, Y. Nishikawa, *Angew. Chem. Int. Ed.* **2007**, *46*, 6481–6484; *Angew. Chem.* **2007**, *119*, 6601–6604.

[14] F. Cen-Pacheco, F. Mollinedo, J. A. Villa-Pulgarín, M. Norte, J. J. Fernández, A. H. Daranas, *Tetrahedron* **2012**, *68*, 7275–7279.

[15] a) M. Ishiyama, Y. Miyazono, K. Sasamoto, Y. Ohkura, K. Ueno, *Talanta* **1997**, *44*, 1299–1305; b) H. Tominaga, M. Ishiyama, F. Ohseto, K. Sasamoto, T. Hamamoto, K. Suzuki, M. Watanabe, *Anal. Commun.* **1999**, *36*, 47–50.

Author contributions

Kento Nishikibe (type: investigation and data curation; degree: equal) and Keisuke Nishikawa (type: project administration, funding acquisition, investigation, data curation, and writing of original draft; degree: lead) contributed to the syntheses of aplysiol B (laurenmariannol) and saiyacenol A. Keisuke Nishikawa and Yoshiki Morimoto (type: project administration, funding acquisition, and writing of original draft; degree: lead) contributed to the writing of research proposals and the manuscript. Momochika Kumagai (type: validation and formal analysis; degree: supporting) contributed to the evaluation of the biological activity. Matsumi Doe (type: validation and formal analysis; degree: supporting) contributed to the NMR analysis of newly synthesized compounds.

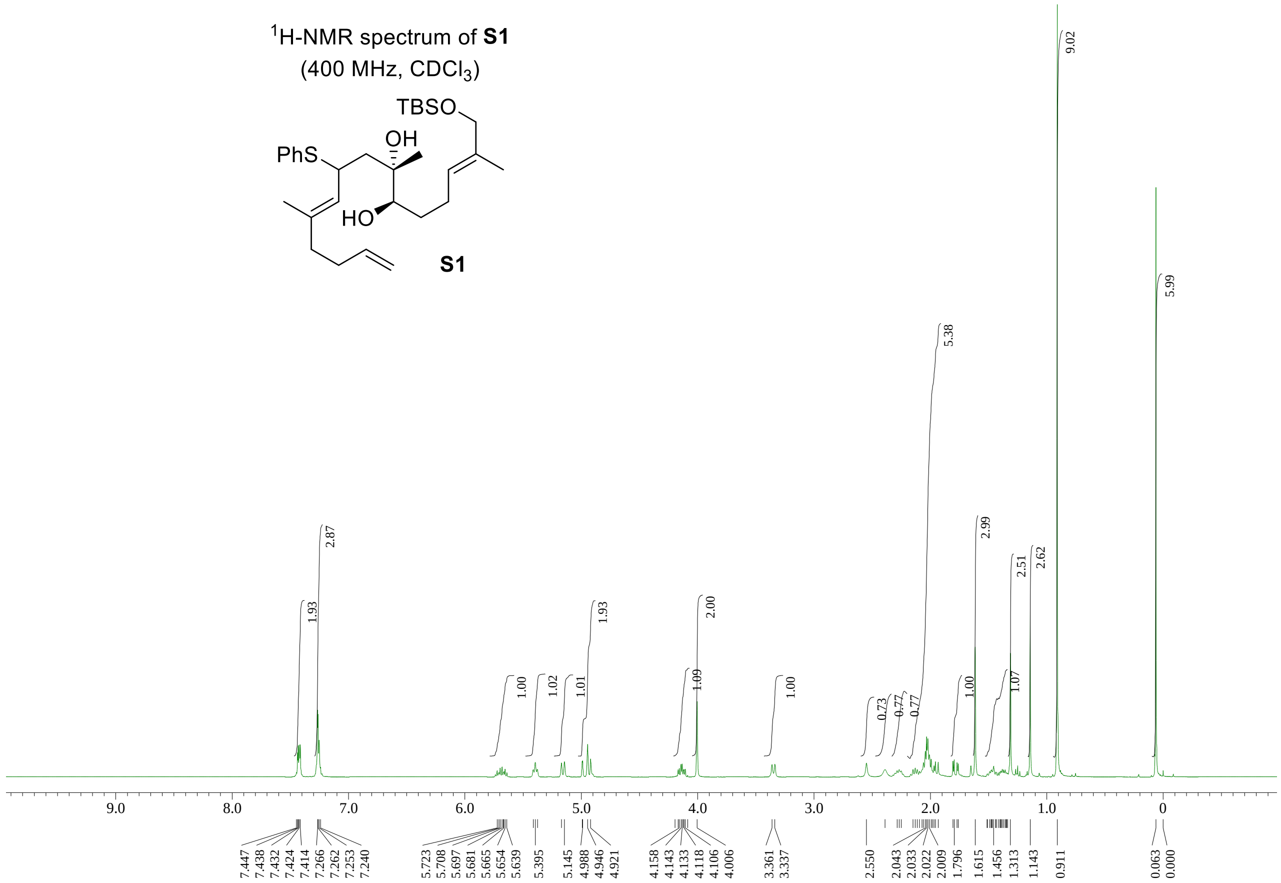
^{1}H-NMR spectrum of **S1**
(400 MHz, $CDCl_3$)
TBSO
OH
PhS
HO
S1
9.02
5.99
5.38
2.87
1.93
1.00
1.02
1.01
1.93
1.09
2.00
1.00
0.73
0.77
0.77
1.00
2.99
2.51
2.62
1.07
9.0 8.0 7.0 6.0 5.0 4.0 3.0 2.0 1.0 0
7.447 7.438 7.432 7.424 7.414 7.266 7.262 7.253 7.240
5.723 5.708 5.697 5.681 5.665 5.654 5.639 5.395 5.145 4.988 4.946 4.921
4.158 4.143 4.133 4.118 4.106 4.006 3.361 3.337
2.550 2.043 2.033 2.022 2.009 1.796 1.615 1.456 1.313 1.143 0.911
0.063 0.000

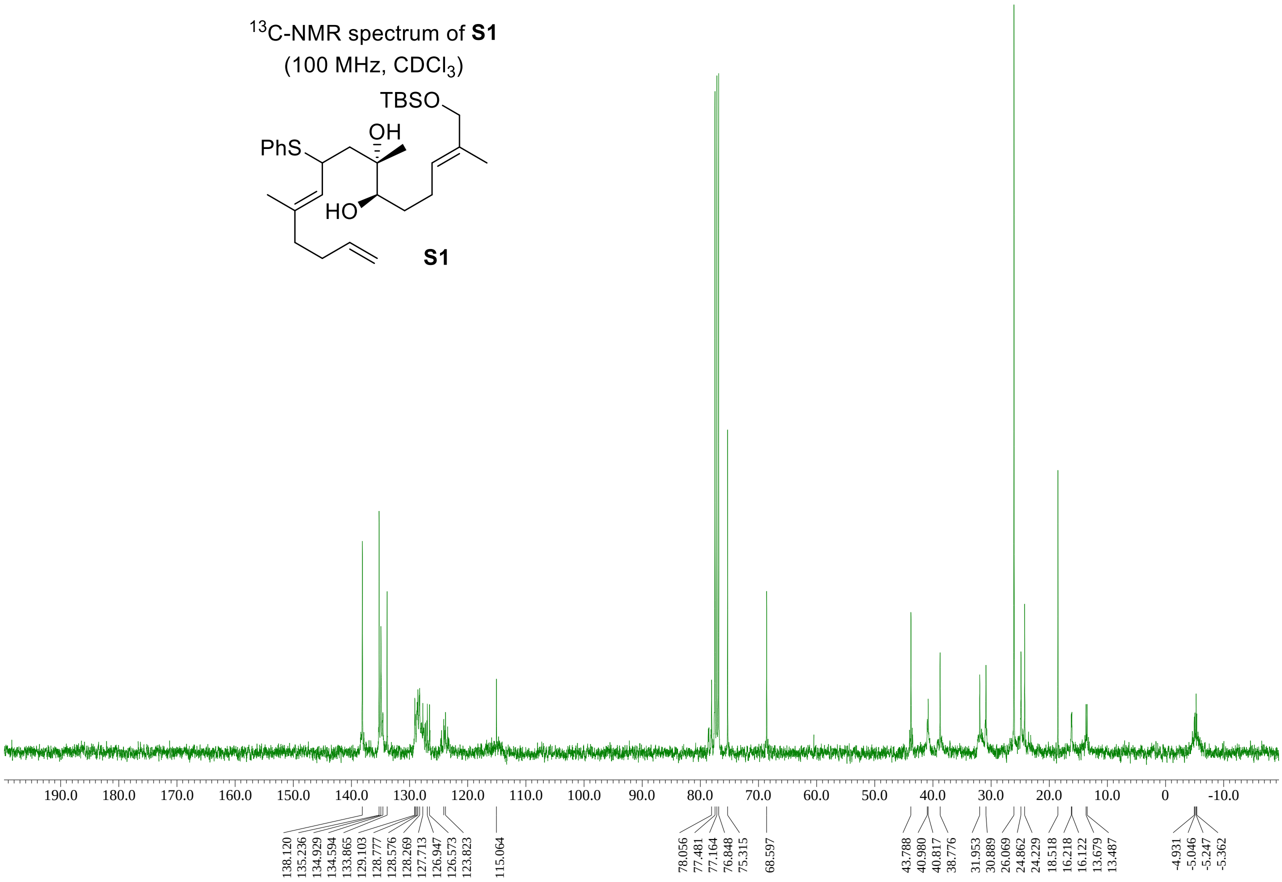
^{13}C-NMR spectrum of **S1**
(100 MHz, $CDCl_3$)
TBSO
OH
PhS
HO
S1
190.0 180.0 170.0 160.0 150.0 140.0 130.0 120.0 110.0 100.0 90.0 80.0 70.0 60.0 50.0 40.0 30.0 20.0 10.0 0 -10.0
138.120 135.236 134.929 134.594 133.865 129.103 128.777 128.576 128.269 127.713 126.947 126.573 123.823 115.064
78.056 77.481 77.164 76.848 75.315 68.597
43.788 40.980 40.817 38.776 31.953 30.889 26.069 24.862 24.229 18.518 16.218 16.122 13.679 13.487
-4.931 -5.046 -5.247 -5.362

^{1}H-NMR spectrum of **S2**
(400 MHz, $CDCl_3$)

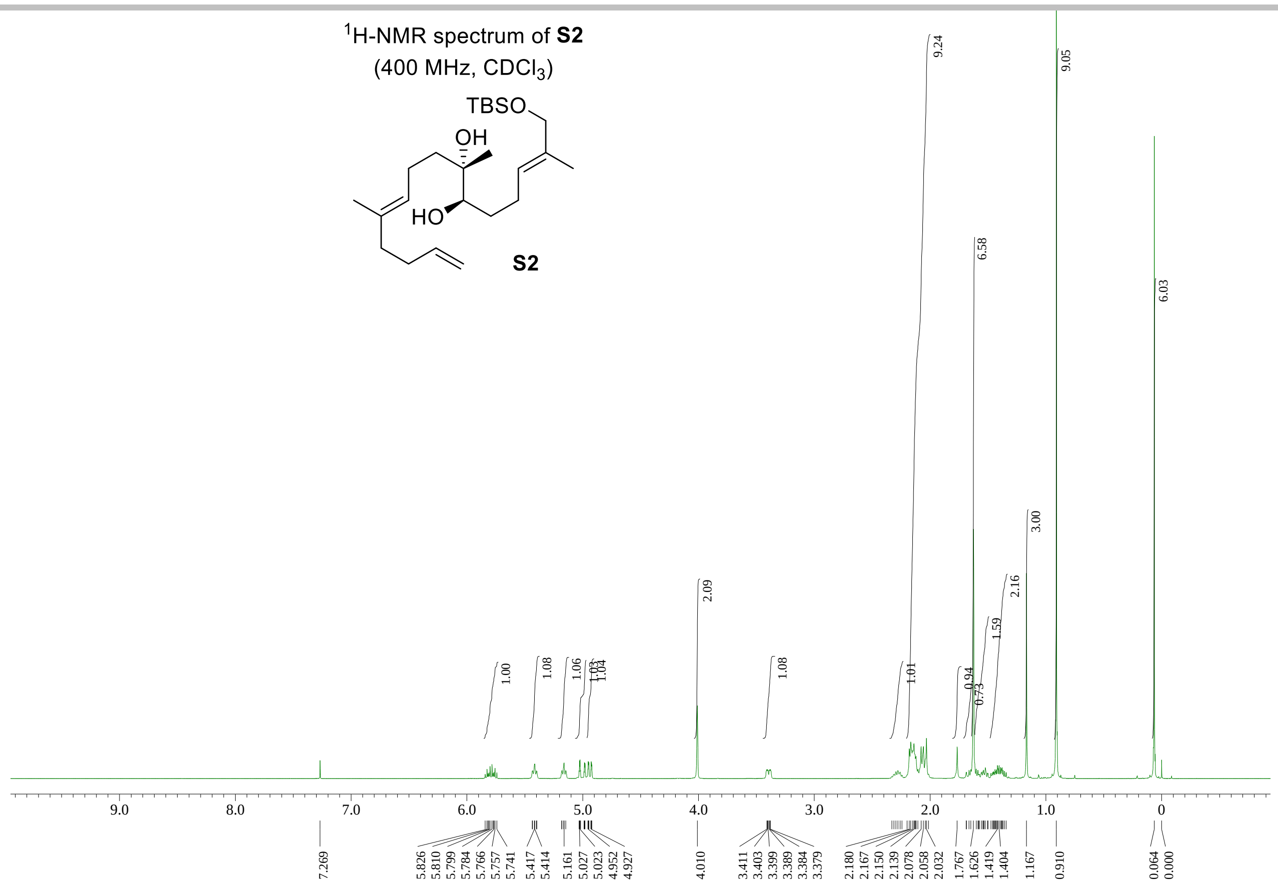

^{13}C-NMR spectrum of **S2**
(100 MHz, $CDCl_3$)

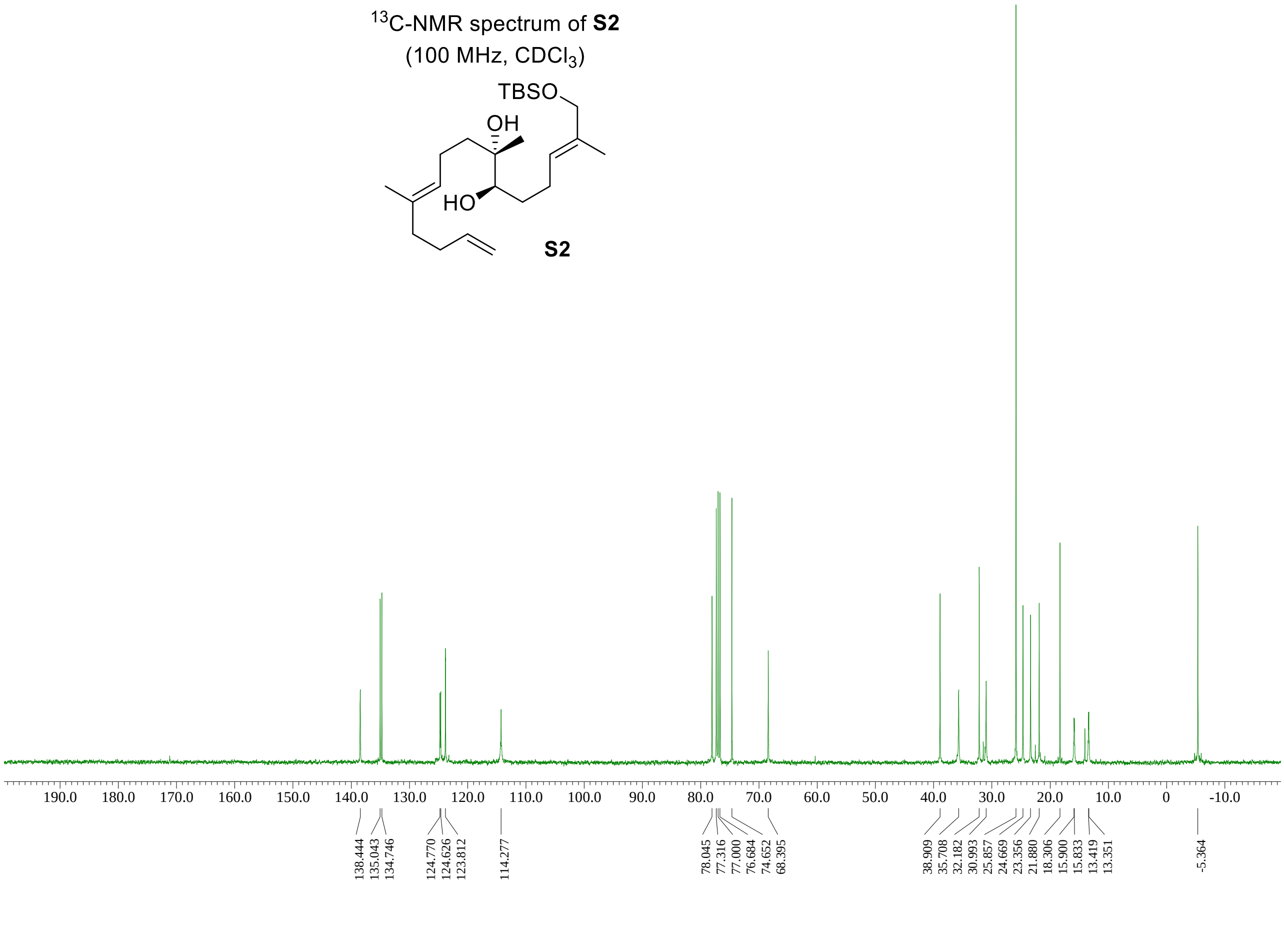

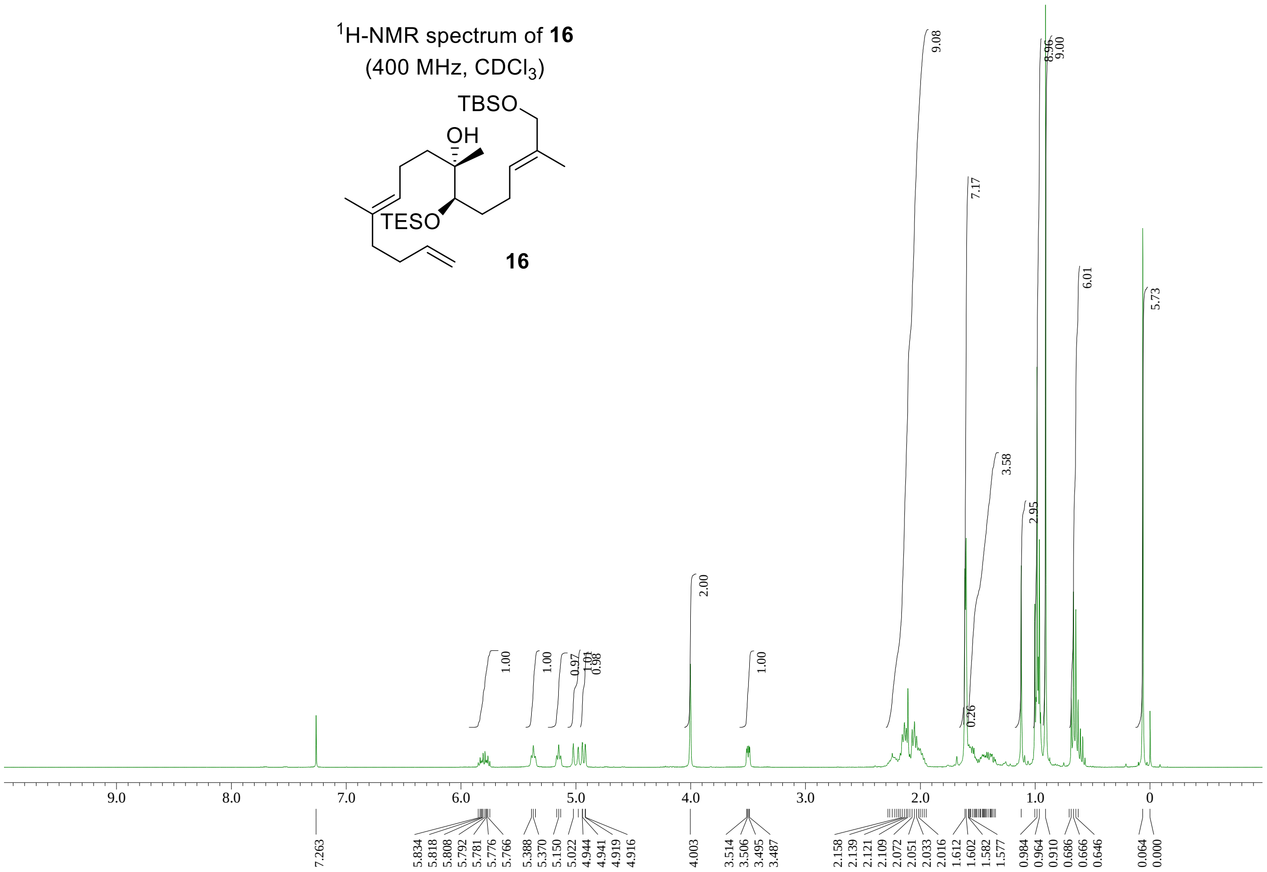
^{1}H-NMR spectrum of **16**
(400 MHz, $CDCl_3$)
TBSO
OH
TESO
16

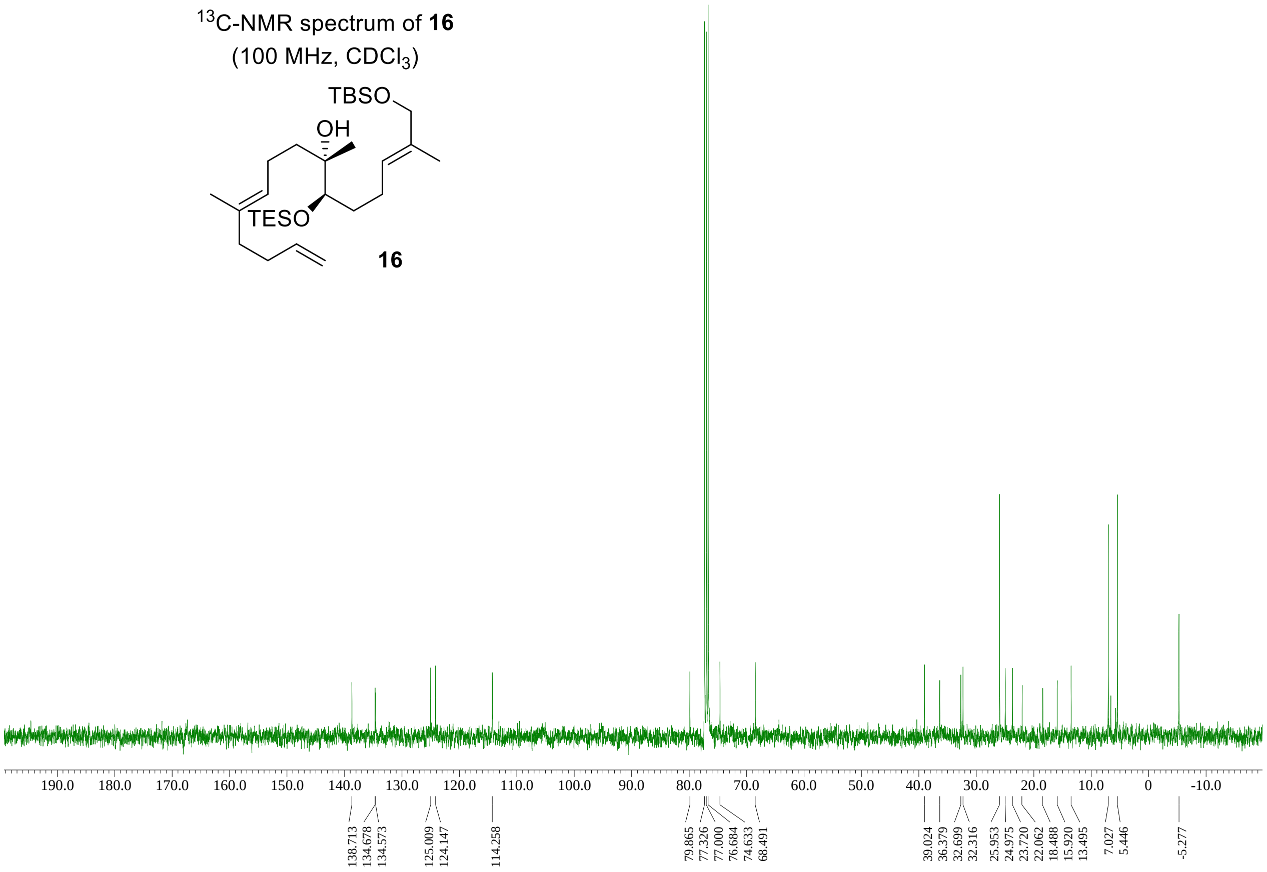
^{13}C-NMR spectrum of **16**
(100 MHz, $CDCl_3$)
TBSO
OH
TESO
16

^{1}H-NMR spectrum of **17**
(400 MHz, $CDCl_3$)

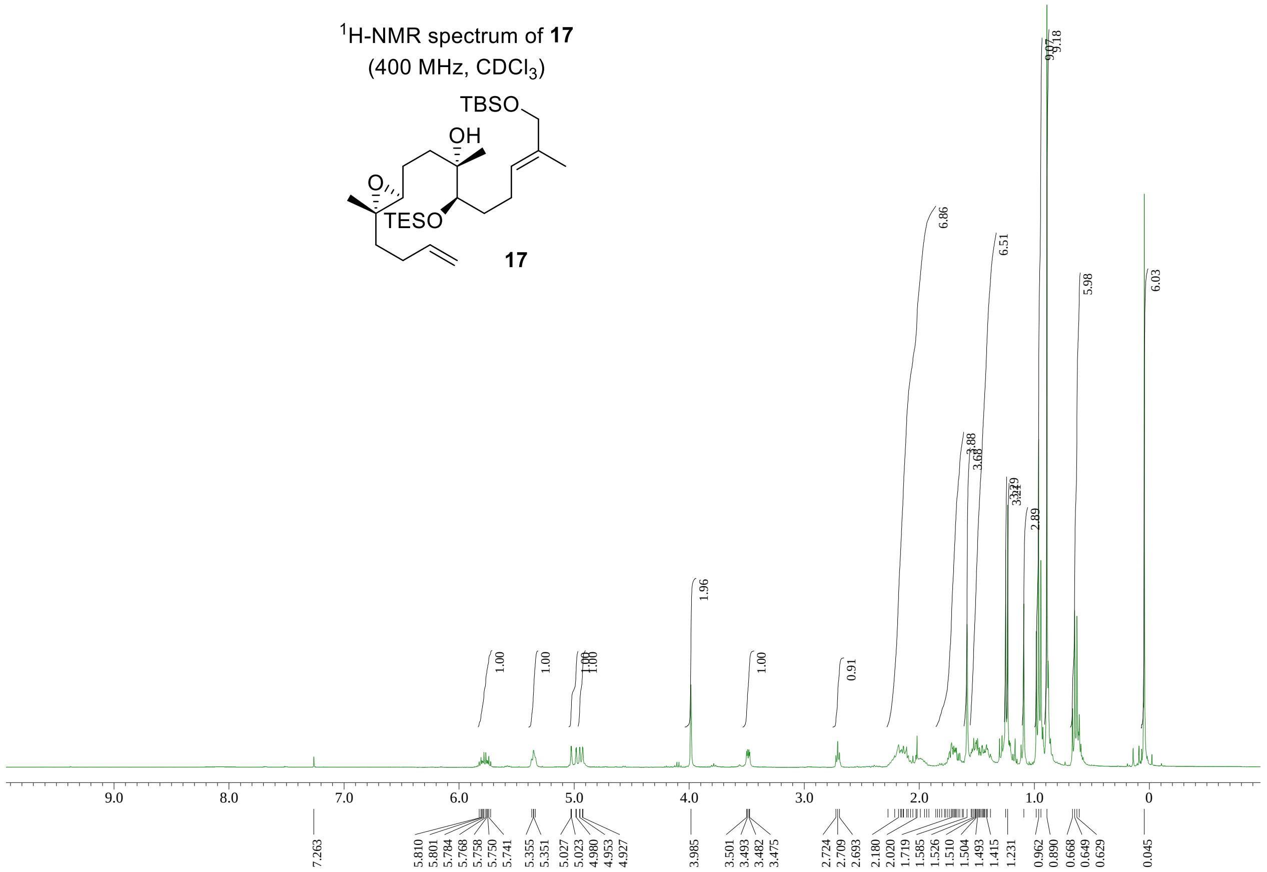

^{13}C-NMR spectrum of **17**
(100 MHz, $CDCl_3$)

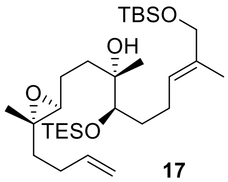

17

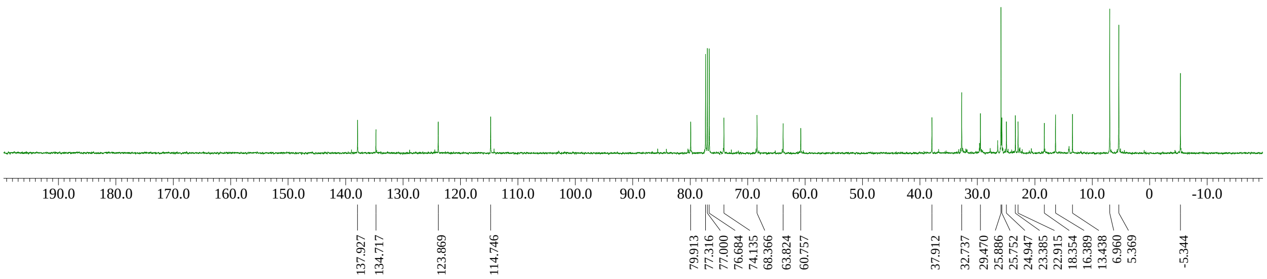

^{1}H-NMR spectrum of **S3**
(400 MHz, $CDCl_3$)

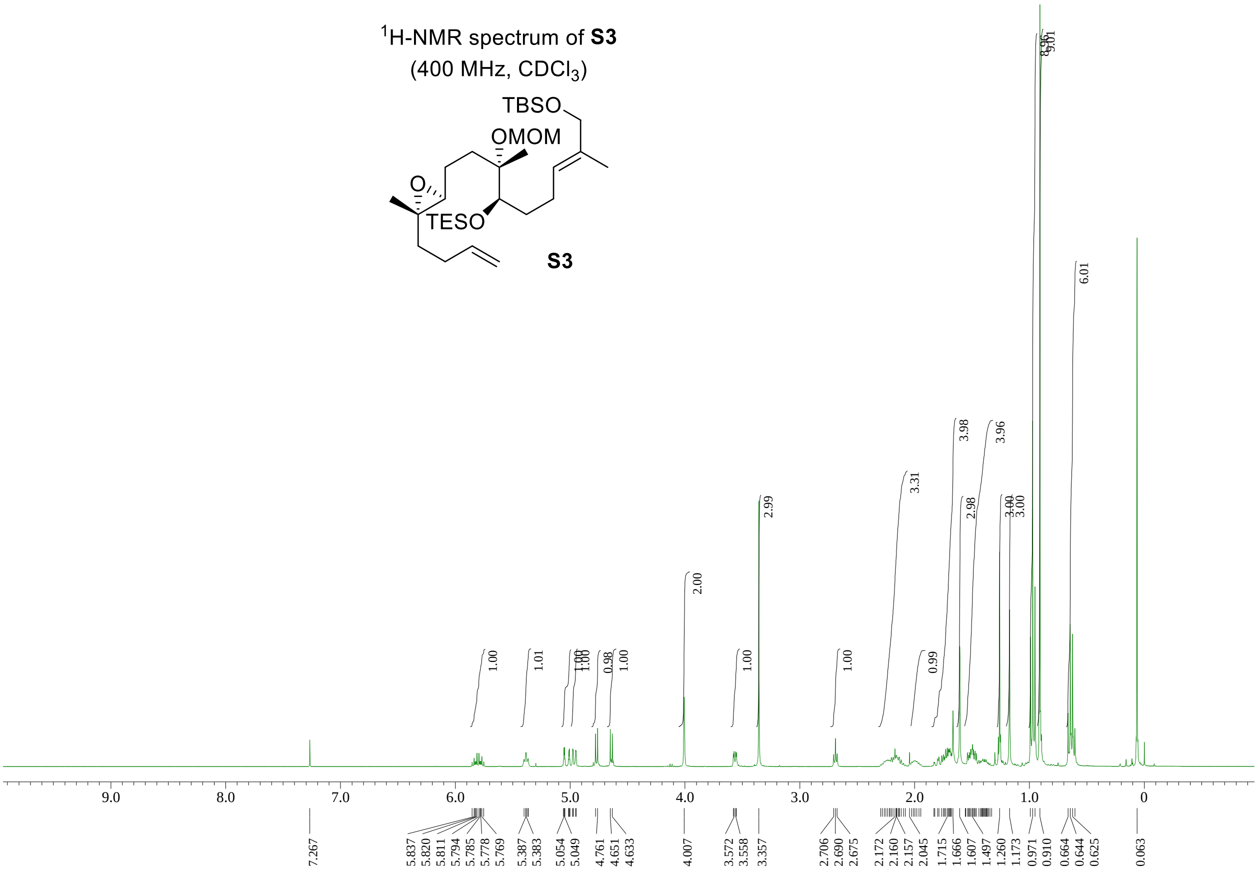

S3

^{13}C-NMR spectrum of **S3**
(100 MHz, $CDCl_3$)

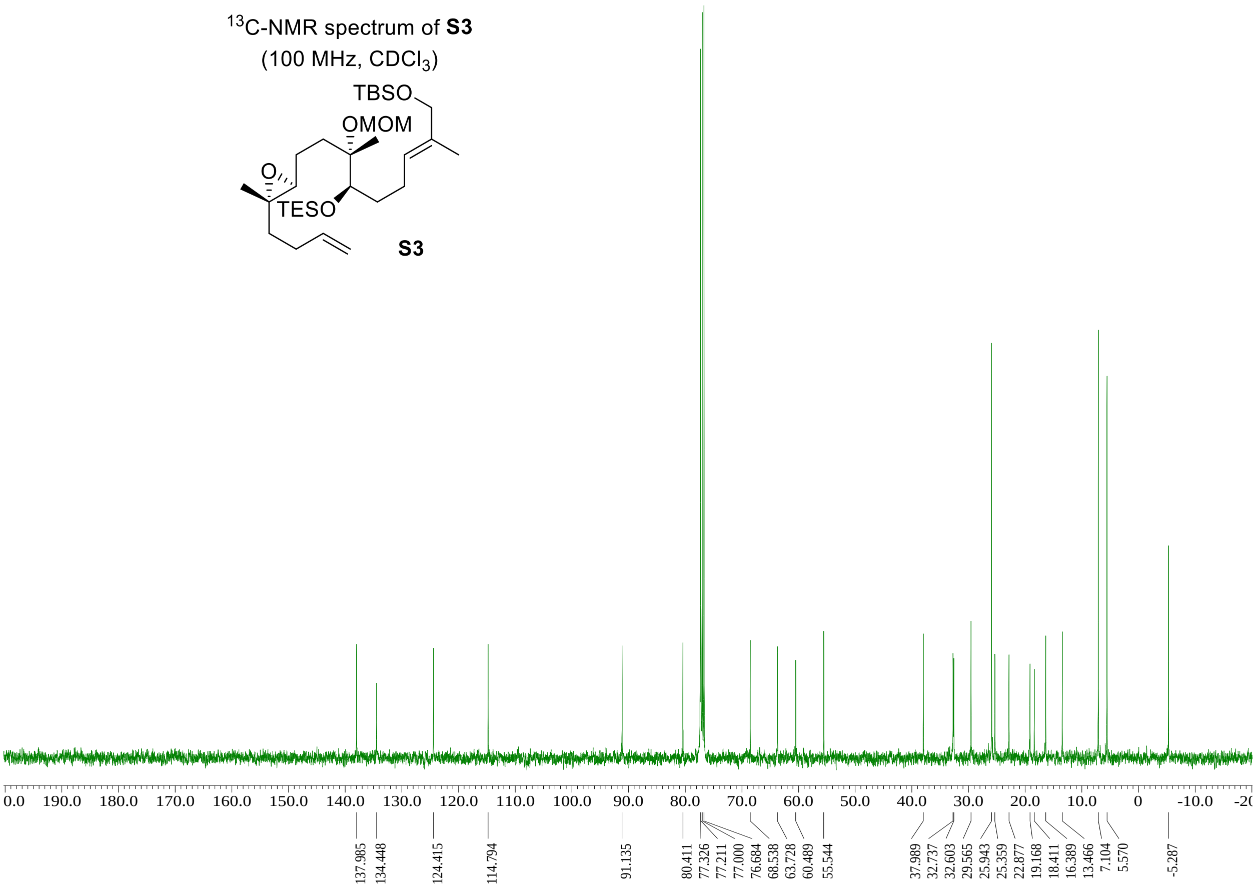

S3

^{1}H-NMR spectrum of **S4**
(400 MHz, $CDCl_3$)

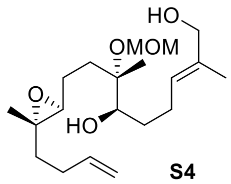

S4

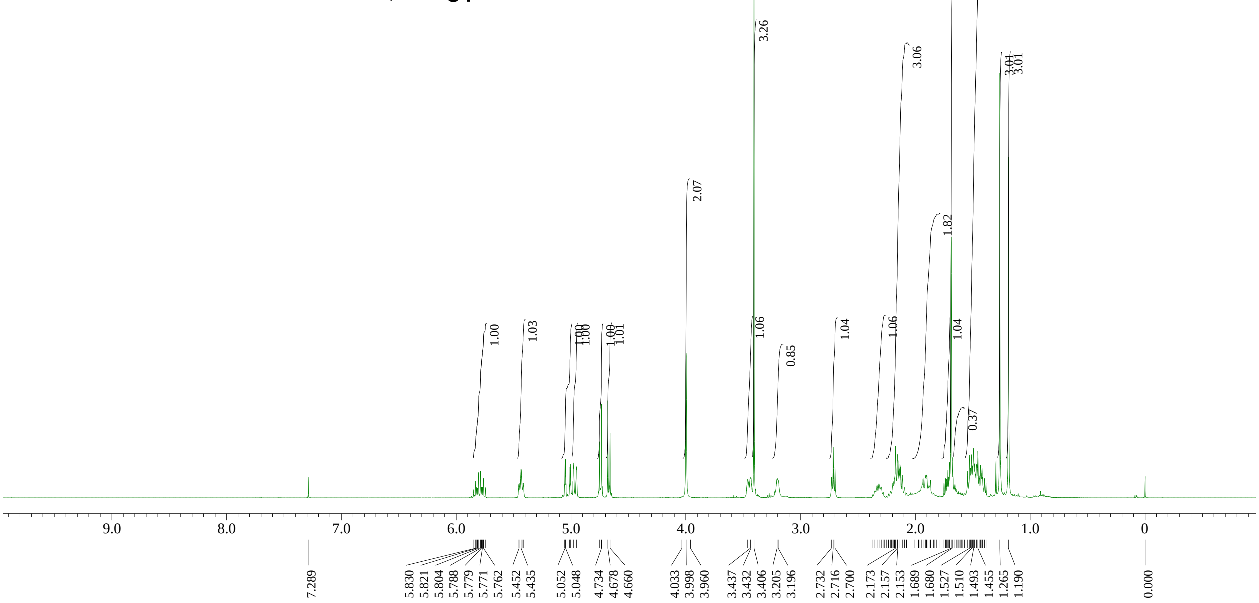

^{13}C-NMR spectrum of **S4**
(100 MHz, $CDCl_3$)

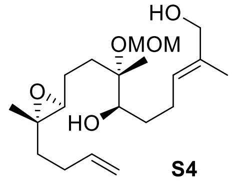

S4

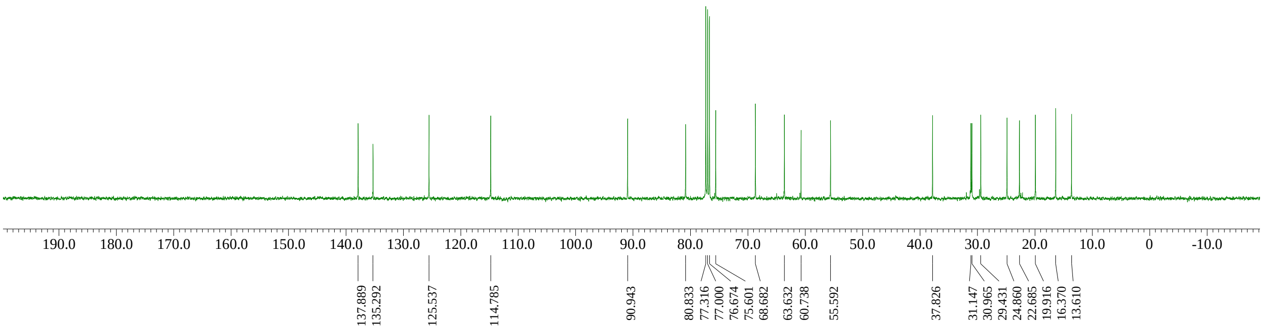

^{1}H-NMR spectrum of **18**
(600 MHz, $CDCl_3$)

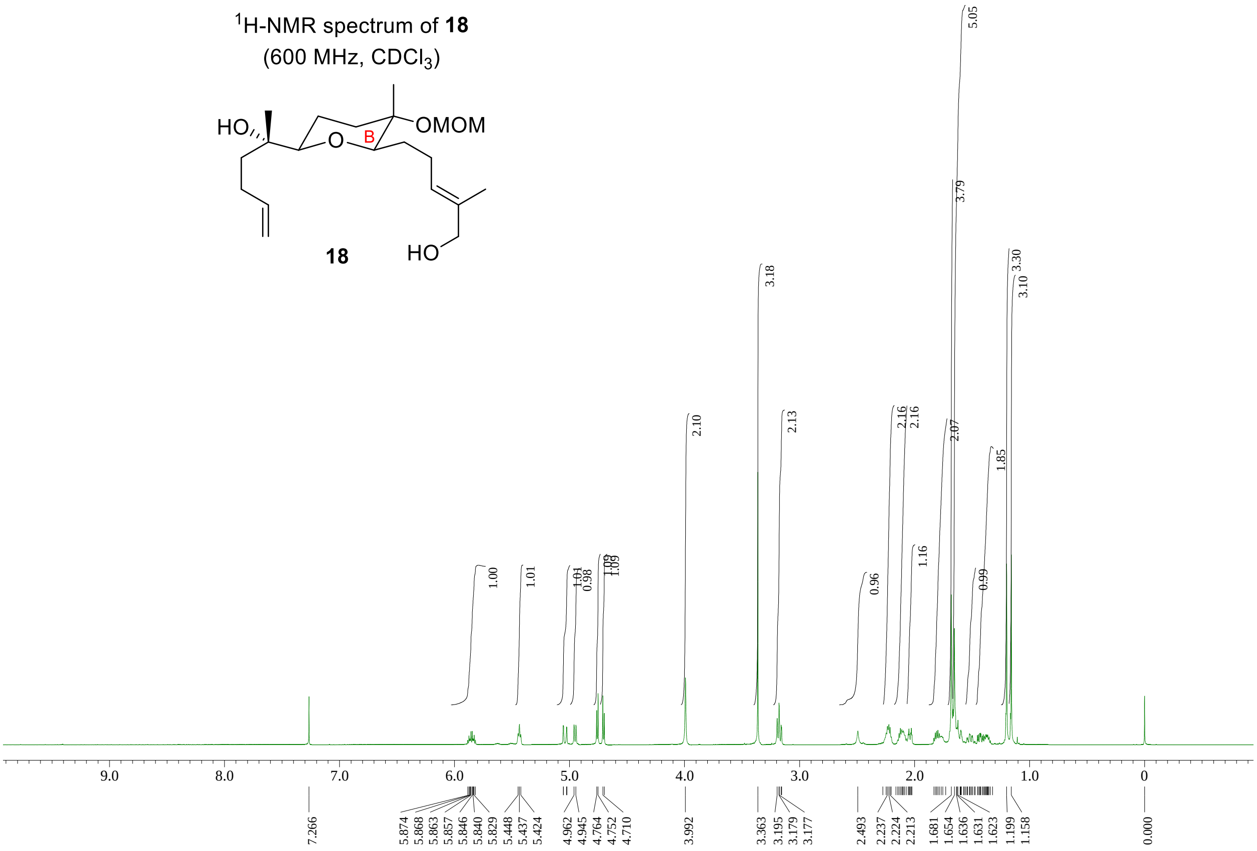

^{13}C-NMR spectrum of **18**
(150 MHz, $CDCl_3$)

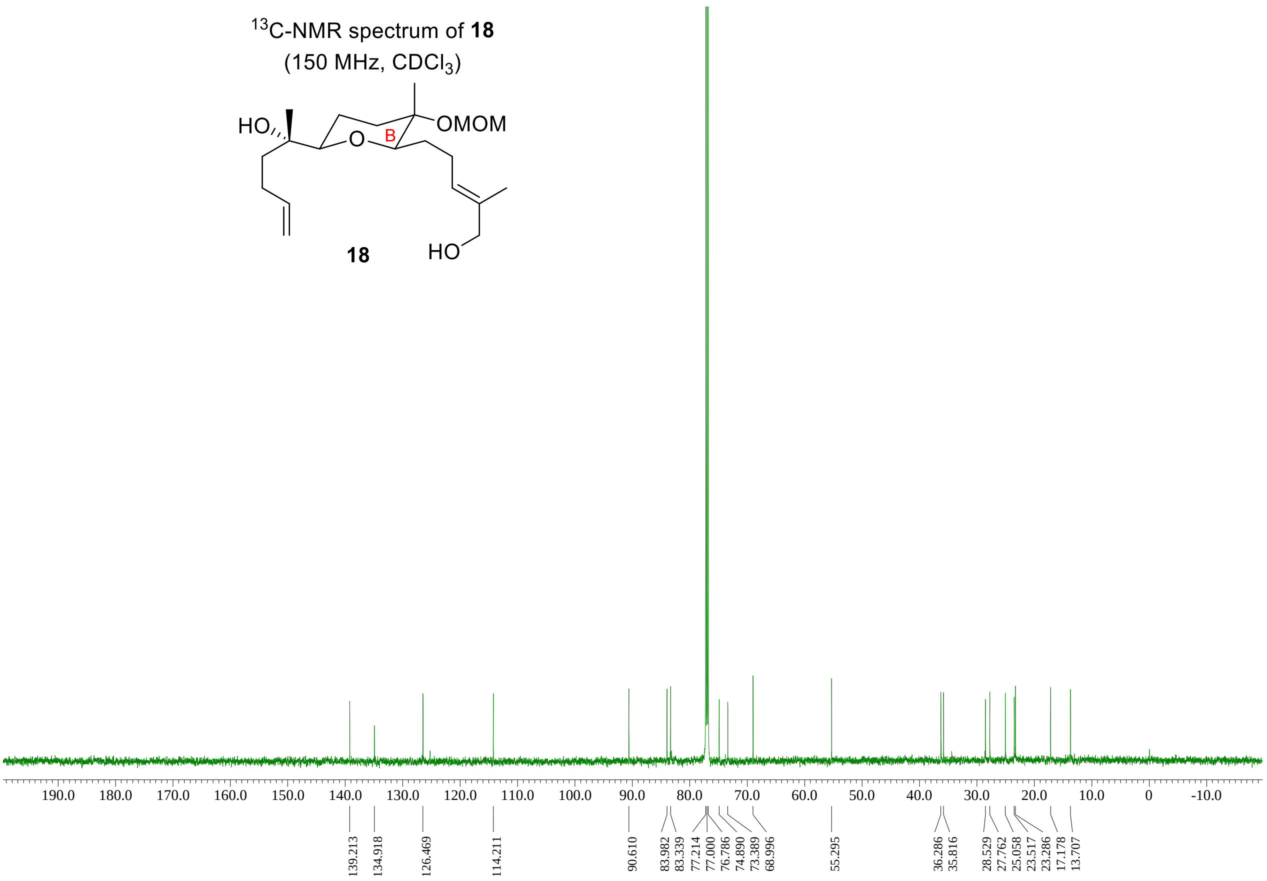

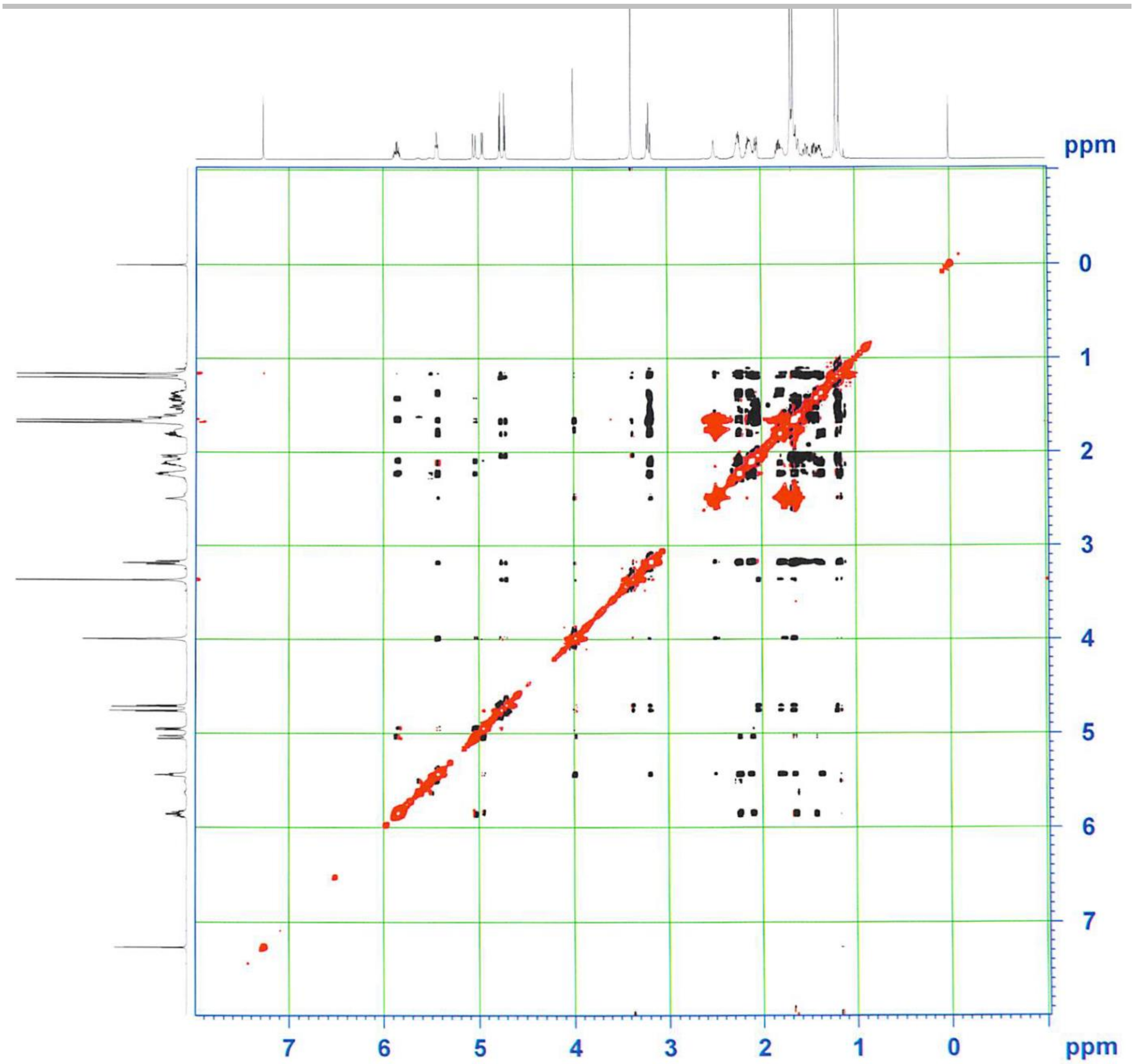

NOESY spectrum of **18**
(25 °C)
($CDCl_3$)

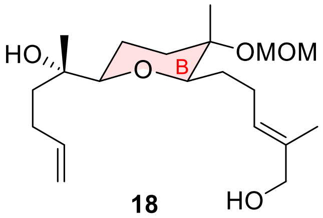

18

Observed NOEs

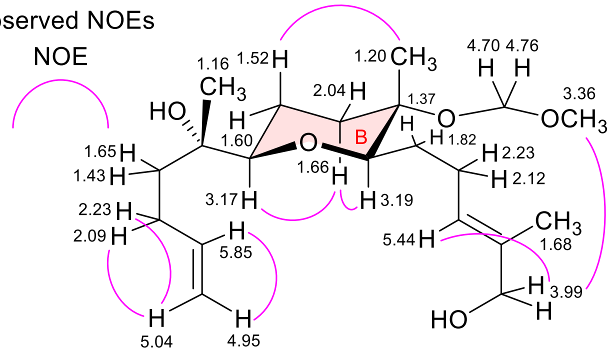

^{1}H-NMR spectrum of **S5**
(400 MHz, $CDCl_3$)

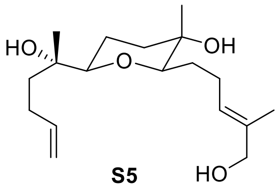

S5

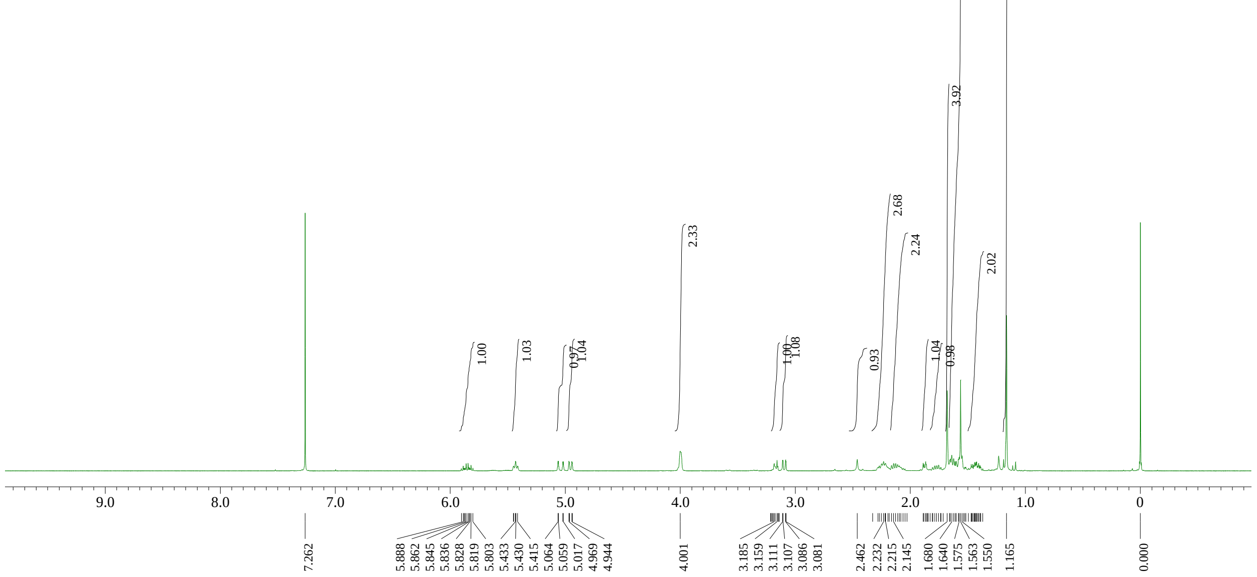

^{13}C-NMR spectrum of **S5**
(100 MHz, $CDCl_3$)

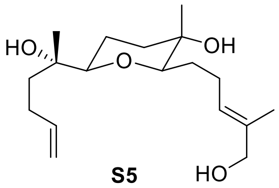

S5

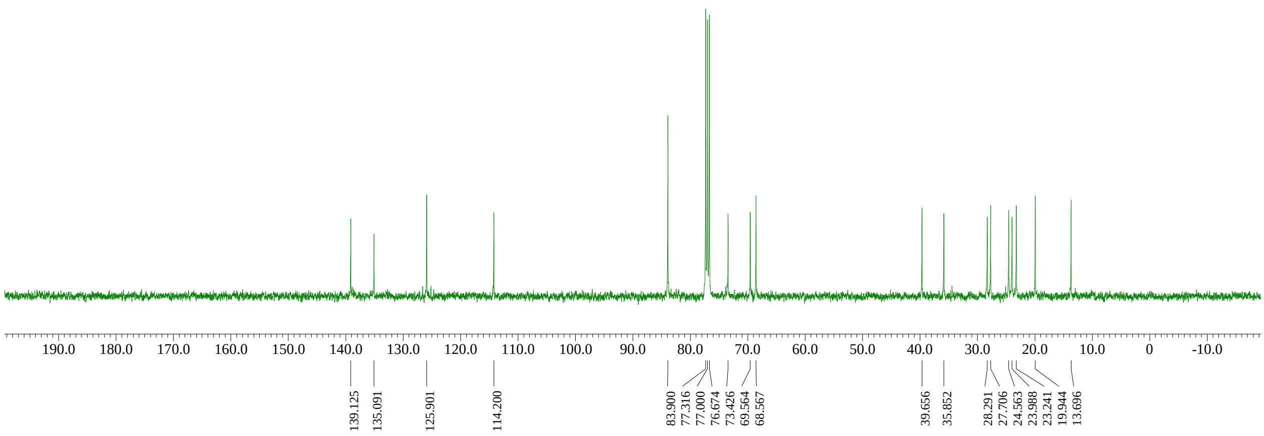

^{1}H-NMR spectrum of **19**
(600 MHz, $CDCl_3$)

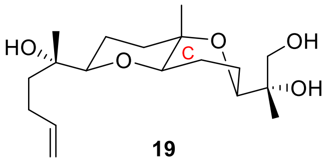

19

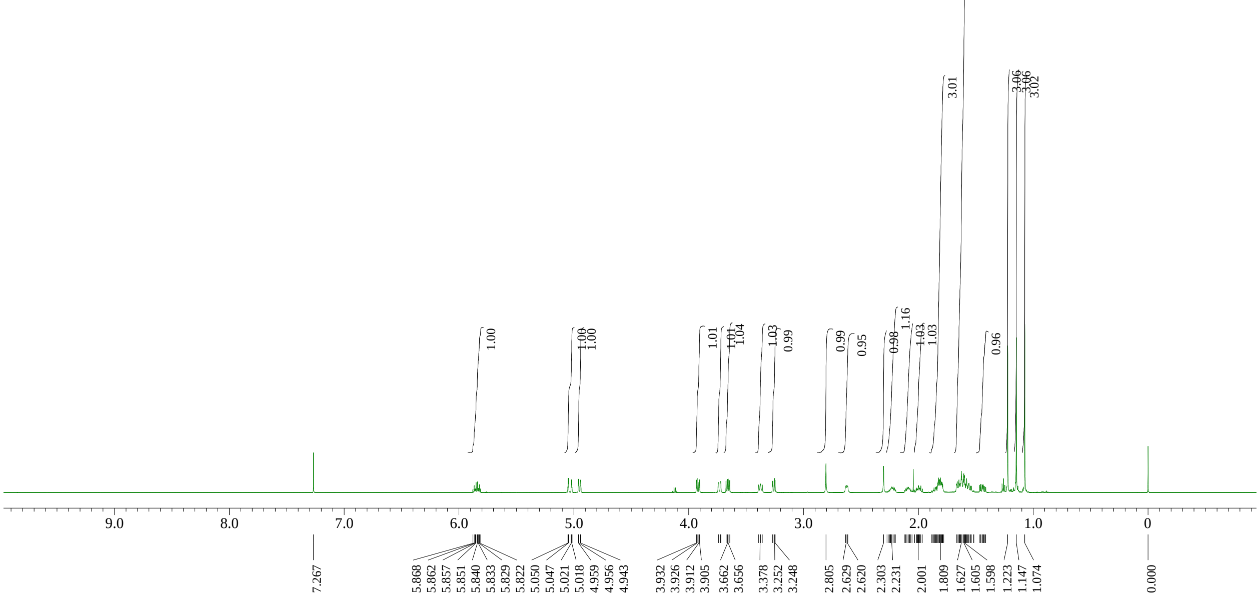

^{13}C-NMR spectrum of **19**
(150 MHz, $CDCl_3$)

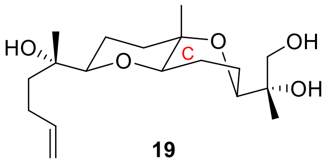

19

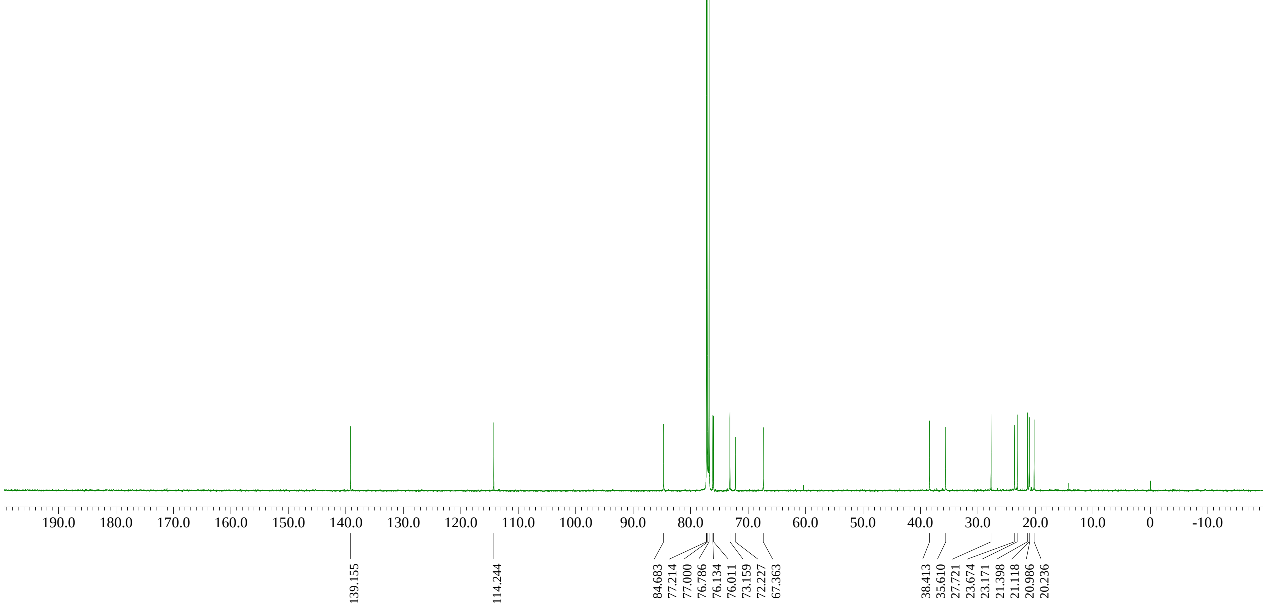

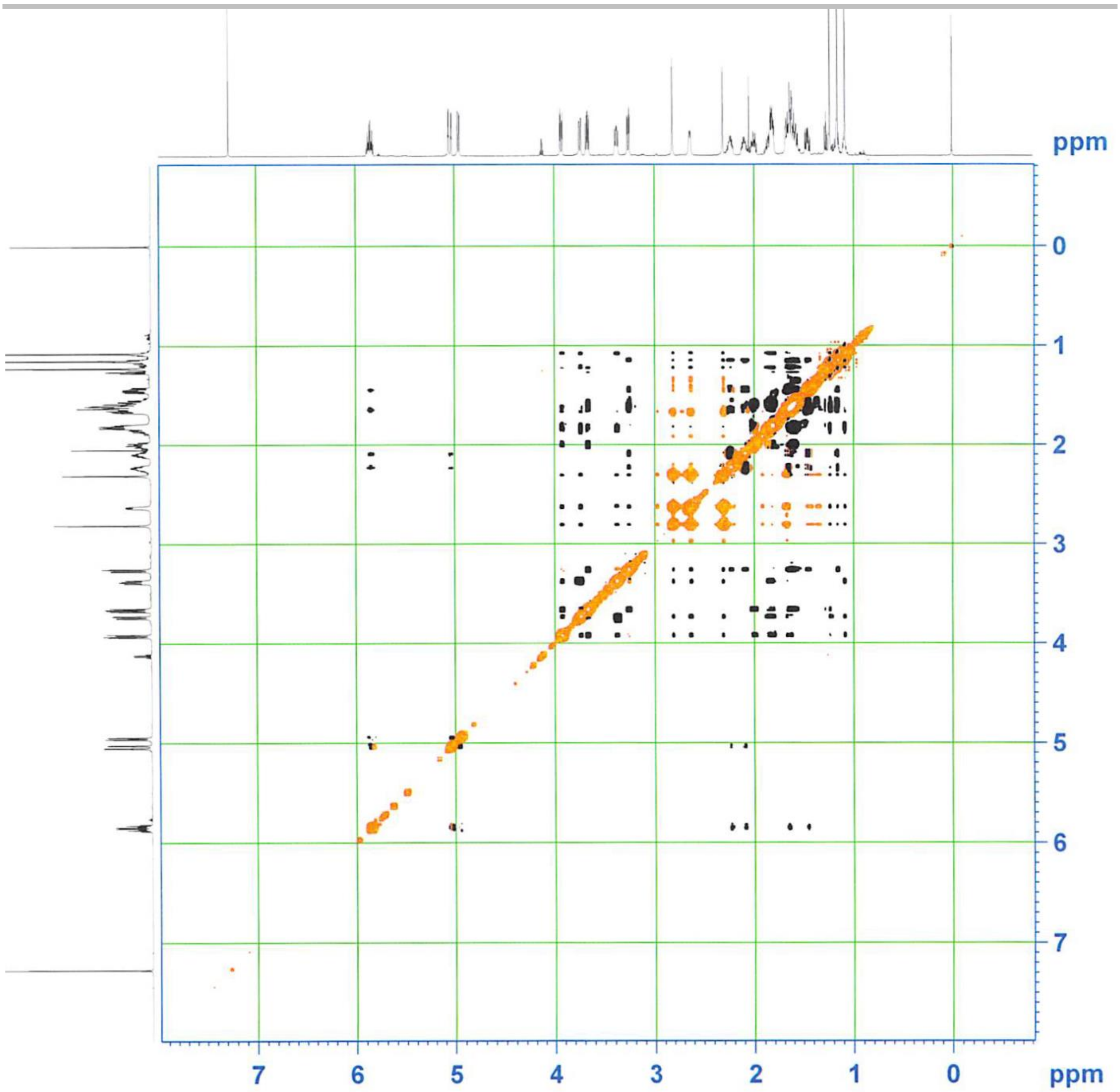

NOESY spectrum of **19**
(25 °C)
($CDCl_3$)

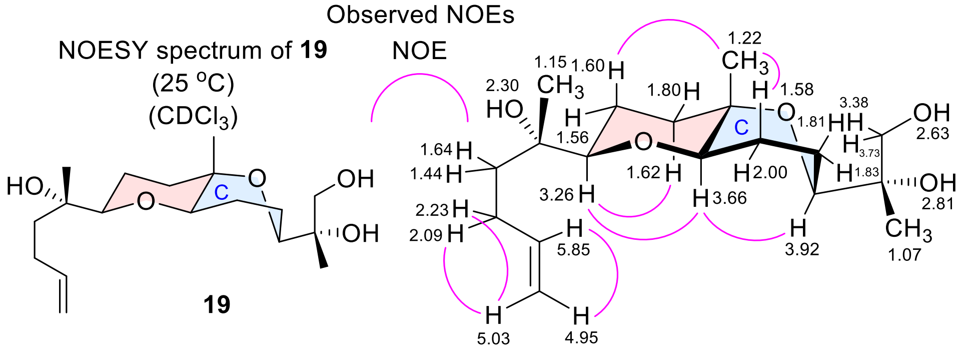

19

^{1}H-NMR spectrum of **12**
(400 MHz, $CDCl_3$)

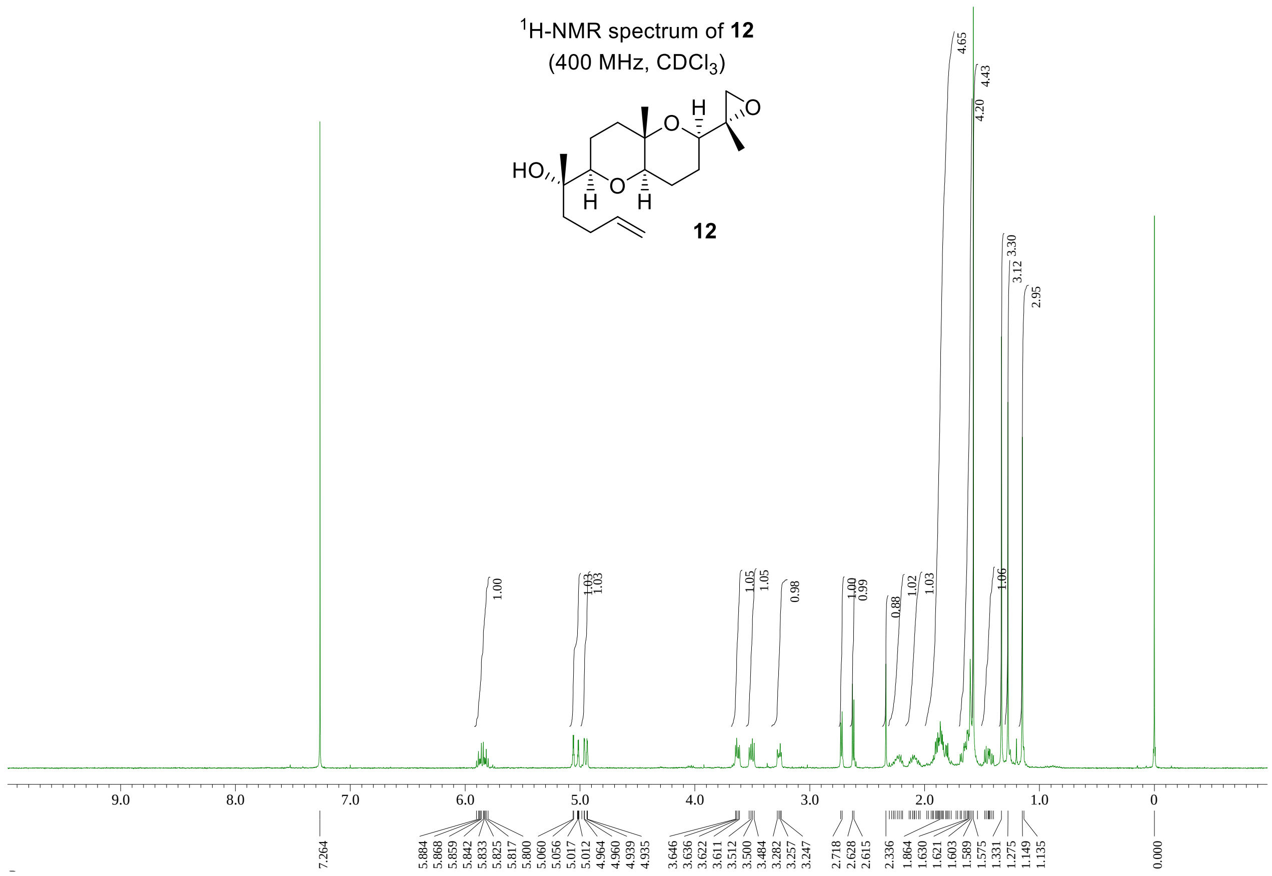

^{13}C-NMR spectrum of **12**
(100 MHz, $CDCl_3$)

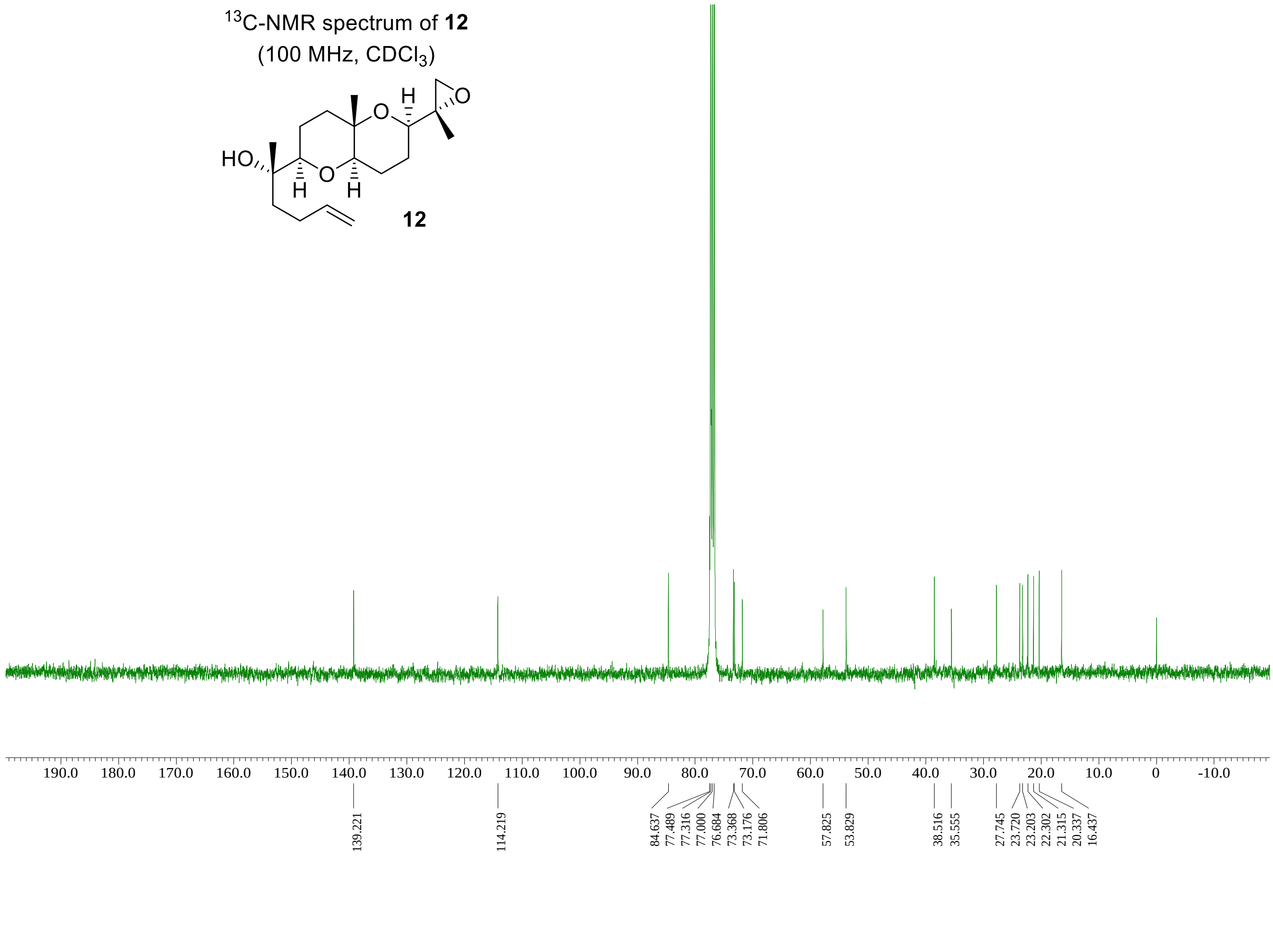

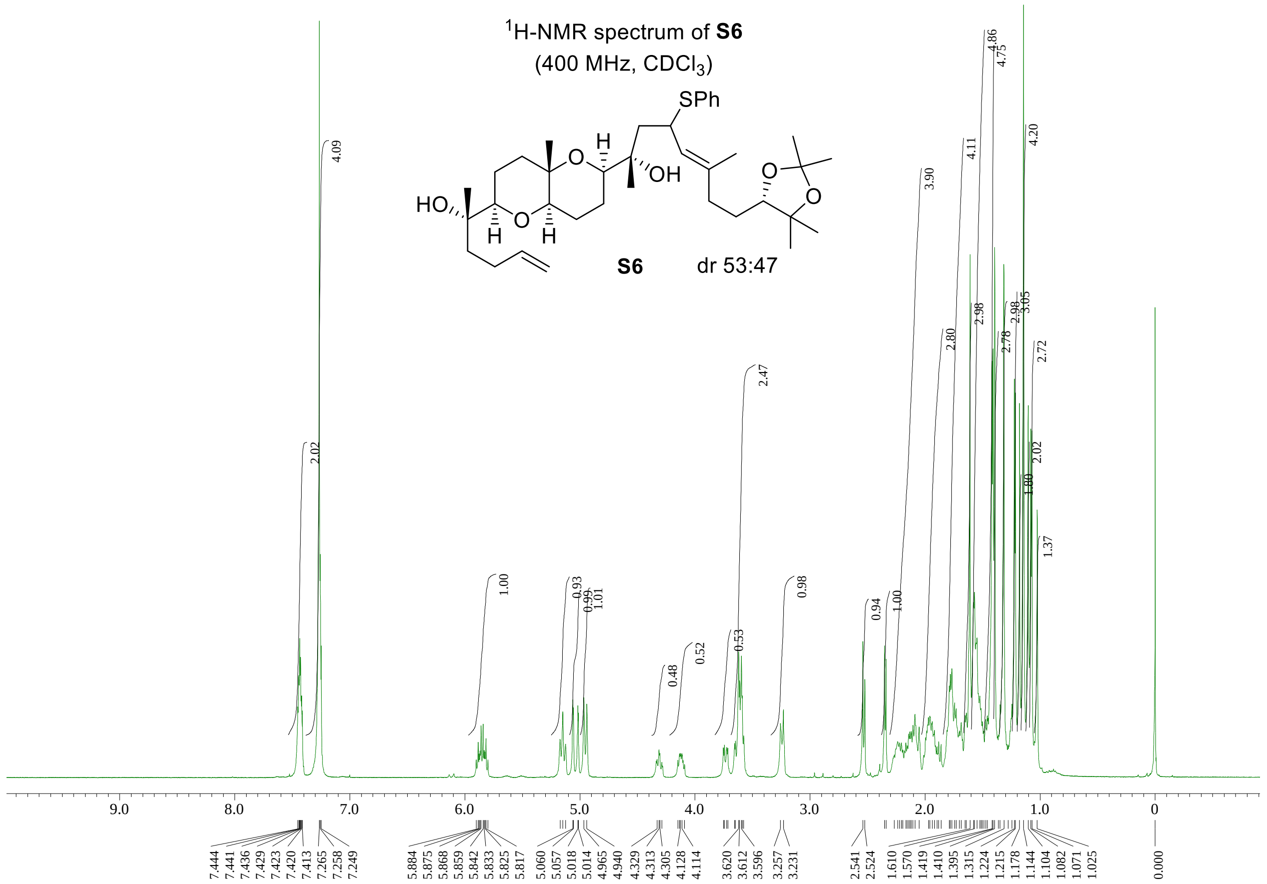
1H-NMR spectrum of S6
(400 MHz, CDCl3)
SPh
HO
OH
S6
dr 53:47
4.09
2.02
1.00
0.93
0.99
1.01
0.48
0.52
0.53
2.47
0.98
0.94
1.00
3.90
2.80
4.11
2.98
4.86
4.75
2.78
2.98
3.05
4.20
2.72
2.02
1.80
1.37
9.0
8.0
7.0
6.0
5.0
4.0
3.0
2.0
1.0
0
7.444
7.441
7.436
7.429
7.423
7.420
7.413
7.265
7.258
7.249
5.884
5.875
5.868
5.859
5.842
5.833
5.825
5.817
5.060
5.057
5.018
5.014
4.965
4.940
4.329
4.313
4.305
4.128
4.114
3.620
3.612
3.596
3.257
3.231
2.541
2.524
1.610
1.570
1.419
1.410
1.395
1.315
1.224
1.215
1.178
1.144
1.104
1.082
1.071
1.025
0.000

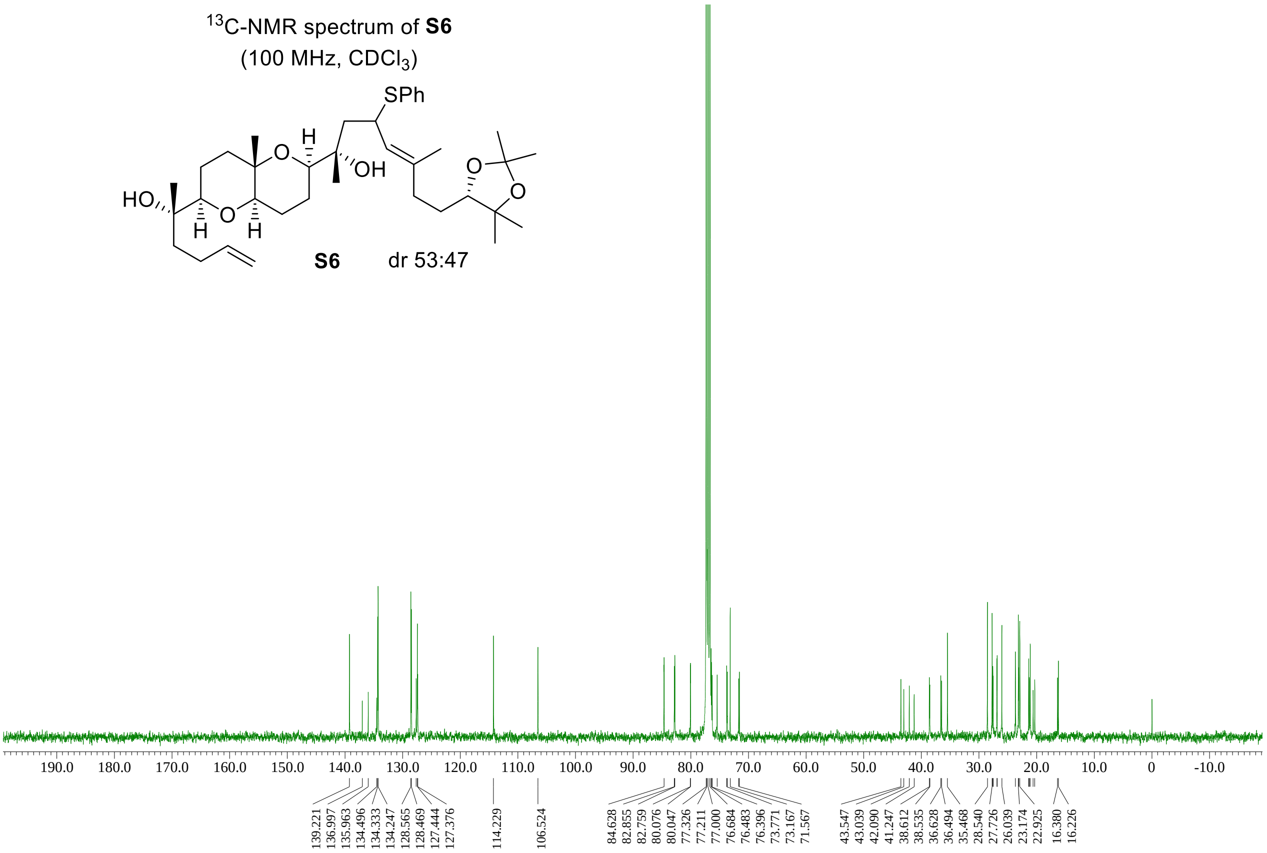
13C-NMR spectrum of S6
(100 MHz, CDCl3)
SPh
HO
OH
S6
dr 53:47
190.0
180.0
170.0
160.0
150.0
140.0
130.0
120.0
110.0
100.0
90.0
80.0
70.0
60.0
50.0
40.0
30.0
20.0
10.0
0
-10.0
139.221
136.997
135.963
134.496
134.333
134.247
128.565
128.469
127.444
127.376
114.229
106.524
84.628
82.855
82.759
80.076
80.047
77.326
77.211
77.000
76.684
76.483
76.396
73.771
73.167
71.567
43.547
43.039
42.090
41.247
38.612
38.535
36.628
36.494
35.468
28.540
27.726
26.039
23.174
22.925
16.380
16.226

^{1}H-NMR spectrum of **11**
(400 MHz, $CDCl_3$)

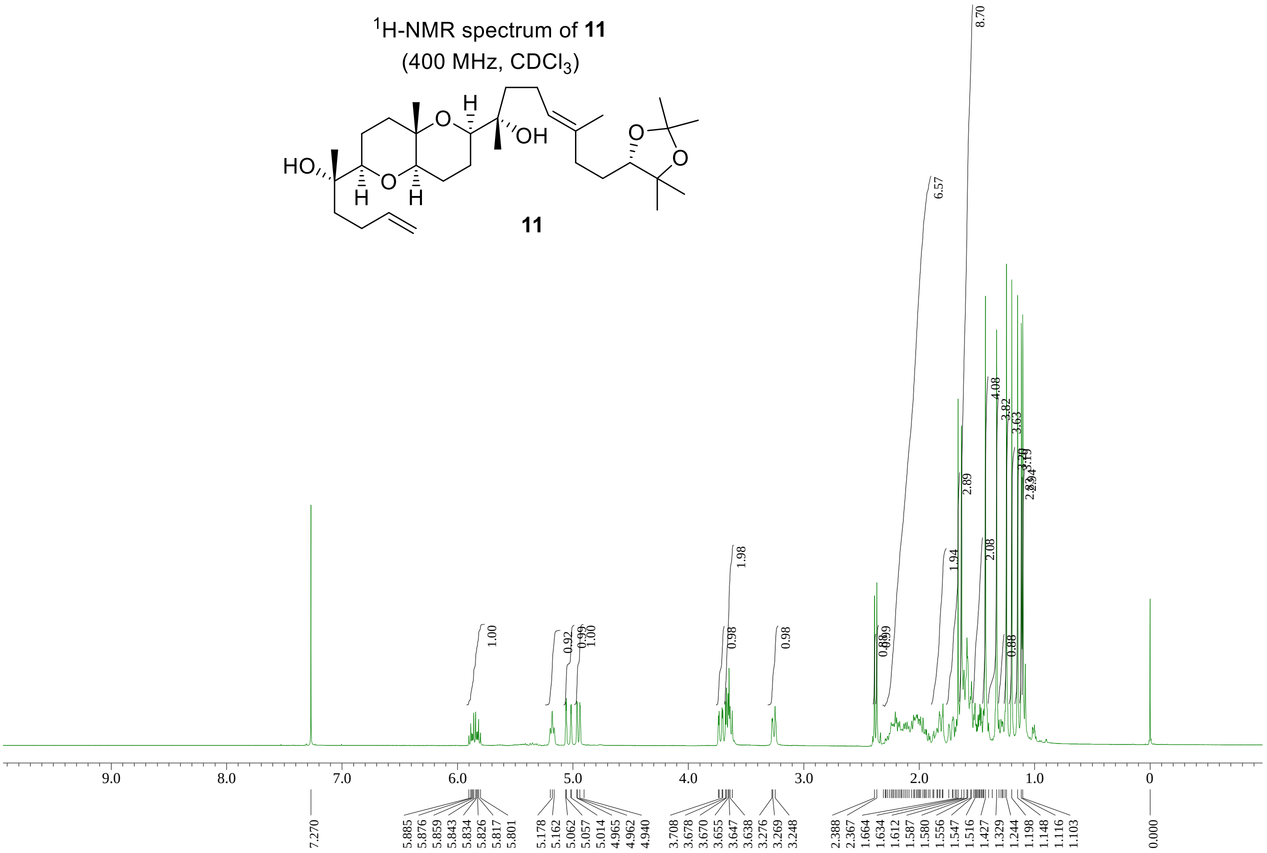

^{13}C-NMR spectrum of **11**
(100 MHz, $CDCl_3$)

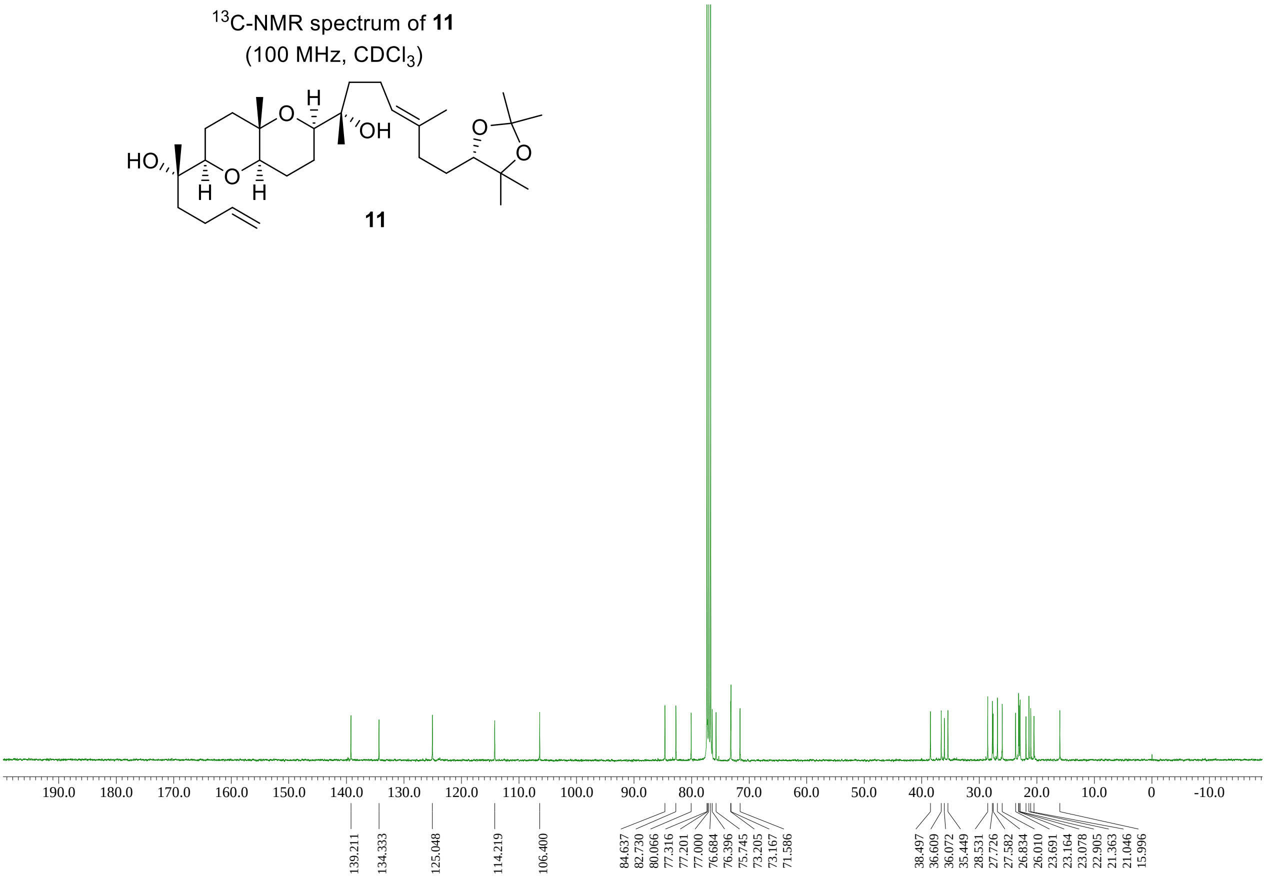

^{1}H-NMR spectrum of **21**
(400 MHz, $CDCl_3$)

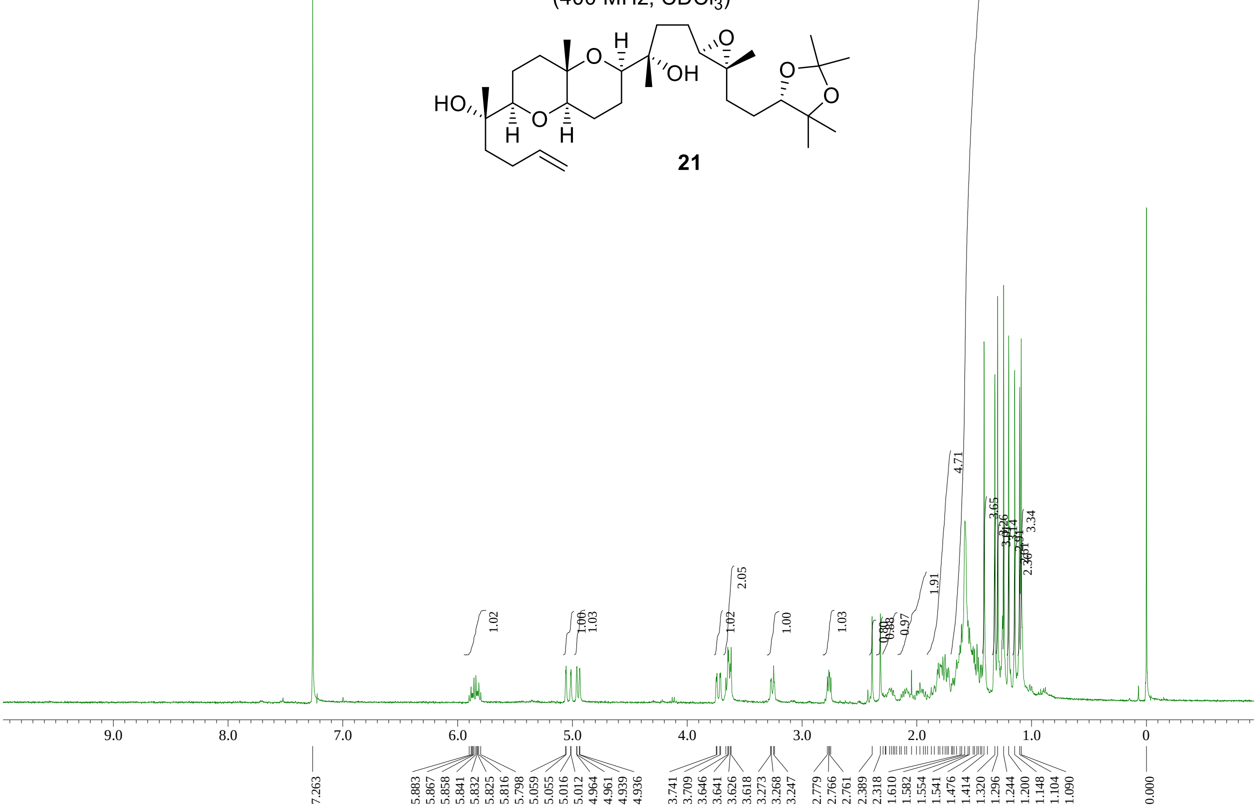

^{13}C-NMR spectrum of **21**
(100 MHz, $CDCl_3$)

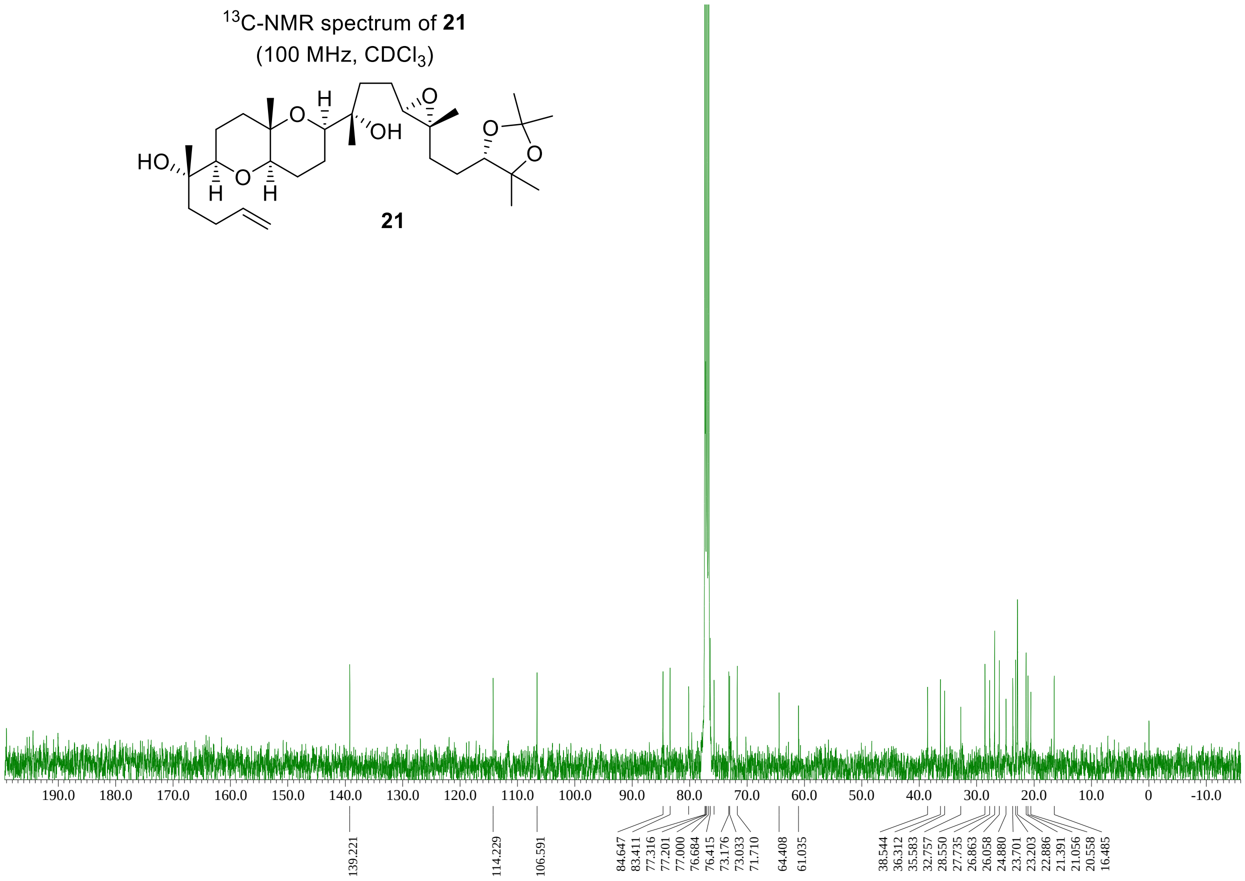

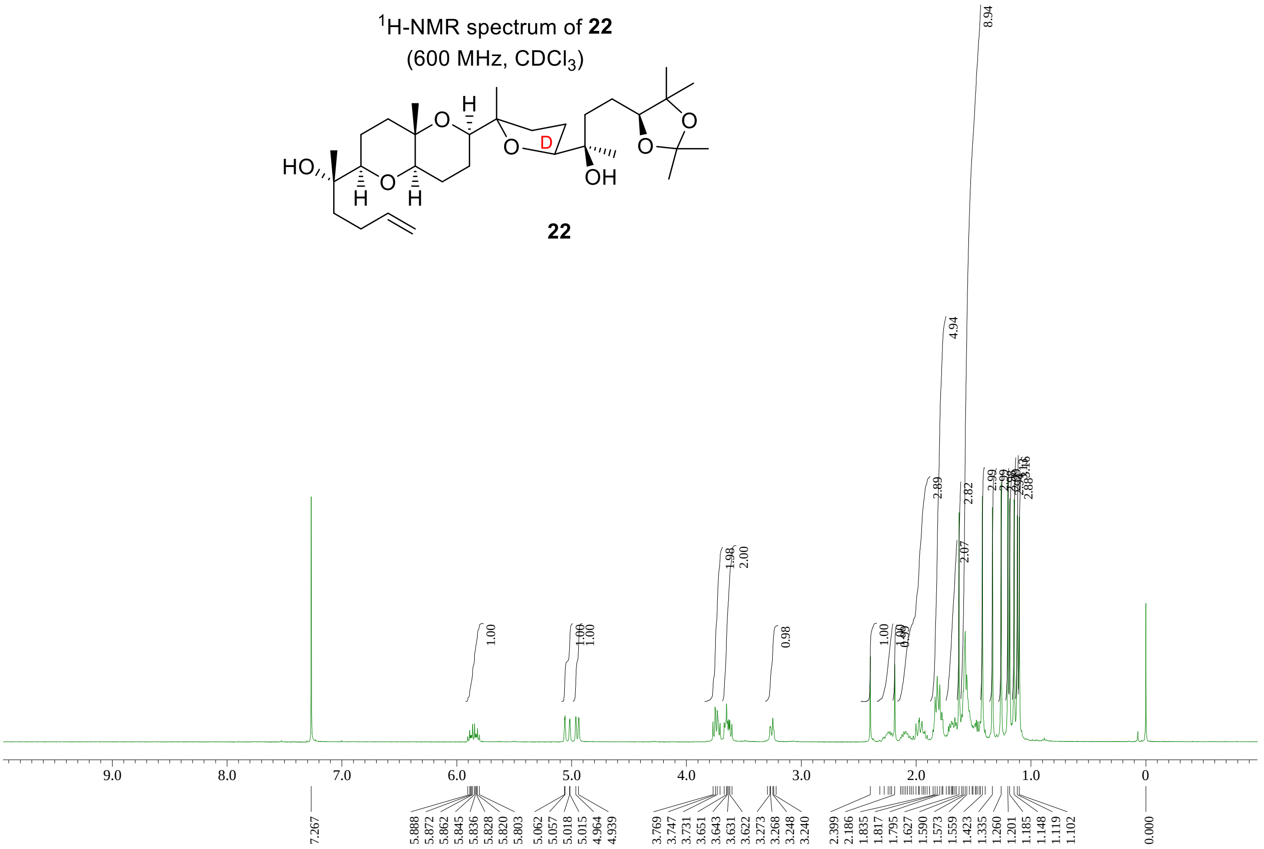

1H-NMR spectrum of 22
(600 MHz, CDCl3)
HO
OH
D
22
9.0
8.0
7.0
6.0
5.0
4.0
3.0
2.0
1.0
0
7.267
5.888 5.872 5.862 5.845 5.836 5.828 5.820 5.803
5.062 5.057 5.018 5.015 4.964 4.939
3.769 3.747 3.731 3.651 3.643 3.631 3.622
3.273 3.268 3.248 3.240
2.399 2.186 1.835 1.817 1.795 1.627 1.590 1.573 1.559 1.423 1.335 1.260 1.201 1.185 1.148 1.119 1.102
0.000
1.00
1.00
1.00
1.98
2.00
0.98
1.00
2.89
2.82
2.07
4.94
8.94

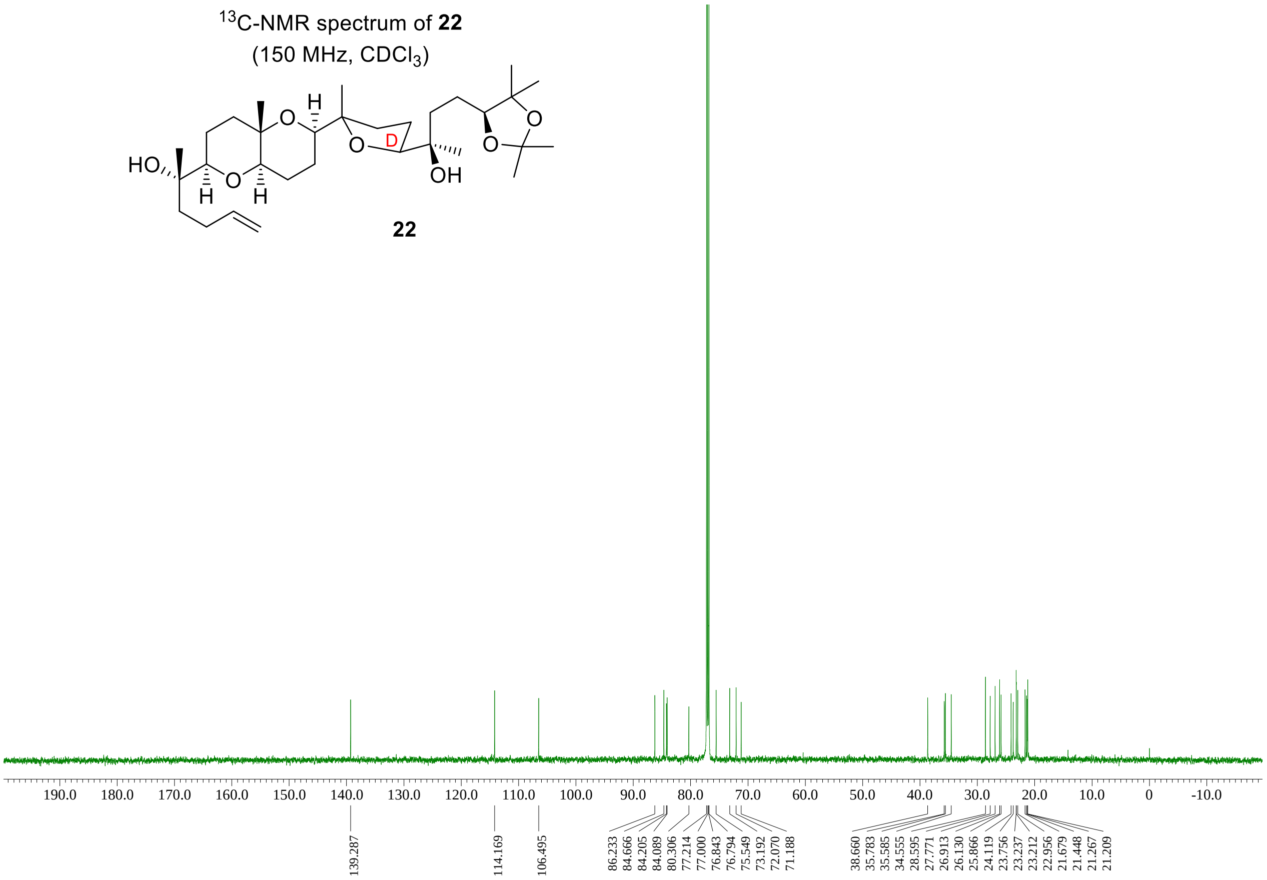

13C-NMR spectrum of 22
(150 MHz, CDCl3)
HO
OH
D
22
190.0
180.0
170.0
160.0
150.0
140.0
130.0
120.0
110.0
100.0
90.0
80.0
70.0
60.0
50.0
40.0
30.0
20.0
10.0
0
-10.0
139.287
114.169
106.495
86.233 84.666 84.205 84.089 80.306 77.214 77.000 76.843 76.794 75.549 73.192 72.070 71.188
38.660 35.783 35.585 34.555 28.595 27.771 26.913 26.130 25.866 24.119 23.756 23.237 23.212 22.956 21.679 21.448 21.267 21.209

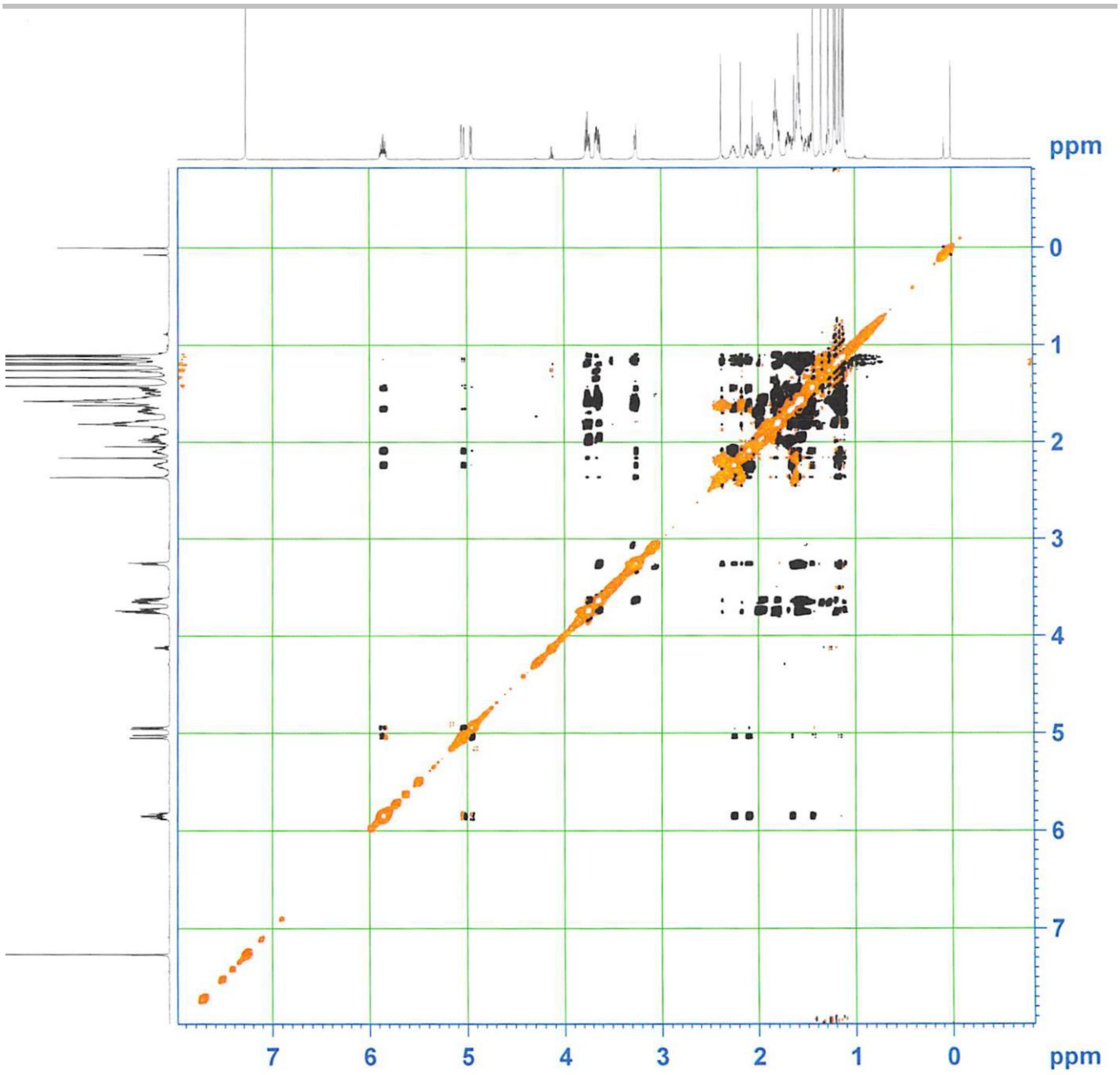

NOESY spectrum of **22**
(25 °C)
($CDCl_3$)

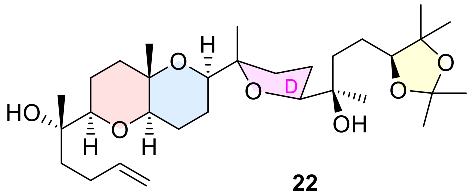

22

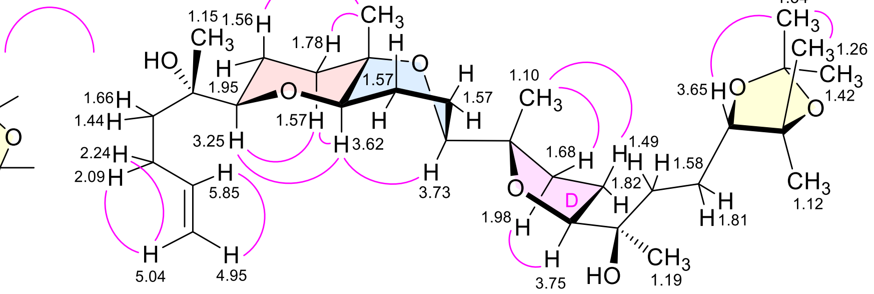

^{1}H-NMR spectrum of **10**
(400 MHz, $CDCl_3$)

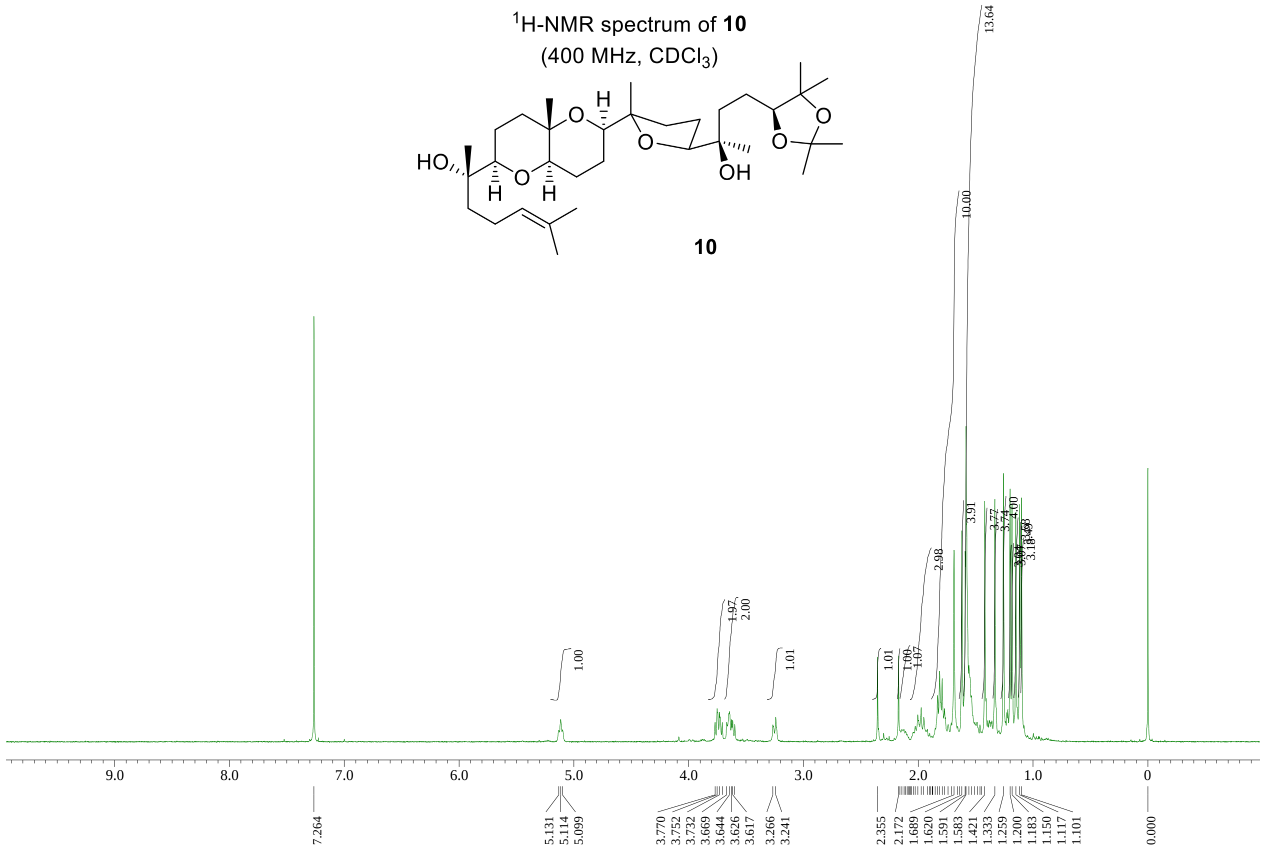

^{13}C-NMR spectrum of **10**
(100 MHz, $CDCl_3$)

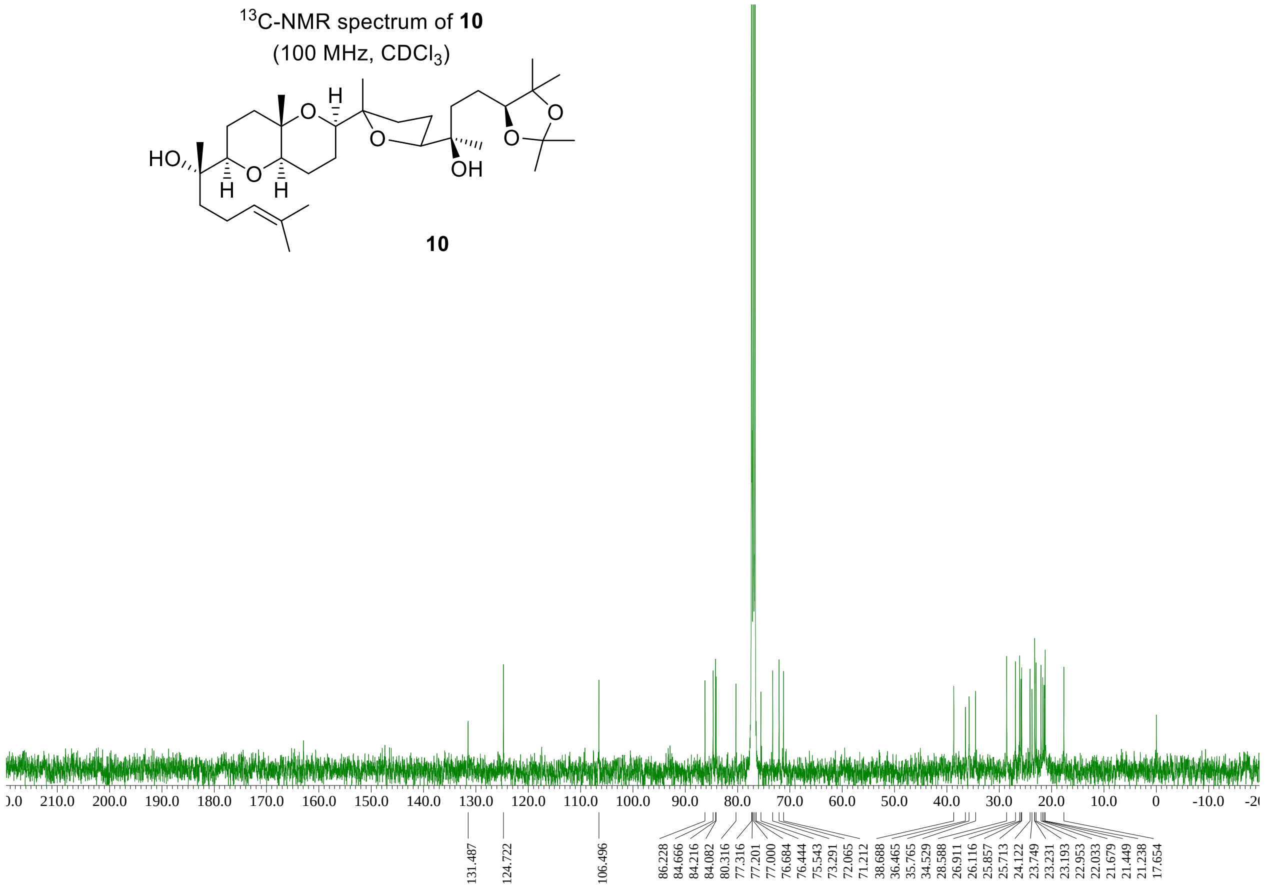

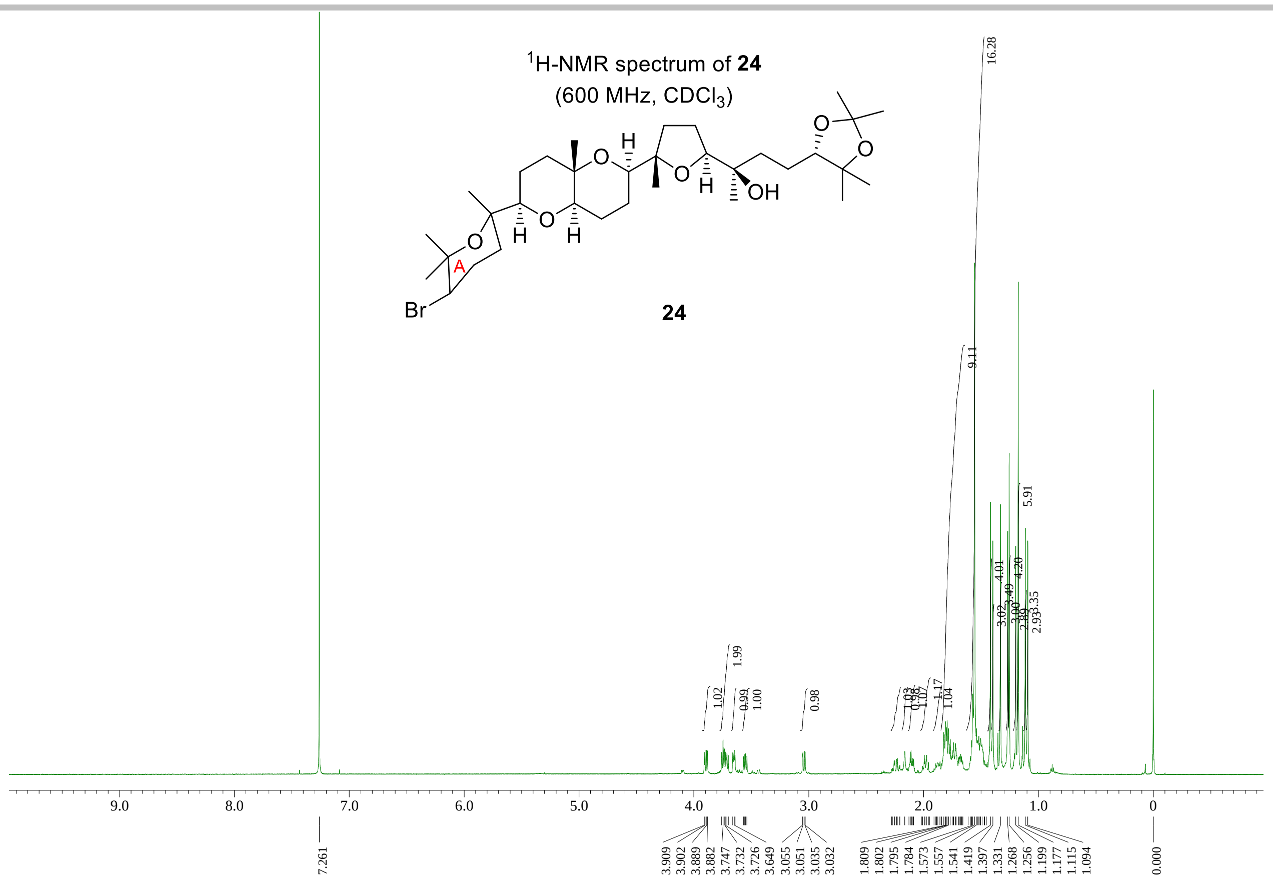
^{1}H-NMR spectrum of **24**
(600 MHz, CDCl$_3$)
24
Br
OH
9.0 8.0 7.0 6.0 5.0 4.0 3.0 2.0 1.0 0
7.261
3.909 3.902 3.889 3.882 3.747 3.732 3.726 3.649 3.055 3.051 3.035 3.032
1.809 1.802 1.795 1.784 1.573 1.557 1.541 1.419 1.397 1.331 1.268 1.256 1.199 1.177 1.115 1.094
0.000
16.28 9.11 5.91 1.02 1.99 0.99 1.00 0.98

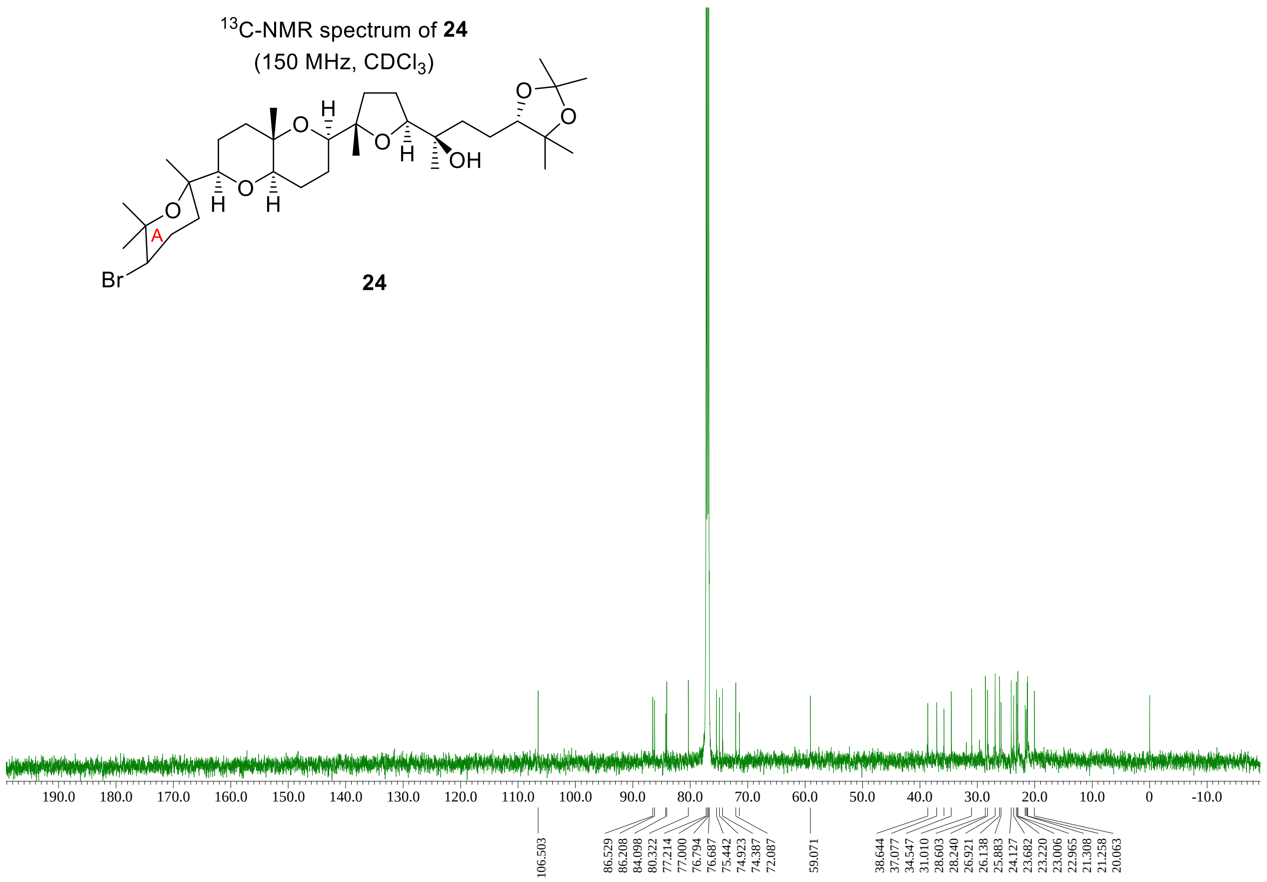
^{13}C-NMR spectrum of **24**
(150 MHz, CDCl$_3$)
24
Br
OH
190.0 180.0 170.0 160.0 150.0 140.0 130.0 120.0 110.0 100.0 90.0 80.0 70.0 60.0 50.0 40.0 30.0 20.0 10.0 0 -10.0
106.503
86.529 86.208 84.098 80.322 77.214 77.000 76.794 76.687 75.442 74.923 74.387 72.087
59.071
38.644 37.077 34.547 31.010 28.603 28.240 26.921 26.138 25.883 24.127 23.682 23.220 23.006 22.965 21.308 21.258 20.063

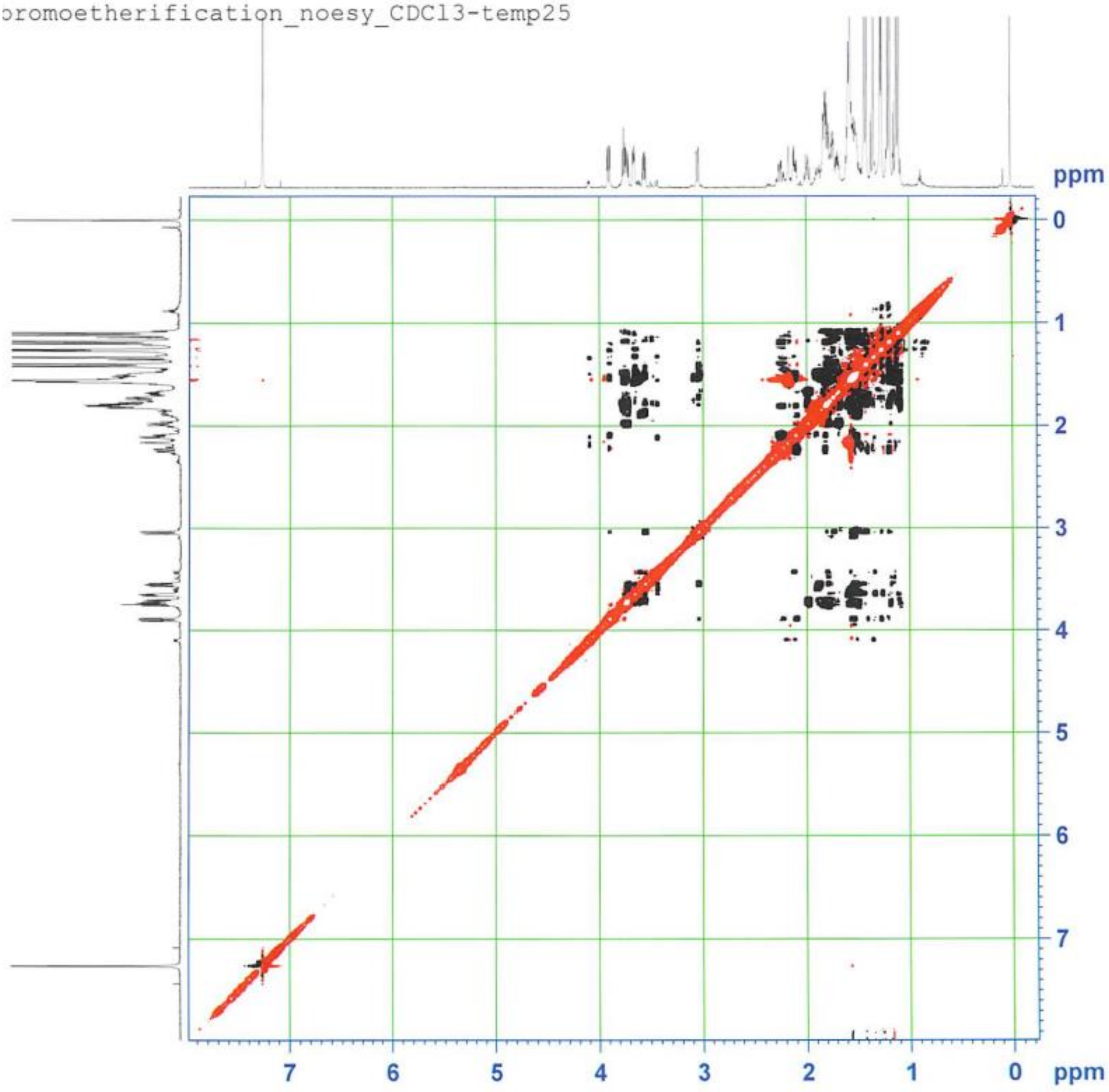

NOESY spectrum of **24**
(25 °C)
($CDCl_3$)

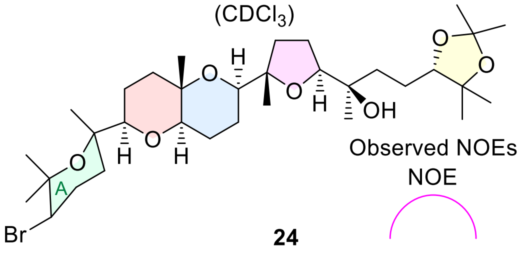

Observed NOEs
NOE

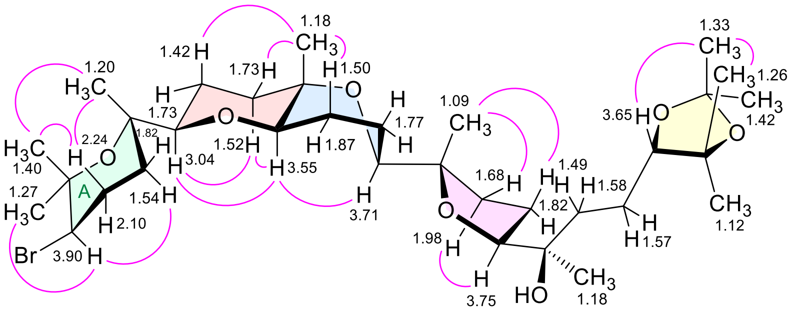

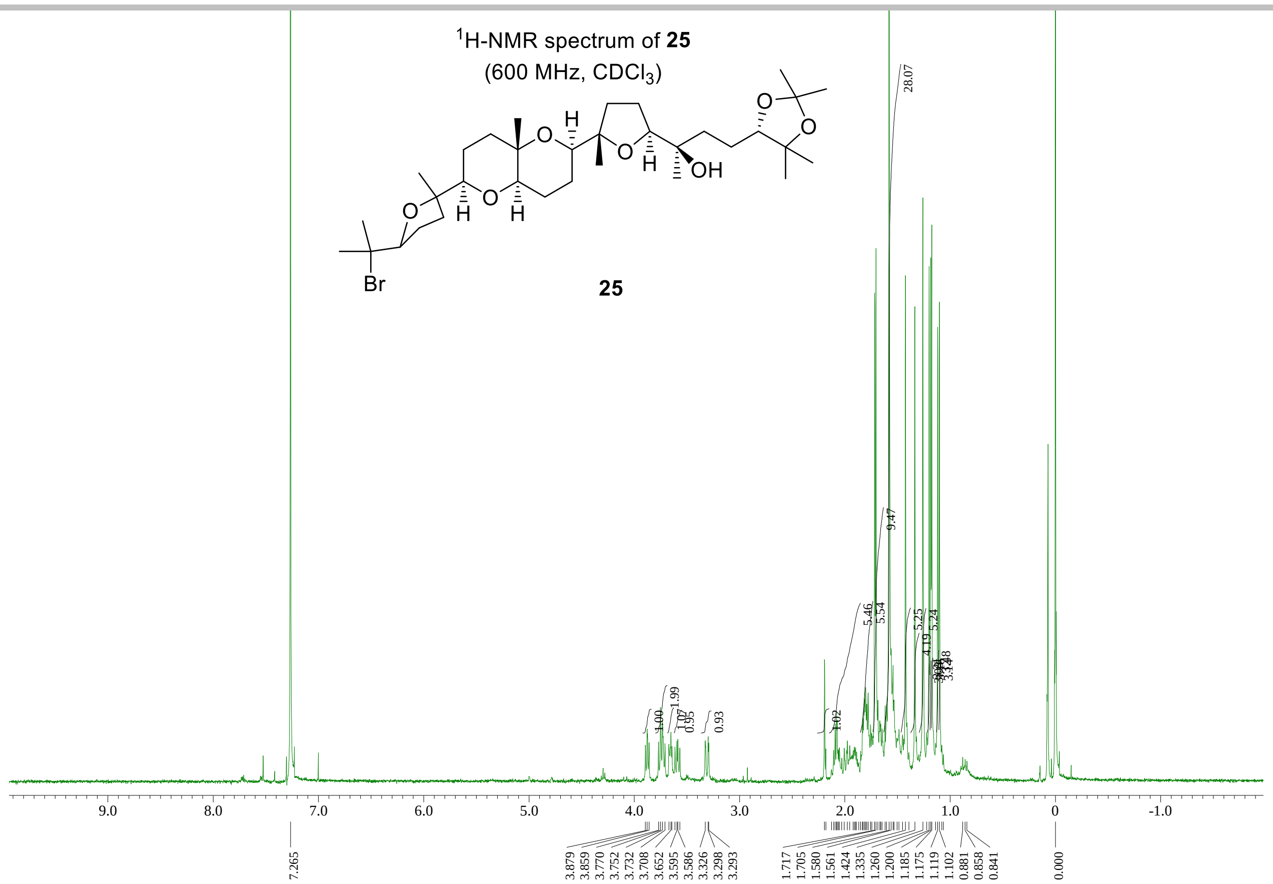

^{1}H-NMR spectrum of **25**
(600 MHz, $CDCl_3$)
25
9.0 8.0 7.0 6.0 5.0 4.0 3.0 2.0 1.0 0 -1.0
7.265
3.879 3.859 3.770 3.752 3.732 3.708 3.652 3.595 3.586 3.326 3.298 3.293
1.717 1.705 1.580 1.561 1.424 1.335 1.260 1.200 1.185 1.175 1.119 1.102 0.881 0.858 0.841
0.000

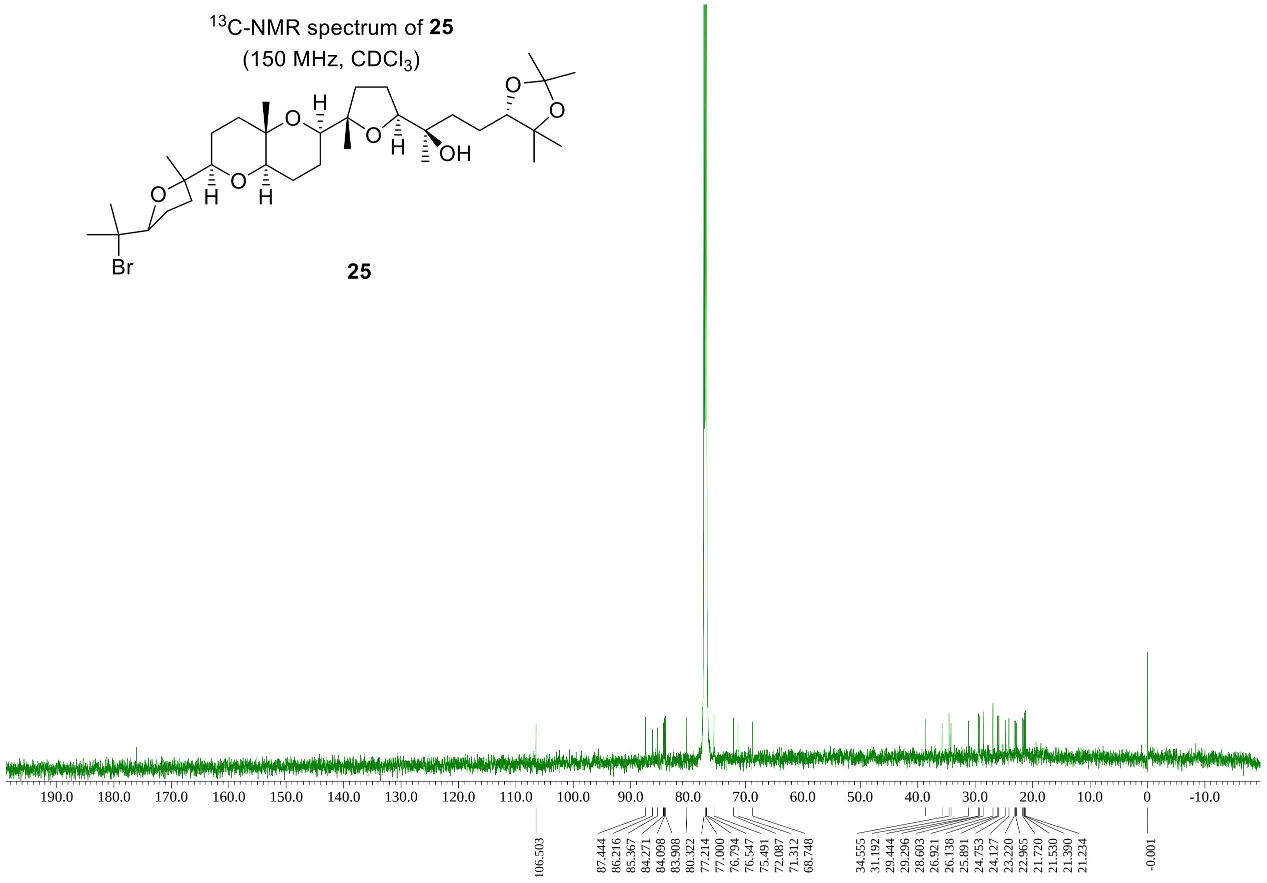

^{13}C-NMR spectrum of **25**
(150 MHz, $CDCl_3$)
25
190.0 180.0 170.0 160.0 150.0 140.0 130.0 120.0 110.0 100.0 90.0 80.0 70.0 60.0 50.0 40.0 30.0 20.0 10.0 0 -10.0
106.503
87.444 86.216 85.367 84.271 84.098 83.908 80.322 77.214 77.000 76.794 76.547 75.491 72.087 71.312 68.748
34.555 31.192 29.444 29.296 28.603 26.921 26.138 25.891 24.753 24.127 23.220 22.965 21.720 21.530 21.390 21.234
-0.001

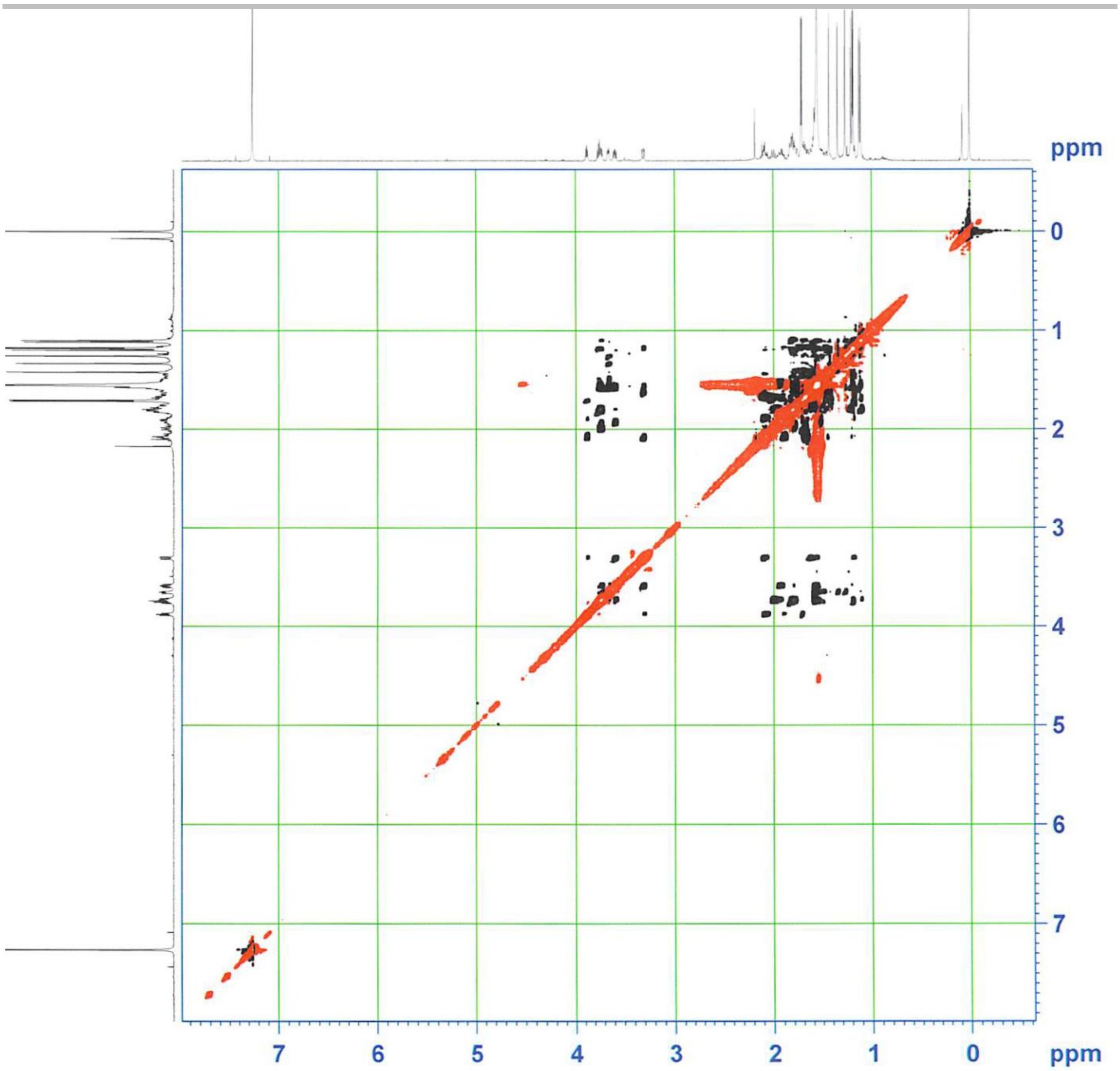

NOESY spectrum of **25**
(25 °C)
($CDCl_3$)

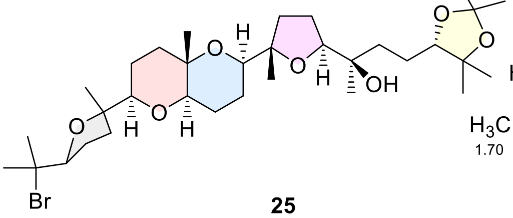

25

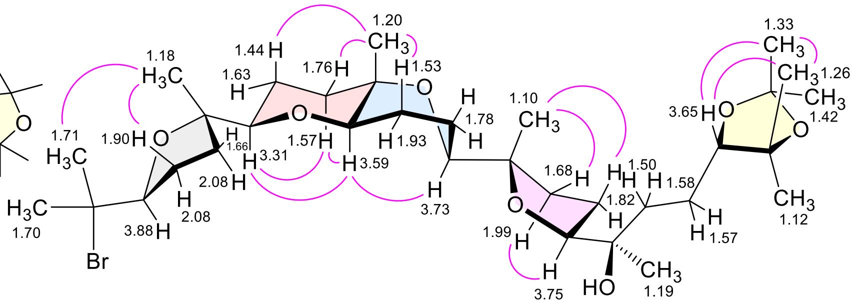

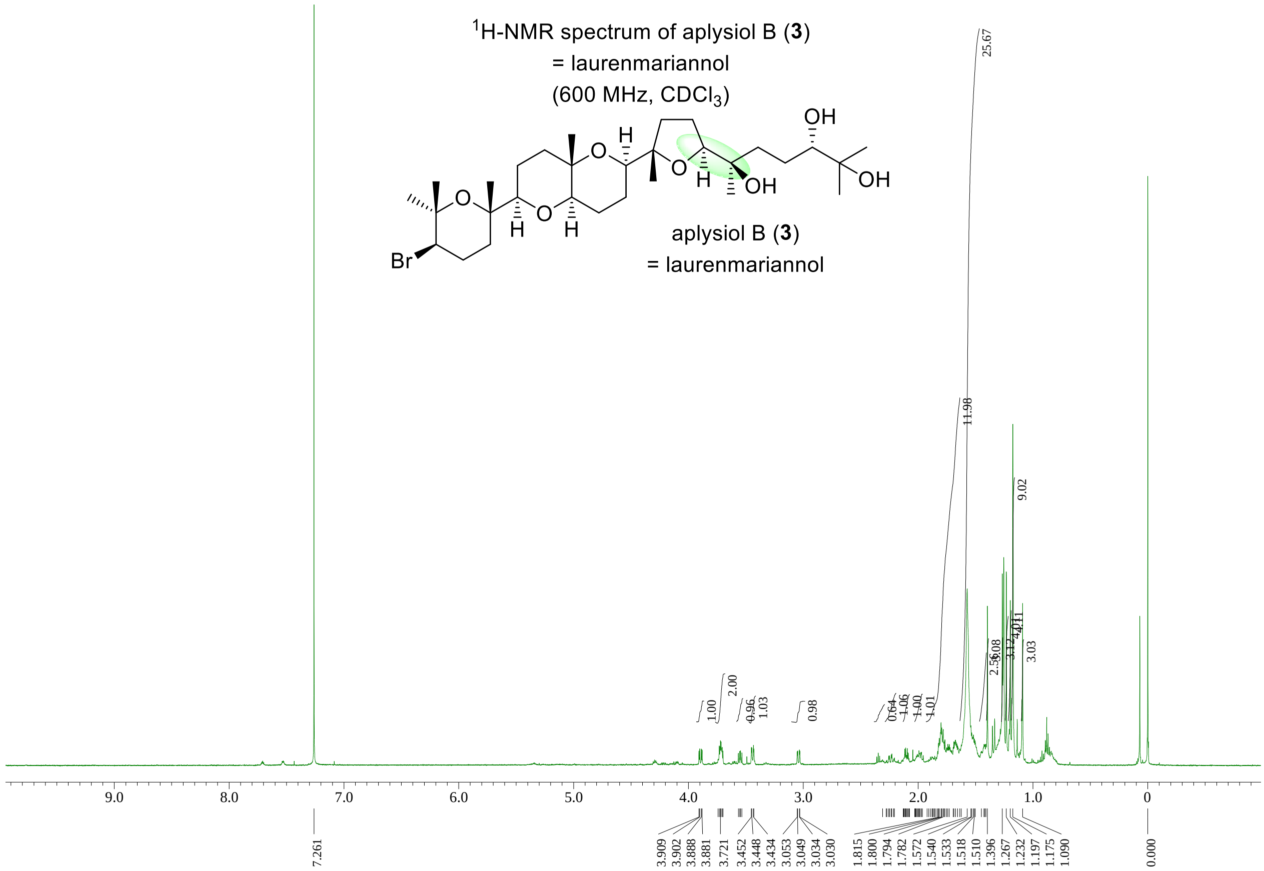
^{1}H-NMR spectrum of aplysiol B (**3**)
= laurenmariannol
(600 MHz, CDCl$_3$)
aplysiol B (**3**)
= laurenmariannol

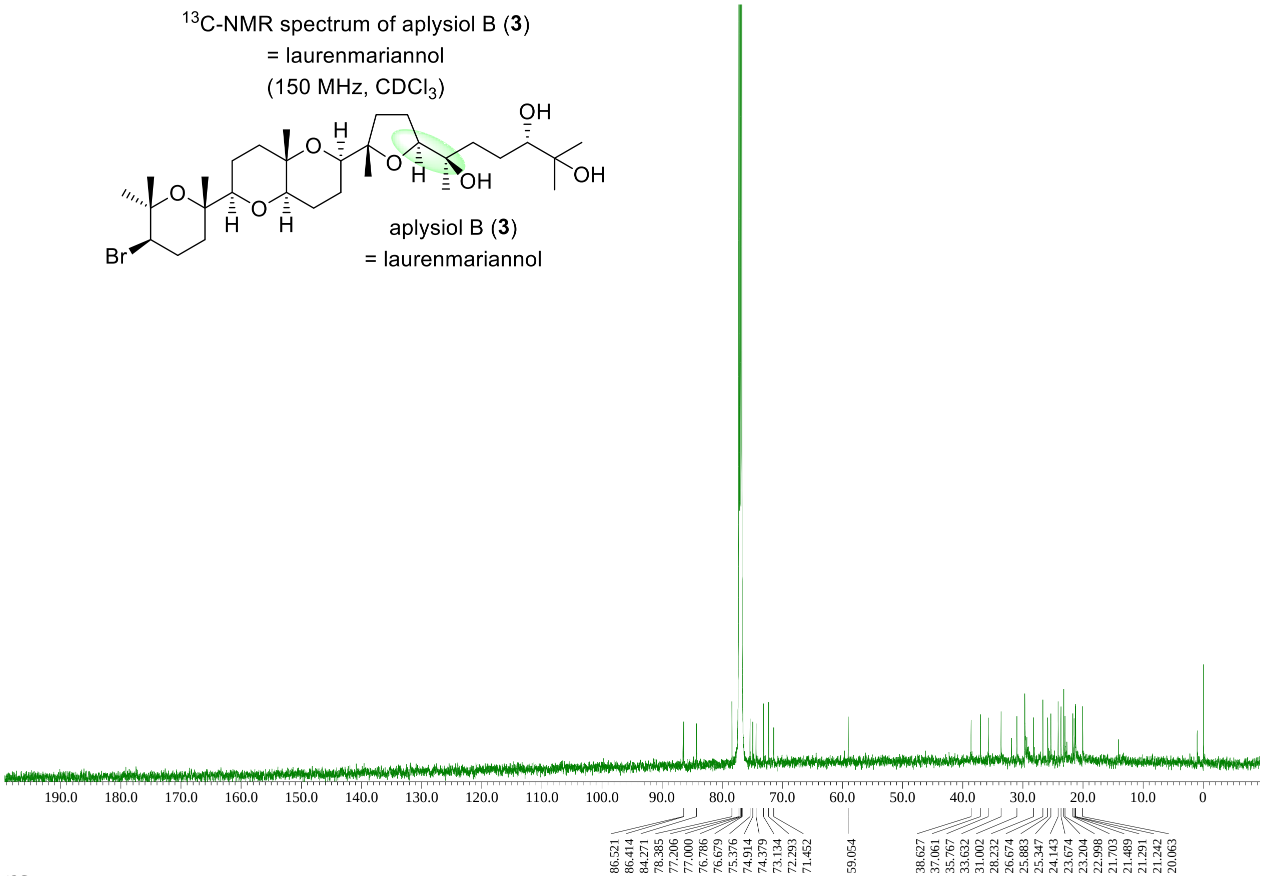
^{13}C-NMR spectrum of aplysiol B (**3**)
= laurenmariannol
(150 MHz, CDCl$_3$)
aplysiol B (**3**)
= laurenmariannol

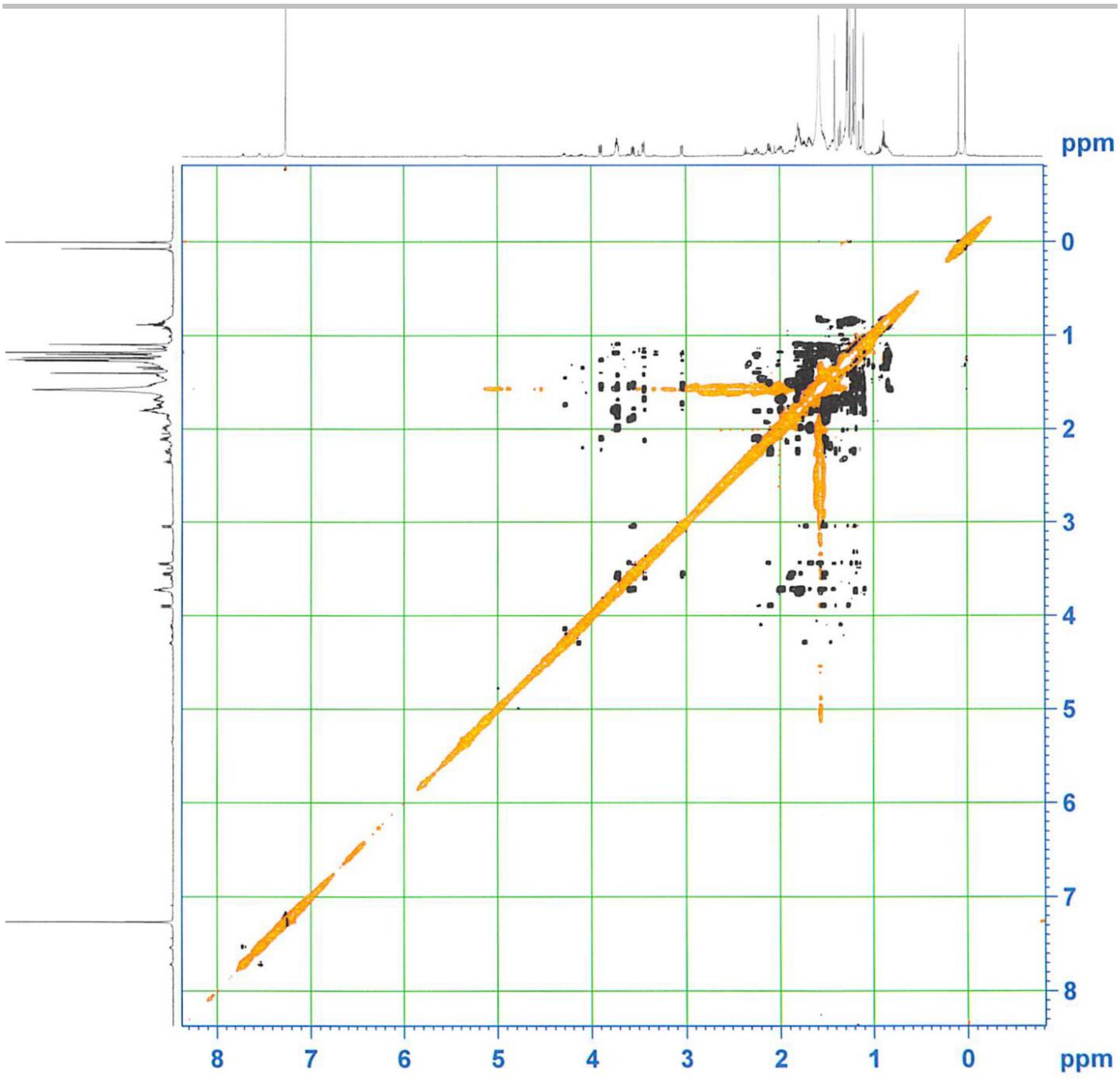

NOESY spectrum of aplysiol B (**3**) = laurenmariannol
(25 °C)
($CDCl_3$)

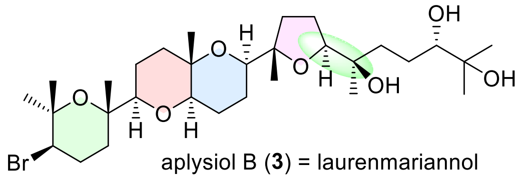

aplysiol B (**3**) = laurenmariannol

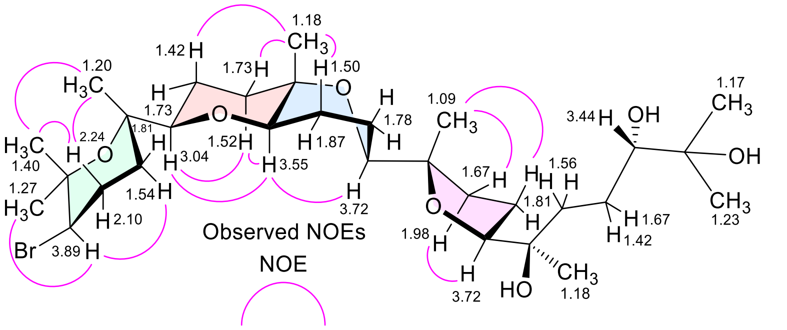

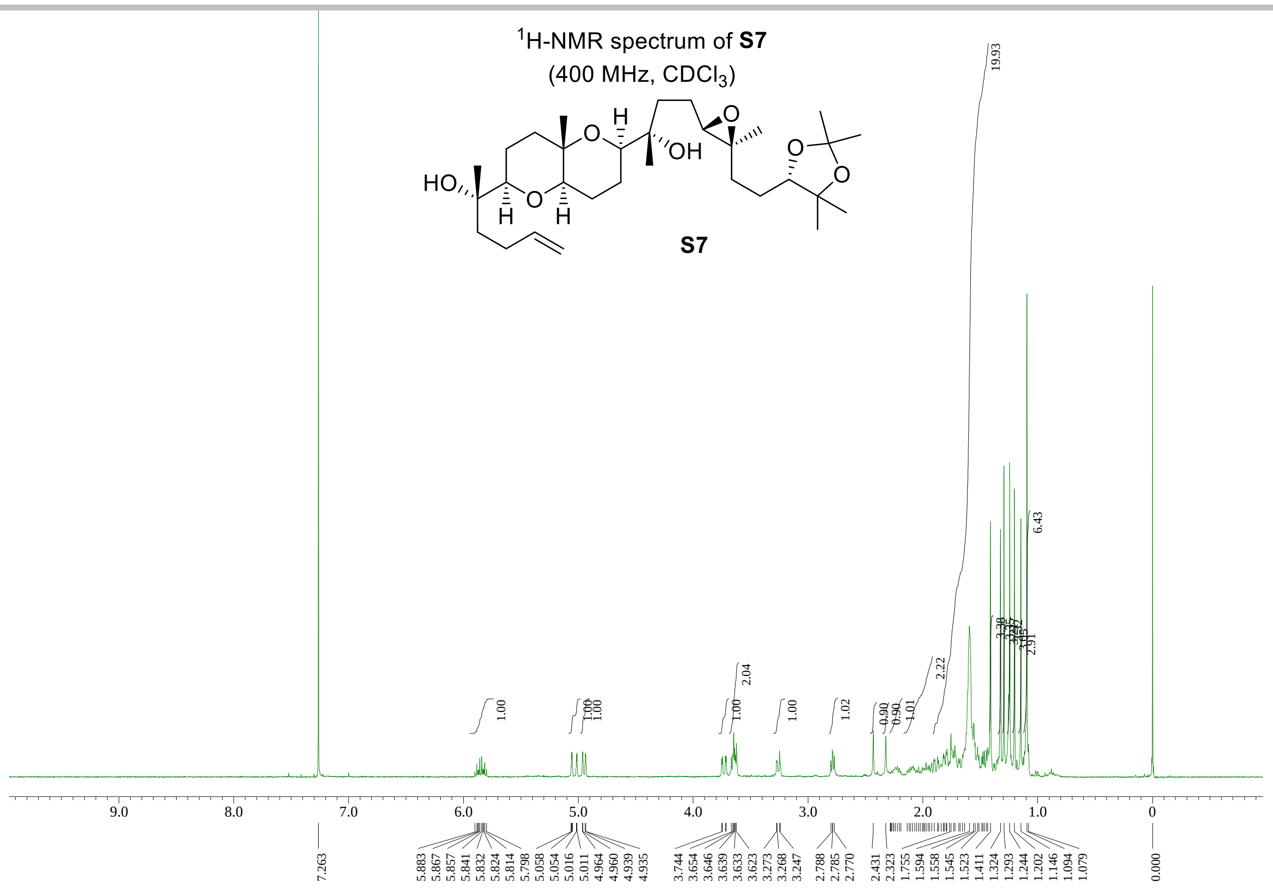

^{1}H-NMR spectrum of **S7** (400 MHz, $CDCl_3$)

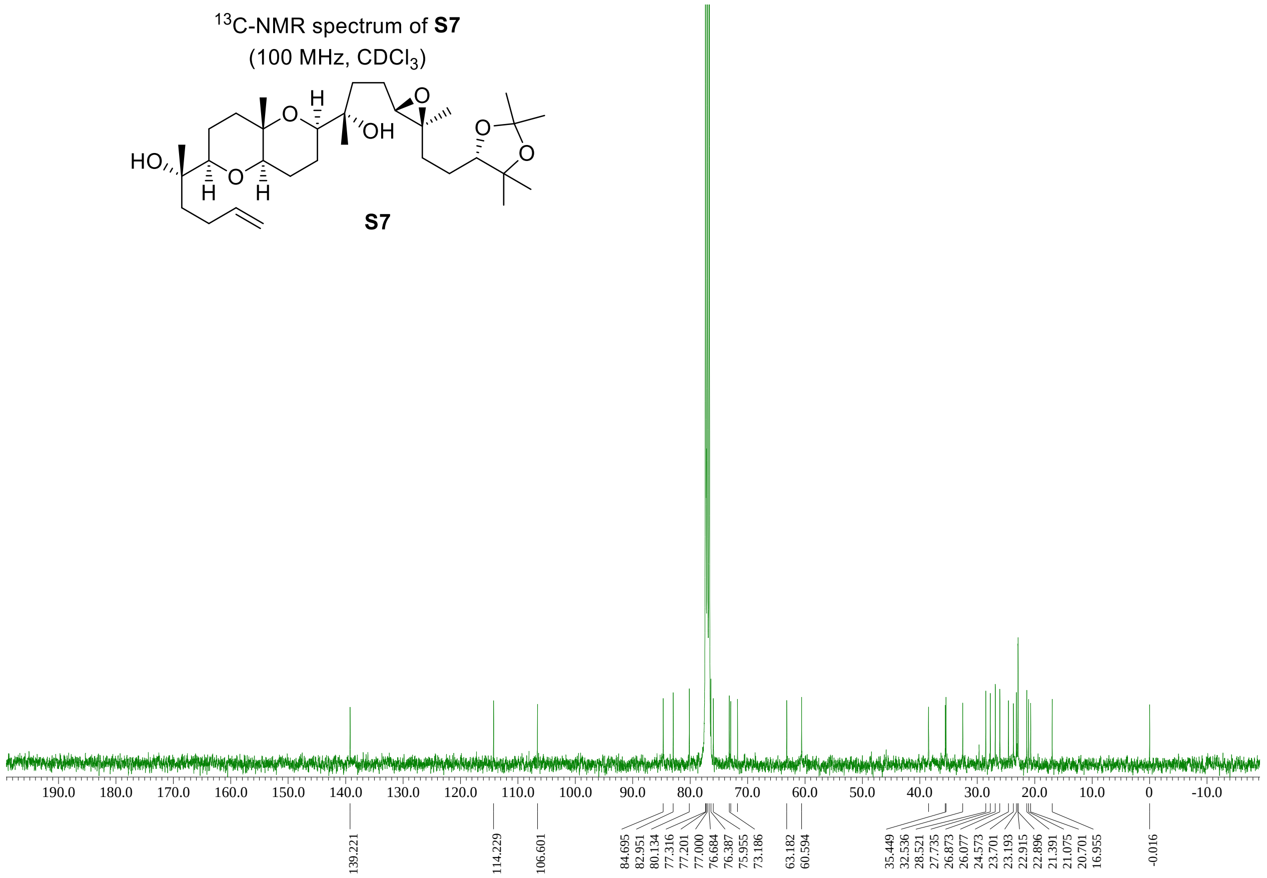

^{13}C-NMR spectrum of **S7** (100 MHz, $CDCl_3$)

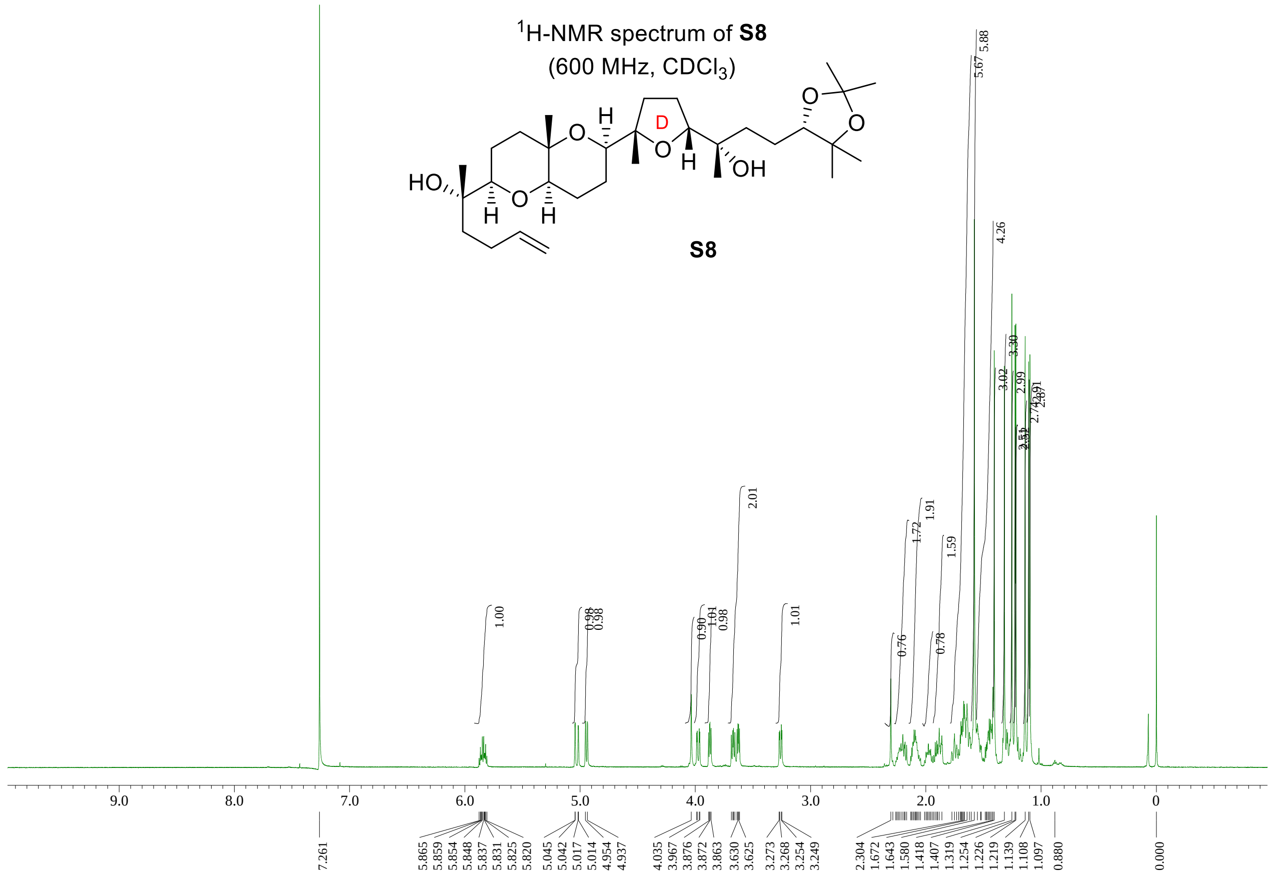

^{1}H-NMR spectrum of **S8**
(600 MHz, $CDCl_3$)

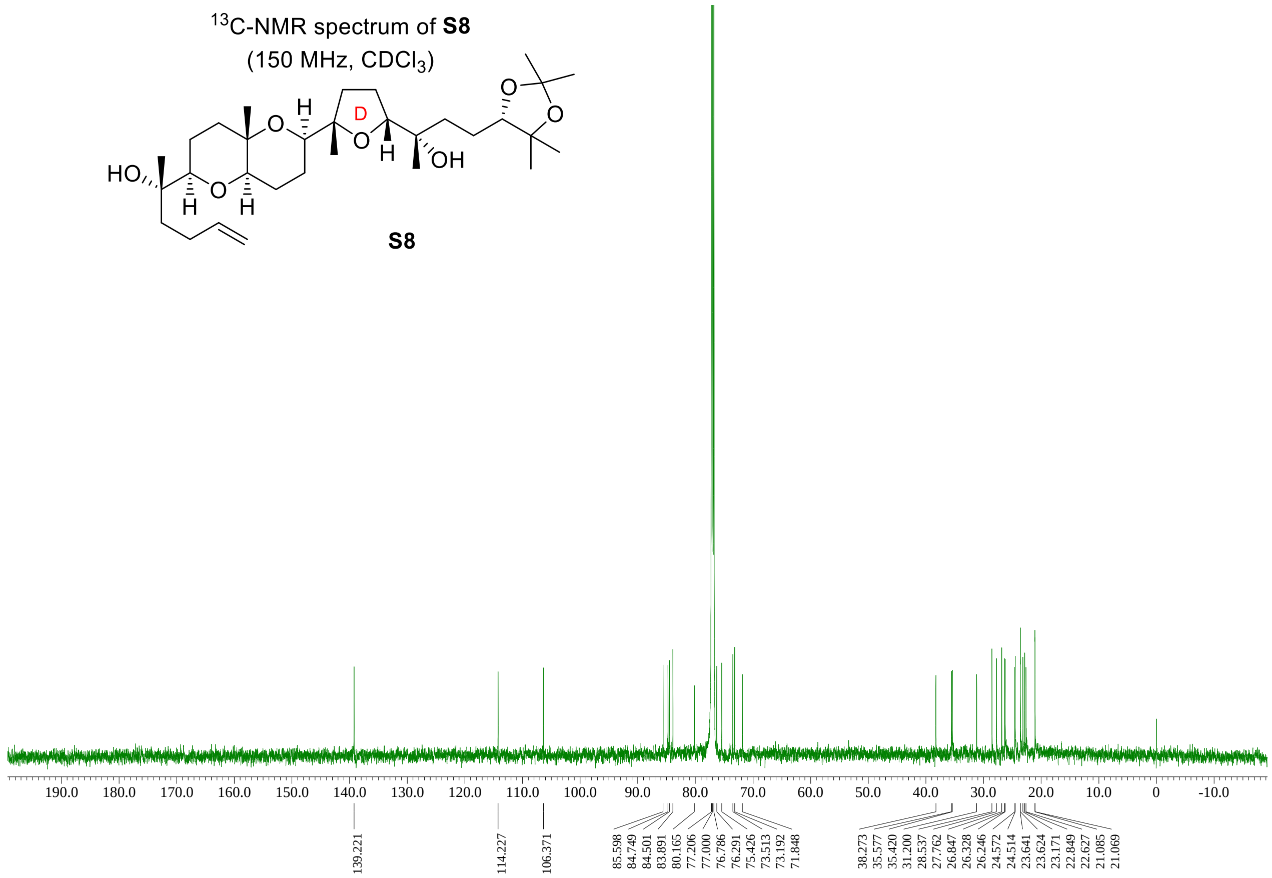

^{13}C-NMR spectrum of **S8**
(150 MHz, $CDCl_3$)

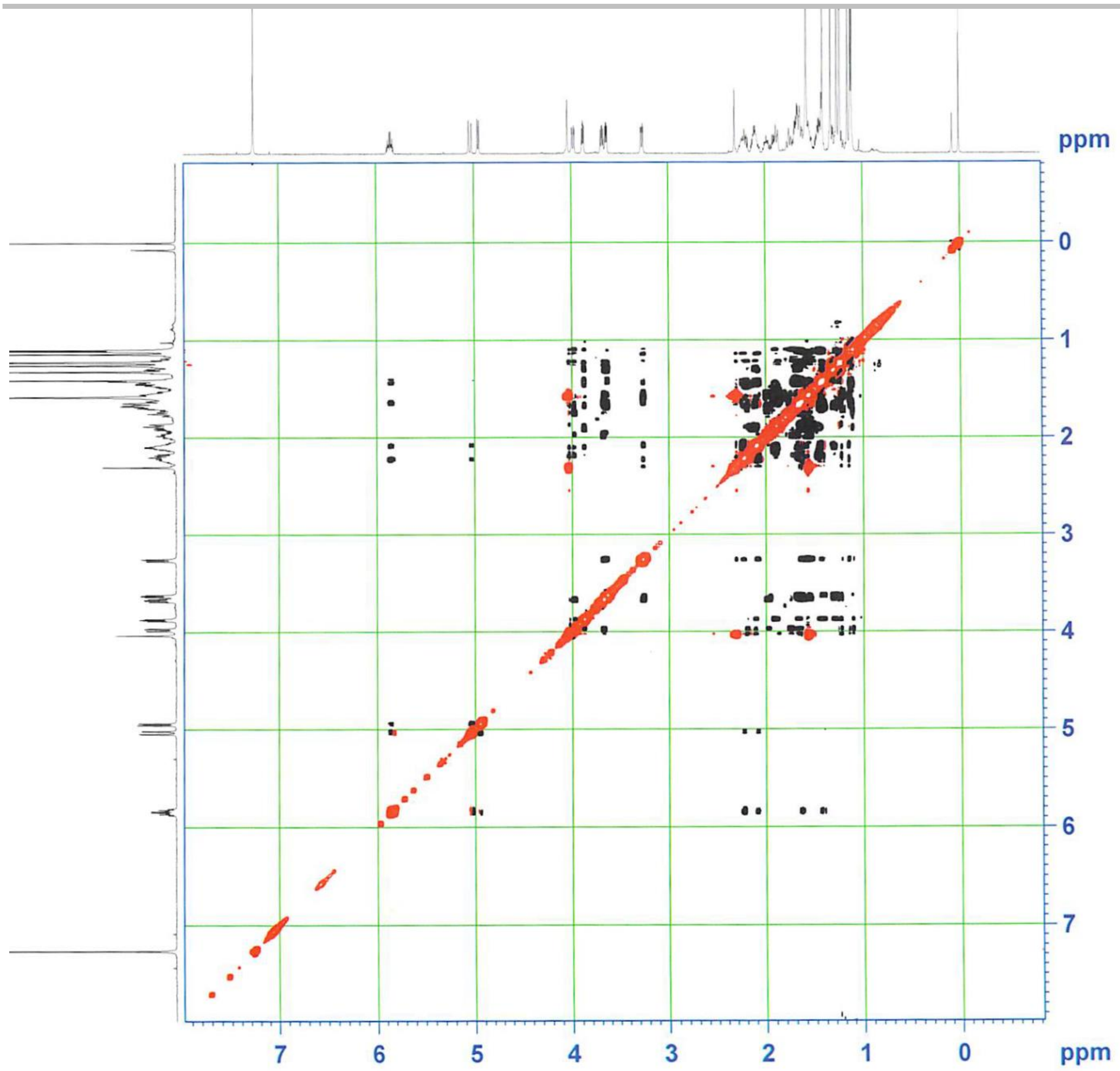

NOESY spectrum of **S8**
(25 °C)
($CDCl_3$)

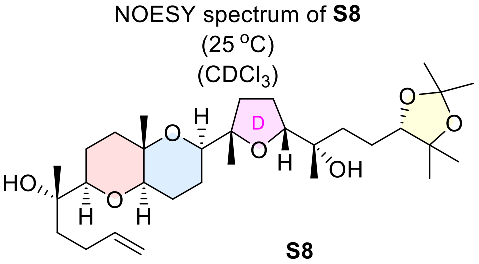

S8

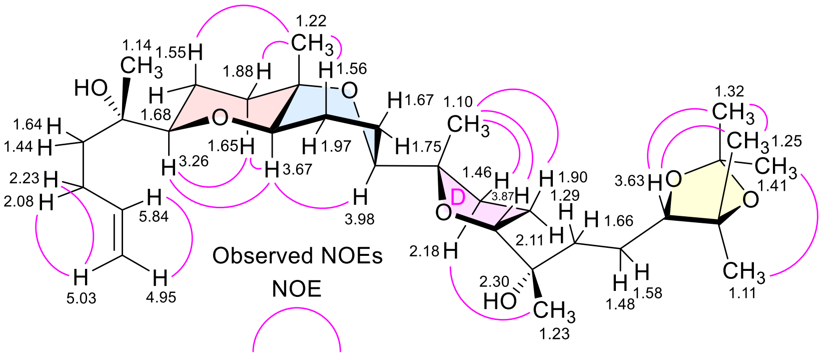

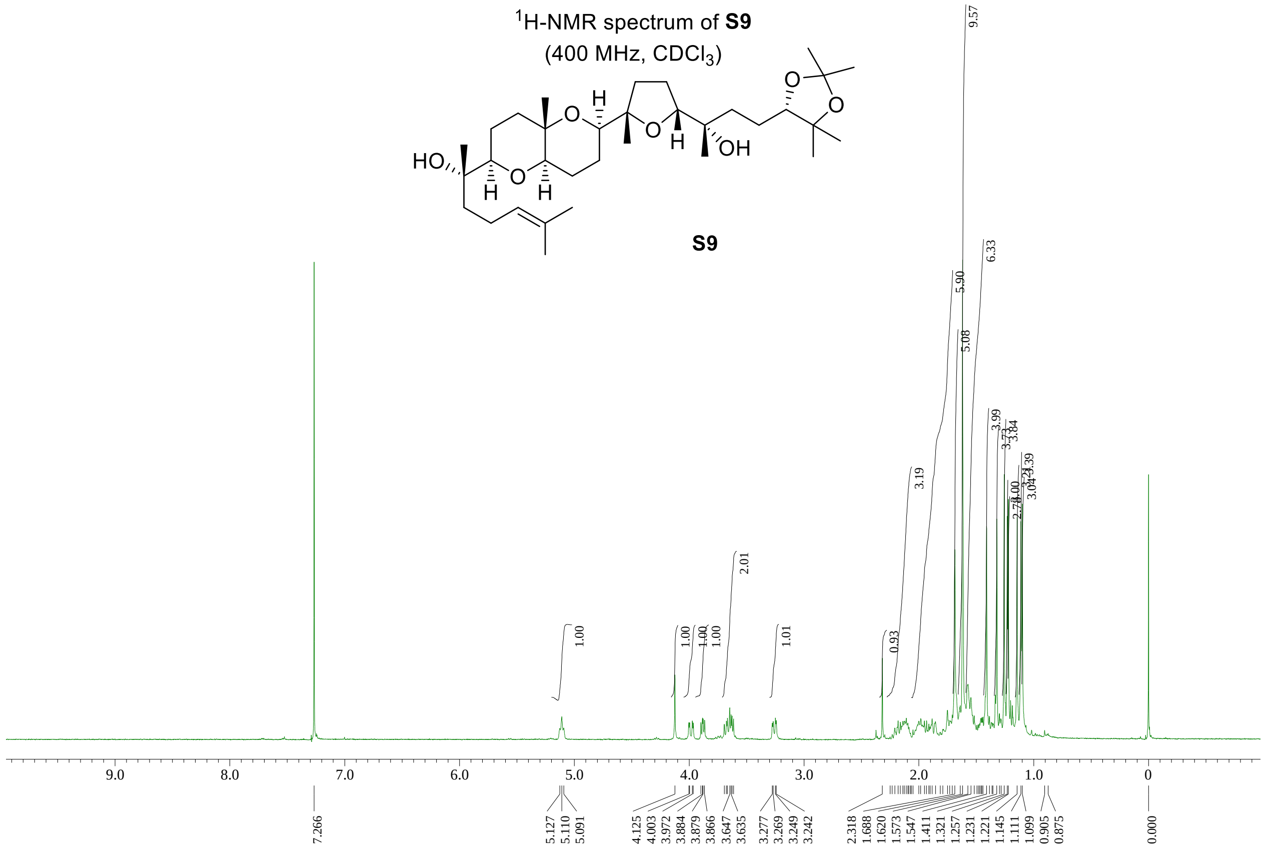
1H-NMR spectrum of S9
(400 MHz, CDCl3)
S9

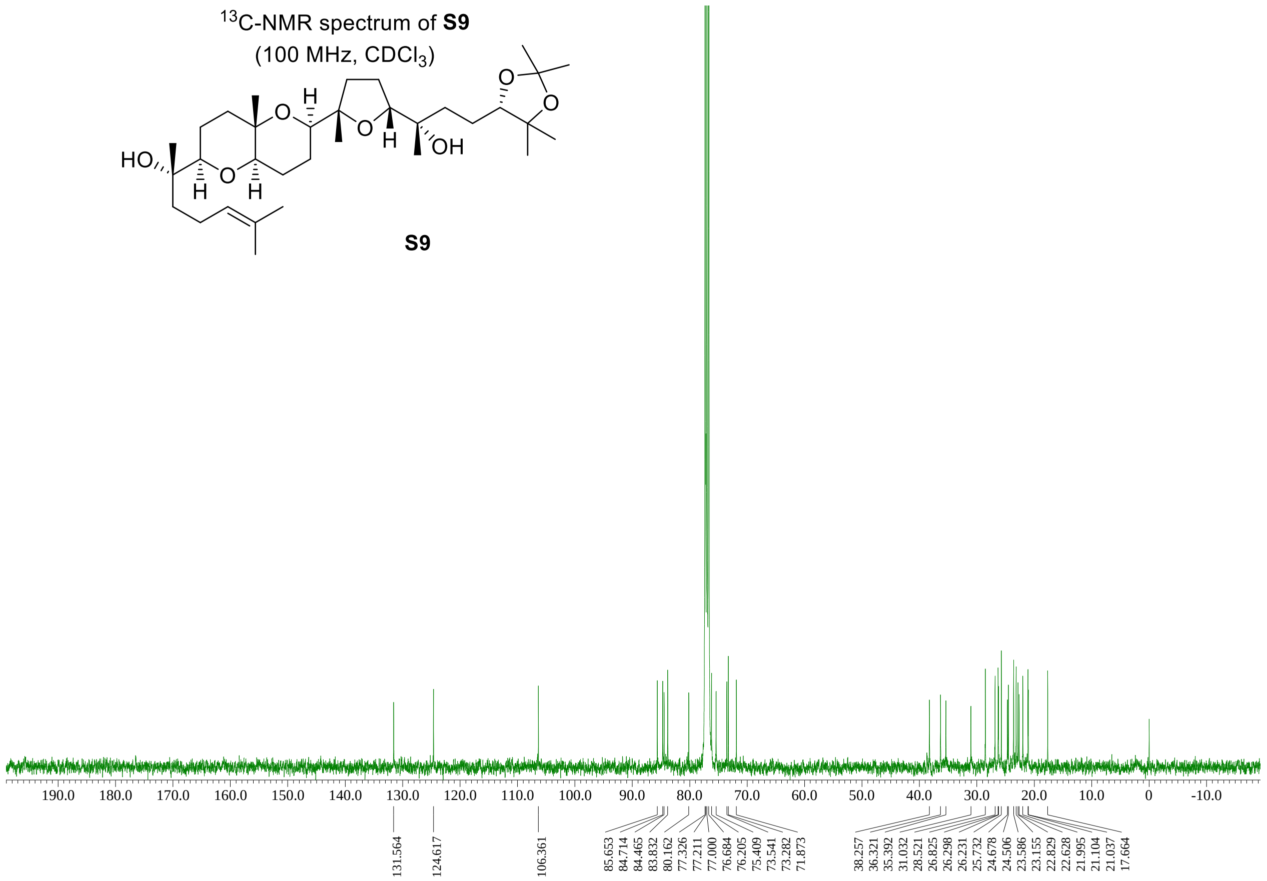
13C-NMR spectrum of S9
(100 MHz, CDCl3)
S9

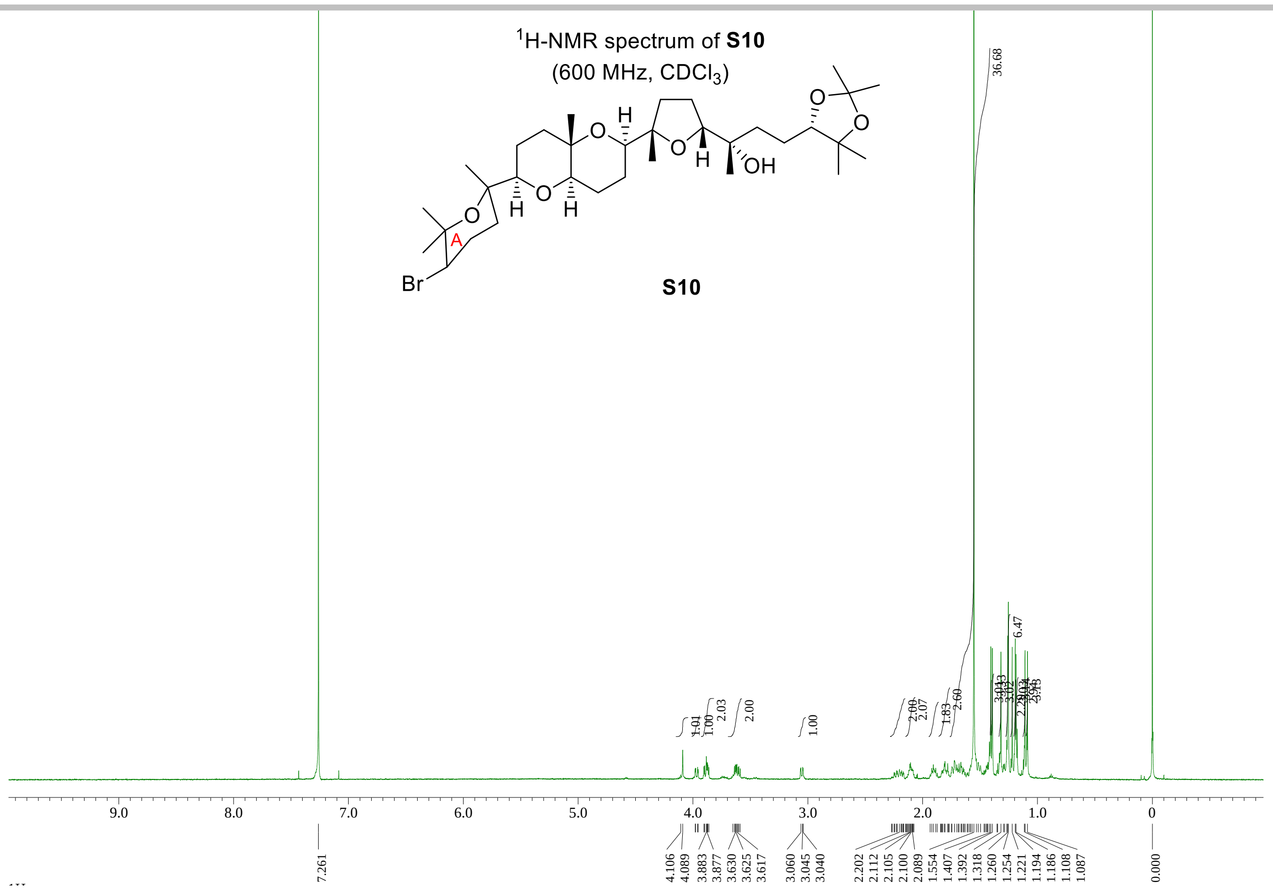

1H-NMR spectrum of **S10**
(600 MHz, CDCl3)
Br
A
O
H
OH
S10

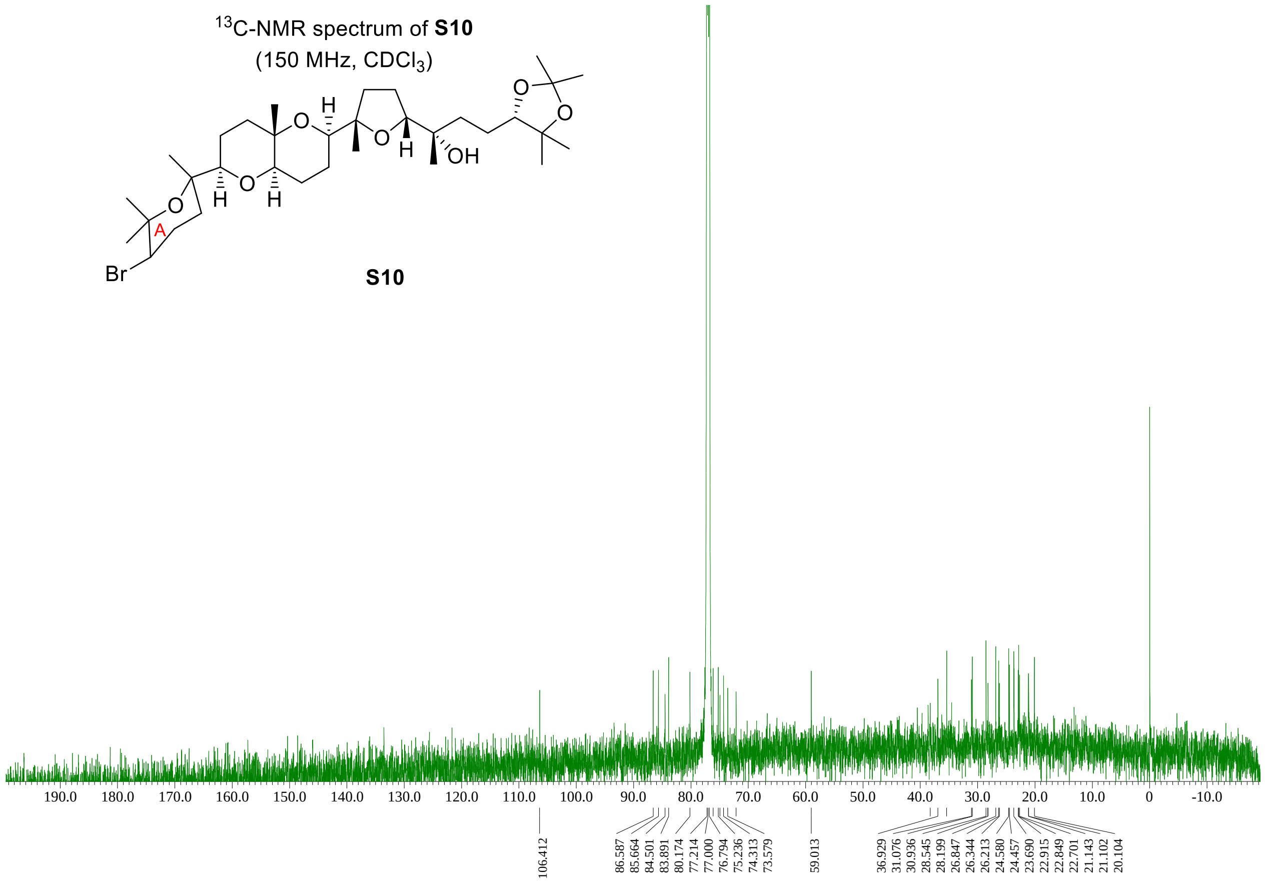

13C-NMR spectrum of **S10**
(150 MHz, CDCl3)
Br
A
O
H
OH
S10

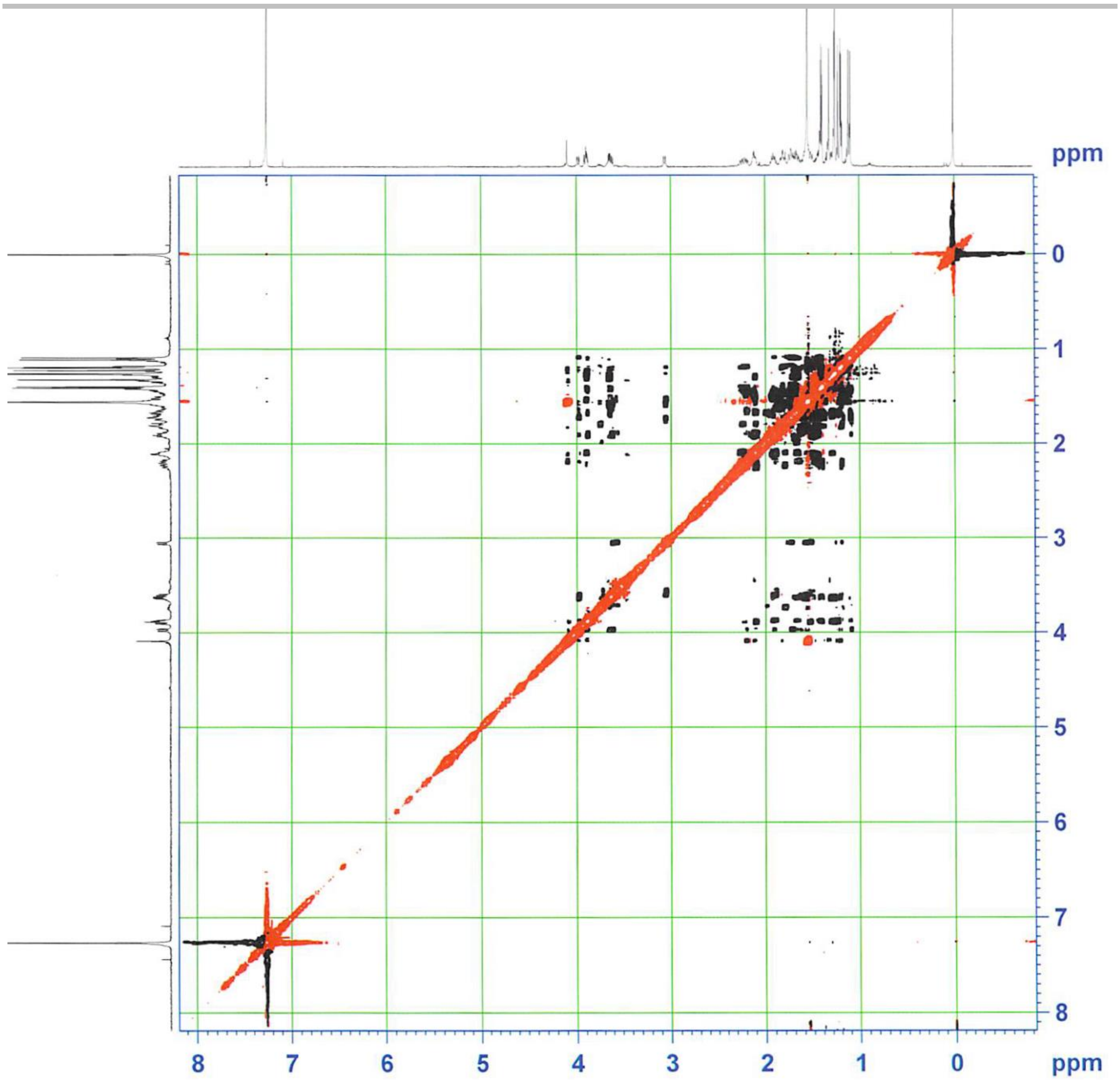

NOESY spectrum of **S10**
(25 °C)
($CDCl_3$)

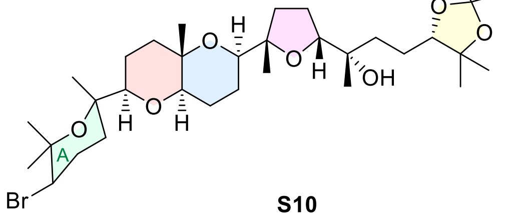

S10

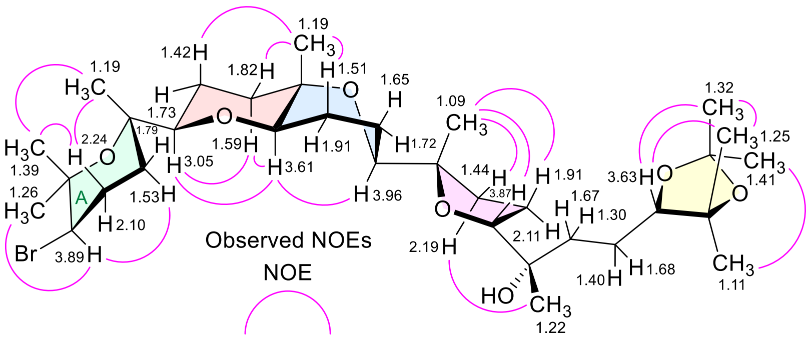

^{1}H-NMR spectrum of **S11**
(600 MHz, $CDCl_3$)

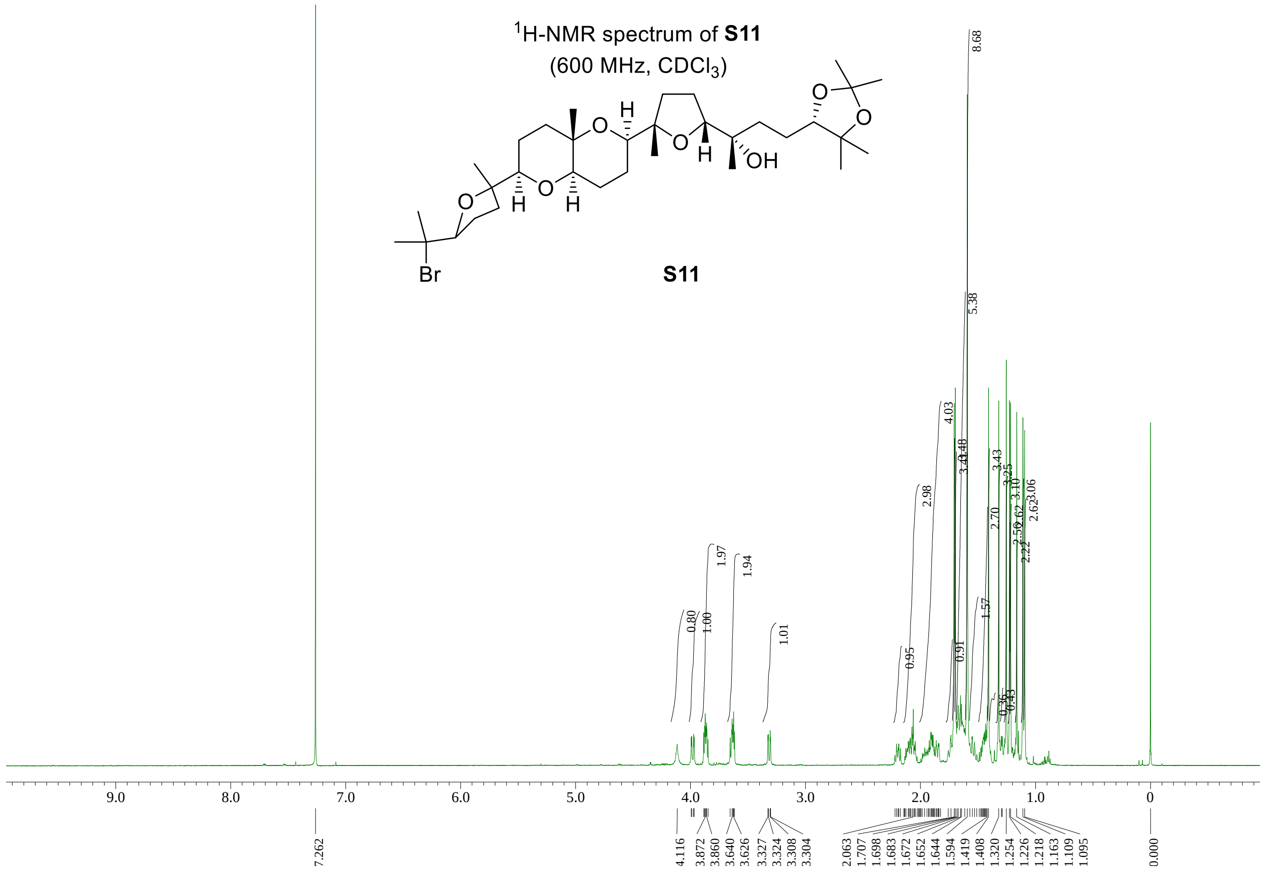

^{13}C-NMR spectrum of **S11**
(150 MHz, $CDCl_3$)

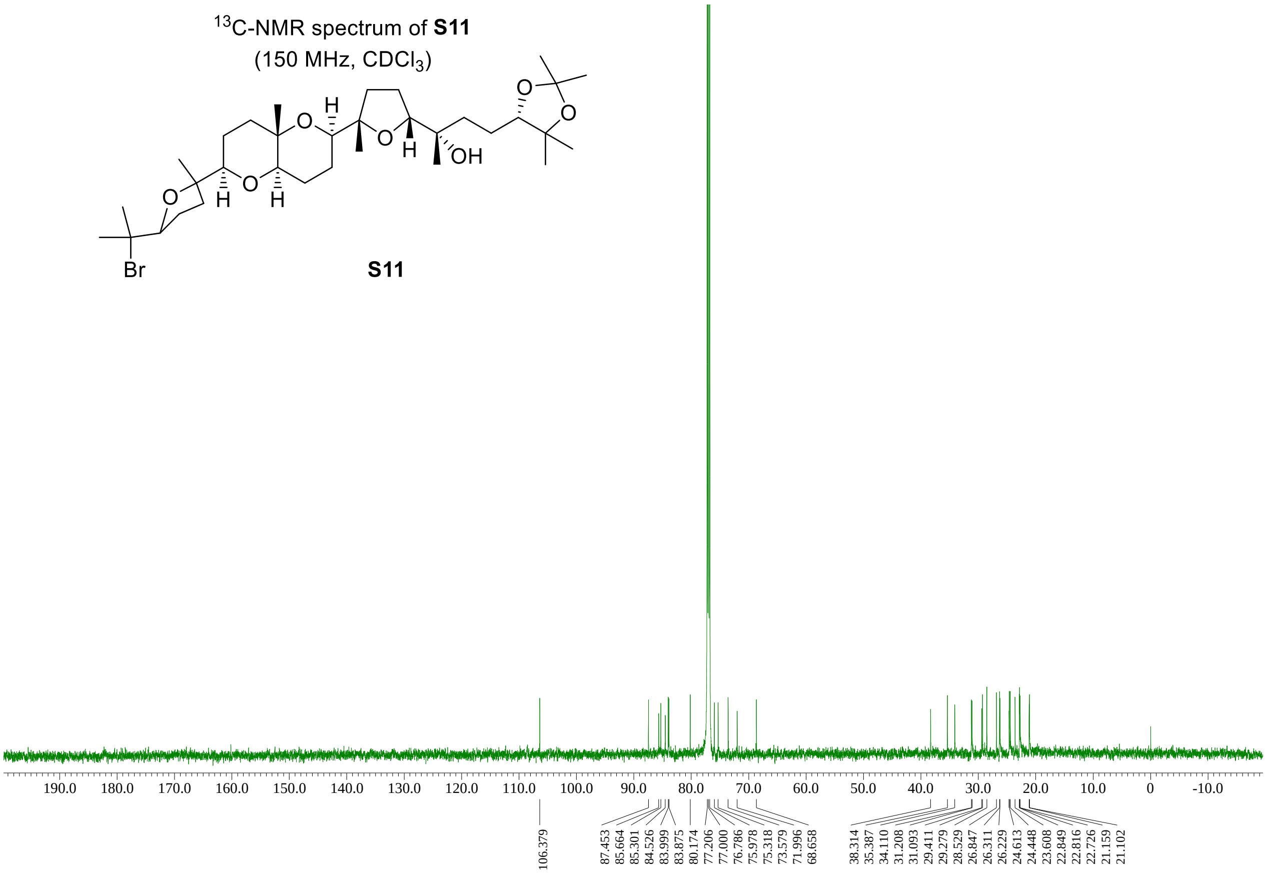

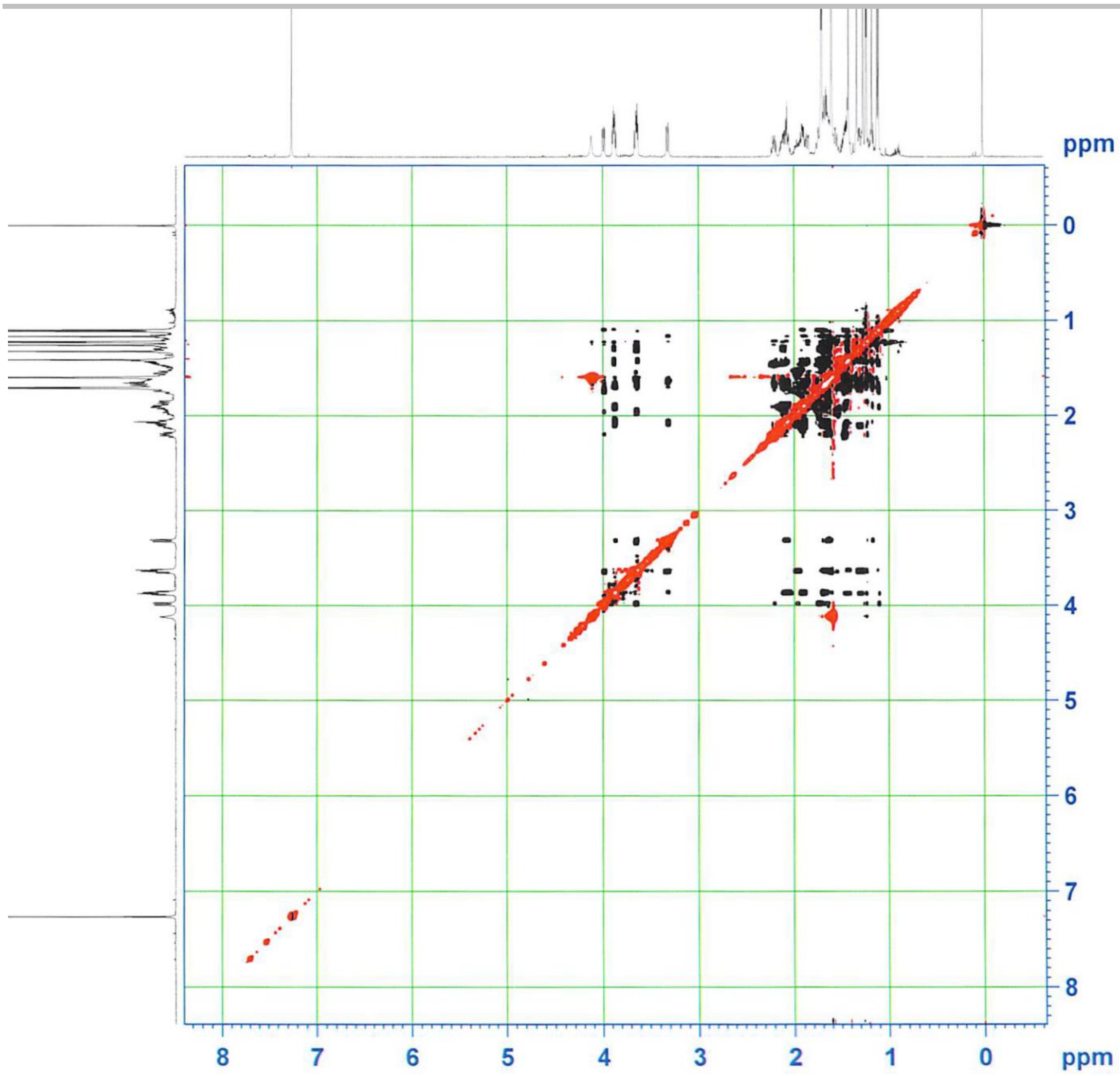

NOESY spectrum of **S11**
(25 °C)
($CDCl_3$)

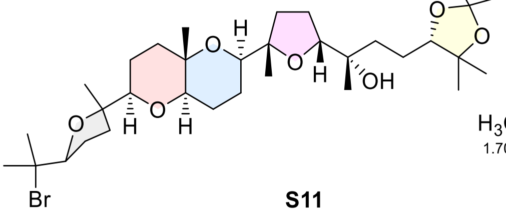

S11

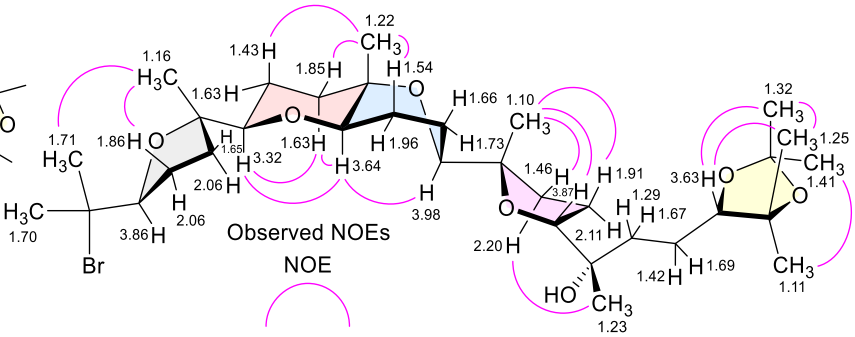

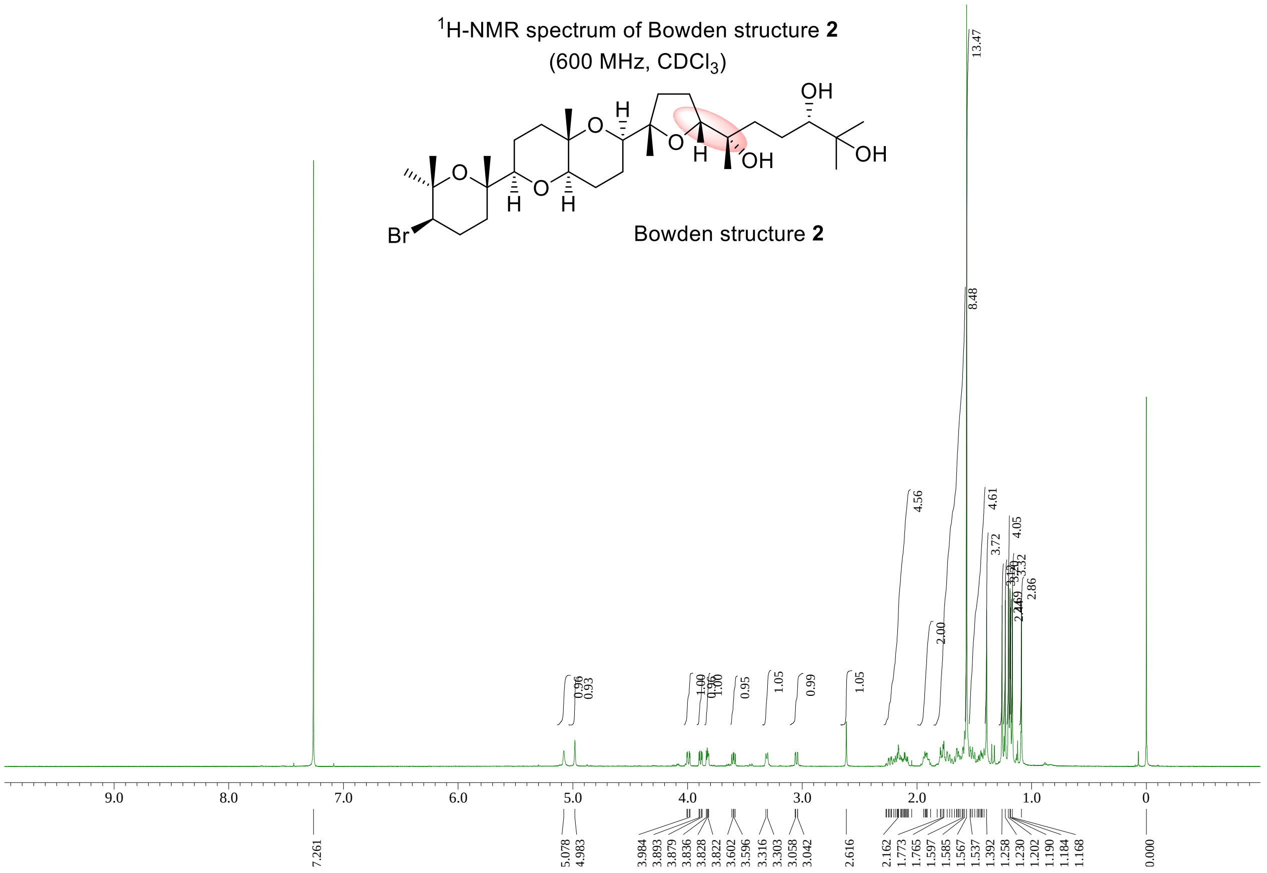
1H-NMR spectrum of Bowden structure 2
(600 MHz, CDCl3)
Bowden structure 2
9.0 8.0 7.0 6.0 5.0 4.0 3.0 2.0 1.0 0
7.261
5.078 4.983
3.984 3.893 3.879 3.836 3.828 3.822 3.602 3.596 3.316 3.303 3.058 3.042
2.616
2.162 1.773 1.765 1.597 1.585 1.567 1.537 1.392 1.258 1.230 1.202 1.190 1.184 1.168
0.000
13.47 8.48 4.56 4.61 3.72 4.05 3.32 2.86 2.00 0.96 0.93 1.00 1.00 0.95 1.05 0.99 1.05

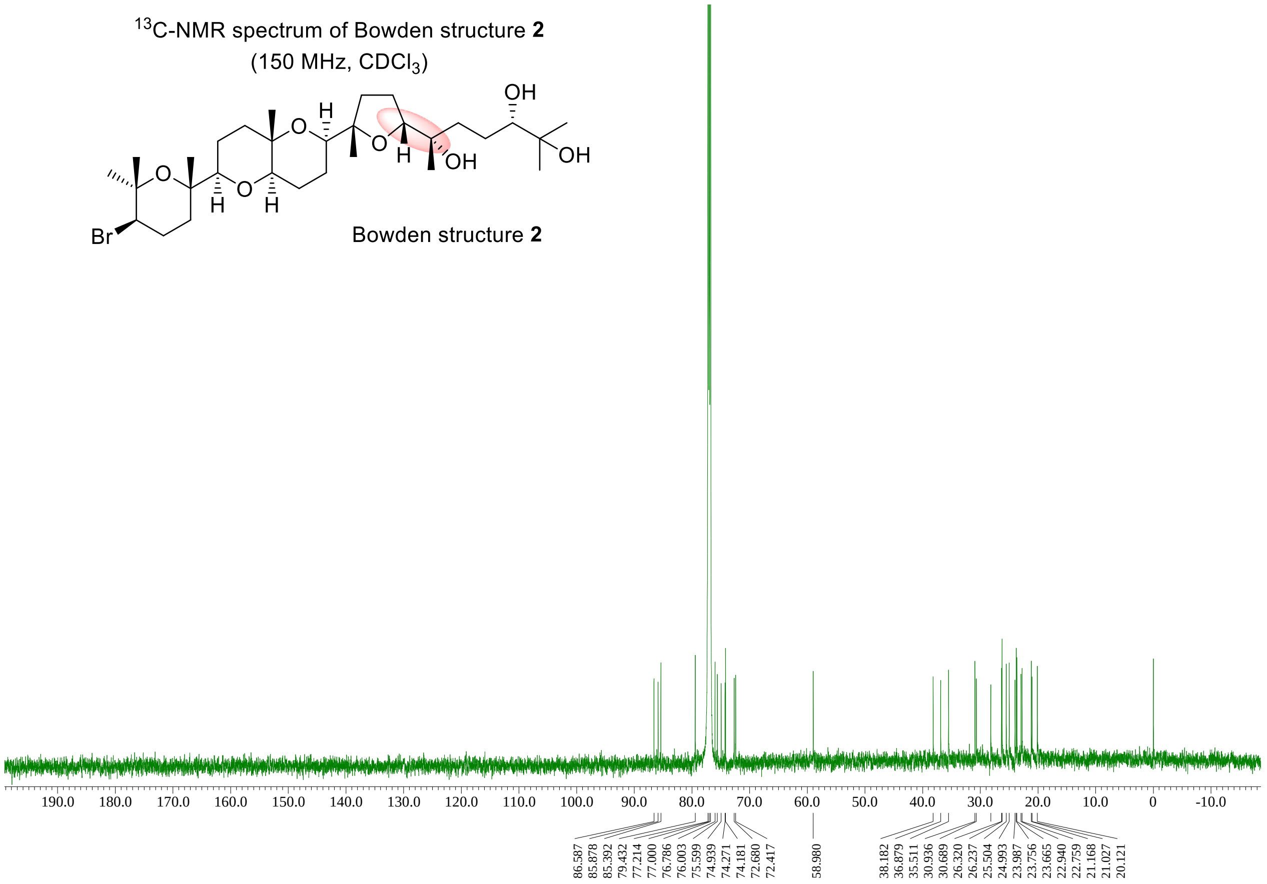
13C-NMR spectrum of Bowden structure 2
(150 MHz, CDCl3)
Bowden structure 2
190.0 180.0 170.0 160.0 150.0 140.0 130.0 120.0 110.0 100.0 90.0 80.0 70.0 60.0 50.0 40.0 30.0 20.0 10.0 0 -10.0
86.587 85.878 85.392 79.432 77.214 77.000 76.786 76.003 75.599 74.939 74.271 74.181 72.680 72.417
58.980
38.182 36.879 35.511 30.936 30.689 26.320 26.237 25.504 24.993 23.987 23.756 23.665 22.940 22.759 21.168 21.027 20.121

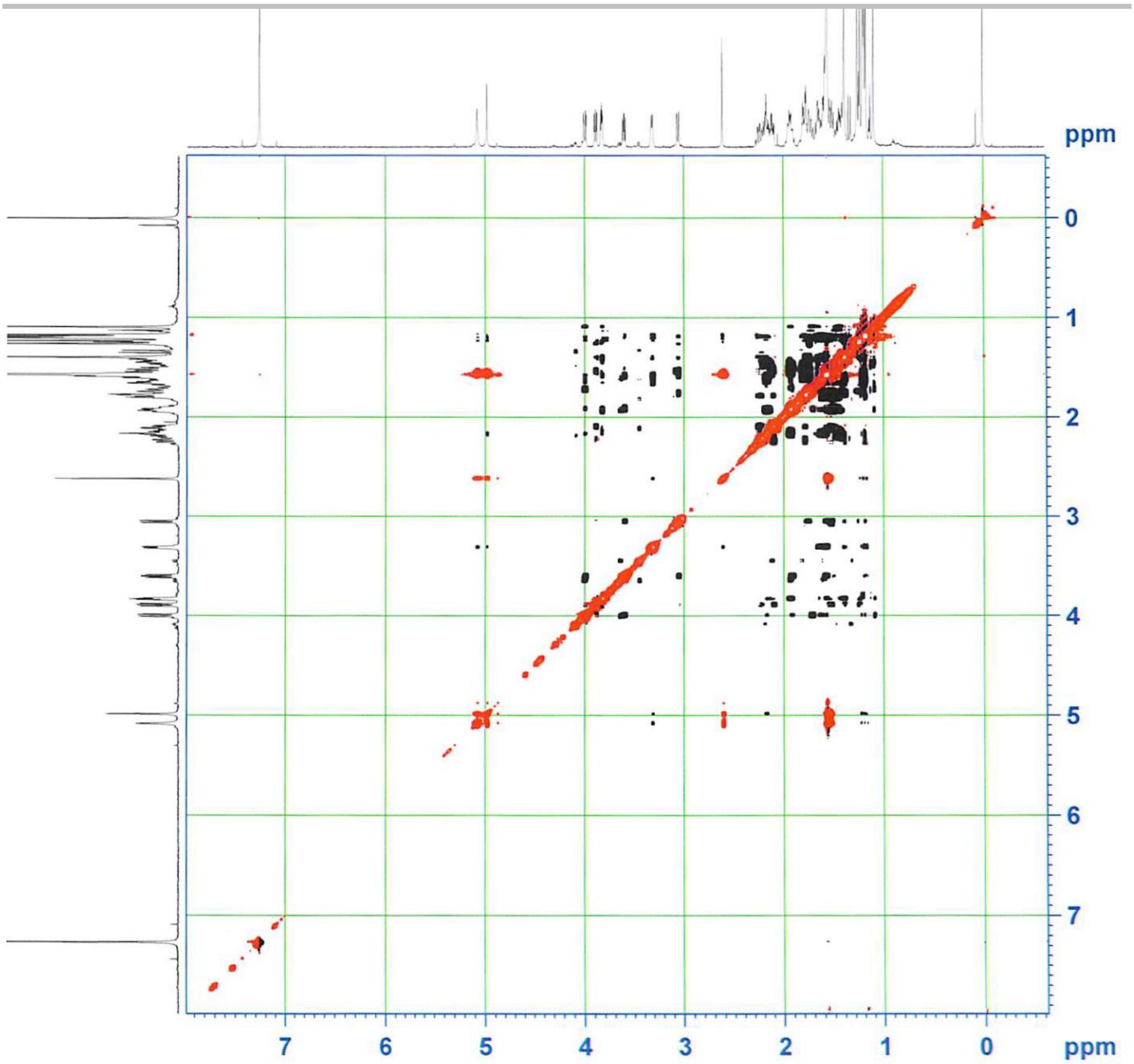

NOESY spectrum of Bowden structure **2**
(25 °C)
($CDCl_3$)

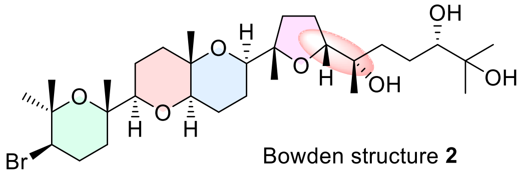

Bowden structure **2**

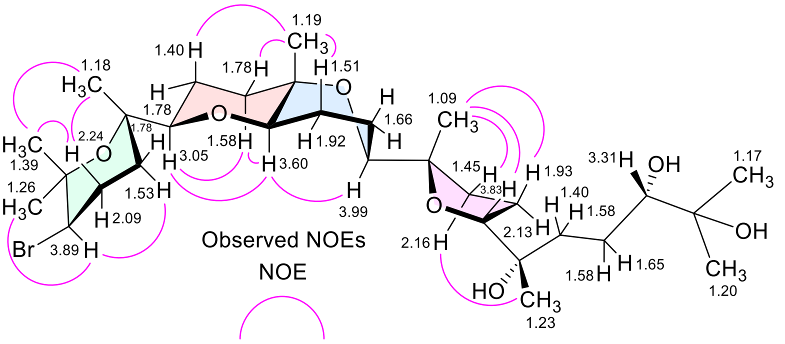

^{1}H-NMR spectrum of **S12**
(400 MHz, $CDCl_3$)

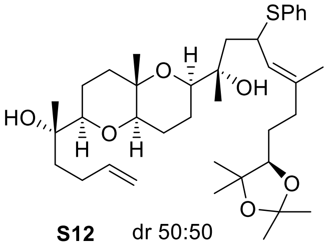

S12 dr 50:50

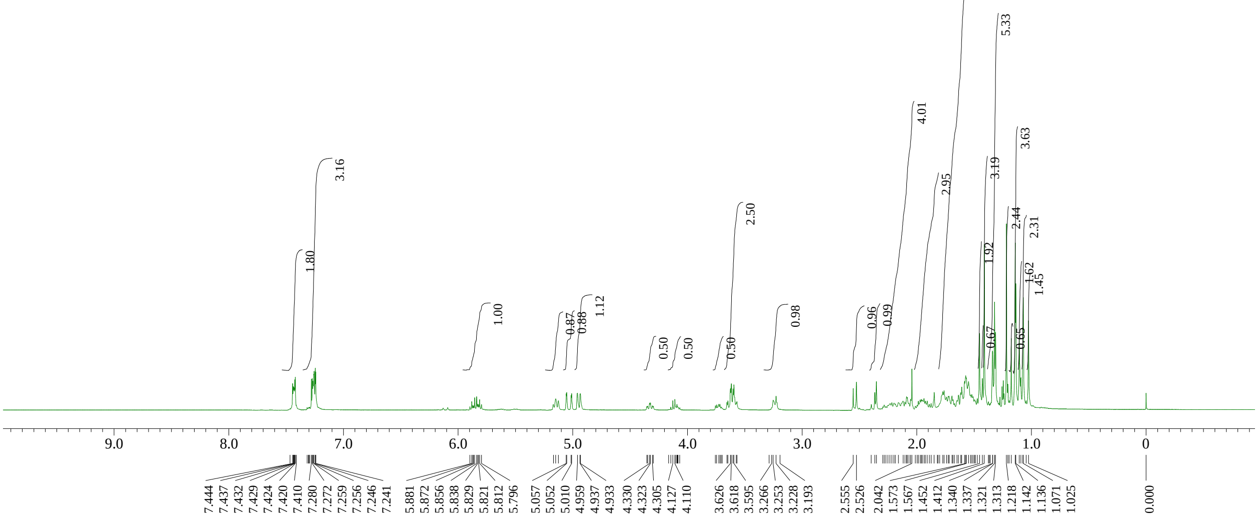

^{13}C-NMR spectrum of **S12**
(100 MHz, $CDCl_3$)

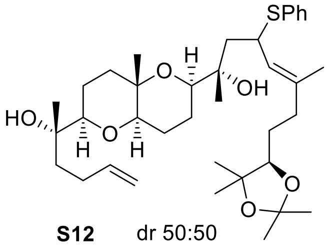

S12 dr 50:50

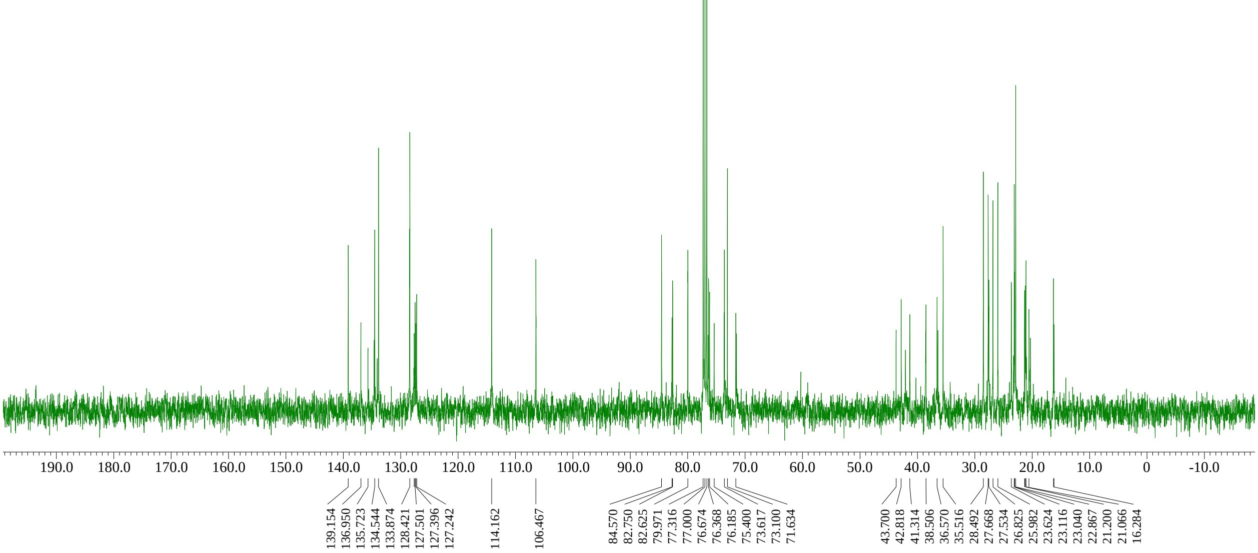

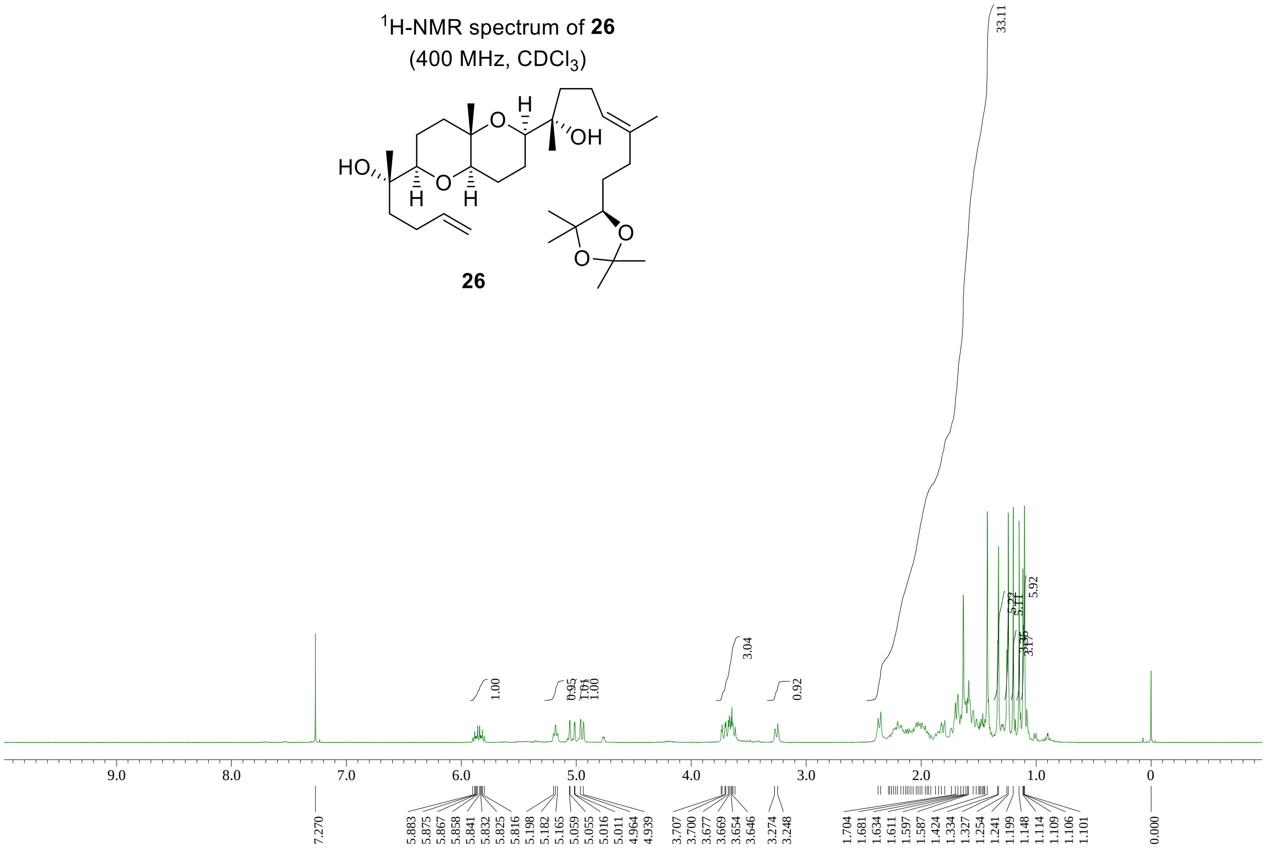
^{1}H-NMR spectrum of **26**
(400 MHz, $CDCl_3$)
26

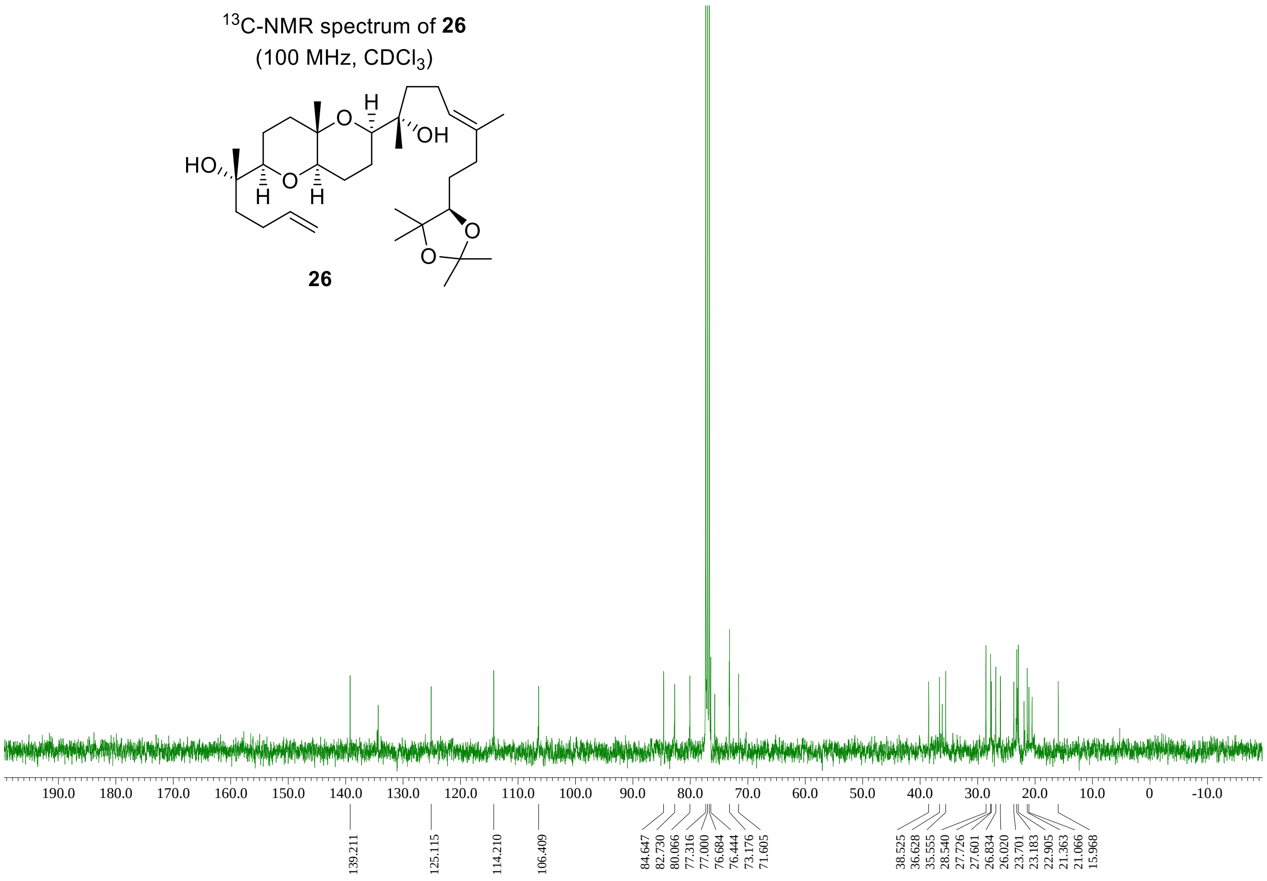
^{13}C-NMR spectrum of **26**
(100 MHz, $CDCl_3$)
26

^{1}H-NMR spectrum of **S13**
(400 MHz, $CDCl_3$)

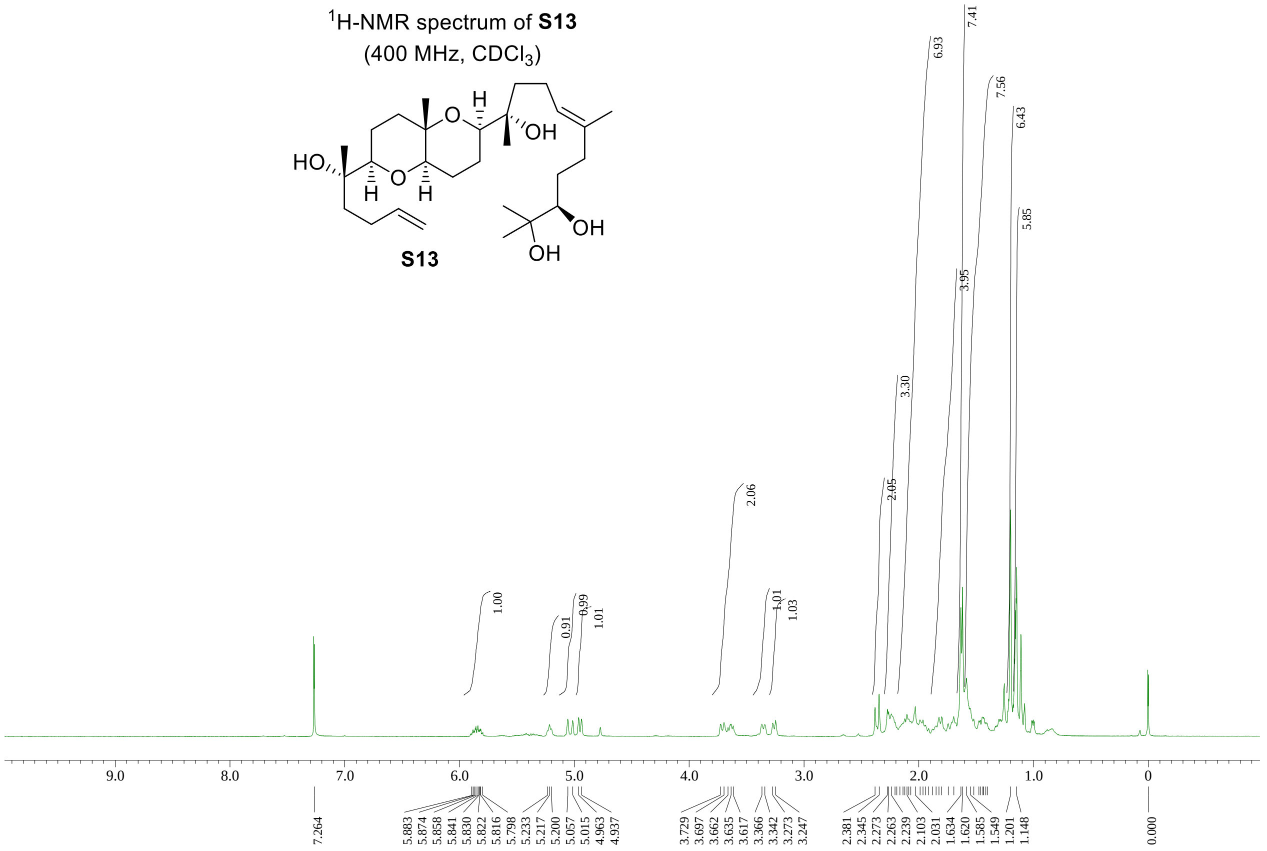

^{13}C-NMR spectrum of **S13**
(100 MHz, $CDCl_3$)

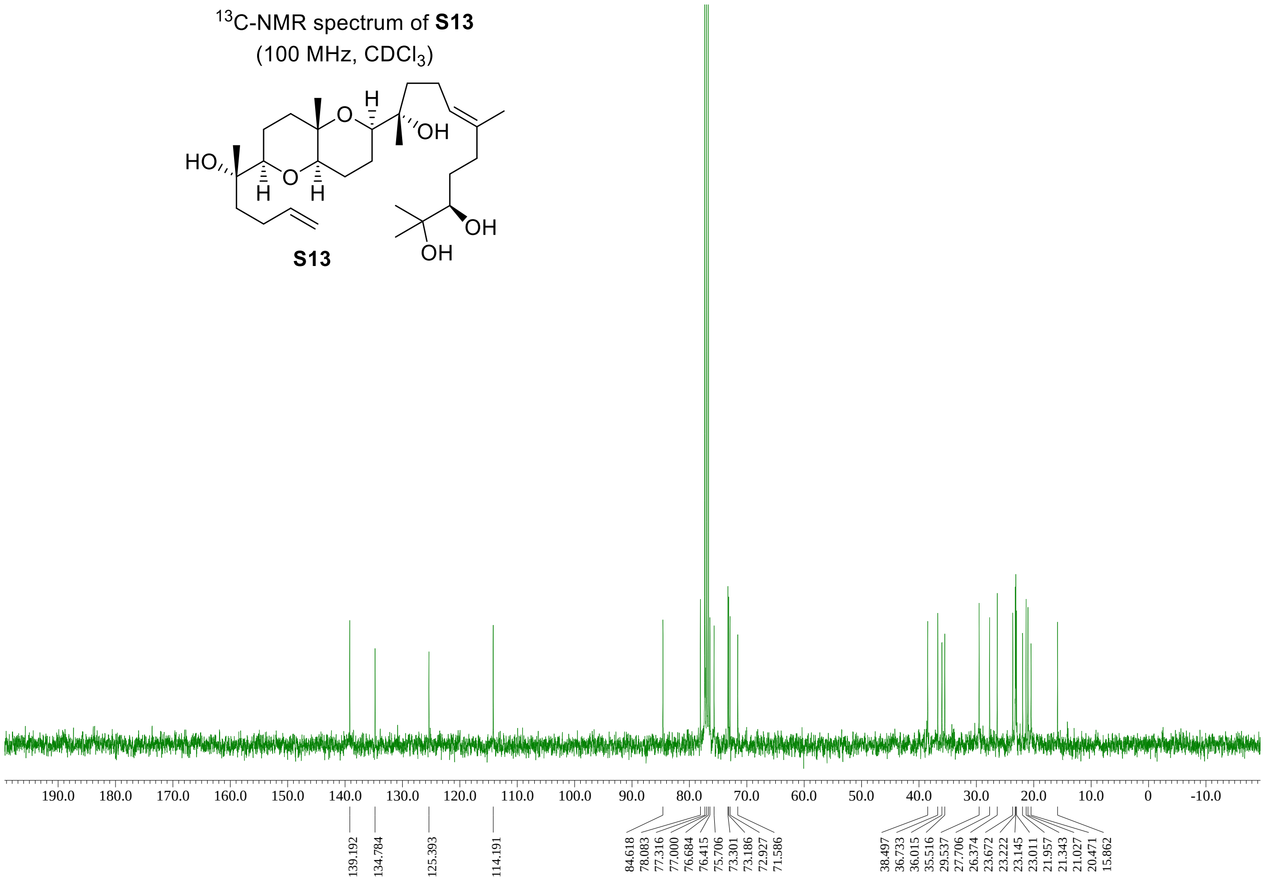

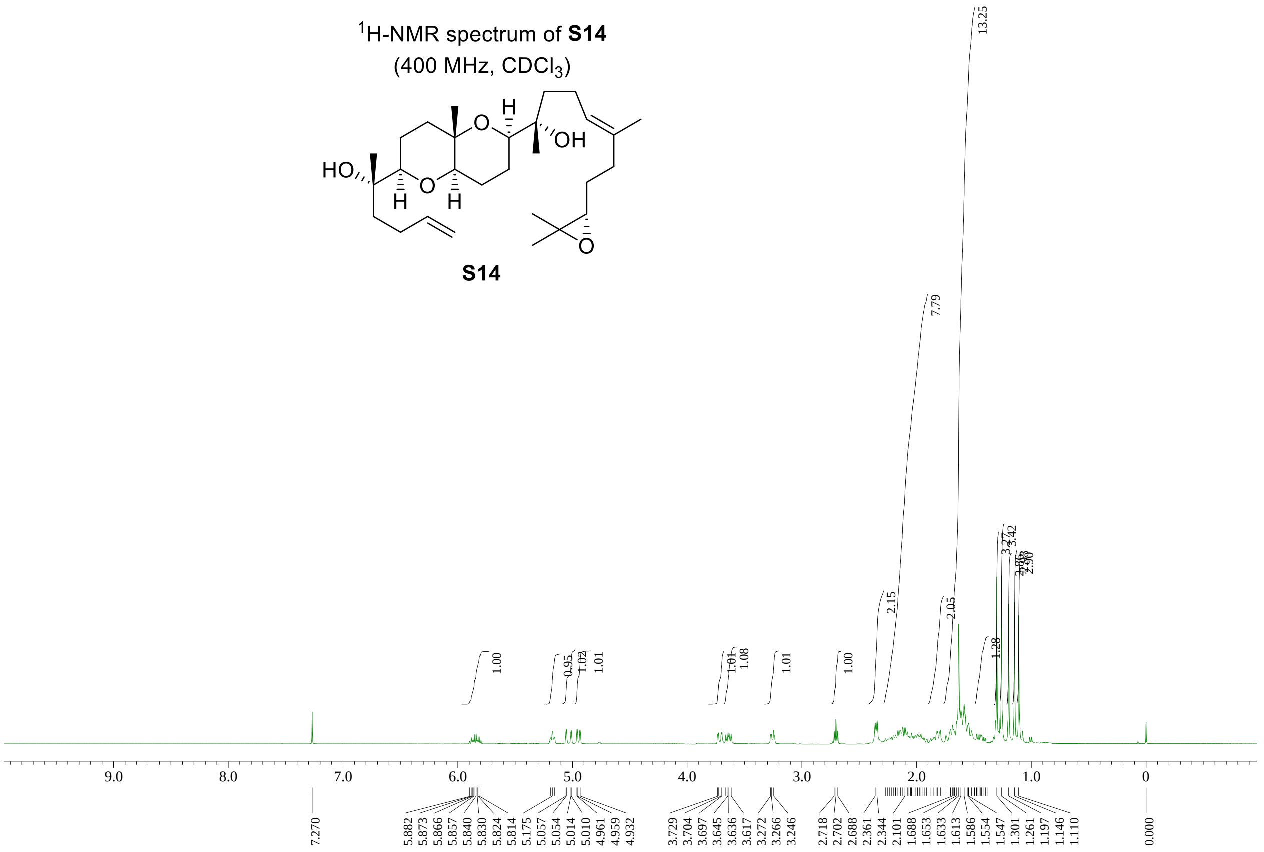
1H-NMR spectrum of S14
(400 MHz, CDCl3)
HO
OH
H
O
S14
1.00
0.95
1.02
1.01
1.01
1.08
1.01
1.00
2.15
7.79
2.05
1.28
13.25
3.42
9.0
8.0
7.0
6.0
5.0
4.0
3.0
2.0
1.0
0
7.270
5.882
5.873
5.866
5.857
5.840
5.830
5.824
5.814
5.175
5.057
5.054
5.014
5.010
4.961
4.959
4.932
3.729
3.704
3.697
3.645
3.636
3.617
3.272
3.266
3.246
2.718
2.702
2.688
2.361
2.344
2.101
1.688
1.653
1.633
1.613
1.586
1.554
1.547
1.301
1.261
1.197
1.146
1.110
0.000

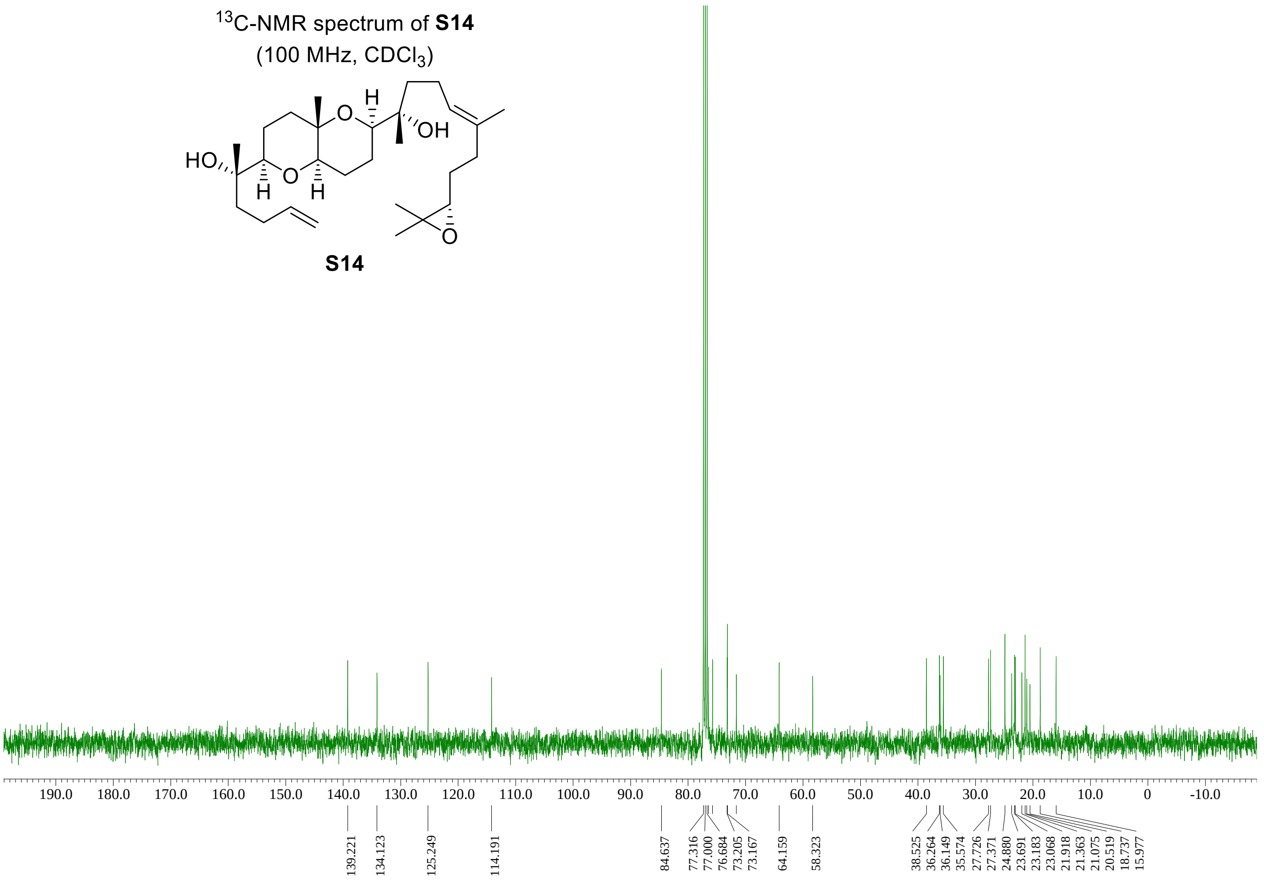
13C-NMR spectrum of S14
(100 MHz, CDCl3)
HO
OH
H
O
S14
190.0
180.0
170.0
160.0
150.0
140.0
130.0
120.0
110.0
100.0
90.0
80.0
70.0
60.0
50.0
40.0
30.0
20.0
10.0
0
-10.0
139.221
134.123
125.249
114.191
84.637
77.316
77.000
76.684
73.205
73.167
64.159
58.323
38.525
36.264
36.149
35.574
27.726
27.371
24.880
23.691
23.183
23.068
21.918
21.363
21.075
20.519
18.737
15.977

^{1}H-NMR spectrum of **27**
(400 MHz, $CDCl_3$)

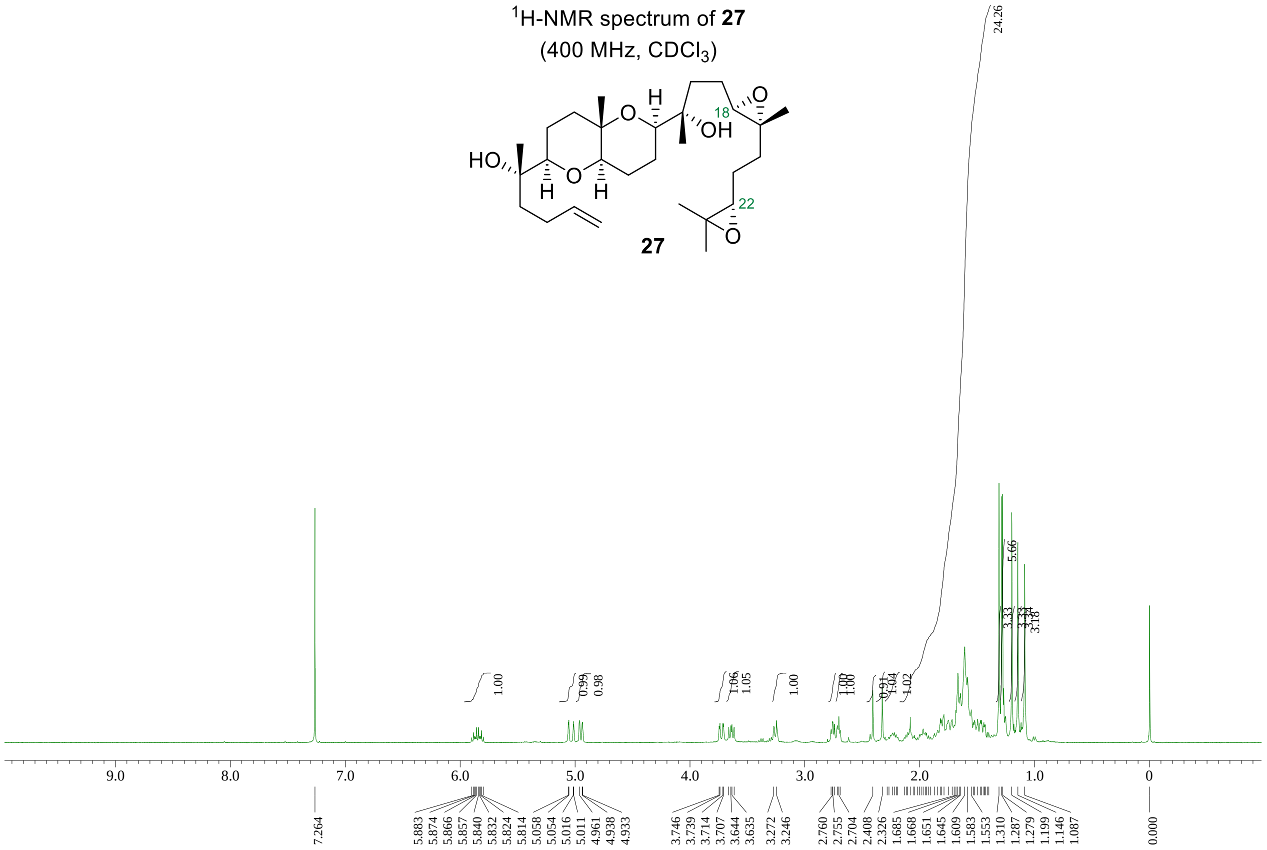

^{13}C-NMR spectrum of **27**
(100 MHz, $CDCl_3$)

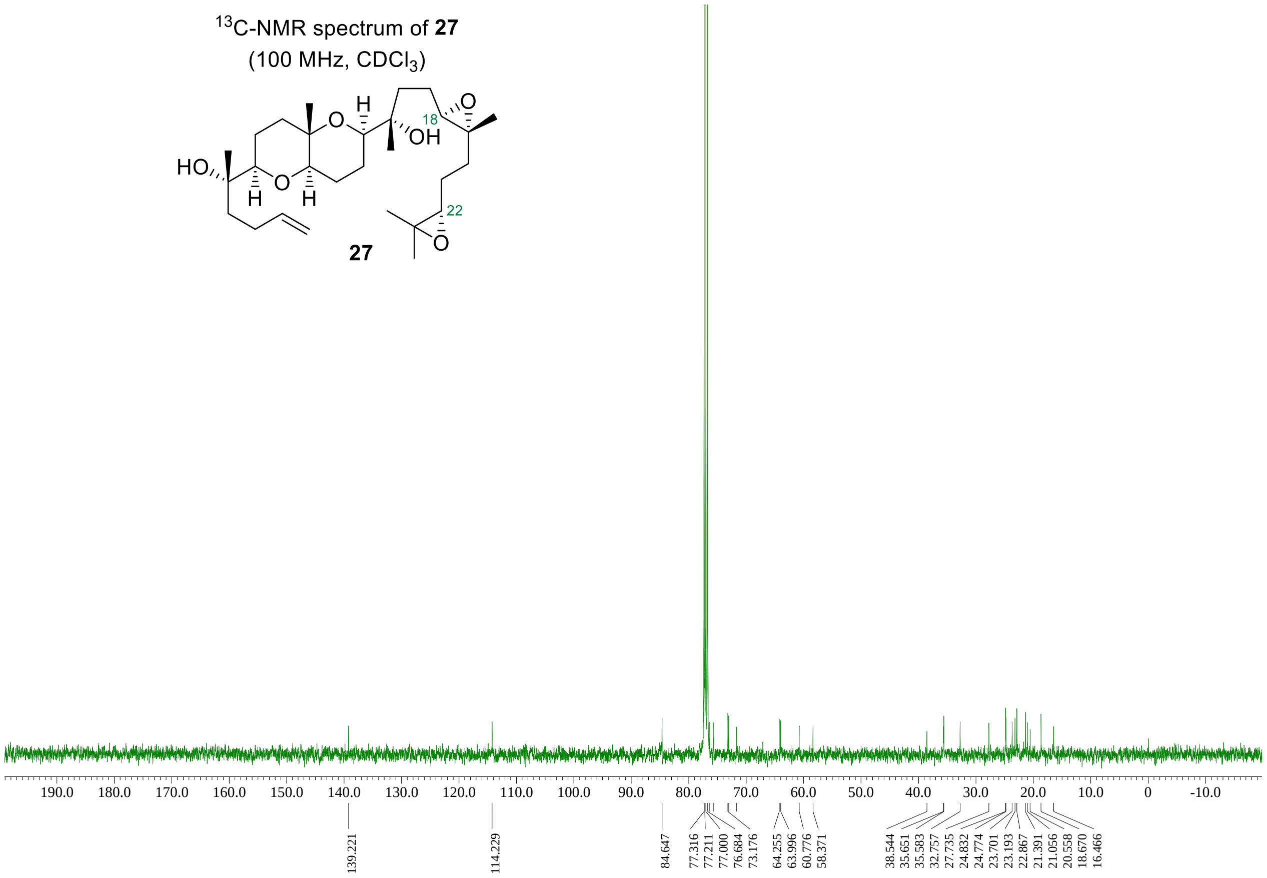

^{1}H-NMR spectrum of **28**
(600 MHz, $CDCl_3$)

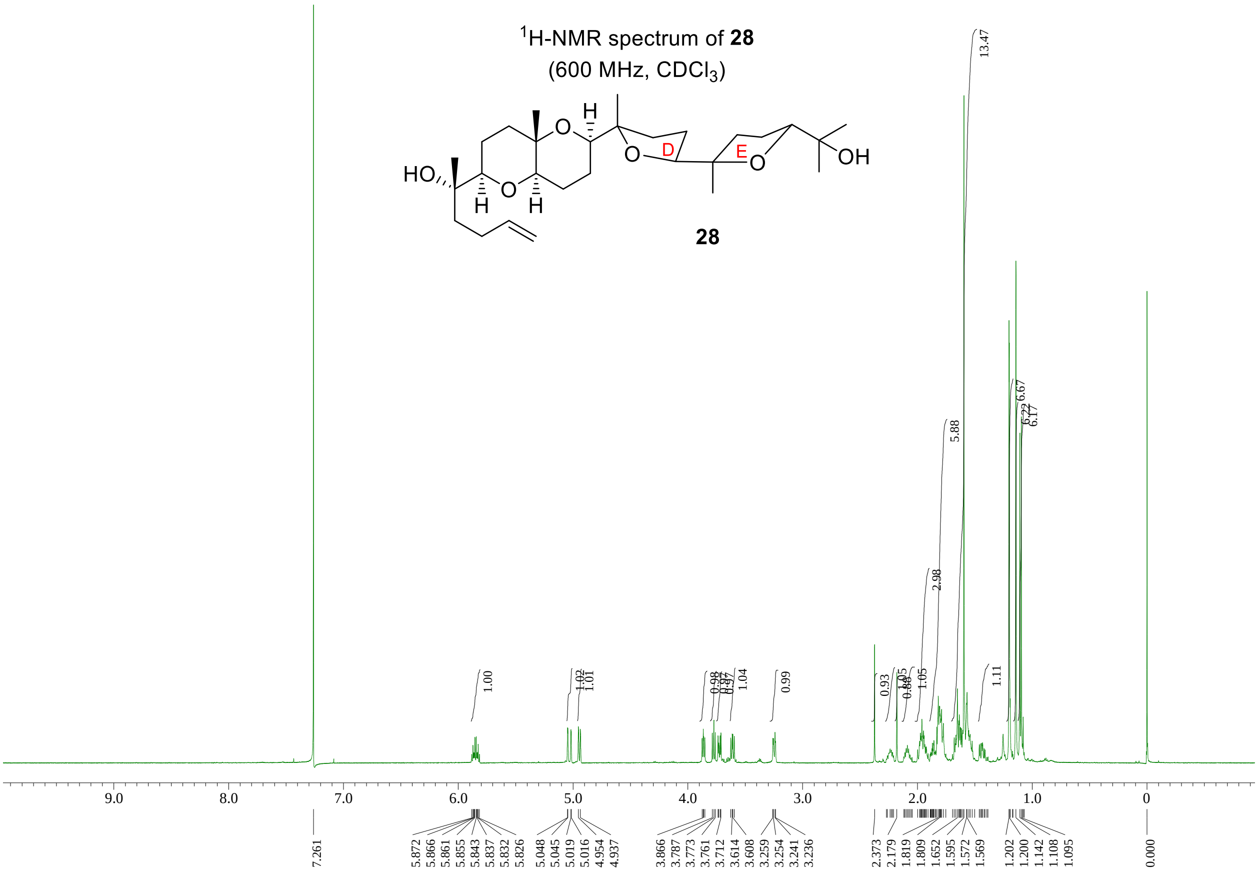

^{13}C-NMR spectrum of **28**
(150 MHz, $CDCl_3$)

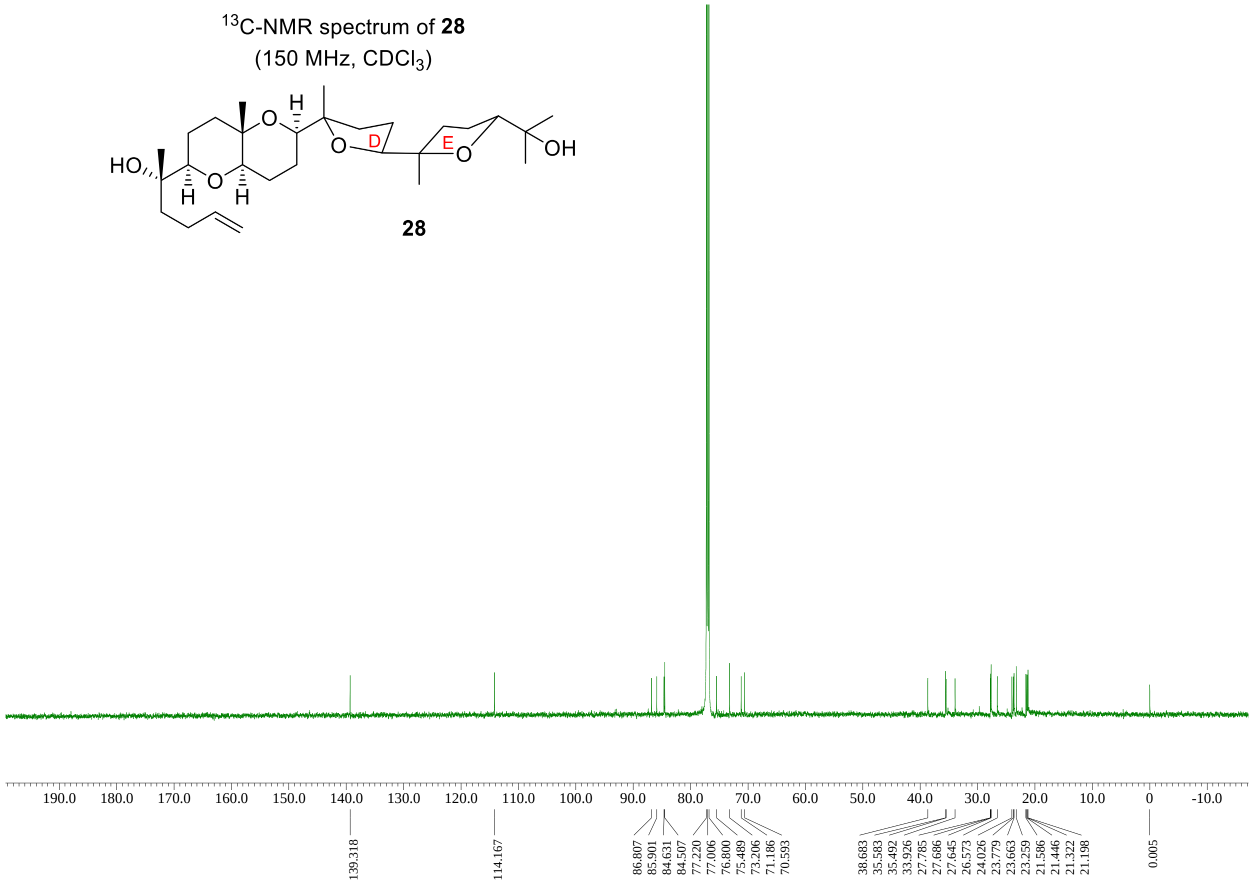

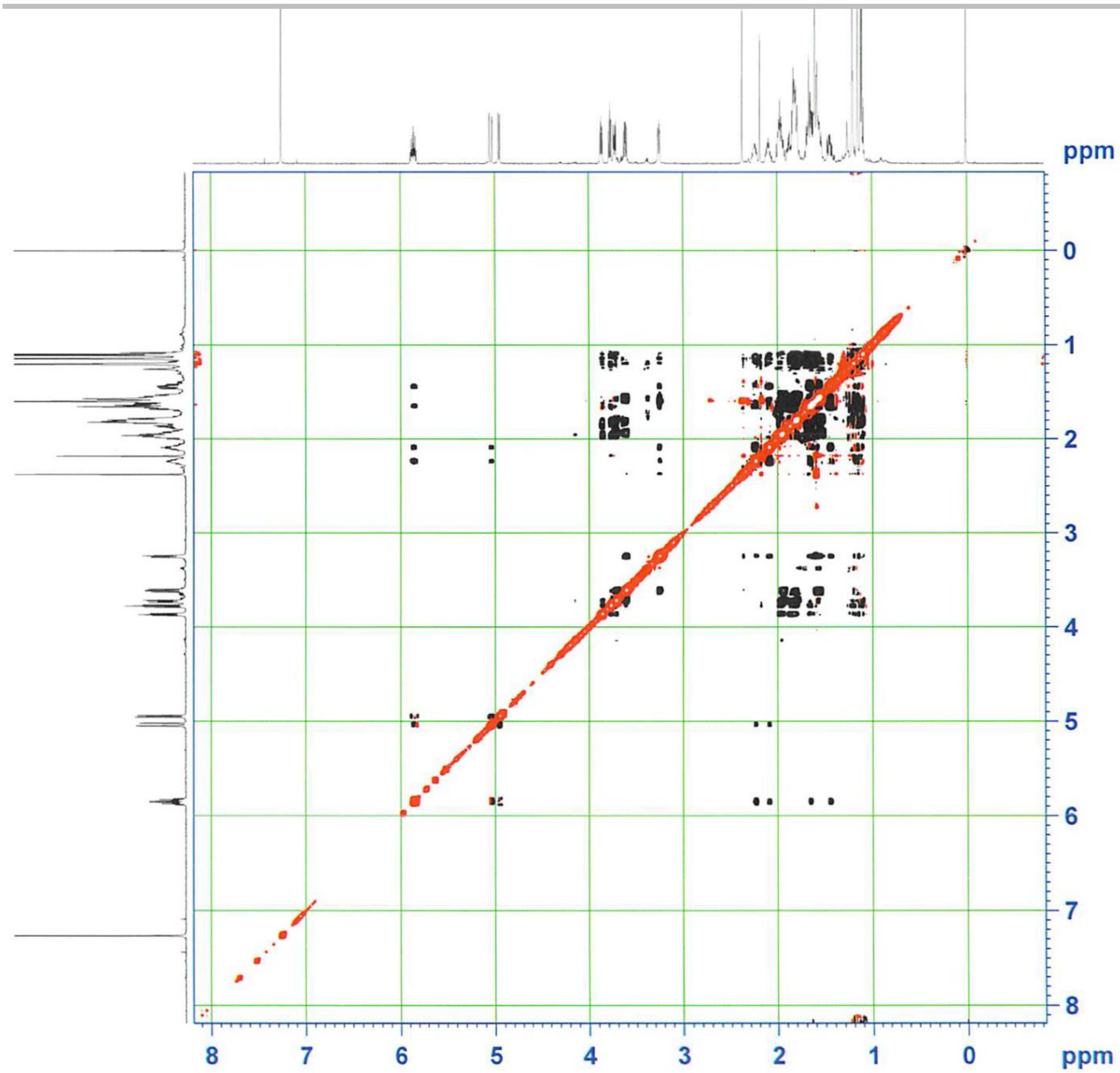

NOESY spectrum of **28**
(25 °C)
($CDCl_3$)

Observed NOEs
NOE

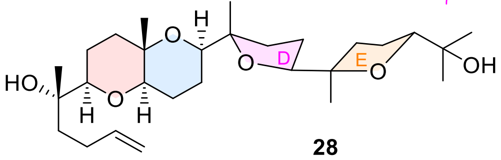

28

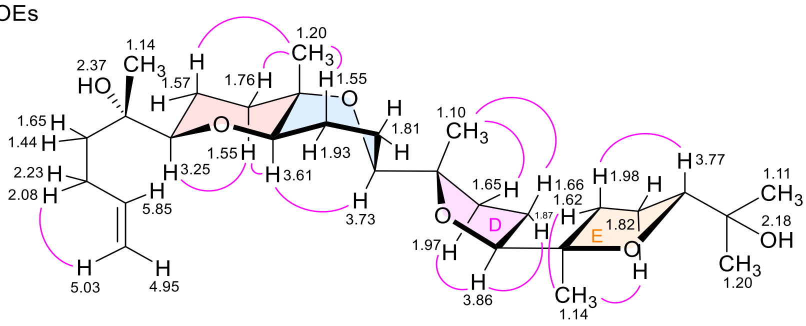

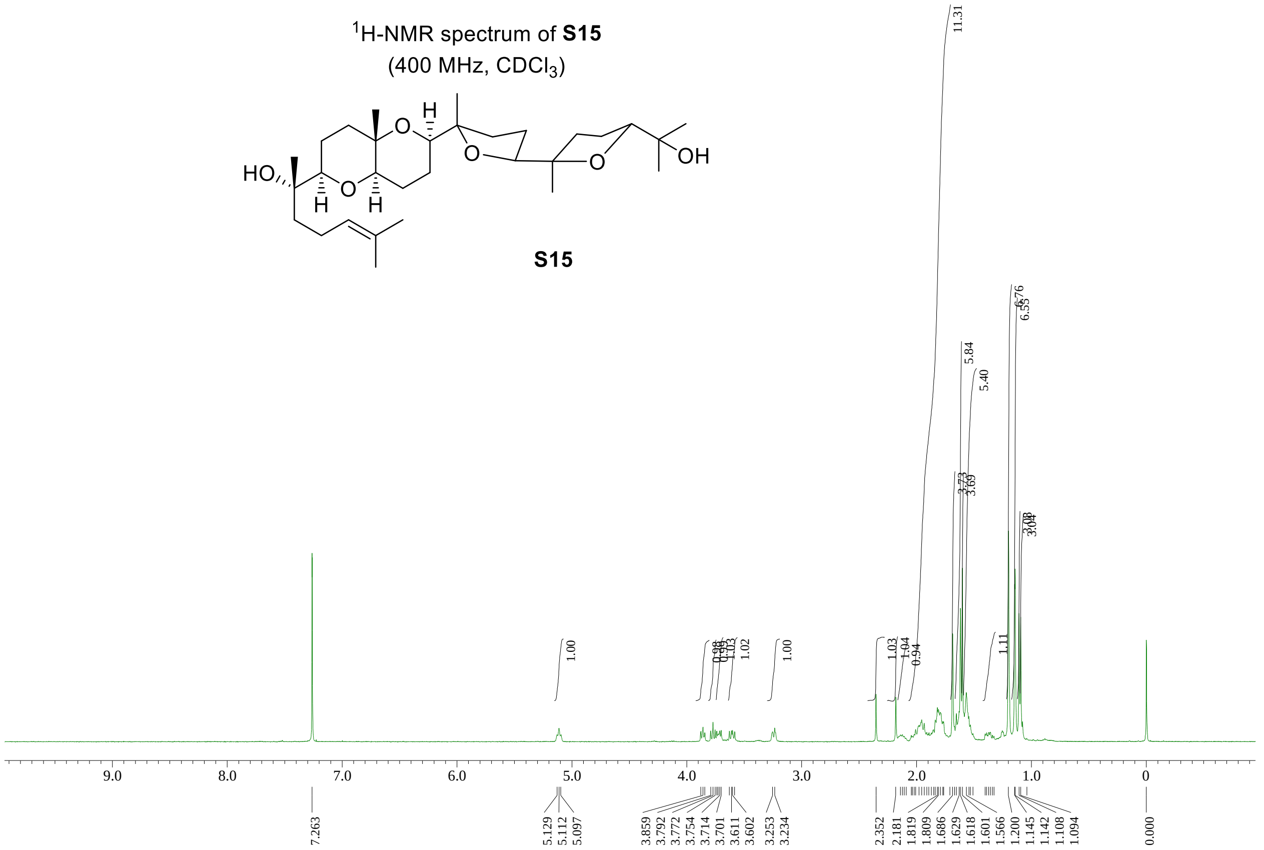
^{1}H-NMR spectrum of **S15**
(400 MHz, CDCl$_3$)
HO
OH
S15
9.0 8.0 7.0 6.0 5.0 4.0 3.0 2.0 1.0 0
7.263
5.129 5.112 5.097
3.859 3.792 3.772 3.754 3.714 3.701 3.611 3.602
3.253 3.234
2.352 2.181 1.819 1.809 1.686 1.629 1.618 1.601 1.566 1.200 1.145 1.142 1.108 1.094
0.000

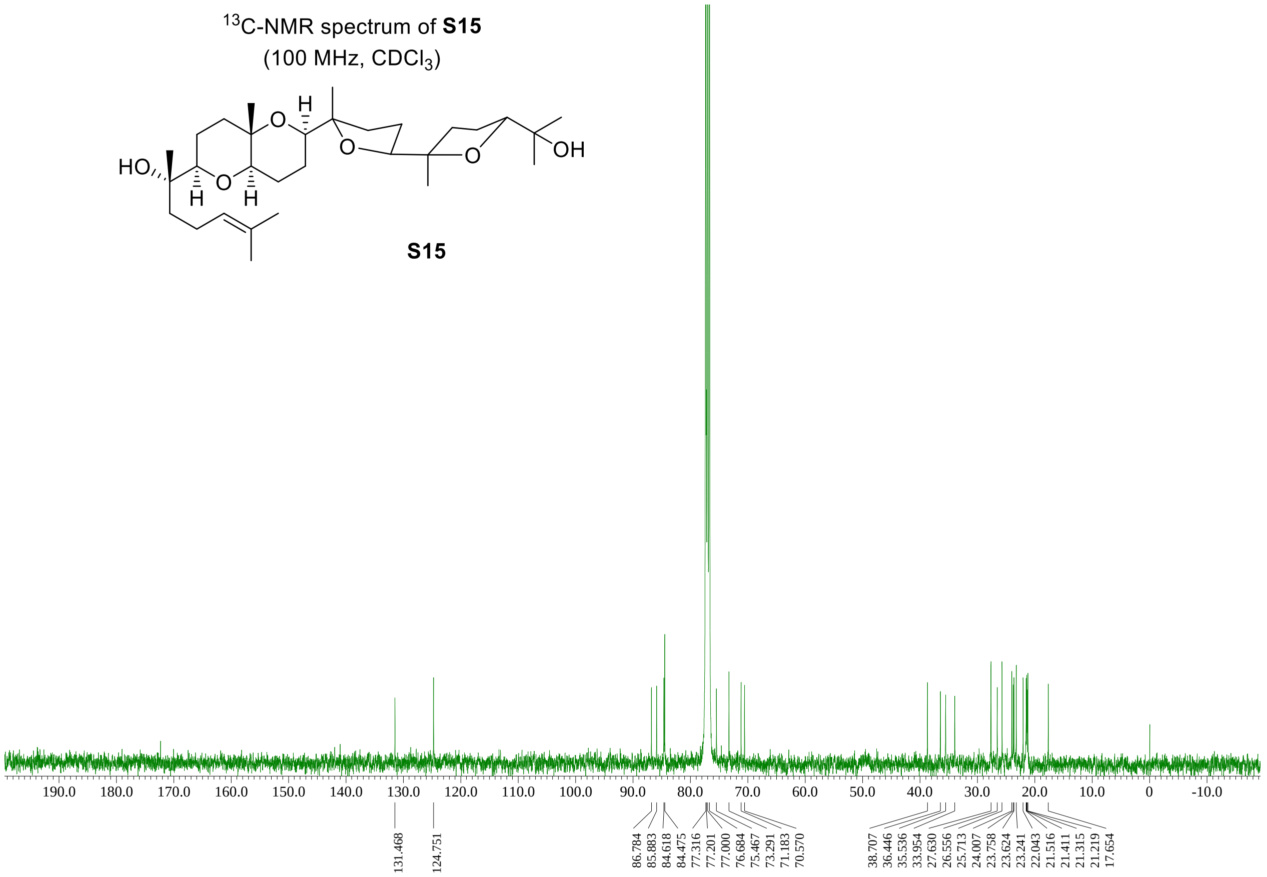
^{13}C-NMR spectrum of **S15**
(100 MHz, CDCl$_3$)
HO
OH
S15
190.0 180.0 170.0 160.0 150.0 140.0 130.0 120.0 110.0 100.0 90.0 80.0 70.0 60.0 50.0 40.0 30.0 20.0 10.0 0 -10.0
131.468
124.751
86.784 85.883 84.618 84.475 77.316 77.201 77.000 76.684 75.467 73.291 71.183 70.570
38.707 36.446 35.536 33.954 27.630 26.556 25.713 24.007 23.758 23.624 23.241 22.043 21.516 21.411 21.315 21.219 17.654

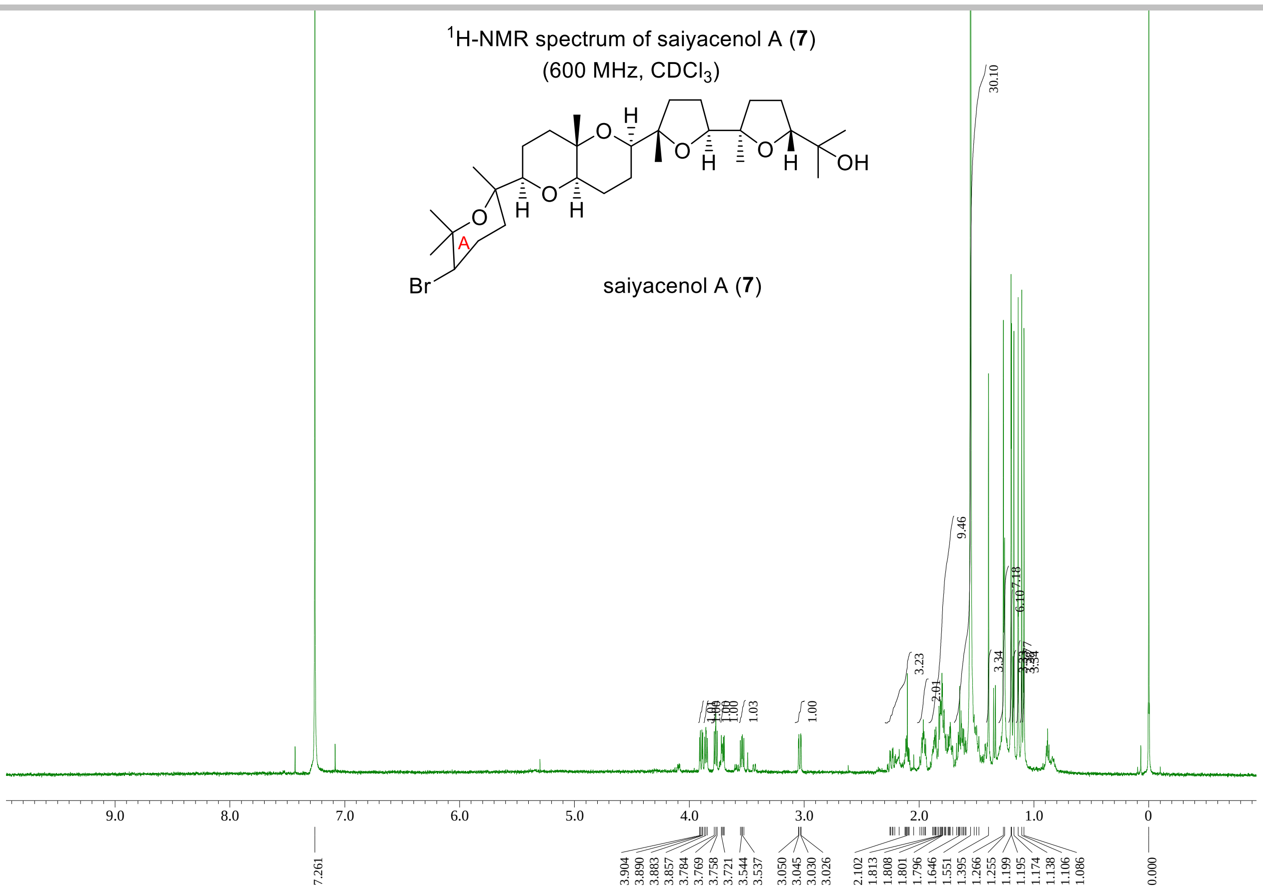
1H-NMR spectrum of saiyacenol A (7)
(600 MHz, CDCl3)
saiyacenol A (7)

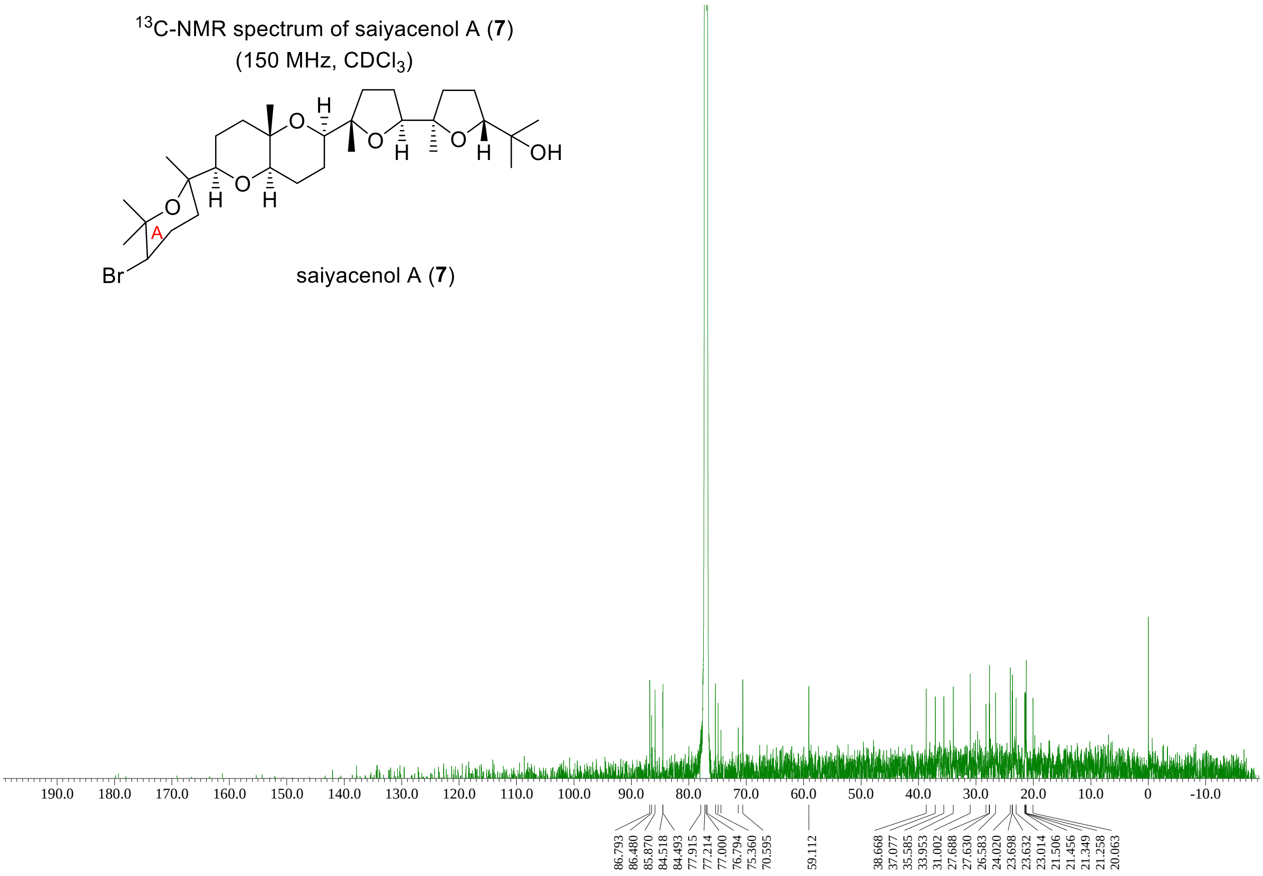
13C-NMR spectrum of saiyacenol A (7)
(150 MHz, CDCl3)
saiyacenol A (7)

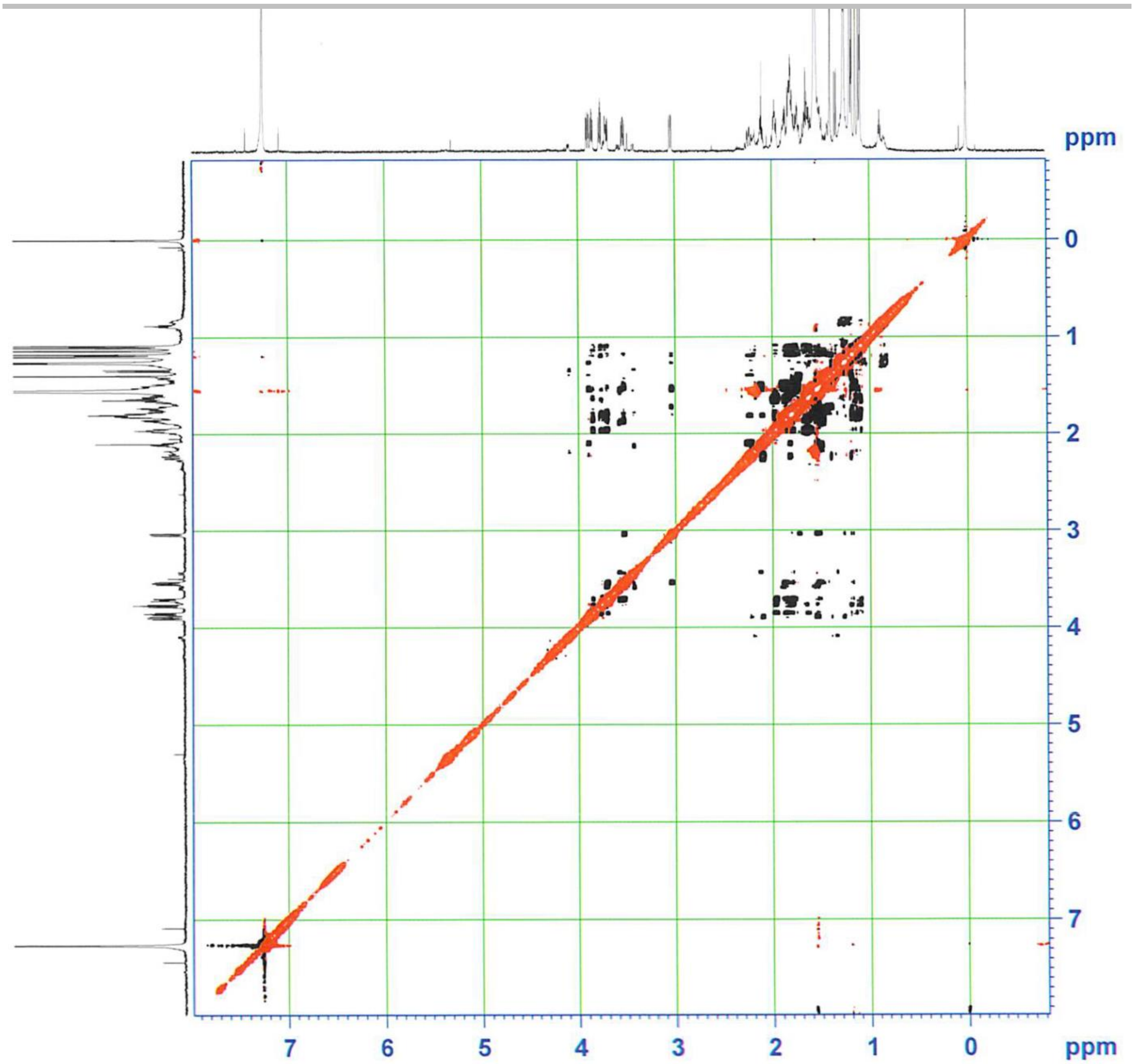

NOESY spectrum of saiyacenol A (**7**)
(25 °C)
($CDCl_3$)

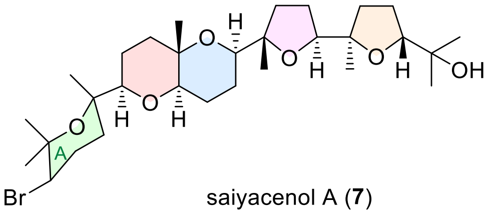

saiyacenol A (**7**)

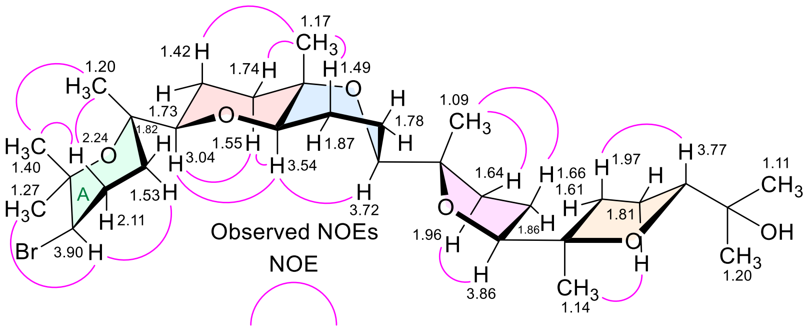

^{1}H-NMR spectrum of **S16**
(600 MHz, $CDCl_3$)

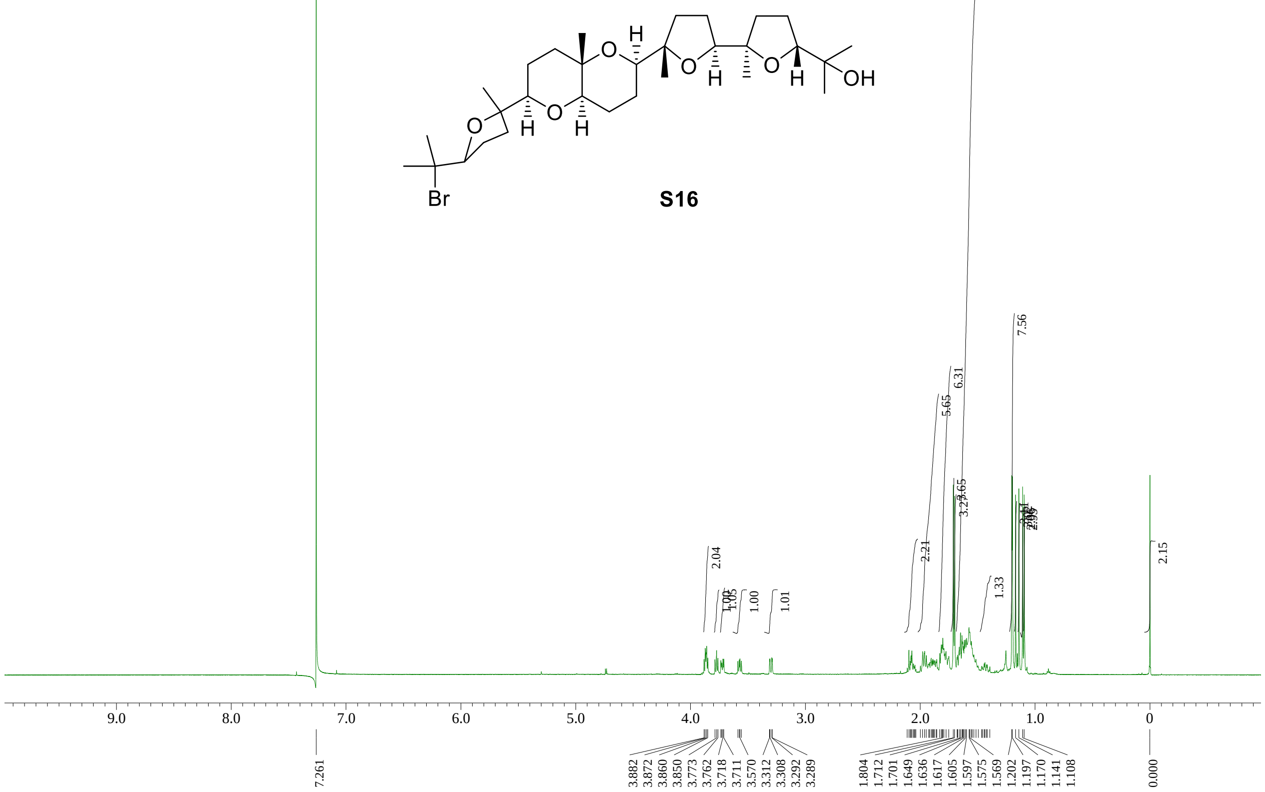

^{13}C-NMR spectrum of **S16**
(150 MHz, $CDCl_3$)

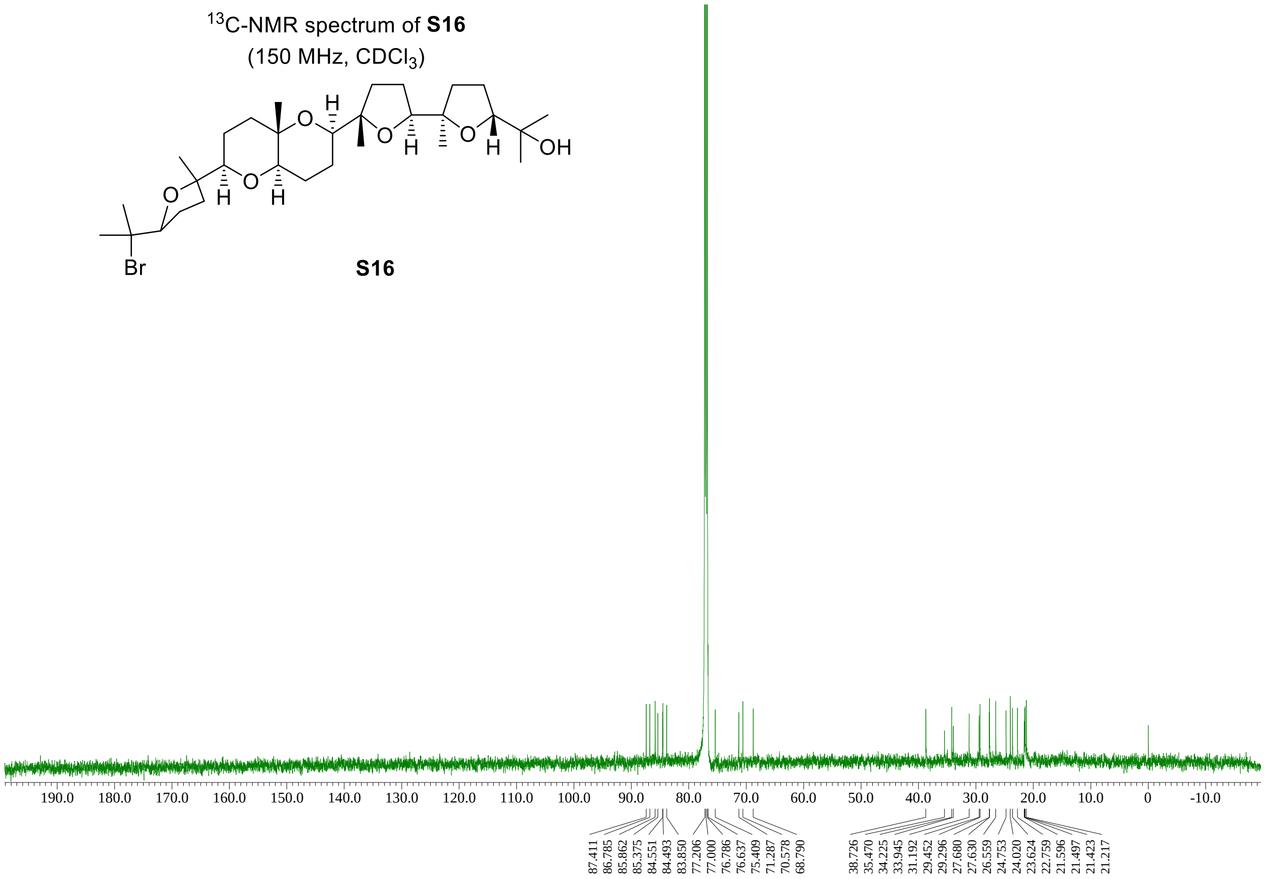

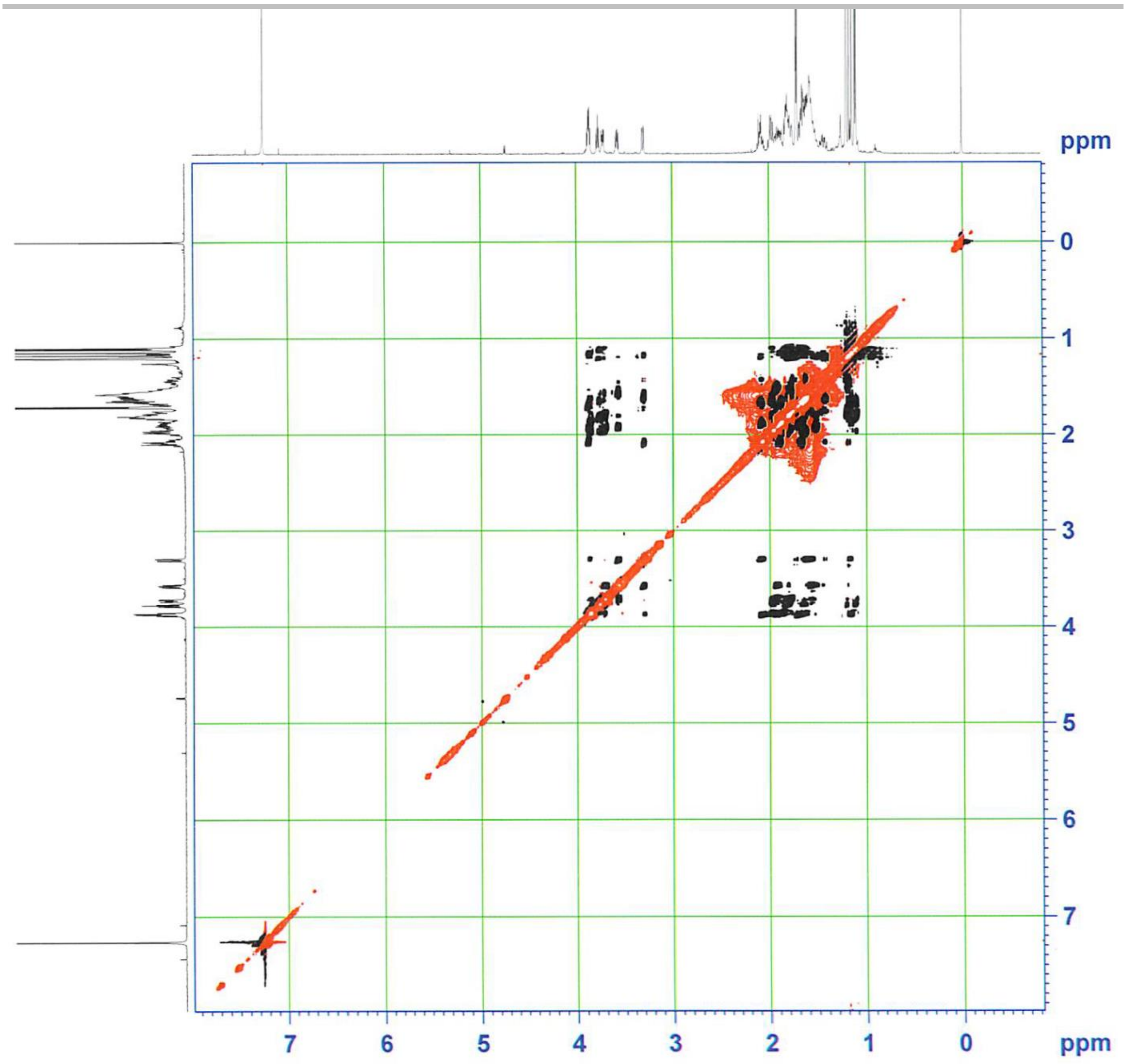

NOESY spectrum of **S16**
(25 °C)
($CDCl_3$)

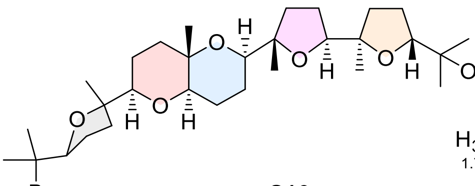

S16

Observed NOEs
NOE

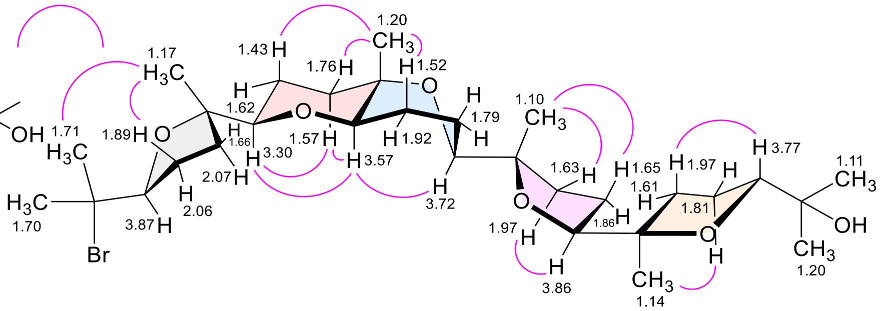

Supplement: Supplementary file 1 — Supporting Information [file ASIA-17-0-s001.pdf]
